# Fluorinated Analogues to the Pentuloses of the Pentose Phosphate Pathway

**DOI:** 10.1002/ejoc.202300339

**Published:** 2023-06-02

**Authors:** Lukas Scheibelberger, Toda Stankovic, Kaja Liepert, Andreas Kienzle, Eva‐Maria Patronas, Theresa Balber, Markus Mitterhauser, Arvand Haschemi, Katharina Pallitsch

**Affiliations:** ^1^ Institute of Organic Chemistry University of Vienna Währinger Straße 38 1090 Vienna Austria; ^2^ Vienna Doctoral School in Chemistry (DoSChem) University of Vienna Währinger Straße 42 1090 Vienna Austria; ^3^ Division of Nuclear Medicine Department of Biomedical Imaging and Image-guided Therapy Medical University of Vienna Währinger Gürtel 18–20 1090 Vienna Austria; ^4^ Ludwig Boltzmann Institute Applied Diagnostics Währinger Gürtel 18–20 1090 Vienna Austria; ^5^ Institute of Inorganic Chemistry University of Vienna Währinger Straße 42 1090 Vienna Austria; ^6^ Department of Laboratory Medicine Medical University of Vienna Währinger Gürtel 18–20 1090 Vienna Austria

**Keywords:** deoxyfluorinated carbohydrates, d-Ribulose, d-Xylulose, pentose-phosphate-pathway, rare sugars

## Abstract

Fluorinated carbohydrates are valuable tools for enzymological studies due to their increased metabolic stability compared to their non‐fluorinated analogues. Replacing different hydroxyl groups within the same monosaccharide by fluorine allows to influence a wide range of sugar–receptor interactions and enzymatic transformations. In the past, this principle was frequently used to study the metabolism of highly abundant carbohydrates, while the metabolic fate of rare sugars is still poorly studied. Rare sugars, however, are key intermediates of many metabolic routes, such as the pentose phosphate pathway (PPP). Here we present the design and purely chemical synthesis of a set of three deoxyfluorinated analogues of the rare sugars d‐xylulose and d‐ribulose: 1‐deoxy‐1‐fluoro‐d‐ribulose (**1DFRu**), 3‐deoxy‐3‐fluoro‐d‐ribulose (**3DFRu**) and 3‐deoxy‐3‐fluoro‐d‐xylulose (**3DFXu**). Together with a designed set of potential late‐stage radio‐fluorination precursors, they have the potential to become useful tools for studies on the complex equilibria of the non‐oxidative PPP.

## Graphical abstract

Deoxyfluorinated analogues of rare sugars are valuable tools in mechanistic studies on the enzymes involved in their metabolism. Here we describe the first chemical synthesis of 1‐deoxy‐1‐fluoro‐d‐ribulose, 3‐deoxy‐3‐fluoro‐d‐ribulose and 3‐deoxy‐3‐fluoro‐d‐xylulose as tools to study the non‐oxidative phase of the pentose‐phosphate‐pathway. Additionally, three potential radiolabeling precursors for future fluorine‐18 radio‐fluorinations were synthesized.

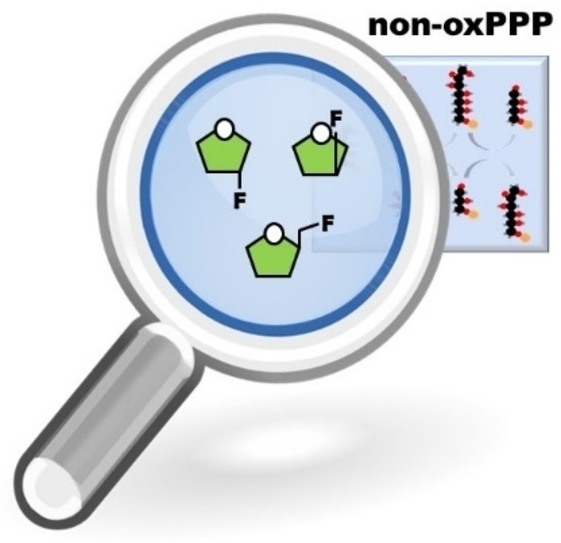

## Introduction

The share of fluorinated pharmaceuticals on all marketed drugs has been constantly rising for over 50 years. Today, fluorinated small molecules make up for 20 % of all marketed pharmaceuticals[^1^, ^2^] and with a total of 300 registered drugs in 2020.^[3]^ This development may be surprising given the low number of fluorinated natural products. However, the strategic positioning of fluorine often beneficially changes the biological properties of a compound, leading to altered selectivity, improved lipophilicity, and improved metabolic stability.^[2]^

Deoxyfluorinated carbohydrates play an outstanding role in this regard. They have been long recognized as valuable tools for mechanistic studies on a wide range of sugar‐processing enzymes on the molecular level.^[4]^ As inhibitors of carbohydrate‐processing enzymes, deoxyfluorinated sugars are potent tools to study carbohydrate transport and enzymology. By functioning as stable analogues to their non‐fluorinated counter‐parts, they contributed to answering many fundamental research questions in the past and were used to study glycosyl transferases, neuramidase, or glycosidase activity.[^2^, ^4^, ^5^] Polyfluorinated sugars find applications in cell uptake‐ and enzyme binding studies, as well as in the development of synthetic vaccines and therapeutic antibodies.^[4]^

Additionally, replacing the stable isotope fluorine‐19 by the positron‐emitting, cyclotron‐derived fluorine‐18, opens up the possibility to study carbohydrate metabolism *in vivo* by positron emission tomography (PET imaging).^[6]^ Today, such radiofluorinated deoxy‐sugars are important diagnostic drugs with applications in oncology, neurology and for the detection of bacterial infections.[^4^, ^5^] However, fueled by the success of [^18^F]2‐fluoro‐2‐deoxy‐d‐glucose ([^18^F]FDG) in PET imaging, most studies focused on the synthesis and possible applications of deoxyfluorinated carbohydrate analogues to highly abundant C_6_‐ and several C_5_ sugars such as glucose, mannose or ribose.[^7^, ^8^, ^9^, ^10^]

The metabolism of rare sugars^[11]^ on the other hand, remains poorly studied despite their high potential as drug building blocks and their high biological importance for a variety of metabolic pathways such as the pentose phosphate pathway (PPP). Rare sugar phosphates of different chain length (C_4_ to C_7_) are key intermediates of the non‐oxidative phase of the PPP (non‐oxPPP), Several of the enzymes of the non‐oxPPP are known to be crucial for cell survival (transketolase, TKT) and a lack of others is associated with a wide spectrum of diseases (e. g. transaldolase, TALDO),^[12]^ but its metabolic contribution is still poorly understood. Deoxyfluorinated analogues to the rare sugar intermediates of the non‐oxPPP, with strategically chosen fluorination sites, have the potential to become useful tools for mechanistic studies on the complex equilibria of this pathway.

## Results and Discussion

### Design of target compounds

**Scheme 1:**
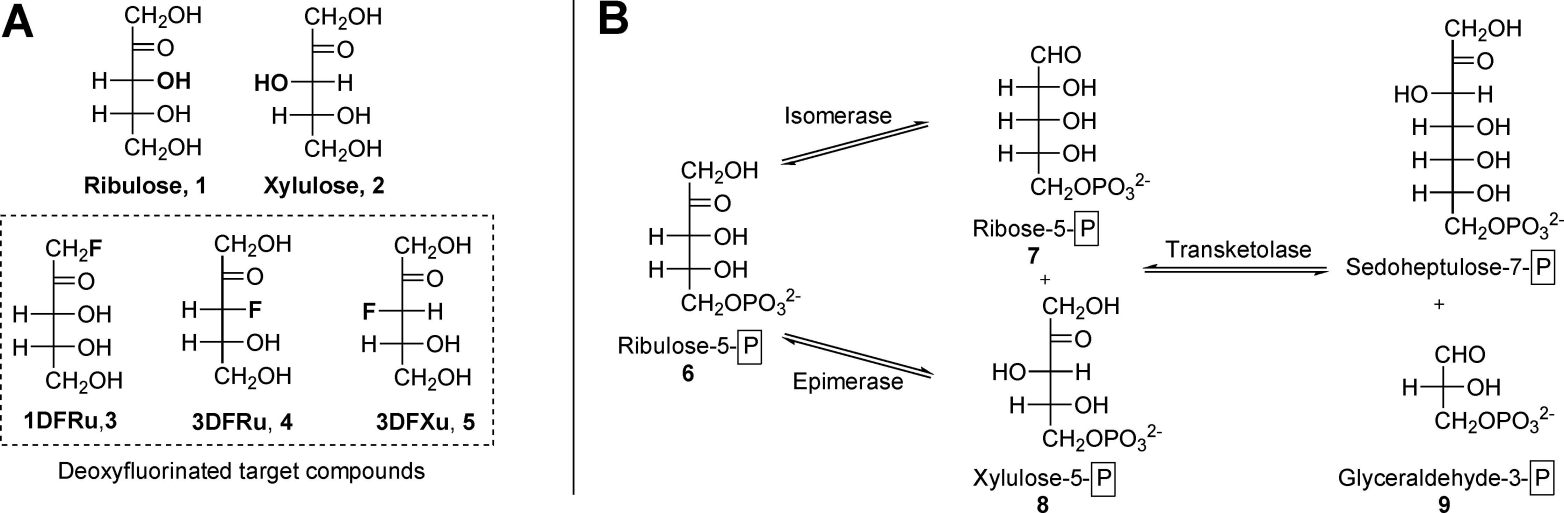
**A**: Structures of designed deoxyfluorinated target compounds **3**–**5** and the respective native sugars **1** and **2**; **B**: Selected enzymatic transformations of the non‐oxidative PPP. Shown are only those equilibria that are assumed to be influenced in the presence of **3**–**5**; P in a square box symbolizes −PO_3_^2−^.

Here we describe the design and synthesis of a set of three deoxyfluorinated analogues to the rare pentuloses d‐ribulose (**1**) and d‐xylulose (**2**): 1‐deoxy‐1‐fluoro‐d‐ribulose (**1DFRu**, **3**), 3‐deoxy‐3‐fluoro‐d‐ribulose (**3DFRu**, **4**) and 3‐deoxy‐3‐fluoro‐d‐xylulose (**3DFXu**, **5**). The biodistribution and metabolism of d‐ribulose (**1**) and d‐xylulose (**2**) and their regulatory role on the PPP have never been studied using deoxyfluorinated analogues of the native sugars. The compounds we suggest are expected to become phosphorylated by their respective kinases, ribulokinase (EC : 2.7.1.15) and xylulokinase (EC : 2.7.1.17), but not be further metabolized by the enzymes of the non‐oxPPP (Scheme 1).

d‐Ribulose 5‐phosphate (**6**) is a substrate for two enzymes of the non‐oxPPP, namely isomerase (transforming **6** to d‐ribose 5‐phosphate (**7**)), and epimerase (inverting the configuration of **6** at C‐3 to form d‐xylulose 5‐phosphate (**8**)). Since the free hydroxyl group at position 1 is crucial for the conversion of d‐ribulose 5‐phosphate (**6**) to d‐ribose 5‐phosphate (**7**) via an ene‐diol intermediate, 1‐deoxy‐1‐fluoro‐d‐ribulose (**1DFRu**, **3**) will, after phosphorylation inside cells, presumably not be a substrate for isomerase.^[13]^ Alternatively, the phosphate of 3‐deoxy‐3‐fluoro‐d‐ribulose (**3DFRu**, **4**) is suggested to hinder the abstraction of an acidic proton by epimerase at C‐3.^[14]^ 3‐Deoxy‐3‐fluoro‐d‐xylulose (3DFXu, **5**) has the potential to act in a similar way on the reverse transformation. d‐Xylulose 5‐phosphate (**8**) is also a substrate of transketolase (TKT), which transfers a C_2_‐unit (composed of the C1–C2 fragment of d‐xylulose‐5‐phosphate (**8**)) to either d‐ribose 5‐phosphate (**7**) or d‐erythrose 4‐phosphate (**9**). As the C‐3 hydroxyl group of **8** is mechanistically needed to cleave the C2–C3 bond, 3‐deoxy‐3‐fluoro‐d‐xylulose (**5**) is additionally assumed to be a potential tool to study TKT.^[15]^

As *in vivo* studies using fluorine‐19 deoxyfluorinated carbohydrates require a comparably high substance amount, studying reversible enzymatic reactions is difficult. By replacing fluorine‐19 with fluorine‐18, the detection limit is significantly lowered. Thus, only picomolar quantities of ^18^F‐deoxyfluorinated carbohydrates are needed and *in vivo* studies can be performed without interfering with the involved reaction equilibria.

Therefore, we designed suitable precursors for the synthesis of the respective ^18^F‐deoxyfluorinated analogues of **1DFRu** (**3**), **3DFRu** (**4**) and **3DFXu** (**5**). Given the short half‐life of fluorine‐18 (110 min) and the need for radiation protection, the synthetic options to effectively radio‐fluorinate carbohydrate‐scaffolds are limited. The designed precursors are specifically tailored to meet these time constraints and could be used for the late‐stage radio‐fluorination of these carbohydrates. All three precursors contain a triflate leaving group (which is commonly used in radiolabeling settings) and protecting groups that can be easily cleaved under mild conditions.

#### Synthesis of 1‐deoxy‐1‐fluoro‐d‐ribulose and its potential radiolabelling precursor

**Scheme 2:**
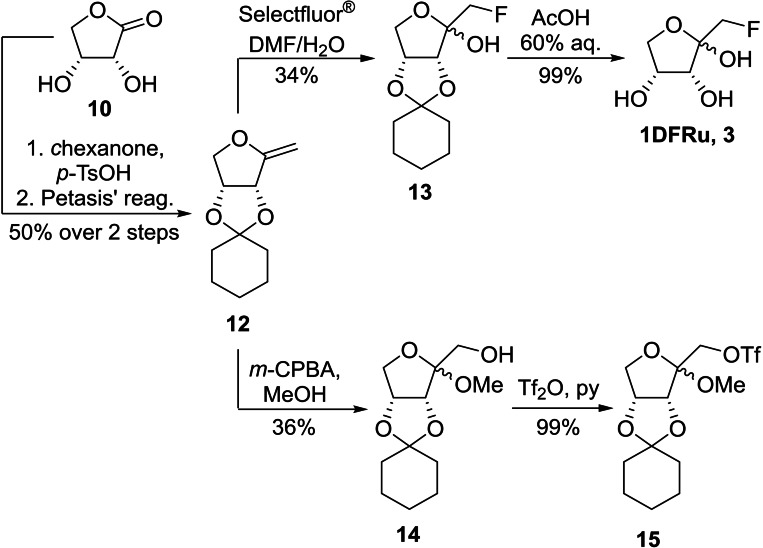
Key steps of the convergent synthesis of 1‐deoxy‐1‐fluoro‐d‐ribulose (**1DFRu**, **3**) and methyl 3,4‐*O*‐cyclohexylidene‐1‐*O*‐trifluoromethanesulfonyl‐d‐*ribulo*‐furanoside (**15**) from intermediate **12**.

For the synthesis of 1‐deoxy‐1‐fluoro‐d‐ribulose we strived for a convergent approach to access both the fluorinated sugar as well as its potential radiolabeling precursor from the same, common intermediate. The synthesis starts with the acetalization of d‐*erythrono*‐1,4‐lactone (**10**) in the presence of cyclohexanone and *para*‐toluenesulfonic acid under Dean‐Stark conditions.^[16]^ The protected lactone was then methylenated using Petasis’ reagent[^17^, ^18^] in toluene at 70 °C yielding compound **12** (Scheme 2).

Here, the cyclohexylidene acetal was crucial since the corresponding isopropylidene acetal proved to be volatile during isolation. With exocyclic enol ether **12** in hand, we turned our attention to the versatility of the electron‐rich double bond at position 1. It allowed us to perform an electrophilic fluorination with Selectfluor^®^ in DMF/H_2_O to obtain fluorinated ribulose **13**.^[19]^ Subsequent deprotection with aqueous acetic acid at 100 °C^[20]^ gave smooth access to 1‐deoxy‐1‐fluoro‐d‐ribulose (**1DFRu**, **3**) as a 2.3 : 1 mixture of anomers. On the other hand, enol ether **12** can also be subjected to an epoxidation‐alcoholysis protocol using *m*‐CPBA in methanol^[21]^ to yield methyl furanoside **14**, albeit in modest yield. Triflation of the primary alcohol under standard conditions yielded triflate **15** in excellent yield. The latter can presumably be used for the nucleophilic substitution of the triflate leaving group with [^18^F]fluoride under standard radiofluorination conditions. This approach gives access to both **1DFRu** (**3**) and its potential radiolabeling precursor in a short and convenient 4‐step synthesis.

#### Synthesis of 3‐deoxy‐3‐fluoro‐d‐ribulose and its potential radiolabelling precursor

**Scheme 3:**
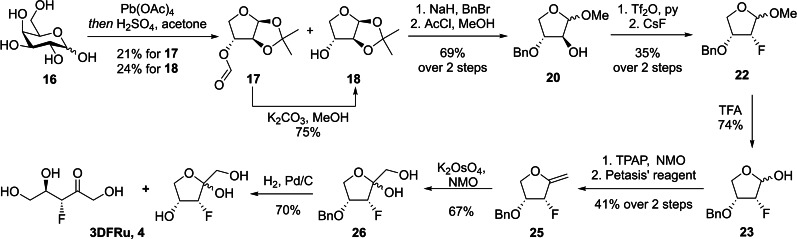
Synthesis of 3‐deoxy‐3‐fluoro‐d‐ribulose (**3DFRu**) from d‐galactose.

The synthesis of 3‐deoxy‐3‐fluoro‐d‐ribulose (**3DFRu**, **4**) proved to be synthetically demanding. The *syn*‐relationship of the hydroxyl‐groups in positions C‐3 and C‐4 rules out the possibility to install a fluorine atom by an epoxide opening approach. Thus, we opted for a classical S_N_2‐reaction with inversion of configuration at C‐3. Cesium fluoride (CsF) proved to be a suitable fluoride donor in combination with a threose scaffold. For this, d‐galactose (**16**) was trimmed down by Criegee oxidation^[22]^ to yield a mixture of 3‐*O*‐formyl‐1,2‐*O*‐isopropylidene‐d‐*threo*‐furanose (**17**) as well as 1,2‐*O*‐isopropylidene‐d‐*threo*‐furanose (**18**)^[23]^ which could be separated during work‐up and isolated **17** smoothly converted to **18** (Scheme 3).

Compound **18** was benzylated at position 3 and after removal of the isopropylidene acetal^[24]^ gave methyl glycoside **20** on a multi‐gram scale.^[25]^ Subsequent triflation and nucleophilic substitution with CsF in *tert*‐amylalcohol at 90 °C^[26]^ yielded 35 % of our desired fluorinated *erythro*‐compound **22**.^[27]^ The methyl furanoside was then hydrolyzed in aqueous trifluoroacetic acid^[28]^ to give erythrose **23**. Ley–Griffith oxidation^[29]^ of **23** to the corresponding lactone **24** again set the stage for a methylenation with Petasis’ reagent to give exocyclic enol ether **25**. The double bond could be easily dihydroxylated with catalytic amounts of potassium osmate in the presence of *N*‐morpholine‐*N*‐oxide (NMO).^[28]^ Final hydrogenolysis yielded 3‐deoxy‐3‐fluoro‐d‐ribulose (**3DFRu**, **4**) as a 2.1 : 2 : 1 mixture of both anomeric furanoses and the corresponding open chain conformation.

**Scheme 4:**
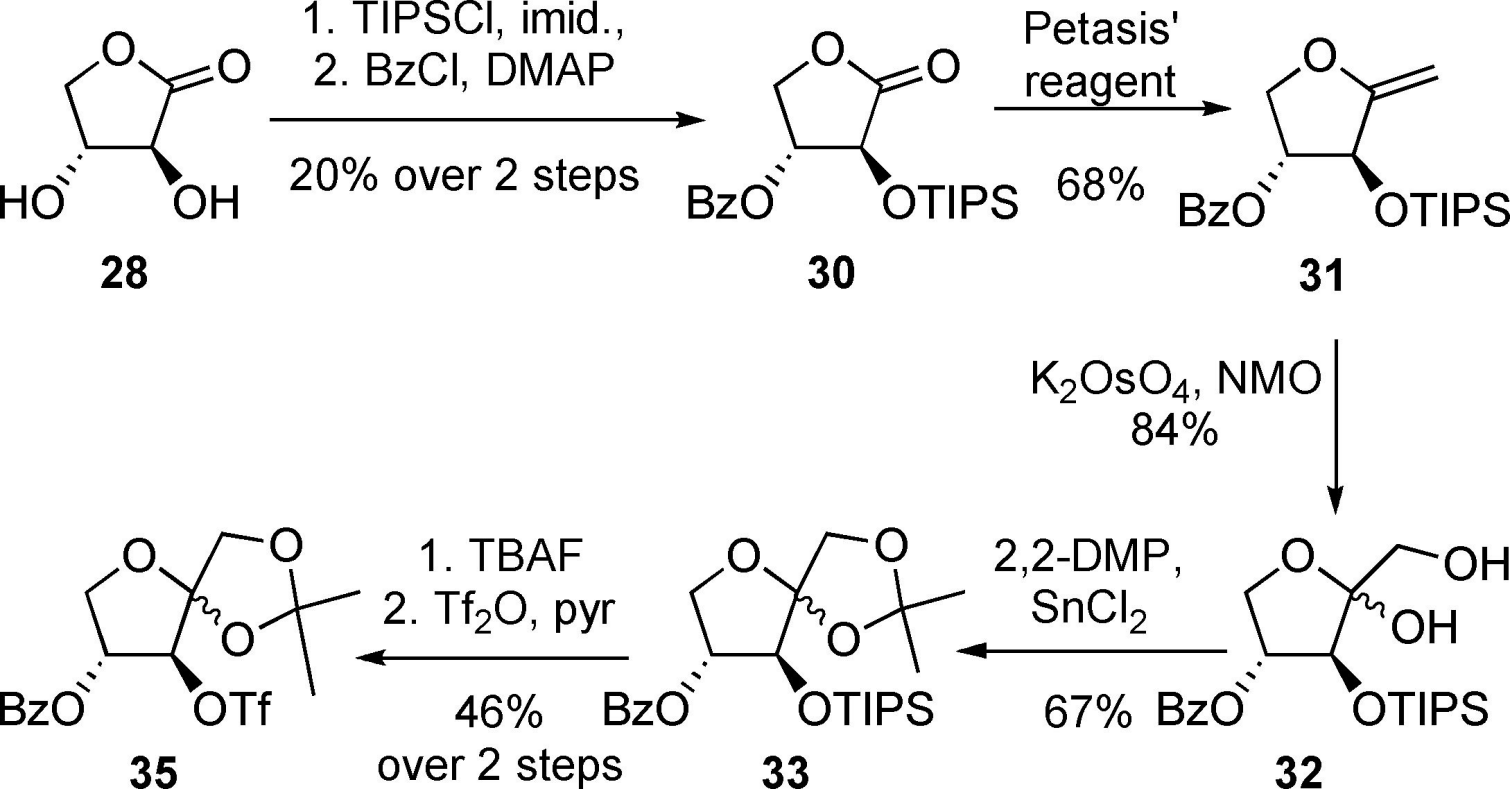
Key steps of the synthesis of 4‐*O*‐benzoyl‐1,2‐*O*‐isopropylidene‐3‐*O*‐trifluoromethanesulfonyl‐d‐xylulose (**35**) from d‐*threono*‐1,4‐lactone (**28**).

We then identified d‐xylose (**27**) as the ideal, cheap, and readily available starting material for the synthesis of a suitable radiolabeling precursor for **3DFRu** (**4**) (Scheme 4).

First, a Spengler–Pfannenstiel oxidation, followed by an acidic esterification gave d‐*threono*‐1,4‐lactone (**28**) in acceptable yield.^[30]^ Selective silyl ether formation at position 2,^[31]^ using triisopropylsilyl chloride (TIPSCl) and imidazole proved troublesome, probably due to the *anti*‐orientation of the hydroxyl groups not providing enough steric hindrance to avoid diprotection. Subsequent benzoylation with benzoyl chloride and 4‐(dimethylamino)pyridine (DMAP) in pyridine yielded **30** in 20 % over 2 steps. With lactone **30** in hand, we followed the above described methylenation and dihydroxylation protocol to get to xylulose **32**. The TIPS ether proved to be crucial for this transformation, since *tert*‐butyldimethylsilyl (TBS) and even *tert*‐butyldiphenylsilyl (TBDPS) groups were prone to migration to the primary hydroxyl group at C‐1 (data not shown). Isopropylidene protection of the newly formed diol **32** under Lewis acidic conditions^[32]^ gave the fully protected pentulose **33**. Desilylation with tetrabutylammonium fluoride (TBAF) at 0 °C^[33]^ paved the way for triflation under standard conditions at C‐3^[34]^ to yield a suitable radiolabelling precursor of **4** in 9 steps from d‐xylose.

#### Synthesis of 3‐deoxy‐3‐fluoro‐d‐xylulose and its potential radiolabelling precursor

**Scheme 5:**
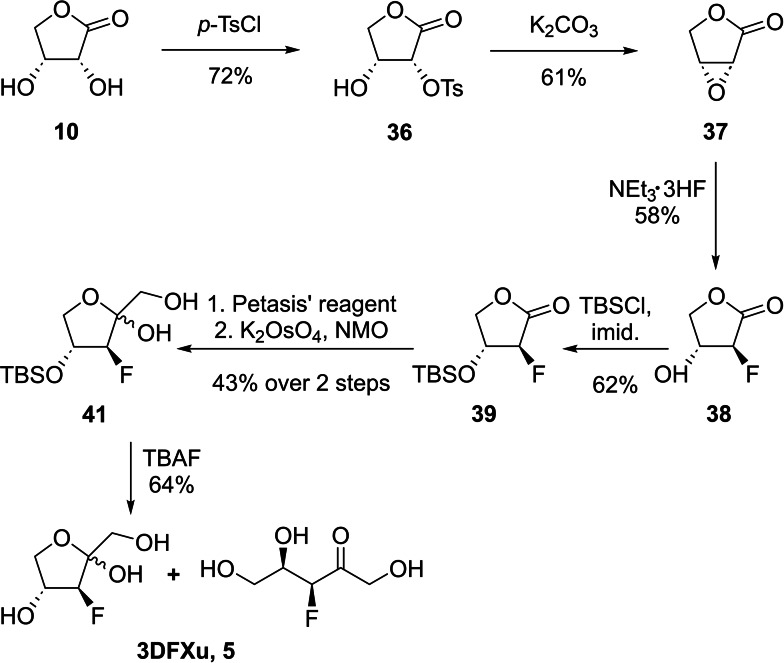
Synthesis of 3‐deoxy‐3‐fluoro‐d‐xylulose (**3DFXu**, **5**) from tosylate **36**.

The readily available d‐*erythrono*‐1,4‐lactone (**10**) was also used as starting material for the synthesis of 3‐deoxy‐3‐fluoro‐d‐xylulose (**3DFXu**, **5**). Since the two hydroxyl groups at positions C‐3 and C‐4 in xylulose are *anti‐*oriented, installation of fluorine by an epoxide opening approach was possible in this case. d‐*Erythrono*‐1,4‐lactone (**10**) was first selectively tosylated at position O‐2. Keeping the reaction temperature below 0 °C showed to be crucial to avoid di‐*O*‐tosylation.^[35]^ The resulting mono‐tosylate **36** then underwent a two‐step nucleophilic substitution process in the presence of potassium carbonate^[36]^ to give the desired 2,3‐anhydro‐d‐*erythro*‐1,4‐lactone (**37**)^[37]^ with a net retention of configuration at C‐2 (Scheme 5).

**Scheme 6:**
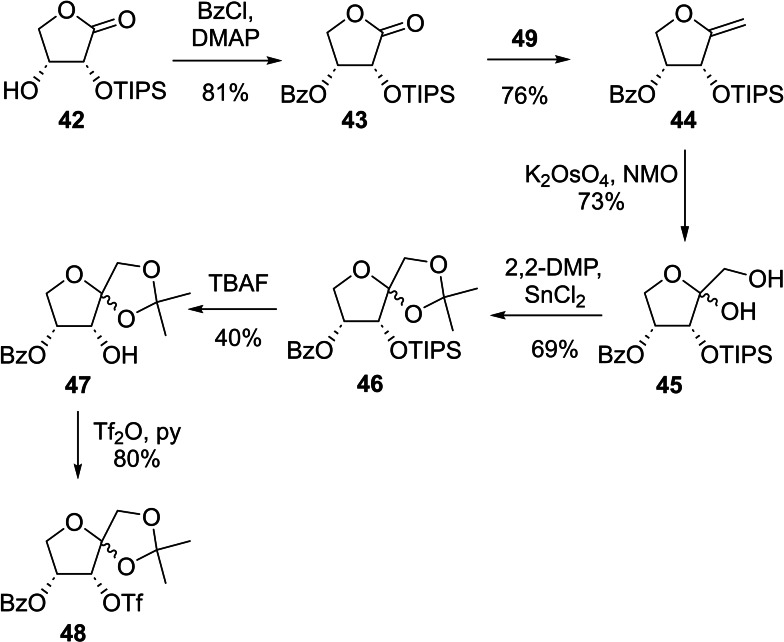
Synthesis of 4‐*O*‐benzoyl‐1,2‐*O*‐isopropylidene‐3‐*O*‐trifluoro‐methanesulfonyl‐d‐ribulose (**48**) from lactone **42**.

Lundt *et al*. described a variety of different fluoride sources for epoxide opening.^[38]^ Of these, triethylamine trihydrofluride, proved to be the ideal candidate in our case as it is a very mild, selective, and readily available fluorinating agent.^[39]^ Subsequently, TBS was chosen for the protection of the C‐3 hydroxyl group of 2‐deoxy‐2‐fluoro‐d‐*threono*‐1,4‐lactone (**38**), due to its stability under Petasis’ methylenation conditions, while simultaneously reducing the volatility of the intermediate enol ether **40**. After dihydroxylation of the double bond,^[28]^ the TBS group was removed in the presence of TBAF to yield 3‐deoxy‐3‐fluoro‐d‐xylulose (**3DFXu**, **5**) as a 4 : 5 : 1 mixture of both anomeric furanoses and the corresponding open chain conformation. Since the envisioned radiolabeling precursor for **3DFXu** (**5**) needs a *syn*‐relationship between the installed leaving group and the hydroxy group at position C‐4, again lactone **10** proved to serve as ideal starting material (Scheme 6). Conveniently, the TIPS‐protection at position O‐2 proceeded with significantly higher yield than the silylation of the *anti*‐oriented lactone **28**. This result provides additional evidence for the assumed relation between the relative orientation of the C‐2 and C‐3 hydroxyl groups and the observed diprotection in lactone **28**. Following a benzoylation, methylenation and dihydroxylation sequence as described above, the resulting diol **45** was isopropylidene protected. This three‐step sequence worked as smoothly as described for the synthesis of the **3DFRu** precursor **35**.

Unfortunately, TIPS deprotection using the above described conditions (1 M TBAF in THF at 0 °C) partly led to migration of the benzoate from position 4 to the ‐ now unprotected ‐ hydroxyl group at C‐3, presumably caused by the *syn*‐orientation of both groups.^[40]^ After triflation of the ribulose derivative **47** at position O‐3 with trifluoromethanesulfonic anhydride (Tf_2_O) and pyridine at 0 °C, the proposed radiolabeling precursor 4‐*O*‐benzoyl‐1,2‐*O*‐isopropylidene‐3‐*O*‐trifluoromethanesulfonyl‐d‐ribulose (**48**) was obtained as a 5 : 1 mixture of two anomers.

## Conclusion

In summary, we have designed and synthesized a set of three deoxyfluorinated analogues to the rare pentuloses of the pentose phosphate pathway. We succeeded to develop the first chemical synthesis of 1‐deoxy‐1‐fluoro‐d‐ribulose (**3**), 3‐deoxy‐3‐fluoro‐d‐ribulose (**4**) and 3‐deoxy‐3‐fluoro‐d‐xylulose (**5**) from cheap and readily available starting materials. Additionally, we devised routes to access three potential radiolabeling precursors for these sugars. Compounds **15**, **35** and **48** all contain triflate leaving groups in appropriate positions for late‐stage radio‐fluorination attempts as well as protecting groups that can be rapidly cleaved under mild conditions. In future studies, we will investigate their applicability to access the respective fluorine‐18 labeled pentuloses.

## Conflict of interest

The authors declare no conflict of interest.

1

## Supporting information

As a service to our authors and readers, this journal provides supporting information supplied by the authors. Such materials are peer reviewed and may be re‐organized for online delivery, but are not copy‐edited or typeset. Technical support issues arising from supporting information (other than missing files) should be addressed to the authors.

Supporting Information

## Data Availability

Pictures of full NMR spectra are available in the supporting information. Raw data files are available from the authors upon request.
