# Supplementary material for: Fluorinated Analogues to the Pentuloses of the Pentose Phosphate Pathway
Source: European J Org Chem. 2023 Jun 2;26(31):e202300339. doi: 10.1002/ejoc.202300339 (PMC10946780; doi:10.1002/ejoc.202300339)

European Journal of Organic Chemistry

Supporting Information

Fluorinated Analogues to the Pentuloses of the Pentose Phosphate Pathway

Lukas Scheibelberger[+], Toda Stankovic[+], Kaja Liepert, Andreas Kienzle, Eva-Maria Patronas, Theresa Balber, Markus Mitterhauser, Arvand Haschemi, and Katharina Pallitsch*

Synthetic Procedures

General Experimental Details

All used chemicals and solvents were purchased from commercial sources and used without further purification. ^{1}H, ^{13}C and ^{19}F NMR spectra were recorded on either a Bruker AV III HD 700 (^{1}H: 700.40 MHz, ^{13}C: 176.12 MHz, ^{19}F: 659.03 MHz), AV III 600 (^{1}H: 600.25 MHz, ^{13}C: 150.93 MHz, ^{19}F: 564.803 MHz) or AV NEO 500 (^{1}H: 500.32 MHz, ^{13}C: 125.81 MHz) spectrometer. Chemical shifts (δ) are given in parts per million (ppm) and were referenced to (residual) solvent signals as follows: ^{1}H NMR spectra: $CDCl_3$: δ_H ($CHCl_3$) 7.26, *d*$_6$-DMSO: δ_H [(CD_2H)SO(CD_3)] 2.50, *d*$_4$-MeOH: δ_H (CD_3OH) 3.31, and D_2O: δ_H (HDO) 4.79; ^{13}C NMR spectra:$CDCl_3$ (δ_C 77.16), *d*$_6$-DMSO (δ_C 39.52), and *d*$_4$-MeOH (δ_C 49.00). ^{13}C NMR spectra in D_2O were referenced indirectly to the ^{1}H NMR frequency of the sample using the ”xiref”-command in Bruker Topspin. External CCl_3F (δ_F 0.00) served as reference for ^{19}F NMR spectra. Coupling constants (*J*) are reported in Hz. ^{13}C spectra were recorded *j*-modulated. The chemical shift of the two parts of AB-systems are given separately as unweighted mean value of the single signals. “A” is used to denote the down-field part and “B” to denote the highfield part of the AB-system. High resolution mass spectrometry (HRMS) was conducted on a Bruker maXis UHR-TOF instrument with electrospray ionization (ESI) in the positive ion mode. Optical rotations were measured on a Schmidt-Haensch Digital Polarimeter Unipol L 2000 and are given in 10^{-1} deg cm^2 g^{-1}. Chromatographic separations (MPLC) were carried out on a Biotage Isolera Prime (Biotage, Uppsala, Sweden) flash purification system using Macherey-Nagel silica gel 60 (0.04–0.063 mm) in self-packed cartridges. Thin layer chromatography (TLC) was carried out on precoated Merck silica gel 60 F_{254} glass plates or precoated Macherey-Nagel ALUGRAM Xtra SIL G UV254 aluminum plates. Compounds were visualized with UV light (254 nm) and/or by dipping the plate in one of the following solutions, followed by heating with a heat gun: Cerium ammonium molybdate solution (CAM, 46 g $(NH_4)_6Mo_7O_{24}$×4 H_2O, 2 g $Ce(SO_4)_2$×4H_2O) in 1 L 10%(w/w) aq. H_2SO_4) or $KMnO_4$ solution (9 g $KMnO_4$, 60 g K_2CO_3 in 900 mL H_2O and 15 mL 5%(w/w) aq. NaOH).

Bis(cyclopentadienyl)dimethyltitanium (Petasis’ reagent, 49).[17] This reaction is light sensitive and was carried out in the dark. Bis(cyclopentadienyl)titanium dichloride (3.5 g, 14.1 mmol, 1 equiv.) was suspended in dry Et_2O (70.3 mL) in a flame-dried flask under argon atmosphere and cooled to 10°C. Methyl lithium (1.6 M in Et_2O, 21.1 mL, 33.7 mmol, 2.4 equiv.) was added dropwise and the reaction mixture was stirred for 15 min at room temperature. The reaction was quenched by the dropwise addition of ice-cold H_2O (30 mL) at 0°C. The layers were separated, and the aqueous layer was extracted with Et_2O (2 × 20 mL). The combined organic layers were dried ($MgSO_4$), filtered, and evaporated at 25°C to give bis(cyclopentadienyl)dimethyltitanium (**49**) as an orange solid (2.9 g, 99%). The product was stored as a 0.5 M solution in dry toluene under argon at −20°C: ^{1}H NMR (600 MHz,

$CDCl_3$) δ = 6.06 (s, 10H, CH^{Cp}), −0.15 (s, 6H, CH_3); ^{13}C NMR (151 MHz, $CDCl_3$) δ = 113.23 (10 CH^{Cp}), 45.61 (2 CH_3).

Synthesis of 1-deoxy-1-fluoro-D-ribulose (1DFRu, 3) and its potential radiolabelling precursor 15

D-*Erythrono*-1,4-lactone (10).[41] D-Isoascorbic acid (10 g, 56.8 mmol, 1 equiv.) was dissolved in water (142 mL) in a three-necked flask equipped with a thermometer and an addition funnel. The solution was cooled to 0°C and solid Na_2CO_3 (12.035 g, 113.6 mmol, 2 equiv.) was added in small portions. H_2O_2 [30 % (w/w), 13.1 mL, 127.8 mmol, 2.25 equiv.] was added dropwise over 10 min at 0°C (internal temperature rose to 19°C) and the reaction mixture was stirred at 0°C for another 5 min, followed by 30 min at 42°C. Activated charcoal (2.27 g) was added and the reaction mixture was heated to 75°C until the gas formation ceased, followed by hot filtration over a pad of Celite®, moistened with water (30 mL). The filtrate was carefully acidified to pH 1 with aq. HCl (6 M, 42.5 mL) followed by evaporation of the solvent. The colorless residue was dried under high vacuum, suspended in refluxing EtOAc (92 mL) and the supernatant was decanted. The process was repeated once with an additional portion of EtOAc (34 mL). The combined organic solutions were left to stand at 4°C overnight. The precipitate was collected by filtration, washed with ice-cold EtOAc (1 × 30 mL) and dried under high vacuum to give D-*erythrono*-1,4-lactone (**10**) as colorless needles (4.45 g, 66%): R_f = 0.19 (EtOAc, $KMnO_4$); $[\alpha]_D^{20}$ = −74.1 (*c* 1.0, H_2O), {lit.[41]: $[\alpha]_D^{25}$ = −72.8 (*c* 0.498, H_2O)}; ^1H NMR (600 MHz, d_6-DMSO): δ = 5.76 (broad s, 1H, OH), 5.35 (broad s, 1H, OH), 4.37 (d, $^3J_{2,3}$ = 4.3 Hz, 1H, H-2), 4.28 (dd, $^2J_{4a,4b}$ = 9.9 Hz, $^3J_{4a,3}$ = 3.1 Hz, 1H, H-4a), 4.23 (dd, $^3J_{3,2}$ = 4.3 Hz, $^3J_{3,4a}$ = 3.1 Hz, 1H, H-3), 4.04 (d, $^2J_{4b,4a}$ = 9.9 Hz, 1H, H-4b); ^{13}C-NMR (151 MHz, d_6-DMSO): δ = 176.41 (C-1), 71.78 (C-4), 69.45 (C-2), 68.38 (C-3); HRMS (ESI+): m/z calc. for $C_4H_6O_4Na^+$ $[M+Na]^+$: 141.0158, found: 141.0157.

2,3-*O*-Cyclohexylidene-D-*erythrono*-1,4-lactone (11). Cyclohex-anone (3.24 g, 3.42 mL, 33.0 mmol, 1.3 equiv.) and *para*-toluenesulfonic acid monohydrate (242 mg, 1.3 mmol, 0.05 equiv.) were added to D-*erythrono*-1,4-lactone (**10**, 3 g, 25.4 mmol, 1 equiv.) suspended in dry toluene (150 mL), and the resulting mixture was refluxed with a Dean-Stark apparatus for 16 h. After cooling to room temperature, the mixture was poured into a sat. aq. $NaHCO_3$-solution (200 mL) and extracted with Et_2O (3 × 100 mL). The combined organic layers were washed with brine, dried ($MgSO_4$), filtered and evaporated. The crude residue was crystallized (cyclohexane) to give 2,3-*O*-cyclohexylidene-D-*erythrono*-1,4-lactone (**11**) as a colorless solid (4.057 g, 81%): R_f = 0.26 (*n*hept/EtOAc 2:1, $KMnO_4$); $[\alpha]_D^{20}$ = −113.7 (*c* 1.0, $CHCl_3$), {lit. [16]] $[\alpha]_D^{21}$ = −106.6 (*c* 1.03, $CHCl_3$)}; ^1H NMR (600 MHz, $CDCl_3$) δ = 4.86 (dd, $^3J_{3,2}$ = 5.5 Hz, $^3J_{3,4b}$ = 3.9 Hz, 1H, H-3), 4.74 (d, $^3J_{2,3}$ = 5.5 Hz, 1H, H-2), 4.48 (d, $^2J_{4a,4b}$ = 11.0 Hz, 1H, H-4a), 4.40 (dd, $^2J_{4b,4a}$ = 11.0 Hz, $^3J_{4b,3}$ = 3.9 Hz, 1H, H-4b), 1.71-1.56 (m, 8H, CH_2^{chex}), 1.44-1.37 (m, 2H, CH_2^{chex}); ^{13}C NMR (151 MHz, $CDCl_3$) δ = 174.32 (C-1), 114.98 (C^{chex}), 75.17 (C-3), 74.46 (C-2), 70.51 (C-4), 36.54 (CH_2^{chex}), 35.28 (CH_2^{chex}), 24.90 (CH_2^{chex}), 23.97 (CH_2^{chex}), 23.86 (CH_2^{chex}.); HRMS (ESI+): m/z calc. for $C_{10}H_{14}O_4Na^+$ $[M+Na]^+$: 221.0784, found: 221.0788.

2,5-Anhydro-3,4-*O*-cyclohexylidene-1-deoxy-D-*erythrono*-pent-1-enitol (12). Petasis' reagent (**49**, 0.5 M in toluene, 15.1 mL, 7.6 mmol, 1.5 equiv.) was slowly added to 2,3-*O*-Cyclohexylidene-D-*erythrono*-1,4-lactone (**11**, 1 g, 5.0 mmol, 1 equiv.) in dry toluene (25 mL) under argon atmosphere. The flask was equipped with a reflux condenser and the mixture was heated to 70°C for 16 h. After cooling to room temperature, *n*-heptane (80 mL) was added and stirring was continued for 30 min. The orange suspension was filtered over Celite® and the filtrate was evaporated at 25°C. The crude residue was purified by MPLC (100 g silica gel, 2-18% Et_2O in *n*-pentane) to give 2,5-anhydro-3,4-*O*-cyclohexylidene-1-deoxy-D-*erythrono*-pent-1-enitol (**12**) as a yellow oil (70 mg, 57%): R_f = 0.25 (*n*hept/EtOAc 9:1, CAM); $[\alpha]_D^{20}$ = −231.3 (*c* 1.0, $CHCl_3$); ^{1}H NMR (600 MHz, $CDCl_3$) δ = 5.00 (d, $^3J_{3,4}$ = 5.5 Hz, 1H, H-3), 4.78 (t, $^3J_{4,3}$ = $^3J_{4,5b}$ = 5.5 Hz, 1H, H-4), 4.47 (s, 1H, H-1a), 4.27 (s, 1H, H-1b), 4.24 (d, $^2J_{5a,5b}$ = 10.4 Hz, 1H, H-5a), 4.08 (dd, $^2J_{5b,5a}$ = 10.4 Hz, $^3J_{5b,4}$ = 5.5 Hz, 1H, H-5b), 1.75-1.69 (m, 2H, CH_2^{chex}), 1.67-1.55 (m, 6H, CH_2^{chex}), 1.44-1.35 (m, 2H, CH_2^{chex}); ^{13}C NMR (151 MHz, $CDCl_3$) δ = 162.46 (C-2), 114.10 (C^{chex}), 85.82 (C-1), 79.22 (C-3), 78.29 (C-4), 74.51 (C-5), 36.84 (CH_2^{chex}), 35.48 (CH_2^{chex}), 25.15 (CH_2^{chex}), 24.12 (CH_2^{chex}), 24.01 (CH_2^{chex}); HRMS (ESI+): m/z calc. for $C_{11}H_{17}O_3^+$ [M+H]$^+$: 197.1172, found: 197.1170.

3,4-*O*-Cyclohexylidene-1-deoxy-1-fluoro-D-ribulose (13). 2,5-Anhydro-3,4-*O*-cyclohexylidene-1-deoxy-D-*erythrono*-pent-1-enitol (**12**, 200 mg, 1.0 mmol, 1 equiv.) was dissolved in a 1:1 mixture of DMF and water (4 mL) and cooled to 0°C. Selectfluor (3.61 g, 10.2 mmol, 10 equiv.) was added and the mixture was stirred at room temperature for 1 h. The mixture was diluted with EtOAc (5 mL) and washed with water (2 × 5 mL) and brine (5 mL), dried ($MgSO_4$), filtered, and evaporated. The crude residue was purified by MPLC (10 g silica gel, 8-60% EtOAc in *n*-heptane) yielding 3,4-*O*-cyclohexylidene-1-deoxy-1-fluoro-D-ribulose (**13**) as a mixture of anomers as a colourless oil (81 mg, 34%): R_f = 0.19 (*n*hept/EtOAc 2:1, CAM); $[\alpha]_D^{20}$ = −60.2 (*c* 1.0, $CHCl_3$); <u>major anomer</u>: ^{1}H NMR (700 MHz, $CDCl_3$) δ = 4.91-4.88 (m, 1H, H-4), 4.64 (dd, $^2J_{1a,F}$ = 46.4 Hz, $^2J_{1a,1b}$ = 9.8 Hz, 1H, H-1a), 4.57 (d, $^3J_{3,4}$ = 5.7 Hz, 1H, H-3), 4.53 (dd, $^2J_{1b,F}$ = 48.0 Hz, $^2J_{1b,1a}$ = 9.8 Hz, 1H, H-1b), 4.07 (dd, $^2J_{5a,5b}$ = 10.3 Hz, $^3J_{5a,4}$ = 3.3 Hz, 1H, H-5a), 4.05 (d, $^2J_{5b,5a}$ = 10.3 Hz, 1H, H-5b), 2.68 (d, $^4J_{OH,F}$ = 2.7 Hz, 1H, OH), 1.70-1.64 (m, 2H, CH_2^{chex}), 1.63-1.58 (m, 2H, CH_2^{chex}), 1.57-1.51 (m, 4H, CH_2^{chex}), 1.45-1.35 (m, 2H, CH_2^{chex}); ^{13}C NMR (176 MHz, $CDCl_3$) δ = 113.87 (C^{chex}), 104.26 (d, $^2J_{2,F}$ = 18.1 Hz, C-2), 84.47 (d, $^3J_{3,F}$ = 1.4 Hz, C-3), 84.36 (d, $^1J_{1,F}$ = 170.7 Hz, C-1), 80.33 (C-4), 72.21 (C-5), 36.08 (CH_2^{chex}), 34.47 (CH_2^{chex}), 25.17 (CH_2^{chex}), 24.10 (CH_2^{chex}), 23.85 (CH_2^{cy}); ^{19}F NMR (659 MHz, $CDCl_3$) δ = −232.25 (t, $^2J_{F,1a}$ = $^2J_{F,1b}$ = 47.5 Hz); <u>minor anomer</u>: ^{1}H NMR (700 MHz, $CDCl_3$) δ = 4.88 (ddd, $^3J_{4,3}$ = 6.4 Hz, $^3J_{4,5b}$ = 4.5 Hz, $^3J_{4,5a}$ = 1.7 Hz, 1H, H-4), 4.65 (dd, $^3J_{3,4}$ = 6.4 Hz, $^4J_{3,F}$ = 1.2 Hz, 1H, H-3), 4.44 (dd, $^2J_{1a,F}$ = 47.0 Hz, $^2J_{1a,1b}$ = 9.6 Hz, 1H, H-1a), 4.40 (dd, $^2J_{1b,F}$ = 47.0 Hz, $^2J_{1b,1a}$ = 9.6 Hz, 1H, H-1b), 4.21 (d, $^4J_{OH,F}$ = 1.6 Hz, 1H, OH), 4.05 (dt, $^2J_{5a,5b}$ = 10.7 Hz, $^3J_{5a,4}$ = $^5J_{5a,F}$ = 1.7 Hz, 1H, H-5a), 3.91 (ddd, $^2J_{5b,5a}$ = 10.7 Hz, $^3J_{5b,4}$ = 4.5 Hz, $^5J_{5b,F}$ = 0.9 Hz, 1H, H-5b), 1.82-1.78 (m, 2H, CH_2^{chex}), 1.71-1.64 (m, 2H, CH_2^{chex}), 1.64-1.59 (m, 2H, CH_2^{chex}), 1.57-1.50 (m, 2H, CH_2^{chex}), 1.45-1.33 (m, 2H, CH_2^{chex}). ^{13}C NMR (176 MHz, $CDCl_3$) δ = 115.11 (C^{chex}), 101.64 (C-2), 84.62 (d, $^1J_{1,F}$ = 172.5 Hz, C-1), 80.02 (C-4), 79.17 (d, $^3J_{3,F}$ = 2.0 Hz, C-3), 70.50 (d, $^4J_{5,F}$ = 2.1 Hz, C-5), 36.06 (CH_2^{chex}), 34.32 (CH_2^{chex}), 25.09 (CH_2^{chex}), 24.12 (CH_2^{chex}), 23.72 (CH_2^{chex}); ^{19}F NMR (659

MHz, $CDCl_3$) δ = −239.58 (t, $^2J_{F,1a}$ = $^2J_{F,1b}$ = 47.0 Hz). HRMS (ESI+): m/z calc. for $C_{11}H_{17}FO_4Na^+$ [M+Na]$^+$: 255.1003, found: 255.1006.

1-Deoxy-1-fluoro-D-ribulose (1DFRu, 3). 3,4-*O*-Cyclohexylidene-1-deoxy-1-fluoro-D-ribulose (**13**, 73 mg, 0.3 mmol, 1 equiv.) was dissolved in aq. acetic acid (60% (v/v), 3 mL) and the mixture was heated for 2 h at 100°C. The mixture was cooled to room temperature and co-evaporated with toluene (3 × 2 mL). The crude residue was purified by reversed-phase chromatography (C_{18}-silica gel, H_2O) yielding 1-deoxy-1-fluoro-D-ribulose (**1DFRu**, **3**) as a 2.3 : 1 mixture of anomers as a colorless solid (44 mg, 92%): R_f = 0.27 (EtOAc, CAM); $[\alpha]_D^{20}$ = −14.9 (*c* 1.0, H_2O); major anomer: ^1H NMR (700 MHz, D_2O) δ = 4.43 (dd, $^2J_{1a,F}$ = 46.6 Hz, $^2J_{1a,1b}$ = 10.0 Hz, 1H, H-1a), 4.39 (dd, $^2J_{1b,F}$ = 46.6 Hz, $^2J_{1b,1a}$ = 10.0 Hz, 1H, H-1b), 4.37 (td, $^3J_{4,3}$ = $^3J_{4,5a}$ = 4.9 Hz, $^3J_{4,5b}$ = 2.5 Hz, 1H, H-4), 4.18 (d, $^3J_{3,4}$ = 4.9 Hz, 1H, H-3), 4.08 (dd, $^2J_{5a,5b}$ = 10.1 Hz, $^3J_{5a,4}$ = 4.9 Hz, 1H, H-5a), 3.99 (dd, $^2J_{5b,5a}$ = 10.1 Hz, $^3J_{5b,4}$ = 2.5 Hz, 1H, H-5b); ^{13}C NMR (176 MHz, D_2O) δ 103.41 (d, $^2J_{2,F}$ = 19.7 Hz, C-2), 85.38 (d, $^1J_{1,F}$ = 172.5 Hz, C-1), 74.43 (C-5), 73.47 (d, $^3J_{3,F}$ = 1.7 Hz, C-3), 72.64 (C-4); ^{19}F NMR (659 MHz, D_2O) δ = −228.06 (t, $^2J_{F,1a}$ = $^2J_{F,1b}$ = 46.6 Hz); minor anomer: ^1H NMR (700 MHz, D_2O) δ = 4.63 (dd, $^2J_{1a,F}$ = 46.7 Hz, $^2J_{1a,1b}$ = 10.1 Hz, 1H, H-1a), 4.59 (q, $^3J_{4,3}$ = $^3J_{4,5a}$ = $^3J_{4,5b}$ = 5.3 Hz, 1H, H-4), 4.47 (dd, $^2J_{1b,F}$ = 46.7 Hz, $^2J_{1b,1a}$ = 10.1 Hz, 1H, H-1b), 4.23 (ddd, $^2J_{5a,5b}$ = 9.5 Hz, $^3J_{5a,4}$ = 5.3, $^5J_{5a,F}$ = 1.0 Hz, 1H, H-5a), 4.16 (d, $^3J_{3,4}$ = 5.0 Hz, 1H, H-3), 3.82 (dd, $^2J_{5b,5a}$ = 9.5 Hz, $^3J_{5b,4}$ = 5.3 Hz, 1H, H-5b); ^{13}C NMR (176 MHz, D_2O) δ = 106.58 (d, $^2J_{2,F}$ = 19.4 Hz, C-2), 85.64 (d, $^1J_{1,F}$ = 169 Hz, C-1), 78.66 (C-3), 73.61 (C-5), 73.06 (C-4); ^{19}F NMR (659 MHz, D_2O) δ = −231.46 (t, $^2J_{F,1a}$ = $^2J_{F,1b}$ = 46.7 Hz); HRMS (ESI+): m/z calc. for $C_5H_9FO_4Na^+$ [M+Na]$^+$: 175.0377, found: 175.0378.

Methyl 3,4-*O*-cyclohexylidene-D-rib-2-ulofuranoside (14). Purification of *m*-CPBA: An aq. NaOH-solution (0.1 M, 41 mL) was mixed with an aq. KH_2PO_4-solution (0.2 M, 25 mL) and filled up to 100 mL with water. The solution was stirred for 5 min and, if necessary, the pH was adjusted to pH 7.5. Commercially available *m*-CPBA (3.0 g, 70−75% purity) was dissolved in Et_2O (20 mL) and washed with the prepared buffer solution (3 × 15 mL, pH 7.5). The organic layer was dried ($MgSO_4$), filtered and evaporated to yield pure *m*-CPBA (1.83 g) as a colorless solid which was stored at +4°C.

2,5-Anhydro-3,4-*O*-cyclohexylidene-1-deoxy-D-*erythrono*-pent-1-enitol (**12**, 184 mg, 0.9 mmol, 1 equiv.) was dissolved in dry MeOH (3.75 mL) and cooled to 0°C. *m*-CPBA (243 mg, 1.4 mmol, 1.5 equiv.) was added and stirring was continued for 1 h at 0°C. The mixture was diluted with CH_2Cl_2 (5 mL) and washed with an aq. solution of NaOH (1 M, 2 × 4 mL) and brine (5 mL). The organic layer was dried ($MgSO_4$), filtered and concentrated. The crude residue was purified by MPLC (10 g silica gel, 12-100% EtOAc in *n*-heptane) yielding methyl 3,4-*O*-cyclohexylidene-D-rib-2-ulofuranoside (**14**) as a colorless oil (82 mg, 36%): R_f = 0.17 (*n*hept/EtOAc 2:1, CAM); $[\alpha]_D^{20}$ = −99.2 (*c* 1.0, $CHCl_3$); ^1H NMR (700 MHz, $CDCl_3$) δ = 4.84 (dd, $^3J_{4,3}$ = 5.9 Hz, $^3J_{4,5b}$ = 3.9 Hz, 1H, H-4), 4.50 (d, $^3J_{3,4}$ = 5.9 Hz, 1H, H-3), 4.01 (d, $^2J_{5a,5b}$ = 10.3 Hz, 1H, H-5a), 3.87 (dd, $^2J_{1a,1b}$ = 12.1 Hz, $^3J_{1a,OH}$ = 2.6 Hz, 1H, H-1a), 3.81 (dd, $^2J_{5b,5a}$ = 10.3 Hz, $^3J_{5b,4}$ = 3.9 Hz, 1H, H-5b), 3.79 (d, $^2J_{1b,1a}$ = 12.1 Hz, 1H, H-1b), 3.24 (s, 3H, OCH_3), 2.09 (broad s, 1H, OH), 1.74-1.67 (m, 2H, CH_2^{chex}), 1.66-1.60 (m, 2H, CH_2^{chex}), 1.60-1.50 (m,

4H, CH_2^{chex}), 1.45-1.39 (m, 1H, CH_2^{chex}), 1.39-1.33 (m, 1H, CH_2^{cy}); ^{13}C NMR (176 MHz, $CDCl_3$) δ 113.68 (C^{chex}), 108.82 (C-2), 84.65 (C-3), 80.12 (C-4), 71.83 (C-5), 59.03 (C-1), 48.60 (OCH_3), 36.09 (CH_2^{chex}), 34.35 (CH_2^{chex}), 25.20 (CH_2^{chex}), 24.17 (CH_2^{chex}), 23.85 (CH_2^{chex}); HRMS (ESI+): m/z calc. for $C_{12}H_{20}O_5Na^+$ [M+Na]$^+$: 267.1203, found: 267.1213.

Methyl 3,4-*O*-cyclohexylidene-1-*O*-trifluoromethanesulfonyl-D-rib-2-ulofuranoside (15). Dry pyridine (99 mg, 100 µl, 1.25 mmol, 8.5 equiv.) and trifluoromethanesulfonic anhydride (83 mg, 49 µl, 0.3 mmol, 2 equiv.) were added dropwise to a solution of methyl 3,4-*O*-cyclohexylidene-D-rib-2-ulofuranoside (**14**, 36 mg, 0.15 mmol, 1 equiv.) in dry CH_2Cl_2 (740 µL) under argon atmosphere at 0°C. The reaction mixture was stirred at 0°C for 1 h before addition of MeOH (100 µL) and was partitioned between CH_2Cl_2 (2 mL) and water (2 mL). The layers were separated, and the aqueous layer was extracted with CH_2Cl_2 (2 × 5 mL). The combined organic layers were washed with ice-cold water (2 × 5 mL), ice-cold sat. aq. $NaHCO_3$-solution (5 mL) and ice-cold brine (5 mL). The organic layer was dried ($MgSO_4$), filtered and concentrated. The crude residue was dried under high vacuum to give methyl 3,4-*O*-cyclohexylidene-1-*O*-trifluoromethanesulfonyl-D-rib-2-ulofuranoside (**15**) as a yellow oil (55 mg, 99%): R_f = 0.53 (*n*hept/EtOAc 2:1, CAM); $[\alpha]_D^{20}$ = −71.5 (*c* 1.0, $CHCl_3$); ^{1}H NMR (600 MHz, $CDCl_3$) δ = 4.86 (dd, $^3J_{4,3}$ = 5.7 Hz, $^3J_{4,5b}$ = 4.0 Hz, 1H, H-4), 4.65 (d, $^2J_{1a,1b}$ = 10.4 Hz, 1H, H-1a), 4.59 (d, $^2J_{1b,1a}$ = 10.4 Hz, 1H, H-1b), 4.48 (d, $^3J_{3,4}$ = 5.7 Hz, 1H, H-3), 4.03 (d, $^2J_{5a,5b}$ = 10.3 Hz, 1H, H-5a), 3.83 (dd, $^2J_{5b,5a}$ = 10.3 Hz, $^3J_{5b,4}$ = 4.0 Hz, 1H, H-5b), 3.26 (s, 3H, OCH_3), 1.73-1.50 (m, 8H, CH_2^{chex}), 1.47-1.34 (m, 2H, CH_2^{chex}); ^{13}C NMR (151 MHz, $CDCl_3$) δ = 118.77 (q, $^1J_{C,F}$ = 319.6 Hz, C^{Tf}), 114.08 (C^{chex}), 106.50 (C-2), 84.03 (C-3), 80.00 (C-4), 72.71 (C-5), 69.26 (C-1), 48.95 (OCH_3), 36.13 (CH_2^{chex}), 34.42 (CH_2^{chex}), 25.19 (CH_2^{chex}), 24.00 (CH_2^{chex}), 23.80 (CH_2^{chex}); ^{19}F NMR (659 MHz, $CDCl_3$) δ = −74.83 (s); HRMS (ESI+): m/z calc. for $C_{13}H_{19}F_3o_7SNa^+$ [M+Na]$^+$: 399.0696, found: 399.0687.

Synthesis of 3-deoxy-3-fluoro-D-ribulose (3DFRu, 4) and its potential radiolabelling precursor 35

3-*O*-Formyl-1,2-*O*-isopropylidene-β-D-*threo*-furanose & 1,2-*O*-isopropylidene-β-D-*threo*-furanose (17 & 18). A suspension of D-galactose (**16**, 8 g, 44.4 mmol, 1 equiv.) in water (44 mL) was added to glacial acetic acid (590 mL) and the mixture was heated to dissolution with a heat gun. Thereafter, the solution was cooled to 16°C and solid $Pb(OAc)_4$ (39.376 g, 88.8 mmol, 2 equiv., previously dried in high vacuum) was added in small portions over 10 min. The reaction mixture was stirred for 15 min at room temperature before the addition of oxalic acid dihydrate (10.077 g, 79.9 mmol, 1.8 equiv.) in glacial acetic acid (80 mL). Stirring was continued for 30 min, then the suspension was filtered over Celite® and the filtrate was concentrated at 50°C on a rotary evaporator followed drying in high vacuum. The remaining solid was suspended in EtOAc (240 mL) and the resulting suspension was washed with ice-cold water (2 × 16 mL). The organic layer was dried ($MgSO_4$), filtered and evaporated. The remaining yellow oil was dissolved in acetone (160 mL) under argon atmosphere, cooled to 0°C and sulfuric acid (0.828 mL, 1.52 g, 15.4 mmol, 0.35 equiv.) was added dropwise. The reaction mixture was warmed to room temperature and stirring was continued for 16 h. The mixture was neutralized by the addition of an aq. sat. $NaHCO_3$-

solution (80 mL). EtOAc (80 mL) was added, the layers were separated, and the organic Layer was extracted with an aq. sat. $NaHCO_3$-solution (8 × 50 mL). The organic layer was dried (($MgSO_4$), filtered and evaporated to give 3-*O*-formyl-1,2-*O*-isopropylidene-β-D-*threo*-furanose (**17**) as a yellow oil of sufficient purity for the next reaction (1.74 g, 21%): R_f = 0.59 (*n*hept/EtOAc 2:1, CAM); $[\alpha]_D^{20}$ = −28.0° (*c* 1.0, $CHCl_3$), {lit.[23] $[\alpha]_D^{25}$ = −39.8 (*c* 0.17, $CHCl_3$)}; ^{1}H NMR (500 MHz, $CDCl_3$): δ = 8.04 (s, 1H, H^{formyl}), 5.97 (d, $^3J_{1,2}$ = 3.7 Hz, 1H, H-1), 5.26 (d, $^3J_{3,4a}$ = 2.9 Hz, 1H, H-3), 4.60 (d, $^3J_{2,1}$ = 3.7 Hz, 1H, H-2), 4.19 (dd, $^2J_{4a,4b}$ = 10.9 Hz, $^3J_{4a,3}$ = 2.9 Hz, 1H, H-4a), 3.99 (d, $^2J_{4b,4a}$ = 10.9 Hz, 1H, H-4b), 1.51 (s, 3H, CH_3^{isopr}), 1.33 (s, 3H, CH_3^{isopr}); ^{13}C NMR (126 MHz, $CDCl_3$): δ = 159.93 (C^{formyl}), 112.41 (C^{isopr}), 105.50 (C-1), 83.05 (C-2), 76.84 (C-3), 70.53 (C-4), 26.83 (CH_3^{isopr}), 26.40 (CH_3^{isopr}).

NaCl (100 g) was added to the combined aq. layers, followed by extraction with EtOAc (3 × 100 mL). The combined org. layers were dried ($MgSO_4$), filtered and evaporated to give 1,2-*O*-isopropylidene-β-D-*threo*-furanose (**18**, 1.678 g, 24%) as a colorless solid: R_f = 0.22 (*n*hept/EtOAc 2:1, CAM); $[\alpha]_D^{20}$ = −7.6° (*c* 1.0, $CHCl_3$), {lit.[23] $[\alpha]_D^{25}$ = −10.6 (*c* 0.17, $CHCl_3$)}; ^{1}H-NMR (700 MHz, $CDCl_3$): δ = 5.96 (d, $^3J_{1,2}$ = 3.7 Hz, 1H, H-1), 4.50 (d, $^3J_{2,1}$ = 3.7 Hz, 1H, H-2), 4.28 (broad s, 1H, H-3), 4.10 (dd, $^2J_{4a,4b}$ = 10.2 Hz, $^3J_{4a,3}$ = 2.7 Hz, 1H, H-4a), 3.88 (dd, $^2J_{4b,4a}$ = 10.2 Hz, $^3J_{4b,3}$ = 1.0 Hz, 1H, H-4b), 1.76 (d, $^3J_{OH,3}$ = 4.5 Hz, 1H, OH), 1.48 (s, 3H, CH_3^{ipr}), 1.32 (s, 3H, CH_3^{ipr}); ^{13}C-NMR (176 MHz, $CDCl_3$): δ = 111.92 (C^{ipr}), 105.33 (C-1), 85.11 (C-2), 75.48 (C-3), 73.04 (C-4), 26.85 (CH_3^{ipr}), 26.35 (CH_3^{ipr}); HRMS (ESI+): m/z calc. for $C_7H_{12}O_4Na^+$ $[M+Na]^+$: 183.0628, found: 183.0628.

17 can be easily converted to **18** by the following procedure: 3-*O*-Formyl-1,2-*O*-isopropylidene-β-D-*threo*-furanose (**17**, 3.071 g, 16.32 mmol, 1 equiv.) was dissolved in methanol (8.33 mL) and solid K_2CO_3 (23 mg, 0.16 mmol, 0.01 equiv.) was added. The reaction mixture was stirred at room temperature for 16 h and concentrated. The crude residue was purified by MPLC (50 g silica gel, 12-100% EtOAc in *n*-heptane) to yield 1,2-*O*-isopropylidene-β-D-*threo*-furanose (**18**) as a colorless solid (1.969 g, 75%).

3-*O*-Benzyl-1,2-*O*-isopropylidene-β-D-*threo*-furanose (19). 1,2-*O*-Isopropylidene-β-D-*threo*-furanose (**18**, 4.66 g, 29.09 mmol, 1 equiv.) was dissolved in dry DMF (291 mL) under argon atmosphere and cooled to 0°C. Sodium hydride (1.746 g, 72.74 mmol, 2.5 equiv.) was added and the reaction mixture was stirred for 1 h at 0°C before dropwise addition of benzyl bromide (12.441 g, 8.64 mL, 72.7 mmol, 2.5 equiv.) . The reaction mixture was warmed to room temperature and stirred for 16 h. Then, MeOH (120 mL) was added dropwise at 0°C, followed by water (50 mL), and EtOAc (50 mL). The reaction mixture was allowed to warm to room temperature, the layers were separated, and the aq. layer was extracted with EtOAc (2 × 25 mL). The combined org. layers were washed with water (2 × 100 mL) and with brine (50 mL). The org. layer was dried ($MgSO_4$), filtered and evaporated. The crude residue was purified by MPLC (100 g silica gel, 5-20% EtOAc in *n*-heptane) to yield 3-*O*-benzyl-1,2-*O*-isopropylidene-β-D-*threo*-furanose (**19**) as a colorless oil (5.627 g, 77%): R_f = 0.24 (*n*hept/EtOAc 9:1, UV light and CAM); $[\alpha]_D^{20}$ = −24.2 (*c* 1.0, $CHCl_3$); ^{1}H-NMR (700 MHz, $CDCl_3$): δ = 7.37-7.32 (m, 4H, H^{Ar}), 7.31-7.28 (m, 1H, H^{Ar}), 5.96 (d, $^3J_{1,2}$ = 3.7 Hz, 1H, H-1), 4.62 (d, $^3J_{2,1}$ = 3.8 Hz, 1H, H-2), 4.57 (s, 2H, CH_2^{Bn}), 4.05-4.03 (m, 2H, H-4a & H-4b), 4.03-

4.02 (m, 1H, H-3), 1.48 (s, 3H, CH_3^{ipr}), 1.32 (s, 3H, CH_3^{ipr}); ^{13}C-NMR (176 MHz, $CDCl_3$): δ = 137.54 (C^{Ar}), 128.67 (2 CH^{Ar}), 128.06 (CH^{Ar}), 127.84 (2 CH^{Ar}), 111.74 (C^{ipr}), 105.69 (C-1), 83.09 (C-2), 82.05 (C-3), 71.36 (CH_2^{Bn}), 70.53 (C-4), 26.96 (CH_3^{ipr}), 26.41 (CH_3^{ipr}); HRMS (ESI+): m/z calc. for $C_{14}H_{18}O_4Na^+$ $[M+Na]^+$: 273.1097, found: 273.1098.

Methyl 3-*O*-benzyl-D-*threo*-furanoside (20). 3-*O*-Benzyl-1,2-*O*-isopropylidene-β-D-*threo*-furanose (**19**, 5.577 g, 22. mmol, 1 equiv.) was dissolved in methanol (290 mL) under argon atmosphere and cooled to 0°C. Acetyl chloride (12.715 g, 11.56 mL, 162.0 mmol, 7.27 equiv.) was added dropwise and the reaction was stirred at room temperature for 5 h. A sat. aq. $NaHCO_3$-solution (200 mL) was added, and the solvent was evaporated. The residue was redissolved in CH_2Cl_2 and washed with a sat. aq. $NaHCO_3$-solution (200 mL) and brine (200 mL). The organic layer was dried ($MgSO_4$), filtered and concentrated *in vacuo*. The crude residue was purified by MPLC (100 g silica gel, 10-55% EtOAc in *n*-heptane) to give methyl 3-*O*-benzyl-D-*threo*-furanoside (**20**) as a mixture of anomers as a colorless oil (4.445 g, 89%): R_f = 0.06 & 0.14 (*n*hept/EtOAc 3:1, UV light and CAM); $[\alpha]_D^{20}$ = +13.3 (*c* 1.0, $CHCl_3$); major anomer: ^{1}H NMR (600 MHz, $CDCl_3$) δ = 7.37-7.28 (m, 5H, H^{Ar}), 4.81 (s, 1H, H-1), 4.64 (d, $^2J_{H,H}$ = 12.1 Hz, 1H, CH_2^{Bn}), 4.57 (d, $^2J_{H,H}$ = 12.1 Hz, 1H, CH_2^{Bn}), 4.20 (broad s, 1H, H-2), 4.19 (dd, $^2J_{4a,4b}$ = 9.5 Hz, $^3J_{4a,3}$ = 6.5 Hz, 1H, H-4a), 3.96 (td, $^3J_{3,4a}$ = $^3J_{3,4b}$ = 6.5 Hz, $^3J_{3,2}$ = 2.2 Hz, 1H, H-3), 3.84 (dd, $^2J_{4b,4a}$ = 9.5 Hz, $^3J_{4b,3}$ = 6.5 Hz, 1H, H-4b), 3.39 (s, 3H, OCH_3); ^{13}C NMR (151 MHz, $CDCl_3$) δ = 137.84 (C^{Ar}), 128.67 (2 CH^{Ar}), 128.09 (3 CH^{Ar}), 109.54 (C-1), 84.29 (C-3), 80.62 (C-2), 72.46 (CH_2^{Bn}), 70.75 (C-4), 55.20 (OCH_3); minor anomer: ^{1}H NMR (600 MHz, $CDCl_3$) δ = 7.36-7.27 (m, 5H, H^{Ar}), 4.95 (d, $^3J_{1,2}$ = 4.2 Hz, 1H, H-1), 4.70 (d, $^2J_{H,H}$ = 11.8 Hz, 1H, CH_2^{Bn}), 4.56 (d, $^2J_{H,H}$ = 11.8 Hz, 1H, CH_2^{Bn}), 4.23 (t, $^3J_{2,1}$ = $^3J_{2,3}$ = 4.2 Hz, 1H, H-2), 4.09 (dd, $^2J_{4a,4b}$ = 9.6 Hz, $^3J_{4a,3}$ = 6.0 Hz, 1H, H-4a), 4.04 (dt, $^3J_{3,4a}$ = 6.0 Hz, $^3J_{3,2}$ = $^3J_{3,4b}$ = 4.2 Hz, 1H, H-3), 3.80 (dd, $^2J_{4b,4a}$ = 9.6 Hz, $^3J_{4b,3}$ = 4.2 Hz, 1H, H-4b), 3.46 (s, 3H, OCH_3); ^{13}C NMR (151 MHz, $CDCl_3$) δ = 137.96 (C^{Ar}), 128.60 (2 CH^{Ar}), 127.96 (2 CH^{Ar}), 127.94 (CH-Ar), 102.93 (C-1), 83.75 (C-3), 77.84 (C-2), 71.69 (CH_2^{Bn}), 69.97 (C-4), 55.63 (OCH_3); HRMS (ESI+): m/z calc. for $C_{12}H_{16}O_4Na^+$ $[M+Na]^+$: 247.0941, found: 247.0941.

Methyl 3-*O*-benzyl-2-*O*-trifluoromethanesulfonyl-D-*threo*-furanoside (21). Methyl 3-*O*-benzyl-D-*threo*-furanoside (**20**, 1.533 mg, 6.84 mmol, 1 equiv.) was dissolved in dry CH_2Cl_2 (34 mL) under argon atmosphere and cooled to 0°C. Dry pyridine (4.596 g, 4.69 mL, 58.1 mmol, 8.5 equiv.) and trifluoromethanesulfonic anhydride (3.857 g, 2.3 mL, 13.67 mmol, 2 equiv.) were added dropwise and the reaction mixture was stirred for 1 h at 0°C, before dropwise addition of ice-cold aq. HCl (1 M, 34 mL). The layers were separated, and the aqueous layer was extracted with CH_2Cl_2 (2 × 20 mL). The combined organic layers were washed with an ice-cold sat. aq. $NaHCO_3$-sol. (20 mL), ice-cold water (30mL) and ice-cold brine (30 mL). The organic layer was dried ($MgSO_4$), filtered and evaporated to give methyl 3-*O*-benzyl-2-*O*-trifluoromethanesulfonyl-D-*threo*-furanoside (**21**) as amixture of anomers as red oil (2.191 g, 90%) of sufficient purity for the following reaction: R_f = 0.37 (*n*hept/EtOAc 9:1, UV light and CAM); $[\alpha]_D^{20}$ = +2.6 (*c* 1.0, $CHCl_3$); major anomer: ^{1}H NMR (700 MHz, $CDCl_3$) δ = 7.38-7.35 (m, 2H, H^{Ar}), 7.34-7.29 (m, 3H, H^{Ar}), 5.19 (s, 1H, H-2), 5.08-5.05 (m, 1H, H-1), 4.72 (d, $^2J_{H,H}$ = 12.2 Hz, 1H, CH_2^{Bn}), 4.54 (d, $^2J_{H,H}$ = 12.2 Hz, 1H, CH_2^{Bn}), 4.26-4.22 (m, 2H, H-3 & H-4a), 3.85 (dd, $^2J_{4b,4a}$ = 12.5 Hz, $^3J_{4b,3}$ = 8.9 Hz, 1H, H-4b), 3.41 (s, 3H, OCH_3); ^{13}C

NMR (176 MHz, $CDCl_3$) δ = 136.73 (C^{Ar}), 128.80 (3 CH^{Ar}), 128.10 (2 CH^{Ar}), 106.04 (C-1), 92.12 (C-2), 81.43 (C-3), 72.75 (CH_2^{Bn}), 70.69 (C-4), 55.19 (OCH_3); ^{19}F NMR (659 MHz, $CDCl_3$) δ = −75.26 (s); minor anomer: ^{1}H NMR (700 MHz, $CDCl_3$) δ = 7.39-7.35 (m, 2H, H^{Ar}), 7.34-7.30 (m, 3H, H^{Ar}), 5.08-5.04 (m, 2H, H-1 & H-2), 4.64 (d, $^2J_{H,H}$ = 12.3 Hz, 1H, CH_2^{Bn}), 4.53 (d, $^2J_{H,H}$ = 12.3 Hz, 1H, CH_2^{Bn}), 4.44 (dt, $^3J_{3,4a}$ = 6.9 Hz, $^3J_{3,2}$ = $^3J_{3,4b}$ = 4.0 Hz, 1H, H-3), 4.11 (dd, $^2J_{4a,4b}$ = 9.7 Hz, $^3J_{4a,3}$ = 6.9 Hz, 1H, H-4a), 3.79 (dd, $^2J_{4b,4a}$ = 9.7 Hz, $^3J_{4b,3}$ = 4.0 Hz, 1H, H-4b), 3.43 (s, 3H, OCH_3); ^{13}C NMR (176 MHz, $CDCl_3$) δ = 136.83 (C^{Ar}), 128.45 (2 CH^{Ar}), 128.42 (CH^{Ar}), 128.00 (2 CH^{Ar}), 100.99 (C-1), 88.58 (C-2), 79.56 (C-3), 72.53 (CH_2^{Bn}), 68.84 (C-4), 55.74 (OCH_3); ^{19}F NMR (659 MHz, $CDCl_3$) δ = −75.16 (s); HRMS (ESI+): m/z calc. for $C_{13}H_{15}F_3O_6SNa^+$ $[M+Na]^+$: 379.0434, found: 379.0437.

Methyl 3-*O*-benzyl-2-deoxy-2-fluoro-D-*erythro*-furanoside (22). The crude triflate **21** (385 mg, 1.7 mmol, 1 equiv.) was dissolved in *tert*-amylalcohol (10 mL) in a 20 mL microwave vial. Cesium fluoride (1.57 g, 10.3 mmol, 4 equiv.) was added, the vial was flushed with argon and sealed. The mixture was stirred for 24 h at 90°C, cooled to room temperature, diluted with water (10 mL) and extracted with Et_2O (3 × 15 mL). The combined organic layers were washed with water (15 mL), a sat. aq. $NaHCO_3$-solution (15 mL) and brine (15 mL), dried ($MgSO_4$), filtered and evaporated. The crude residue was purified by MPLC (100 g silica gel, 2-20% EtOAc in *n*-heptane) to give methyl 3-*O*-benzyl-2-deoxy-2-fluoro-D-*erythro*-furanoside (**22**) as a mixture of anomers as colorless oil (543 mg, 35%): major anomer: R_f = 0.25 (nhept/EtOAc 9:1, UV light and CAM); $[\alpha]_D^{20}$ = −54.9 (*c* 1.0, $CHCl_3$); ^{1}H NMR (600 MHz, $CDCl_3$) δ = 7.38-7.34 (m, 4H, H^{Ar}), 7.34-7.29 (m, 1H, H^{Ar}), 5.03 (d, $^3J_{1,F}$ = 10.3 Hz, 1H, H-1), 4.78 (dd, $^2J_{2,F}$ = 52.9 Hz, $^3J_{2,3}$ = 4.0 Hz, 1H, H-2), 4.69 (d, $^2J_{H,H}$ = 11.7 Hz, 1H, CH_2^{Bn}), 4.55 (d, $^2J_{H,H}$ = 11.7 Hz, 1H, CH_2^{Bn}), 4.31 (dtd, $^3J_{3,F}$ = 21.0 Hz, $^3J_{3,4a}$ = $^3J_{3,4b}$ = 7.0 Hz, $^3J_{3,2}$ = 4.0 Hz, 1H, H-3), 4.10 (dd, $^2J_{4a,4b}$ = 8.8 Hz, $^3J_{4a,3}$ = 7.0 Hz, 1H, H-4a), 3.90 (dd, $^2J_{4b,4a}$ = 8.8, $^3J_{4b,3}$ =7.0 Hz, 1H, H-4b), 3.34 (s, 3H, OCH_3); ^{13}C NMR (151 MHz, $CDCl_3$) δ = 137.50 (C^{Ar}), 128.69 (2 CH^{Ar}), 128.23 (CH^{Ar}), 128.09 (2 CH^{Ar}), 106.20 (d, $^2J_{1,F}$ = 29.8 Hz, C-1), 91.39 (d, $^1J_{2,F}$ = 186.4 Hz, C-2), 76.65 (d, $^2J_{3,F}$ = 15.2 Hz, C-3), 72.94 (CH_2^{Bn}), 69.36 (C-4), 55.21 (OCH_3); ^{19}F NMR (659 MHz, $CDCl_3$) δ = −210.80 (ddd, $^2J_{F,2}$ = 52.9 Hz, $^3J_{F,3}$ = 21.0 Hz, $^3J_{F,1}$ = 10.3 Hz); minor anomer: R_f = 0.08 (*n*hept/EtOAc 9:1, UV light and CAM); $[\alpha]_D^{20}$ = +162.6 (*c* 1.0, $CHCl_3$); ^{1}H NMR (600 MHz, $CDCl_3$) δ = 7.38-7.33 (m, 4H, H^{Ar}), 7.31-7.28 (m, 1H, H^{Ar}), 4.98 (dd, $^3J_{1,2}$ = 4.3 Hz, $^3J_{1,F}$ = 3.1 Hz, 1H, H-1), 4.82 (ddd, $^2J_{2,F}$ = 51.0 Hz, $^3J_{2,3}$ = 5.6 Hz, $^3J_{2,1}$ = 4.3 Hz, 1H, H-2), 4.80 (d, $^2J_{H,H}$ = 12.3 Hz, 1H, CH_2^{Bn}), 4.59 (d, $^2J_{H,H}$ = 12.4 Hz, 1H, CH_2^{Bn}), 4.08-4.02 (m, 2H, H-3 & H-4a), 3.96-3.91 (m, 1H, H-4b), 3.50 (s, 3H, OCH_3); ^{13}C NMR (151 MHz, $CDCl_3$) δ = 137.83 (C^{Ar}), 128.64 (2 CH^{Ar}), 128.06 (CH^{Ar}), 128.03 (2 CH^{Ar}), 101.27 (d, $^2J_{1,F}$ = 15.8 Hz, C-1), 88.51 (d, $^1J_{2,F}$ = 203.8 Hz, C-2), 73.50 (d, $^2J_{3,F}$ = 14.8 Hz, C-3), 72.90 (d, $^4J_{H,F}$ = 2.5 Hz, CH_2-Bn), 69.81 (d, $^3J_{4,F}$ = 2.4 Hz, C-4), 55.97 (OCH_3); ^{19}F NMR (565 MHz, $CDCl_3$) δ = −218.15 (ddd, $^2J_{F,2}$ = 51.0 Hz, $^3J_{F,3}$ = 6.7 Hz, $^3J_{F,1}$ = 3.1 Hz); HRMS (ESI+): m/z calc. for $C_{12}H_{15}FO_3Na^+$ $[M+Na]^+$: 249.0897, found: 249.0897.

3-*O*-Benzyl-2-deoxy-2-fluoro-D-erythrose (23). Methyl 3-*O*-benzyl-2-deoxy-2-fluoro-D-*erythro*-furanoside (**22**, 546 mg, 2.4 mmol, 1 equiv.) was dissolved in an aq. solution of trifluoroacetic acid [TFA, 80% (v/v)]. The solution was stirred for 2 h at room temperature. All volatiles were evaporated under reduced pressure and the crude residue was purified by

MPLC (25 g silica gel, 5-40% EtOAc in *n*-heptane) to give 3-*O*-benzyl-2-deoxy-2-fluoro-D-erythrose (**23**) as a mixture of anomers as a colorless solid (380 mg, 74%): R_f = 0.23 (*n*hept/EtOAc 2:1, UV light and CAM); $[\alpha]_D^{20}$ = +10.2 (*c* 1.0, $CHCl_3$); <u>major anomer</u>: ^{1}H NMR (600 MHz, $CDCl_3$) δ = 7.39-7.30 (m, 5H, H^{Ar}), 5.50 (dd, $^3J_{1,F}$ = 10.2 Hz, $^3J_{1,OH}$ = 3.0 Hz, 1H, H-1), 4.83 (dd, $^2J_{2,F}$ = 52.7 Hz, $^3J_{2,3}$ = 3.8 Hz, 1H, H-2), 4.70 (d, $^2J_{H,H}$ = 11.7 Hz, 1H, CH_2^{Bn}), 4.57 (d, $^2J_{H,H}$ = 11.7 Hz, 1H, CH_2^{Bn}), 4.39 (dtd, $^3J_{3,F}$ = 21.4 Hz, $^3J_{3,4a}$ = $^3J_{3,4b}$ = 7.4 Hz, $^3J_{3,2}$ = 3.8 Hz, 1H, H-3), 4.22 (dd, $^2J_{4a,4b}$ = 8.6 Hz, $^3J_{4a,3}$ = 7.4 Hz, 1H, H-4a), 3.91 (dd, $^2J_{4b,4a}$ = 8.6 Hz, $^3J_{4b,3}$ = 7.4 Hz, 1H, H-4b), 2.50 (t, $^3J_{OH,1}$ = $^4J_{OH,F}$ = 3.0 Hz, 1H, OH); ^{13}C NMR (151 MHz, $CDCl_3$) δ = 137.43 (C^{Ar}), 128.71 (2 CH^{Ar}), 128.28 (CH^{Ar}), 128.11 (2 CH^{Ar}), 100.23 (d, $^2J_{1,F}$ = 30.5 Hz, C-1), 91.83 (d, $^1J_{2,F}$ = 186.4 Hz, C-2), 76.35 (d, $^2J_{3,F}$ = 15.3 Hz, C-3), 73.00 (CH_2^{Bn}), 69.52 (C-4); ^{19}F NMR (659 MHz, $CDCl_3$) δ = −210.79 (ddd, $^1J_{F,2}$ = 52.4 Hz, $^2J_{F,3}$ = 21.5 Hz, $^2J_{F,1}$ = 10.2 Hz); <u>minor anomer</u>: ^{1}H NMR (600 MHz, $CDCl_3$) δ = 7.39-7.30 (m, 5H, H^{Ar}), 5.27 (dd, $^3J_{1,OH}$ = 12.2 Hz, $^3J_{1,2}$ = 3.8 Hz, 1H, H-1), 4.87 (dt, $^2J_{2,F}$ = 50.9 Hz, $^3J_{2,1}$ = $^3J_{2,3}$ = 4.4 Hz, 1H, H-2), 4.77 (d, $^2J_{H,H}$ = 11.8 Hz, 1H, CH_2^{Bn}), 4.66 (d, $^2J_{H,H}$ = 11.8 Hz, 1H, CH_2^{Bn}), 4.17 (qd, $^3J_{3,2}$ = $^3J_{3,4b}$ = $^3J_{3,F}$ = 4.4 Hz, $^3J_{3,4a}$ = 2.5 Hz, 1H, H-3), 4.13 (dt, $^2J_{4a,4b}$ = 9.6 Hz, $^3J_{4a,3}$ = $^4J_{4a,F}$ =2.5 Hz, 1H, H-4a), 3.97 (dd, $^2J_{4b,4a}$ = 9.6 Hz, $^3J_{4b,3}$ = 4.4 Hz, 1H, H-4b), 3.95 (dd, $^3J_{OH,1}$ = 12.2 Hz, $^4J_{OH,F}$ = 2.1 Hz, 1H, OH); ^{13}C NMR (151 MHz, $CDCl_3$) δ = 137.002 (C^{Ar}), 128.80 (2 CH^{Ar}), 128.40 (CH^{Ar}), 128.02 (2 CH^{Ar}), 95.24 (d, $^2J_{1,F}$ = 18.8 Hz, C-1), 89.29 (d, $^1J_{2,F}$ = 200.3 Hz, C-2), 75.71 (d, $^2J_{3,F}$ = 14.6 Hz, C-3), 73.21 (d, $^4J_{C,F}$ = 2.3 Hz, CH_2^{Bn}), 69.50 (d, $^3J_{4,F}$ = 3.1 Hz, C-4); ^{19}F NMR (659 MHz, $CDCl_3$) δ = −217.82 (ddd, $^2J_{F,2}$ = 50.9 Hz, $^3J_{F,3}$ = 4.4 Hz, $^4J_{F,OH}$ = 2.1 Hz); HRMS (ESI+): m/z calc. for $C_{11}H_{13}O_3FNa^+$ $[M+Na]^+$: 235.0741, found: 235.0742, m/z calc. for $C_{12}H_{17}O_4FNa^+$ $[M+MeOH+Na]^+$: 267.1003, found: 267.1007.

3-*O*-Benzyl-2-deoxy-2-fluoro-D-*erythro*-1,4-lactone (24). 3-*O*-Benzyl-2-deoxy-2-fluoro-D-erythrose (**23**, 580 mg, 2.7 mmol, 1 equiv.) was dissolved in dry CH_2Cl_2 (22.8 mL) under argon atmosphere. NMO (480 mg, 4.1 mmol, 1.5 equiv.) was added and the mixture was stirred for 10 min at room temperature before TPAP (48 mg, 0.1 mmol, 0.05 equiv.) was added. The reaction mixture was stirred for 16 h at room temperature, filtered over a short plug of silica gel (EtOAc). The filtrate was evaporated and the crude residue was purified by MPLC (50 g silica gel, 8-58% EtOAc in *n*-heptane) to yield 3-*O*-benzyl-2-deoxy-2-fluoro-D-*erythro*-1,4-lactone (**24**) as a colorless solid (366 mg, 64%): R_f = 0.17 (*n*hept/EtOAc 2:1, CAM); $[\alpha]_D^{20}$ = +9.6 (*c* 1.0, $CHCl_3$); ^{1}H NMR (700 MHz, $CDCl_3$) δ = 7.39-7.31 (m, 5H, H-Ar), 5.20 (dd, $^2J_{2,F}$ = 48.6 Hz, $^3J_{2,3}$ = 4.2 Hz, 1H, H-2), 4.83 (d, $^2J_{H,H}$ = 11.9 Hz, 1H, CH_2^{Bn}), 4.65 (d, $^2J_{H,H}$ = 11.9 Hz, 1H, CH_2^{Bn}), 4.42 (ddd, $^2J_{4a,4b}$ = 10.6 Hz, $^4J_{4a,F}$ = 4.2 Hz, $^3J_{4a,3}$ = 0.7 Hz, 1H, H-4a), 4.39 (ddd, $^3J_{3,F}$ = $^3J_{3,4b}$ = $^3J_{2,3}$ = 4.2 Hz, 1H, H-3), 4.31 (ddd, $^2J_{4b,4a}$ = 10.6, $^3J_{4b,3}$ = 4.2, $^4J_{4b,F}$ = 1.3 Hz, 1H, H-4b); ^{13}C NMR (176 MHz, $CDCl_3$) δ = 169.92 (d, $^2J_{1,F}$ = 22.7 Hz, C-1), 136.83 (C^{Ar}), 128.79 (2 CH^{Ar}), 128.45 (CH^{Ar}), 128.02 (2 CH^{Ar}), 86.16 (d, $^1J_{2,F}$ = 206.0 Hz, C-2), 73.24 (d, $^2J_{3,F}$ = 14.3 Hz, C-3), 73.04 (d, $^4J_{C,F}$ = 3.2 Hz, CH_2^{Bn}), 69.67 (d, $^3J_{4,F}$ = 5.3 Hz, C-4); ^{19}F NMR (659 MHz, $CDCl_3$) δ = −217.91 (dd, $^2J_{F,2}$ = 48.6 Hz, $^3J_{F,3}$ = 4.2 Hz); HRMS (ESI+): m/z calc. for $C_{11}H_{11}O_3FNa^+$ $[M+Na]^+$: 233.0584, found: 233.0583, m/z calc. for $C_{12}H_{15}O_4FNa^+$ $[M+MeOH+Na]^+$: 265.0847, found: 265.0846.

2,5-Anhydro-4-*O*-benzyl-3-deoxy-3-fluoro-D-*erythro*-pent-1-enitol (25). This reaction is light sensitive and was carried out in the dark. 3-*O*-Benzyl-2-deoxy-2-fluoro-D-*erythro*-1,4-

lactone (**24**, 366 mg, 1.7 mmol, 1 equiv.) was suspended in dry toluene (8.7 mL) under argon atmosphere and Petasis' reagent (0.5 M in toluene, 5.224 mL, 544 mg, 2.6 mmol, 1.5 equiv.) was added. The flask was equipped with a reflux condenser and the solution was stirred at 70°C for 16 h in the dark. The reaction mixture was cooled to room temperature, diluted with *n*-pentane (40 mL) and stirring was continued for 30 min. The orange suspension was filtered through a pad of Celite® and concentrated. The crude residuewas purified by MPLC (100 g silica gel, 1-10% Et_2O in *n*-pentane) to give 2,5-anhydro-4-*O*-benzyl-3-deoxy-3-fluoro-D-*erythro*-pent-1-enitol (**25**) as a yellow oil (233 mg, 64%) which was immediately used in the next step: R_f = 0.24 (9:1 *n*hept/EtOAc, UV light and CAM); $[\alpha]_D^{20}$ = +70.7 (*c* 1.0, $CHCl_3$); ^{1}H NMR (600 MHz, $CDCl_3$) δ = 7.39-7.35 (m, 4H, H^{Ar}), 7.35-7.31 (m, 1H, H^{Ar}), 5.12 (dd, $^2J_{3,F}$ = 55.4 Hz, $^3J_{3,4}$ = 3.8 Hz, 1H, H-3), 4.76 (d, $^2J_{H,H}$ = 11.8 Hz, 1H, CH_2^{Bn}), 4.62 (d, $^2J_{H,H}$ = 11.8 Hz, 1H, CH_2^{Bn}), 4.55 (dd, $^4J_{1a,F}$ = 5.5 Hz, $^2J_{1a,1b}$ = 2.1 Hz, 1H, H-1a), 4.36 (dd, $^4J_{1b,F}$ = 5.6 Hz, $^2J_{1b,1a}$ = 2.1 Hz, 1H, H-1b), 4.17 (dd, $^2J_{5a,5b}$ = 7.6 Hz, $^3J_{5a,4}$ = 5.9 Hz, 1H, H-5a), 4.12 (dddd, $^3J_{4,F}$ = 16.7 Hz, $^3J_{4,5b}$ = 7.6 Hz, $^3J_{4,5a}$ = 5.9 Hz, $^3J_{4,3}$ = 3.8 Hz, 1H, H-4), 4.08 (td, $^2J_{5b,5a}$ = $^3J_{5b,4}$ = 7.6 Hz, $^4J_{5b,F}$ = 1.8 Hz, 1H, H-5b); ^{13}C NMR (151 MHz, $CDCl_3$) δ = 158.68 (d, $^2J_{2,F}$ = 14.5 Hz, C-2), 137.27 (C^{Ar}), 128.76 (2 CH^{Ar}), 128.37 (CH^{Ar}), 128.12 (2 CH^{Ar}), 88.15 (d, $^3J_{1,C}$ = 8.3 Hz, C-1), 88.08 (d, $^1J_{3,F}$ = 187.5 Hz, C-3), 76.22 (d, $^2J_{4,F}$ = 16.5 Hz, C-4), 72.72 (CH_2^{Bn}), 70.55 (d, $^3J_{5,F}$ = 1.5 Hz, C-5); ^{19}F NMR (659 MHz, $CDCl_3$) δ = −193.65 (ddt, $^2J_{F,3}$ = 55.4 Hz, $^3J_{F,4}$ = 16.7 Hz, $^4J_{F,1a}$ = $^4J_{F,1b}$ = 5.5 Hz); HRMS (ESI+): m/z calc. for $C_{12}H_{13}O_2FNa^+$ $[M+Na]^+$: 231.0792, found: 231.0789.

4-*O*-Benzyl-3-deoxy-3-fluoro-D-ribulose (26). 2,5-Anhydro-4-*O*-benzyl-3-deoxy-3-fluoro-D-*erythro*-pent-1-enitol (**25**, 207 mg, 1.0 mmol, 1 equiv.) was dissolved in a 4:1 mixture of acetone and H_2O (20 mL) and NMO (233 mg, 2.0 mmol, 2 equiv.) was added. Potassium osmate dihydrate (18 mg, 0.05 mmol, 0.05 equiv.) was added and the mixture was stirred for 24 h at room temperature, diluted with EtOAc (20 mL) and the organic layer was washed with water (15 mL) and brine (15 mL). The organic layer was dried ($MgSO_4$), filtered and evaporated. The crude residue was purified by MPLC (25 g silica gel, 16-100% EtOAc in *n*-heptane) to yield 4-*O*-benzyl-3-deoxy-3-fluoro-D-ribulose (**26**) as a colorless oil as a mixture of both anomers and the corresponding open chain form (161 mg, 67%): R_f = 0.21 (*n*hept/EtOAc 1:2, UV light and CAM); $[\alpha]_D^{20}$ = +9.4 (*c* 1.0, $CHCl_3$); major anomer: ^{1}H NMR (700 MHz, $CDCl_3$) δ = 7.39-7.30 (m, 5H, H^{Ar}), 5.01 (dd, $^2J_{3,F}$ = 50.7 Hz, $^3J_{3,4}$ = 4.5 Hz, 1H, H-3), 4.79 (d, $^2J_{H,H}$ = 11.7 Hz, 1H, CH_2^{Bn}), 4.67 (d, $^2J_{H,H}$ = 11.7 Hz, 1H, CH_2^{Bn}), 4.33 (d, $^4J_{OH,F}$ = 2.7 Hz, 1H, OH-2), 4.23 (qd, $^3J_{4,3}$ = $^3J_{4,F}$ = $^3J_{4,5b}$ = 4.5 Hz, $^3J_{4,5a}$ = 2.6 Hz, 1H, H-4), 4.14 (dt, $^2J_{5a,5b}$ = 10.2 Hz, $^3J_{5a,4}$ = $^4J_{5a,F}$ = 2.6 Hz, 1H, H-5a), 3.97 (ddd, $^2J_{5b,5a}$ = 10.2 Hz, $^3J_{5b,4}$ = 4.5 Hz, $^4J_{5b,F}$ = 1.3 Hz, 1H, H-5b), 3.69 (ddd, $^2J_{1a,1b}$ = 12.0 Hz, $^3J_{1a,OH}$ = 9.1 Hz, $^4J_{1a,F}$ = 0.7 Hz, 1H, H-1a), 3.62 (dd, $^2J_{1b,1a}$ = 12.0 Hz, $^3J_{1b,OH}$ = 4.8 Hz, 1H, H-1b), 1.81 (dd, $^3J_{OH,1a}$ = 9.1 Hz, $^3J_{OH,1b}$ = 4.8 Hz, 1H, OH-1); ^{13}C NMR (176 MHz, $CDCl_3$) δ = 136.83 (C^{Ar}), 128.66 (2 CH^{Ar}), 128.26 (CH^{Ar}), 127.85 (2 CH^{Ar}), 101.65 (d, $^2J_{2,F}$ = 17.9 Hz, C-2), 88.36 (d, $^1J_{3,F}$ = 200.6 Hz, C-3), 76.33 (d, $^2J_{4,F}$ = 14.5 Hz, C-4), 73.07 (d, $^4J_{C,F}$ = 2.4 Hz, CH_2^{Bn}), 69.32 (d, $^3J_{5,F}$ = 5.3 Hz, C-5), 63.25 (C-1); ^{19}F NMR (659 MHz, $CDCl_3$) δ = −216.52 (d, $^2J_{F,3}$ = 50.7 Hz); minor anomer: ^{1}H NMR (700 MHz, $CDCl_3$) δ = 7.39-7.30 (m, 5H, H^{Ar}), 4.83 (dd, $^2J_{3,F}$ = 53.5 Hz, $^3J_{3,4}$ = 3.9 Hz, 1H, H-3), 4.69 (d, $^2J_{H,H}$ = 11.7 Hz, 1H, CH_2^{Bn}), 4.57 (d, $^2J_{H,H}$ = 11.7 Hz, 1H, CH_2^{Bn}), 4.47 (dtd, $^3J_{4,F}$ = 21.0 Hz, $^3J_{4,5a}$ = $^3J_{4,5b}$ = 7.2 Hz, $^3J_{4,3}$ = 3.9 Hz, 1H, H-4), 4.21 (dd, $^2J_{5a,5b}$ = 8.8 Hz,

$^3J_{5a,4}$ = 7.2 Hz, 1H, H-5a), 3.91 (dd, $^2J_{5b,5a}$ = 8.8 Hz, $^3J_{5b,4}$ = 7.2 Hz, 1H, H-5b), 3.84-3.80 (m, 2H, H-1a & H-1b), 3.37 (d, $^4J_{OH,F}$ = 3.1 Hz, 1H, OH-2), 1.94 (dd, $^3J_{OH,1a\ or\ 1b}$ = 7.2 Hz, $^3J_{OH,1a\ or\ 1b}$ = 6.0 Hz, 1H, OH-1); ^{13}C NMR (176 MHz, $CDCl_3$) δ = 137.39 (C^{Ar}), 128.82 (CH^{Ar}), 128.72 (2 CH^{Ar}), 128.10 (2 CH^{Ar}), 104.69 (d, $^2J_{2,F}$ = 24.6 Hz, C-2), 91.75 (d, $^1J_{3,F}$ = 189.5 Hz, C-3), 76.96 (d, $^2J_{4,F}$ = 15.8 Hz, C-4), 72.98 (CH_2^{Bn}), 69.62 (C-5), 64.10 (d, $^3J_{1,F}$ = 6.2 Hz, C-1); ^{19}F NMR (659 MHz, $CDCl_3$) δ = −214.85 (dd, $^2J_{F,3}$ = 53.5 Hz, $^3J_{F,4}$ = 21.0 Hz); open chain form: ^{1}H NMR (700 MHz, $CDCl_3$) δ = 7.39-7.31 (m, 5H, H^{Ar}), 5.18 (dd, $^2J_{3,F}$ = 47.9 Hz, $^3J_{3,4}$ = 2.8 Hz, 1H, H-3), 4.72 (d, $^2J_{H,H}$ = 11.7 Hz, 1H, CH_2^{Bn}), 4.64 (d, $^2J_{H,H}$ = 11.7 Hz, 1H, CH_2^{Bn}), 4.55 (dd, $^3J_{1,OH}$ = 5.0 Hz, $^4J_{1,F}$ = 1.8 Hz, 2H, CH_2-1), 4.04 (dddd, $^3J_{4,F}$ = 20.9 Hz, $^3J_{4,5a\ or\ 5b}$ = 6.7 Hz, $^3J_{4,5a\ or\ 5b}$ = 6.2 Hz, $^3J_{4,3}$ = 2.8 Hz, 1H, H-4), 3.80-3.75 (m, 2H, H-5a & H-5b), 2.85 (t, $^3J_{OH,1}$ = 5.0 Hz, 1H, OH-1), 1.75 (dd, $^3J_{OH,5a\ or\ 5b}$ = 6.5 Hz, $^3J_{OH,5a\ or\ 5b}$ = 5.4 Hz, 1H, OH-5); ^{13}C NMR (176 MHz, $CDCl_3$) δ = 207.82 (d, $^2J_{2,F}$ = 26.4 Hz, C-2), 137.16 (C^{Ar}), 128.44 (CH^{Ar}), 128.30 (2 CH^{Ar}), 128.08 (2 CH^{Ar}), 94.37 (d, $^1J_{3,F}$ = 186.7 Hz, C-3), 79.72 (d, $^2J_{4,F}$ = 19.8 Hz, C-4), 73.48 (CH_2^{Bn}), 67.38 (d, $^3J_{1,F}$ = 4.2 Hz, C-1), 60.14 (d, $^3J_{5,F}$ = 8.4 Hz, C-5); ^{19}F NMR (659 MHz, $CDCl_3$) δ = −210.52 (dd, $^2J_{F,3}$ = 47.9 Hz, $^3J_{F,4}$ = 20.9 Hz); HRMS (ESI+): m/z calc. for $C_{12}H_{15}O_4FNa^+$ $[M+Na]^+$: 265.0847, found: 265.0853.

3-Deoxy-3-fluoro-D-ribulose (3DFRu, 4). 4-*O*-Benzyl-3-deoxy-3-fluoro-D-ribulose (**26**, 156 mg, 0.6 mmol, 1 equiv.) was dissolved in methanol (8.5 mL) and palladium on charcoal (10% Pd, 69 mg, 0.06 mmol, 0.1 equiv.) was added. The mixture was degassed (3 freezing and thawing cycles under vacuum using liquid N_2 to cool the reaction mixture) and stirred under H_2-atmosphere (1 atm) for 16 h at room temperature. The suspension was filtered over a pad of Celite®, concentrated, and the crude residue was purified by reversed-phase chromatography (C_{18}-silica gel, H_2O). This gave3-deoxy-3-fluoro-D-ribulose (**3DFRu**, **4**) as a colorless oil as a 2.1 : 2 : 1 mixture of both anomers and the respective open chain conformation (69 mg, 70%): R_f = 0.19 (EtOAc, CAM); $[\alpha]_D^{20}$ = −16.1 (*c* 1.0, H_2O); major anomer: ^{1}H NMR (700 MHz, D_2O) δ = 4.93 (dd, $^2J_{3,F}$ = 52.2 Hz, $^3J_{3,4}$ = 5.8 Hz, 1H, H-3), 4.50 (dq, $^3J_{4,F}$ = 12.4 Hz, $^3J_{4,3}$ = $^3J_{4,5a}$ = $^3J_{4,5b}$ = 5.8 Hz, 1H, H-4), 4.11 (dd, $^2J_{5a,5b}$ = 9.4 Hz, $^3J_{5a,4}$ = 5.8 Hz, 1H, H-5a), 3.94 (ddd, $^2J_{5b,5a}$ = 9.3 Hz, $^3J_{5b,4}$ = 5.8 Hz, $^4J_{5b,F}$ = 1.4 Hz, 1H, H-5b), 3.64 (dd, $^2J_{1a,1b}$ = 12.2 Hz, $^4J_{1a,F}$ = 1.4 Hz, 1H, H-1a), 3.57 (d, $^2J_{1a,1b}$ = 12.1 Hz, 1H, H-1b); ^{13}C NMR (176 MHz, D_2O) δ = 105.22 (d, $^2J_{2,F}$ = 16.2 Hz, C-2), 92.51 (d, $^1J_{3,F}$ = 193.1 Hz, C-3), 72.78 (d, $^3J_{5,F}$ = 3.0 Hz, C-5), 71.60 (d, $^2J_{4,F}$ = 16.0 Hz, C-4), 66.06 (C-1); ^{19}F NMR (659 MHz, D_2O) δ = −213.53 (dd, $^2J_{F,3}$ = 52.2 Hz, $^3J_{F,4}$ = 12.4 Hz); minor anomer: ^{1}H NMR (700 MHz, D_2O) δ = 4.86 (dd, $^2J_{3,F}$ = 53.4 Hz, $^3J_{3,4}$ = 4.2 Hz, 1H, H-3), 4.75 (dtd, $^3J_{4,F}$ = 21.0 Hz, $^3J_{4,5a}$ = $^3J_{4,5b}$ = 7.1 Hz, $^3J_{4,3}$ = 4.2 Hz, 1H, H-4), 4.30 (dd, $^2J_{5a,5b}$ = 8.9 Hz, $^3J_{5a,4}$ = 7.1 Hz, 1H, H-5a), 3.85 (dd, $^2J_{1a,1b}$ = 12.1 Hz, $^4J_{1a,F}$ = 2.6 Hz, 1H, H-1a), 3.80 (dd, $^2J_{5b,5a}$ = 8.9 Hz, $^3J_{5b,4}$ = 7.1 Hz, 1H, H-5b), 3.65 (dd, $^2J_{1b,1a}$ = 12.1 Hz, $^4J_{1b,F}$ = 4.4 Hz, 1H, H-1b); ^{13}C NMR (176 MHz, D_2O) δ 107.63 (d, $^2J_{2,F}$ = 23.2 Hz, C-2), 96.03 (d, $^1J_{3,F}$ = 186.9 Hz, C-3), 72.93 (d, $^3J_{5,F}$ = 1.5 Hz, C-5), 72.13 (d, $^2J_{4,F}$ = 16.1 Hz, C-4), 64.58 (d, $^3J_{1,F}$ = 5.2 Hz, C-1); ^{19}F NMR (659 MHz, D_2O) δ = −210.50 (dd, $^2J_{F,3}$ = 53.4 Hz, $^3J_{F,4}$ = 21.0 Hz); open chain form: ^{1}H NMR (700 MHz, D_2O) δ = 5.22 (dd, $^2J_{3,F}$ = 47.7 Hz, $^3J_{3,4}$ = 3.0 Hz, 1H, H-3), 4.65 (dd, $^2J_{1a,1b}$ = 19.8 Hz, $^4J_{1a,F}$ = 1.4 Hz, 1H, H-1a), 4.61 (dd, $^2J_{1b,1a}$ = 19.8 Hz, $^4J_{1b,F}$ = 1.0 Hz, 1H, H-1b), 4.22 (dtd, $^3J_{4,F}$ = 23.4 Hz, $^3J_{4,5a}$ = $^3J_{4,5b}$ = 6.5 Hz, $^3J_{4,3}$ = 3.0 Hz, 1H, H-4), 3.71 (d, $^2J_{5a,5b}$ = 10.6 Hz, $^3J_{5a,4}$ = 6.5 Hz, 1H, H-5a), 3.69 (d, $^2J_{5a,5b}$ = 10.6 Hz, $^3J_{5b,4}$ = 6.5 Hz, 1H, H-5b); ^{13}C NMR (176 MHz, D_2O)

δ = 211.33 (d, $^{2}J_{2,F}$ = 23.5 Hz, C-2), 98.18 (d, $^{1}J_{3,F}$ = 184.1 Hz, C-3), 74.43 (d, $^{2}J_{4,F}$ = 20.5 Hz, C-4), 68.82 (d, $^{3}J_{1,F}$ = 4.3 Hz, C-1), 62.72 (d, $^{3}J_{5,F}$ = 7.7 Hz, C-5); ^{19}F NMR (659 MHz, D_2O) δ = −210.13 (dd, $^{2}J_{F,3}$ = 47.7 Hz, $^{3}J_{F,4}$ = 23.4 Hz); HRMS (ESI+): m/z calc. for $C_5H_9FO_4Na^+$ $[M+Na]^+$: 175.0377, found: 175.0375; m/z calc. for $C_{10}H_{16}F_2O_7Na^+$ $[M_2-H_2O+Na]^+$: 309.0756, found: 309.0370; m/z calc. for $C_{10}H_{16}F_2O_8Na^+$ $[M_2-2H+Na]^+$: 325.0706, found: 325.0686.

D-*Threono*-1,4-lactone (28). D-Xylose (**27**, 30 g, 199.8 mmol, 1 equiv.) was dissolved in water (1 L) and cooled to 0°C. A cooled aq. KOH-solution (0.6 M, 1 L, 600.0 mmol, 3 equiv.) was added and the flask was evacuated at 0°C for 3 min. The flask was vented with an O_2-balloon, the mixture was warmed to room temperature and stirred under an oxygen atmosphere for 16 h. The solution was passed through Dowex 50 (H^+ form, 750 mL) and the filtrate was evaporated. The oily residue was dissolved in acetonitrile (333 mL) and *para*-toluenesulfonic acid monohydrate (253 mg, 1.3 mmol, 0.02 equiv.) was added. The mixture was refluxed for 5 h, cooled to room temperature, filtered and evaporated. The remaining solid crude residue was recrystallized (EtOAc) to give D-*threono*-1,4-lactone (**28**) as a colorless solid (12.51 g, 53%): R_f = 0.46 (EtOAc, $KMnO_4$); $[\alpha]_D^{20}$ = −32.1 (*c* 1.0, H_2O); ^{1}H NMR (600 MHz, DMSO-d_6) δ = 6.12 (d, $^{3}J_{OH,2}$ = 6.5 Hz, 1H, OH-2), 5.77 (d, $^{3}J_{OH,3}$ = 4.8 Hz, 1H, OH-3), 4.33 (dd, $^{2}J_{4a,4b}$ = 8.8 Hz, $^{3}J_{4a,3}$ = 6.5 Hz, 1H, H-4a), 4.14 (qd, $^{3}J_{3,2}$ = $^{3}J_{3,4a}$ = $^{3}J_{3,4b}$ = 6.5 Hz, $^{3}J_{3,OH}$ = 4.8 Hz, 1H, H-3), 4.07 (t, $^{3}J_{2,3}$ = $^{3}J_{2,OH}$ = 6.5 Hz, 1H, H-2), 3.84 (dd, $^{2}J_{4b,4a}$ = 8.8 Hz, $^{3}J_{4b,3}$ = 6.5 Hz, 1H, H-4b); ^{13}C NMR (151 MHz, d_6-DMSO) δ = 175.63 (C-1), 73.02 (C-2), 72.21 (C-3), 69.80 (C-4); HRMS (ESI+): m/z calc. for $C_4H_6O_4Na^+$ $[M+Na]^+$: 141.0158, found: 141.0158, m/z calc. for $C_5H_{10}O_5Na^+$ $[M+MeOH+Na]^+$: 173.0420, found: 173.0420.

2-*O*-Triisopropylsilyl-D-*threono*-1,4-lactone (29). D-*Threono*-1,4-lactone (**28**, 8.936 mg, 75. mmol, 1 equiv.) was dissolved in dry acetonitrile (204 mL) under argon atmosphere and imidazole (12.364 g, 181.6 mmol, 2.4 equiv.) was added. The solution was cooled to 0°C and TIPSCl (17.5 g, 90.8 mmol, 1.2 equiv.) was added dropwise. The reaction mixture was warmed to room temperature and stirred for 16 h. All volatiles were removed under reduced pressure and the residue was partitioned between EtOAc (150 mL) and water (150 mL). The layers were separated, and the aqueous layer extracted with EtOAc (2 × 75 mL). The combined organic layers were washed with brine (100 mL), dried ($MgSO_4$), filtered and concentrated. The crude residue was purified by MPLC (100 g silica gel, 6-50% EtOAc in *n*-heptane) to yield 2-*O*-triisopropylsilyl-D-*threono*-1,4-lactone (**29**) as a colorless oil (3.9 g, 22%): R_f = 0.19 (*n*hept/EtOAc 3:1, $KMnO_4$); $[\alpha]_D^{20}$ = −39.8 (*c* 1.0, $CHCl_3$); ^{1}H NMR (600 MHz, $CDCl_3$) δ = 4.48 (dd, $^{2}J_{4a,4b}$ = 9.0, $^{3}J_{4a,3}$ = 6.0 Hz, 1H, H-4a), 4.44 (qd, $^{3}J_{3,2}$ = $^{3}J_{3,4a}$ = $^{3}J_{3,4b}$ = 6.0, $^{3}J_{3,OH}$ = 4.2 Hz, 1H, H-3), 4.36 (d, $^{3}J_{2,3}$ = 6.0 Hz, 1H, H-2), 4.02 (dd, $^{2}J_{4b,4a}$ = 9.0 Hz, $^{3}J_{4b,3}$ = 6.0 Hz, 1H, H-4b), 2.09 (d, $^{3}J_{OH,3}$ = 4.2 Hz, 1H, OH), 1.23-1.15 (m, 3H, CH^{TIPS}), 1.10 (d, $^{3}J_{H,H}$ = 7.0 Hz, 18H, CH_3^{TIPS}); ^{13}C NMR (151 MHz, $CDCl_3$) δ = 173.72 (C-1), 74.99 (C-2), 74.59 (C-3), 70.08 (C-4), 17.95 (3 CH_3^{TIPS}), 17.91 (3 CH_3^{TIPS}), 12.34 (3 CH^{TIPS}); HRMS (ESI+): m/z calc. for $C_{13}H_{26}O_4SiNa^+$ $[M+Na]^+$: 297.1493, found: 297.1492.

3-*O*-Benzoyl-2-*O*-triisopropylsilyl-D-*threono*-1,4-lactone (30). 2-*O*-Triisopropylsilyl-D-*threono*-1,4-lactone (29, 3.9 g, 14.2 mmol, 1 equiv.) was dissolved in dry pyridine (28.4 mL) under argon atmosphere and cooled to 0°C. 4-(Dimethylamino)pyridine (87 mg, 0.7 mmol,

0.05 equiv.) was added, followed by the dropwise addition of benzoyl chloride (3.7 mL, 4.495 g, 32.0 mmol, 2.25 equiv.). The mixture was stirred at room temperature for 2 h before addition of water (10 mL). The mixture was extracted with EtOAc (2 × 20 mL). The combined organic layers were washed with water (15 mL) and brine (15 mL), dried ($MgSO_4$), filtered and evaporated. The crude residue was purified my MPLC (100 g silica gel, 1-9% EtOAc in *n*-heptane) to give 3-*O*-benzoyl-2-*O*-triisopropylsilyl-D-*threono*-1,4-lactone (**30**) as a colorless oil (4.9 g, 91%): R_f = 0.22 (*n*hept/EtOAc 9:1, UV light and CAM); $[\alpha]_D^{20}$ = −102.5 (*c* 1.0, $CHCl_3$); ^{1}H NMR (700 MHz, $CDCl_3$) δ = 8.02 (d, $^3J_{H,H}$ = 7.4 Hz, 2H, H^{Ar}), 7.62 (t, $^3J_{H,H}$ = 7.4 Hz, 1H, H^{Ar}), 7.47 (t, $^3J_{H,H}$ = 7.4 Hz, 2H, H^{Ar}), 5.43 (dt, $^3J_{3,4a}$ = 5.4 Hz, $^3J_{3,2}$ = $^3J_{3,4b}$ = 4.2 Hz, 1H, H-3), 4.81 (dd, $^2J_{4a,4b}$ = 10.3 Hz, $^3J_{4a,3}$ = 5.4 Hz, 1H, H-4a), 4.62 (d, $^3J_{2,3}$ = 4.2 Hz, 1H, H-2), 4.26 (dd, $^2J_{4b,4a}$ = 10.3 Hz, $^3J_{4b,3}$ = 4.2 Hz, 1H, H-4b), 1.24-1.17 (m, 3H, CH^{TIPS}), 1.11 (d, $^3J_{H,H}$ = 7.3 Hz, 9H, CH_3^{TIPS}), 1.10 (d, $^3J_{H,H}$ = 7.3 Hz, 9H, CH_3^{TIPS}); ^{13}C NMR (176 MHz, $CDCl_3$) δ = 173.01 (C-1), 165.76 (C^{Bz}), 134.00 (CH^{Ar}), 129.89 (2 CH^{Ar} & C^{Ar}), 128.77 (2 CH^{Ar}), 76.37 (C-3), 72.02 (C-2), 69.81 (C-4), 17.89 (3 CH_3^{TIPS}), 17.85 (3 CH_3^{TIPS}), 12.28 (3 CH^{TIPS}); HRMS (ESI+): m/z calc. for $C_{20}H_{30}O_5SiNa^+$ $[M+Na]^+$: 401.1755, found: 401.1745.

2,5-Anhydro-4-*O*-benzoyl-1-deoxy-3-*O*-triisopropylsilyl-D-*threo*-pent-1-enitol (31). This reaction is light sensitive and was carried out in the dark. Bis(cyclopentadienyl)dimethyltitanium (**49**, 0.5 M in dry toluene, 12.5 mL, 6.3 mmol, 2.3 equiv.) was added to 3-*O*-benzoyl-2-*O*-triisopropylsilyl-D-*threono*-1,4-lactone (**30**, 1.031 g, 2.7 mmol, 1 equiv.) under argon atmosphere in the dark. The flask was equipped with a reflux condenser and the mixture was heated to 70°C for 16h. The reaction mixture was cooled to room temperature, diluted with *n*-heptane (50 mL) and stirring was continued for 30 min. The orange suspension was filtered through Celite® and the filtrate was concentrated. The crude residue was purified by MPLC (100 g silica gel, 1-5% EtOAc in *n*-heptane) to give 2,5-anhydro-4-*O*-benzoyl-1-deoxy-3-*O*-triisopropylsilyl-D-*threo*-pent-1-enitol (**31**) as a yellow oil (700 mg, 68%) which was immediately used in the next step: R_f = 0.37 (*n*hept/EtOAc 9:1, UV light and CAM); $[\alpha]_D^{20}$ = −61.1 (*c* 1.0, $CHCl_3$); ^{1}H NMR (700 MHz, $CDCl_3$) δ = 8.02 (d, $^3J_{H,H}$ = 7.4 Hz, 2H, H^{Ar}), 7.58 (t, $^3J_{H,H}$ = 7.4 Hz, 1H, H^{Ar}), 7.44 (t, $^3J_{H,H}$ = 7.4 Hz, 2H, H^{Ar}), 5.28-5.27 (m, 1H, H-4), 4.70 (d, $^3J_{3,4}$ = 1.3 Hz, 1H, H-3), 4.54 (dd, $^2J_{5a,5b}$ = 10.4 Hz, $^3J_{5a,4}$ = 3.7 Hz, 1H, H-5a), 4.49 (d, $^2J_{1a,1b}$ = 1.8 Hz, 1H, H-1a), 4.22 (d, $^2J_{1b,1a}$ = 1.8 Hz, 1H, H-1b), 4.21 (d, $^2J_{5b,5a}$ = 10.4 Hz, 1H, H-5b), 1.20-1.14 (m, 3H, CH^{TIPS}), 1.10 (d, $^3J_{H,H}$ = 7.1 Hz, 18H, CH_3^{TIPS}); ^{13}C NMR (176 MHz, $CDCl_3$) δ = 165.87 (C^{Bz}), 162.66 (C-2), 133.55 (CH^{Ar}), 129.95 (2 CH^{Ar}), 129.59 (C^{Ar}), 128.60 (2 CH^{Ar}), 85.01 (C-1), 78.61 (C-4), 74.97 (C-3), 72.93 (C-5), 18.13 (6 CH_3^{TIPS}), 12.47 (3 CH^{TIPS}); HRMS (ESI+): m/z calc. for $C_{21}H_{32}O_4SiNa^+$ $[M+Na]^+$: 399.1962, found: 399.1962, m/z calc. for $C_{21}H_{34}O_5SiNa^+$ $[M+H_2O+Na]^+$: 417.2068, found: 417.2083.

4-*O*-Benzoyl-3-*O*-triisopropylsilyl-D-xylulose (32). 2,5-Anhydro-4-*O*-benzoyl-1-deoxy-3-*O*-triisopropylsilyl-D-*threo*-pent-1-enitol (**31**, 700 mg, 1.9 mmol, 1 equiv.) was dissolved in a 4:1 mixture of acetone and water (37.2 mL) and NMO (436 mg, 3.7 mmol, 2 equiv.) and potassium osmate dihydrate (34 mg, 0.1 mmol, 0.05 equiv.) were added. The reaction mixture was stirred at room temperature for 16 h. The mixture was diluted with EtOAc (15 mL) and washed with water (20 mL) and brine (20 mL). The organic layer was dried ($MgSO_4$), filtered and concentrated. The crude residue was purified by MPLC (100 g silica gel, 6-42% EtOAc

in *n*-heptane) to give 4-*O*-benzoyl-3-*O*-triisopropylsilyl-D-xylulose (**32**) as a mixture of anomers as a colorless oil (642 mg, 84%): R_f = 0.2 (*n*hept/EtOAc 3:1, UV light and CAM); $[\alpha]_D^{20}$ = −47.1 (*c* 1.0, $CHCl_3$); ^{1}H NMR (700 MHz, $CDCl_3$) δ = 8.02 (d, $^3J_{H,H}$ = 7.4 Hz, 2H, H^{Ar}), 7.60 (t, $^3J_{H,H}$ = 7.4 Hz, 1H, H^{Ar}), 7.46 (t, $^3J_{H,H}$ = 7.4 Hz, 2H, H^{Ar}), 5.31 (d, $^3J_{4,5a}$ = 3.7 Hz, 1H, H-4), 4.61 (broad s, 2H, H-3 & OH-2), 4.33 (dd, $^2J_{5a,5b}$ = 10.8 Hz, , $^3J_{5a,4}$ = 3.7 Hz, 1H, H-5a), 3.95 (d, $^2J_{5b,5a}$ = 10.8 Hz, 1H, H-5b), 3.74 (dd, $^2J_{1a,1b}$ = 11.8 Hz, $^3J_{1a,OH}$ = 7.0 Hz, 1H, H-1a), 3.70 (dd, $^2J_{1b,1a}$ = 11.8 Hz, $^3J_{1b,OH}$ = 7.0 Hz, 1H, H-1b), 1.93 (t, $^3J_{OH,1a}$ = $^3J_{OH,1b}$ = 7.0 Hz, 1H, OH-1), 1.28-1.21 (m, 3H, CH^{TIPS}), 1.11 (d, $^3J_{H,H}$ = 7.4 Hz, 9H, CH_3^{TIPS}), 1.11 (d, $^3J_{H,H}$ = 7.5 Hz, 9H, CH_3^{TIPS}); ^{13}C NMR (176 MHz, $CDCl_3$) δ = 165.77 (C^{Bz}), 133.69 (CH^{Ar}), 129.86 (2 CH^{Ar}), 129.41 (C^{Ar}), 128.74 (2 CH^{Ar}), 104.16 (C-2), 80.51 (C-4), 76.53 (C-3), 70.10 (C-5), 65.00 (C-1), 17.97 (3 CH_3^{TIPS}), 17.92 (3 CH_3^{TIPS}), 12.20 (3 CH^{TIPS}); HRMS (ESI+): m/z calc. for $C_{21}H_{34}O_6SiNa^+$ $[M+Na]^+$: 433.2017, found: 433.2015.

4-*O*-Benzoyl-1,2-*O*-isopropylidene-3-*O*-triisopropylsilyl-D-xylulose (33). 4-*O*-Benzoyl-3-*O*-triisopropylsilyl-D-xylulose (**32**, 348 mg, 0.85 mmol, 1 equiv.) was dissolved in 2,2-dimethoxypropane (519 µl, 441 mg, 4.2 mmol, 5 equiv.) and $SnCl_2$ (3 mg, 0.02 mmol, 0.02 equiv.), dissolved in 1,2-dimethoxyethane (170 µl) was added. The mixture was heated to 60°C for 2 h, cooled to room temperature, and the solvent was evaporated under reduced pressure. The crude residue was purified by MPLC (50 g silica gel, 2-20% EtOAc in *n*-heptane) to give 4-*O*-benzoyl-1,2-*O*-isopropylidene-3-*O*-triisopropylsilyl-D-xylulose (**33**) as amixture of anomers as a colorless oil (255 mg, 67%): R_f = 0.45 (*n*hept/EtOAc 9:1, UV light and CAM); $[\alpha]_D^{20}$ = −48.0 (*c* 1.0, $CHCl_3$); <u>major anomer</u>: ^{1}H NMR (700 MHz, $CDCl_3$) δ = 8.06 (d, $^3J_{H,H}$ = 7.8 Hz, 2H, H^{Ar}), 7.59 (t, $^3J_{H,H}$ = 7.8 Hz, 1H, H^{Ar}), 7.46 (t, $^3J_{H,H}$ = 7.8 Hz, 2H, H^{Ar}), 5.20 (ddd, $^3J_{4,5a}$ = 6.0 Hz, $^3J_{4,5b}$ = 4.1, $^3J_{4,3}$ = 2.5 Hz, 1H, H-4), 4.56 (d, $^3J_{3,4}$ = 2.4 Hz, 1H, H-3), 4.41 (dd, $^2J_{5a,5b}$ = 10.1 Hz, $^3J_{5a,4}$ = 6.0 Hz, 1H, H-5a), 4.37 (d, $^2J_{1a,1b}$ = 9.4 Hz, 1H, H-1a), 4.04 (d, $^2J_{1b,1a}$ = 9.4 Hz, 1H, H-1b), 3.92 (dd, $^2J_{5b,5a}$ = 10.1 Hz, $^3J_{5b,4}$ = 4.1 Hz, 1H, H-5b), 1.51 (s, 3H, CH_3^{ipr}), 1.42 (s, 3H, CH_3^{ipr}), 1.19-1.13 (m, 3H, CH^{TIPS}), 1.08 (d, $^3J_{H,H}$ = 6.3 Hz, 9H, CH_3^{TIPS}), 1.08 (d, $^3J_{H,H}$ = 7.2 Hz, 9H, CH_3^{TIPS}); ^{13}C NMR (176 MHz, $CDCl_3$) δ = 166.18 (C^{Bz}), 133.44 (CH^{Ar}), 129.97 (2 CH^{Ar}), 129.79 (C^{Ar}), 128.57 (2 CH^{Ar}), 113.52 (C-2), 111.37 (C^{ipr}), 80.63 (C-3), 79.53 (C-4), 70.12 (C-5), 69.63 (C-1), 26.71 (CH_3^{ipr}), 26.47 (CH_3^{ipr}), 18.11 (3 CH_3^{TIPS}), 18.03 (3 CH_3^{TIPS}), 12.46 (3 CH_3^{TIPS}); <u>minor anomer</u>: ^{1}H NMR (700 MHz, $CDCl_3$) δ 8.03 (d, $^3J_{H,H}$ = 7.8 Hz, 2H, H^{Ar}), 7.59 (t, $^3J_{H,H}$ = 7.8 Hz, 1H, H^{Ar}), 7.46 (t, $^3J_{H,H}$ = 7.8 Hz, 2H, H^{Ar}), 5.34 (ddd, $^3J_{4,5a}$ = 6.4 Hz, $^3J_{4,3}$ = 5.1 Hz, $^3J_{4,5b}$ = 3.4 Hz, 1H, H-4), 4.51 (d, $^3J_{3,4}$ = 5.1 Hz, 1H, H-3), 4.50 (dd, $^2J_{5a,5b}$ = 10.5 Hz, $^3J_{5a,4}$ = 6.4 Hz, 1H, H-5a), 4.13 (d, $^2J_{1a,1b}$ = 8.8 Hz, 1H, H-1a), 4.11 (d, $^2J_{1b,1a}$ = 8.8 Hz, 1H, H-1b), 3.76 (dd, $^2J_{5b,5a}$ = 10.5 Hz, $^3J_{5b,4}$ = 3.4 Hz, 1H, H-5b), 1.53 (s, 3H, CH_3^{ipr}), 1.46 (s, 3H, CH_3^{ipr}), 1.20-1.13 (m, 3H, CH^{TIPS}), 1.08 (d, $^3J_{H,H}$ = 6.3 Hz, 9H, CH_3^{TIPS}), 1.08 (d, $^3J_{H,H}$ = 7.2 Hz, 9H, CH_3^{TIPS}); ^{13}C NMR (176 MHz, $CDCl_3$) δ = 166.39 (C^{Bz}), 133.55 (CH^{Ar}), 129.75 (2 CH^{Ar}), 129.50 (C^{Ar}), 128.65 (2 CH^{Ar}), 111.82 (C^{ipr}), 110.07 (C-2), 80.02 (C-4), 76.15 (C-3), 70.85 (C-1), 69.88 (C-5), 26.93 (CH_3^{ipr}), 26.48 (CH_3^{ipr}), 18.17 (3 CH_3^{TIPS}), 18.08 (3 CH_3^{TIPS}), 12.69 (3 CH^{TIPS}); HRMS (ESI+): m/z calc. for $C_{24}H_{38}O_6SiNa^+$ $[M+Na]^+$: 473.2330, found: 473.2327.

4-*O*-Benzoyl-1,2-*O*-isopropylidene-D-xylulose (34). 4-*O*-Benzoyl-1,2-*O*-isopropylidene-3-*O*-triisopropylsilyl-D-xylulose (**33**, 328 mg, 0.7 mmol, 1 equiv.) was dissolved in dry THF (3.64

mL) under argon atmosphere and cooled to 0°C. Tetrabutylammonium fluoride (1M in THF, 1.16 mL, 1.2 mmol, 1.6 equiv.) was added dropwise and the mixture was stirred for 0.5 h at 0°C before dropwise addition of a sat. aq. $NaHCO_3$-solution (7.7 mL). The mixture was extracted with CH_2Cl_2 (3 × 5 mL), the combined organic layers were dried ($MgSO_4$), filtered and evaporated. The crude residue was purified by MPLC (25 g silica gel, 6-43% EtOAc in *n*-heptane) to give 4-*O*-benzoyl-1,2-*O*-isopropylidene-D-xylulose (**34**) as a mixture of anomers as a colorless oil (141 mg, 66%): R_f = 0.17 (*n*hept/EtOAc 3:1, UV light and CAM); $[\alpha]_D^{20}$ = +7.9 (*c* 1.0, $CHCl_3$); <u>major anomer</u>: ^{1}H NMR (700 MHz, $CDCl_3$) δ = 8.05 (d, $^3J_{H,H}$ = 7.8 Hz, 2H, H^{Ar}), 7.60 (t, $^3J_{H,H}$ = 7.8 Hz, 1H, H^{Ar}), 7.46 (t, $^3J_{H,H}$ = 7.8 Hz, 2H, H^{Ar}), 5.10 (ddd, $^3J_{4,5a}$ = 6.9, $^3J_{4,5b}$ = 6.2, $^3J_{4,3}$ = 3.0 Hz, 1H, H-4), 4.39 (dd, $^2J_{5a,5b}$ = 9.8 Hz, $^3J_{5a,4}$ = 6.9 Hz, 1H, H-5a), 4.37 (d, $^2J_{1a,1b}$ = 9.6 Hz, 1H, H-1a), 4.35 (t, $^3J_{3,4}$ = $^3J_{3,OH}$ = 3.0 Hz, 1H, H-3), 4.06 (d, $^2J_{1b,1a}$ = 9.6 Hz, 1H, H-1b), 4.05 (dd, $^2J_{5b,5a}$ = 9.8 Hz, $^3J_{5b,4}$ = 6.1 Hz, 1H, H-5b), 2.95 (d, $^3J_{OH,3}$ = 3.0 Hz, 1H, OH), 1.51 (s, 3H, $CH_3{}^{ipr}$), 1.40 (s, 3H, $CH_3{}^{ipr}$); ^{13}C NMR (176 MHz, $CDCl_3$) δ = 167.45 (C^{Bz}), 133.74 (CH^{Ar}), 130.01 (2 CH^{Ar}), 129.35 (C^{Ar}), 128.66 (2 CH^{Ar}), 112.82 (C-2), 111.47 (C^{ipr}), 80.77 (C-3), 80.72 (C-4), 69.24 (C-1), 68.32 (C-5), 26.56 ($CH_3{}^{ipr}$), 26.55 ($CH_3{}^{isopr}$); <u>minor anomer</u>: ^{1}H NMR (700 MHz, $CDCl_3$) δ = 8.04 (d, $^3J_{H,H}$ = 7.9 Hz, 2H, H^{Ar}), 7.59 (t, $^3J_{H,H}$ = 7.9 Hz, 1H, H^{Ar}), 7.46 (t, $^3J_{H,H}$ = 7.9 Hz, 2H, H^{Ar}), 5.30 (ddd, $^3J_{4,5a}$ = 6.3 Hz, $^3J_{4,3}$ = 4.5 Hz, $^3J_{4,5b}$ =3.4 Hz, 1H, H-4), 4.42 (dd, $^2J_{5a,5b}$ = 10.5 Hz, $^3J_{5a,4}$ = 6.3 Hz, 1H, H-5a), 4.26 (dd, $^3J_{3,OH}$ = 8.0 Hz, $^3J_{3,4}$ = 4.5 Hz, 1H, H-3), 4.18 (d, $^2J_{1a,1b}$ = 9.2 Hz, 1H, H-1a), 4.14 (d, $^2J_{1b,1a}$ = 9.2 Hz, 1H, H-1b), 3.89 (dd, $^2J_{5b,5a}$ = 10.5 Hz, $^3J_{5b,4}$ = 3.4 Hz, 1H, H-5b), 2.73 (d, $^3J_{OH,3}$ = 8.0 Hz, 1H, OH), 1.55 (s, 3H, $CH_3{}^{ipr}$), 1.47 (s, 3H, $CH_3{}^{ipr}$); ^{13}C NMR (176 MHz, $CDCl_3$) δ = 166.62 (C^{Bz}), 133.62 (CH^{Ar}), 129.90 (2 CH^{Ar}), 129.44 (C^{Ar}), 128.64 (2 CH^{Ar}), 111.95 (C^{ipr}), 109.88 (C-2), 79.75 (C-4), 76.77 (C-3), 70.90 (C-1), 69.85 (C-5), 26.51 ($CH_3{}^{ipr}$), 26.41 ($CH_3{}^{ipr}$); HRMS (ESI+): m/z calc. for $C_{15}H_{18}O_6Na^+$ $[M+Na]^+$: 317.0996, found: 317.1002.

4-*O*-Benzoyl-1,2-*O*-isopropylidene-3-*O*-trifluoromethanesulfonyl-D-xylulose (35). 4-*O*-Benzoyl-1,2-*O*-isopropylidene-D-xylulose (**34**, 39 mg, 0.1 mmol, 1 equiv.) was dissolved in dry CH_2Cl_2 (660 µL) under argon atmosphere. The solution was cooled to 0°C and dry pyridine (89 mg, 91 µl, 1.1 mmol, 8.5 equiv.), followed by trifluoromethanesulfonic anhydride (75 mg, 45 µl, 0.3 mmol, 2 equiv.) were added dropwise. The reaction mixture was stirred at 0°C for 1 h before addition of MeOH (90 µL) and partitioning of the mixture between CH_2Cl_2 (2 mL) and water (2 mL). The layers were separated, the aqueous layer was extracted with CH_2Cl_2 (2 × 5 mL), the combined organic layers washed with ice-cold water (2 × 3 mL), an ice-cold sat. aq. $NaHCO_3$-solution (3 mL) and finally ice-cold brine (3 mL). The organic layer was dried ($MgSO_4$), filtered and the solvent evaporated to give 4-*O*-benzoyl-1,2-*O*-isopropylidene-3-*O*-trifluoromethanesulfonyl-D-xylulose (**35**) as a 3 : 1 mixture of anomers as a yellow oil (48 mg, 85%): R_f = 0.42 (*n*hept/EtOAc 3:1, UV light and CAM); $[\alpha]_D^{20}$ = −55.4 (*c* 1.0, $CHCl_3$); <u>major anomer</u>: ^{1}H NMR (700 MHz, $CDCl_3$) δ = 8.06 (d, $^3J_{H,H}$ = 7.9 Hz, 2H, H^{Ar}), 7.62 (t, $^3J_{H,H}$ = 7.9 Hz, 1H, H^{Ar}), 7.48 (t, $^3J_{H,H}$ = 7.9 Hz, 2H, H^{Ar}), 5.51 (ddd, $^3J_{4,5a}$ = 7.0 Hz, $^3J_{4,5b}$ = 5.4 Hz, $^3J_{4,3}$ = 2.2 Hz, 1H, H-4), 5.41 (d, $^3J_{3,4}$ = 2.2 Hz, 1H, H-3), 4.56 (dd, $^2J_{5a,5b}$ = 10.1 Hz, $^3J_{5a,4}$ = 7.0 Hz, 1H, H-5a), 4.27 (d, $^2J_{1a,1b}$ = 10.0 Hz, 1H, H-1a), 4.18 (d, $^2J_{1b,1a}$ = 10.0 Hz, 1H, H-1b), 3.97 (dd, $^2J_{5b,5a}$ = 10.1 Hz, , $^3J_{5b,4}$ = 5.4 Hz, 1H, H-5b), 1.52 (s, 3H, $CH_3{}^{isopr}$), 1.46 (s, 3H, $CH_3{}^{isopr}$); ^{13}C NMR (176 MHz, $CDCl_3$) δ = 165.54 (C^{Bz}), 134.00 (CH^{Ar}), 130.11 (2 CH^{Ar}), 128.76 (2 CH^{Ar}), 128.72 (C^{Ar}), 113.15 (C^{isopr}), 110.35 (C-2), 91.37 (C-3), 76.03 (C-4),

69.75 (C-5), 69.38 (C-1), 26.31 (CH_3^{isopr}), 26.26 (CH_3^{isopr}); ^{19}F NMR (659 MHz, $CDCl_3$) δ = −74.79 (s); minor anomer: ^{1}H NMR (700 MHz, $CDCl_3$) δ = 8.03 (d, $^3J_{H,H}$ = 7.9 Hz, 2H, H^{Ar}), 7.62 (t, $^3J_{H,H}$ = 7.9 Hz, 1H, H^{Ar}), 7.48 (t, $^3J_{H,H}$ = 7.9 Hz, 2H, H^{Ar}), 5.66 (ddd, $^3J_{4,5a}$ = 6.7 Hz, $^3J_{4,3}$ = 5.2 Hz, $^3J_{4,5b}$ = 3.7 Hz, 1H, H-4), 5.27 (d, $^3J_{3,4}$ = 5.2 Hz, 1H, H-3), 4.54 (dd, $^2J_{5a,5b}$ = 10.3 Hz, $^3J_{5a,4}$ = 6.7 Hz, 1H, H-5a), 4.28 (d, $^2J_{1a,1b}$ = 9.7 Hz, 1H, H-1a), 4.20 (d, $^2J_{1b,1a}$ = 9.7 Hz, 1H, H-1b), 3.91 (dd, $^2J_{5b,5a}$ = 10.3 Hz, $^3J_{5b,4}$ = 3.7 Hz, 1H, H-5b), 1.54 (s, 3H, CH_3^{isopr}), 1.49 (s, 3H, CH_3^{isopr}); ^{13}C NMR (176 MHz, $CDCl_3$) δ = 165.87 (C^{Bz}), 134.05 (CH^{Ar}), 129.94 (2 CH^{Ar}), 128.83 (2 CH^{Ar}), 128.60 (C^{Ar}), 118.57 (q, $^1J_{C,F}$ = 319.8 Hz, C^{Tf}), 113.11 (C^{isopr}), 107.72 (C-2), 85.78 (C-3), 75.82 (C-4), 70.14 (C-1), 69.42 (C-5), 26.19 (CH_3^{isopr}), 25.97 (CH_3^{isopr}). ^{19}F NMR (659 MHz, $CDCl_3$) δ = −75.07 (s); HRMS (ESI+): m/z calc. for $C_{16}H_{17}F_3O_8SNa^+$ $[M+Na]^+$: 449.0488, found: 449.0491.

Synthesis of 3-deoxy-3-fluoro-d-xylulose (3DFXu, 5) and its potential radiolabelling precursor 48

2-*O-para*-Toluenesulfonyl-D-*erythrono*-1,4-lactone (36). *para*-Toluenesulfonyl chloride (8.55 g, 44.5 mmol, 1.05 equiv.) was added to a solution of D-*erythrono*-1,4-lactone (**10**, 5 g, 42.0 mmol, 1 equiv.) in pyridine (21 mL) at 0 °C. After stirring for 18 h, pyridine was removed *in vacuo* at 15 °C. The residue was taken up in EtOAc (100 mL), washed with aq. HCl (1 M, 3 × 50 mL) and with brine (80 mL). The organic layer was dried ($MgSO_4$), filtered and concentrated under reduced pressure to give a viscous residue. 2-*O-para*-Toluenesulfonyl-D-*erythrono*-1,4-lactone (**36**) was precipitated by addition of CH_2Cl_2 as a colorless solid (8.233 g, 72%): $[\alpha]_D^{20}$ = −38.6 (*c* 1.0, acetone), {lit.[35] $[\alpha]_D^{20}$ = −45 (*c* 1.0, acetone)}; ^{1}H NMR ($CDCl_3$, 700 MHz): δ 7.90 (d, *J* = 8.3 Hz, 2H, H^{Ar}), 7.40 (d, *J* = 8.1 Hz, 2H, H^{Ar}), 5.06 (d, $^3J_{2,3}$ = 4.6 Hz, 1H, H-2), 4.77 (ddd, $^3J_{3,2}$ = 4.6 Hz, $^3J_{3,4b}$ = 3.3 Hz, $^3J_{3,OH}$ = 2.1 Hz, 1H, H-3), 4.44 (d, $^2J_{4a,4b}$ = 10.7 Hz, 1H, H-4a), 4.38 (ddd, $^2J_{4b,4a}$ = 10.7 Hz, $^4J_{4b,3}$ = 3.3 Hz, $^3J_{4b,OH}$ = 2.1 Hz, 1H, H-4b), 2.64 (dd, $^3J_{OH,3}$ = 2.1 Hz, d, $^4J_{OH,4b}$ = 2.1 Hz, 1H, OH), 2.48 (s, CH_3^{Tos}); ^{13}C NMR ($CDCl_3$, 176 MHz): δ = 168.47 (C-1), 146.33 (C^{Ar}), 131.52 (C^{Ar}), 130.16 (2 CH^{Ar}), 128.39 (2 CH^{Ar}), 74.25 (C-2), 71.13 (C-4), 68.09 (C-3), 21.79 (CH_3^{Tos}); HRMS (ESI+): m/z calc. for $C_{11}H_{13}O_6SH^+$ $[M+H]^+$: 273.0428, found: 273.0434.

2,3-Anhydro-D-*erythrono*-1,4-lactone (37). K_2CO_3 (3.989 g, 28.9 mmol, 3.93 equiv.) was added to a solution of 2-*O-para*-toluenesulfonyl-D-*erythrono*-1,4-lactone (**36**, 2 g, 7.3 mmol, 1 equiv.) in acetonitrile (30 mL). After stirring for 18 h at room temperature, the orange suspension was filtered over Celite® and the filtrate was concentrated *in vacuo.* The remaining residue was purified by bulb-to-bulb distillation (90°C oven temp./8 mm Hg) to give 2,3-anhydro-D-*erythrono*-1,4-lactone (**37**) as a colorless oil (492 mg, 67%): $[\alpha]_D^{20}$ = +21.7(*c* 1.0, $CHCl_3$), {lit.[35] $[\alpha]_D^{20}$ = +28 (*c* 1.0, $CHCl_3$)}; ^{1}H NMR ($CDCl_3$, 600 MHz): δ = 4.48 (d, $^2J_{4a,4b}$ = 11.4 Hz, 1H, H-4a), 4.31 (dd, $^2J_{4b,4a}$ = 11.4 Hz, $^3J_{4b,3}$ = 1.4 Hz, 1H, H-4b), 4.20 (dd, $^3J_{3,2}$ = 2.5 Hz, $^3J_{3,4b}$ = 1.4 Hz, 1H, H-3), 3.80 (d, $^3J_{2,3}$ = 2.5 Hz, 1H, H-2); ^{13}C NMR ($CDCl_3$, 151 MHz) δ = 170.46 (C-1), 68.20 (C-4), 54.94 (C-3), 49.56 (C-2); HRMS (ESI+): m/z calc. for $C_4H_4O_3H^+$ $[M+H]^+$: 101.0233, found: 101.0231.

2-Deoxy-2-fluoro-D-*threono*-1,4-lactone (38).[26] 2,3-Anhydro-D-*erythrono*-1,4-lactone (**37**, 682 mg, 6.8 mmol, 1 equiv.) and triethylamine trihydrofluoride (14.8 g, 90.6 mmol, 13.3 equiv.)

were heated at 70°C in a PTFE flask for 6 days. The pH of the mixture was adjusted to 5 by addition of a sat. aq. $NaHCO_3$-solution (200 mL). After filtration over Celite®, the filtrate was extracted with EtOAc (3 × 200 mL) and the combined organic layers were dried. The remaining oily residue was purified by bulb-to-bulb destillation (110°C oven temp./1 mm Hg) to give 2-deoxy-2-fluoro-D-*threono*-1,4-lactone **38**) as a colorless oil (474 mg, 58%) $[\alpha]_D^{20}$ = −7.2 (*c* 1.0, $CHCl_3$); ^{1}H NMR ($CDCl_3$, 700 MHz): δ = 5.11 (dd, $^2J_{2,F}$ = 51.0 Hz, $^3J_{2,3}$ = 6.9 Hz, 1H, H-2), 4.80 (ddddd, $^3J_{3,F}$ = 16.9 Hz, $^3J_{3,4b}$ = 7.6 Hz, $^3J_{3,4a}$ = 7.5 Hz, $^3J_{3,2}$ = 6.9 Hz, $^3J_{3,OH}$ = 4.2 Hz, 1H, H-3), 4.55 (ddd, $^2J_{4a,4b}$ = 9.5 Hz, $^3J_{4a,3}$ = 7.5 Hz, $^4J_{4a,F}$ = 1.3 Hz, 1H, H-4a), 4.07 (dd, $^2J_{4b,4a}$ = 9.5 Hz, $^3J_{4b,3}$ = 7.6 Hz, 1H, H-4b), 2.43 (d, $^3J_{OH,3}$ = 4.2 Hz, 1H, OH); ^{13}C NMR ($CDCl_3$, 176 MHz): δ = 168.72 (d, $^2J_{1,F}$ = 16.3 Hz, C-1), 90.66 (d, $^1J_{2,F}$ = 196.8 Hz, C-2), 71.39 (d, $^2J_{3,F}$ = 21.7 Hz, C-3), 68.37 (d, $^3J_{4,F}$ = 9.3 Hz, C-4); ^{19}F NMR ($CDCl_3$, 659 MHz): δ = −204.68 (ddd, $^2J_{F,2}$ = 51.0 Hz, $^3J_{F,3}$ = 16.9 Hz, $^4J_{F,4a}$ = 1.3 Hz).

3-*O*-*tert*-Butyldimethylsilyl-2-deoxy-2-fluoro-D-*threono*-1,4-lactone (39). 2-Deoxy-2-fluoro-D-*threono*-1,4-lactone (**38**, 155 mg, 1.3 mmol, 1 equiv.) was dissolved in DMF (8 mL) under argon atmosphere. TBSCl (389 mg, 2.6 mmol, 2 equiv.) and imidazole (176 mg, 2.6 mmol, 2 equiv.) were added and the reaction mixture was stirred at room temperature for 18 h. The solvent was removed *in vacuo*, the residue taken up in EtOAc (30 mL) and washed with brine (40 mL). The organic layer was dried ($MgSO_4$), filtered and evaporated under reduced pressure. The crude residue was purified by MPLC (25 g silica gel, 12-50% Et_2O in *n*-heptane) to give 3-*O*-*tert*-butyldimethylsilyl-2-deoxy-2-fluoro-D-*threono*-1,4-lactone (**39**) as a colorless oil (217 mg, 72%): R_f = 0.75 (*n*hept/EtOAc 1:1, CAM); $[\alpha]_D^{20}$ = −11.6 (*c* 0.9, $CHCl_3$); ^{1}H NMR ($CDCl_3$, 600 MHz): δ = 5.03 (dd, $^2J_{2,F}$ = 51.2 Hz, $^3J_{2,3}$ = 7.3 Hz, 1H, H-2), 4.66 (dddd, $^3J_{3,F}$ = 15.0 Hz, $^3J_{3,4b}$ = 8.1 Hz, $^3J_{3,2}$ = 7.4 Hz, $^3J_{3,4a}$ = 7.4 Hz, 1H, H-3), 4.42 (ddd, $^2J_{4a,4b}$ = 9.1 Hz, $^3J_{4a,3}$ = 7.4 Hz, $^4J_{4a,F}$ = 1.5 Hz, 1H, H-4a), 3.96 (dd, $^2J_{4b,4a}$ = 9.1 Hz, $^3J_{4b,3}$ = 8.1 Hz, 1H, H-4b), 0.91 (s, 3 CH_3^{tBu}), 0.15 (s, CH_3^{DMS}), 0.13 (s, CH_3^{DMS}); ^{13}C NMR ($CDCl_3$, 151 MHz): δ = 169.29 (d, $^2J_{1,F}$ = 22.4 Hz, C-1), 91.06 (d, $^1J_{2,F}$ = 198.0 Hz, C-2), 72.11 (d, $^2J_{3,F}$ = 20.9 Hz, C-3), 68.95 (d, $^3J_{4,F}$ = 10.9 Hz, C-4), 25.54 (s, 3 CH_3^{tBu}), 17.93 (s, C^{tBu}), –4.89 (s, CH_3^{DMS}), –5.09 (s, CH_3^{DMS}); ^{19}F NMR ($CDCl_3$, 400 MHz): δ = −204.18 (ddd, $^2J_{F,2}$ = 51.2 Hz, $^3J_{F,3}$ = 15.0 Hz, $^4J_{F,4a}$ = 1.5 Hz); HRMS (ESI+): m/z calc. for $C_{10}H_{19}O_3FSiNa^+$ $[M+Na]^+$: 257.0980, found: 257.0978.

2,5-Anhydro-4-*O*-*tert*-butyldimethylsilyl-1,3-dideoxy-3-fluoro-D-*threono*-pent-1-enitol (40). This reaction is light sensitive and was carried out in the dark. Petasis' reagent (**49**, 0.5 M in toluene, 3.75 mL, 2.2 equiv.) was added to 3-*O*-*tert*-butyldimethylsilyl-2-deoxy-2-fluoro-D-*threono*-1,4-lactone (39, 200 mg, 0.85 mmol, 1 equiv.) in toluene (0.7 mL) under argon atmosphere in the dark. The reaction mixture was stirred at 65°C for 24 h and then cooled to room temperature. *n*-Heptane (20 mL) was added and stirring was continued for 30 min. The orange suspension was then filtered over Celite®. After evaporating the solvent, the crude residue was purified by MPLC (50 g silica gel, 10-30% Et_2O in *n*-heptane + 0.5% (v/v) Et_3N) to give 2,5-anhydro-4-*O*-*tert*-butyldimethylsilyl-1,3-dideoxy-3-fluoro-D-*threono*-pent-1-enitol (**40**) as a light yellow oil (118 mg, 60%): R_f = 0.62 (*n*hept/Et_2O 5:1, CAM); $[\alpha]_D^{20}$ = −16.1 (*c* 1.0, $CHCl_3$); ^{1}H NMR ($CDCl_3$, 700 MHz): δ = 4.97 (dd, $^2J_{3,F}$ = 53.8 Hz, $^3J_{3,4}$ = 2.3 Hz, 1H, H-3), 4.59 (dd, $^2J_{1a,ab}$ = 6.2 Hz, $^3J_{1a,F}$ = 2.0 Hz, 1H, H-1a), 4.38 (dddd, $^3J_{4,F}$ = 12.2 Hz, $^3J_{4,5a}$ =

4.4 Hz, $^3J_{4,3}$ = 2.3 Hz, $^3J_{4,5b}$ = 2.4 Hz, 1H, H-4), 4.36 (dd, $^2J_{1b,1a}$ = 6.2 Hz, $^3J_{1b,F}$ = 2.0 Hz, 1H, H-1b), 4.25 (ddd, $^2J_{5a,5b}$ = 9.4 Hz, $^3J_{5a,4}$ = 4.4 Hz, $^4J_{5a,F}$ = 2.2 Hz, 1H, H-5a), 3.97 (dd, $^2J_{5b,5a}$ = 9.4 Hz, $^3J_{5b,4}$ = 2.4 Hz, 1H, H-5b), 0.89 (s, 3 CH_3^{tBu}), 0.12 (s, CH_3^{DMS}), 0.11 (s, CH_3^{DMS}); ^{13}C NMR ($CDCl_3$, 176 MHz): δ = 94.69 (d, $^1J_{3,F}$ = 181.3 Hz, C-3), 87.58 (d, $^4J_{1,F}$ = 9.3 Hz, C-1), 75.05 (s, C-5), 73.93 (d, $^3J_{4,F}$ = 26.5 Hz, C-4), 26.63 (3 CH_3^{tBu}), 18.01 (C^{tBu}), –4.85 (CH_3^{DMS}), –4.93 (CH_3^{DMS}); ^{19}F NMR ($CDCl_3$, 659 MHz): δ = −176.50 (m); HRMS (ESI+): m/z calc. for $C_{11}H_{21}O_2FSiH^+$ [M+H]$^+$: 233.1368, found: 233.1365.

4-*O-tert*-Butyldimethylsilyl-3-deoxy-3-fluoro-D-xylulose (41). 2,5-Anhydro-4-*O-tert*-butyldimethylsilyl-1,3-dideoxy-3-fluoro-D-*threono*-pent-1-enitol (**40**, 110 mg, 0.5 mmol, 1 equiv.) was dissolved in a 4:1 mixture of acetone and water (2.6 mL). Potassium osmate dihydrate (8.7 mg, 0.02 mmol, 0.05 equiv.) and NMO (110 mg, 0.9 mmol, 2 equiv.) were added and the reaction mixture was stirred at room temperature for 18 h. EtOAc (20 mL) was added, and the mixture was washed with water (2 × 25 mL). The organic layer was dried ($MgSO_4$), filtered and concentrated *in vacuo.* The crude residue was purified by MPLC (10 g silica gel, 12-100% EtOAc in *n*-heptane) to give 4-*O-tert*-butyldimethylsilyl-3-deoxy-3-fluoro-D-xylulose (**41**) as a mixture of anomers as colorless oil (99 mg, 79%): R_f = 0.32 (*n*hept/EtOAc 1:1, CAM); $[\alpha]_D^{20}$ = −16.6 (*c* 1.25, $CHCl_3$); <u>major anomer</u>: ^{1}H NMR ($CDCl_3$, 600 MHz): δ = 4.81 (dd, $^2J_{3,F}$ = 50.7 Hz, $^3J_{3,4}$ = 1.6 Hz, 1H, H-3), 4.42 (dddd, $^3J_{4,F}$ = 12.2 Hz, $^3J_{4,5a}$ = 4.3 Hz, $^3J_{4,3}$ = 1.6 Hz, $^3J_{4,5b}$ = 1.8 Hz, 1H, H-4), 4.19 (ddd, $^2J_{5a,5b}$ = 9.5 Hz, $^3J_{5a,4}$ = 4.3 Hz, $^3J_{5a,F}$ = 1.4 Hz, 1H, H-5a), 4.06 (broad s, 1H, OH-2), 4.02 (dd, $^2J_{5b,5a}$ = 9.5 Hz, $^3J_{5b,4}$ = 1.8 Hz, 1H, H-5b), 3.77 (dddd, $^2J_{1a,1b}$ = 9.0 Hz, $^3J_{1a,OH}$ = 8.1 Hz, $^4J_{1a,F}$ = 2.1 Hz, $^4J_{1a,OH}$ = 1.2 Hz, 1H, H-1a), 3.76 (ddd, $^2J_{1b,1a}$ = 9.0 Hz, $^3J_{1b,OH1}$ = 5.3 Hz, $^4J_{1b,F}$ = 1.0 Hz, 1H, H-1b), 1.95 (dd, $^3J_{OH,1a}$ = 8.1 Hz, $^3J_{OH,1b}$ = 5.3 Hz, 1H, OH-1), 0.91 (s, 3 CH_3^{tBu}), 0.15 (s, CH_3^{DMS}), 0.14 (s, CH_3^{DMS}); ^{13}C NMR ($CDCl_3$, 176 MHz): δ = 105.34 (d, $^2J_{2,F}$ = 23.7 Hz, C-2), 96.24 (d, $^1J_{3,F}$ = 189.1 Hz, C-3), 74.81 (d, $^2J_{4,F}$ = 28.4 Hz, C-4), 74.40 (s, C-5), 62.73 (d, $^2J_{1,F}$ = 5.3 Hz, C-1), 25.60 (3 CH_3^{tBu}), 17.91 (C^{tBu}), –5.05 (CH_3^{DMS}), –5.07 (CH_3^{DMS}); ^{19}F NMR ($CDCl_3$, 659 MHz): δ = -194.42 (dd, $^2J_{F,3}$ = 50.7 Hz, $^3J_{F,4}$ = 12.2 Hz); <u>minor anomer</u>: ^{1}H NMR ($CDCl_3$, 600 MHz): δ = 4.78 (dd, $^2J_{3,F}$ = 51.8 Hz, $^3J_{3,4}$ = 2.8 Hz, 1H, H-3), 4.52 (dddd, $^3J_{4,F}$ = 14.3 Hz, $^3J_{4,5a}$ = 5.2 Hz, $^3J_{4,3}$ = 2.8 Hz, $^3J_{4,5b}$ = 2.8 Hz, 1H, H-4), 4.21 (ddd, $^2J_{5a,5b}$ = 9.5 Hz, $^3J_{5a,4}$ = 5.2 Hz, $^3J_{5a,F}$ = 1.2 Hz, 1H, H-5a), 3.71 (ddd, $^2J_{5b,5a}$ = 9.5 Hz, $^3J_{5b,4}$ = 2.8 Hz, $^3J_{5b,F}$ = 0.6 Hz, 1H, H-5b), 3.70 (dddd, $^2J_{1a,1b}$ = 9.5 Hz, $^4J_{1a,OH}$ = 6.0 Hz, $^3J_{1a,OH}$ = 5.5 Hz, $^4J_{1a,F}$ = 3.5 Hz, 1H, H-1a), 3.65 (dd, $^2J_{1b,1a}$ = 9.5 Hz, $^3J_{1b,OH}$ = 8.0 Hz, 1H, H-1b), 3.41 (d, $^4J_{OH,1a}$ = 6.0 Hz, 1H, OH-2), 2.0 (dd, $^3J_{OH,1b}$ = 8.0 Hz, $^3J_{OH,1a}$ = 5.5 Hz, 1H, OH-1), 0.89 (s, 3 CH_3^{tBu}), 0.12 (s, CH_3^{DMS}), 0.09 (s, CH_3^{DMS}); ^{13}C NMR ($CDCl_3$, 176 MHz): δ = 103.06 (d, $^2J_{2,F}$ = 16.2 Hz, C-2), 96.00 (d, $^1J_{3,F}$ = 190.6 Hz, C-3), 74.84 (d, $^2J_{4,F}$ = 26.9 Hz, C-4), 72.20 (d, $^3J_{5,F}$ = 4.3 Hz, C-5), 64.81 (C-1), 25.62 (3 CH_3^{tBu}), 17.93 (C^{tBu}), –4.94 (CH_3^{DMS}), –5.05 (CH_3^{DMS}); ^{19}F NMR ($CDCl_3$, 659 MHz): δ = −200.40 (m); HRMS (ESI+): m/z calc. for $C_{11}H_{23}O_4FSiNa^+$ [M+Na]$^+$: 289.1242, found: 289.1245.

3-Deoxy-3-fluoro-D-xylulose (3DFXu, 5). Tetrabutylammonium fluoride (1 M in THF, 0.45 mL, 0.45 mmol, 2 equiv.) was added to 4-*O-tert*-butyldimethylsilyl-3-deoxy-3-fluoro-D-xylulose (**41**, 60 mg, 0.2 mmol, 1 equiv.) and the resulting mixture was stirred at room temperature for 1 h. The solvent was removed *in vacuo*, and the crude residue was purified by MPLC (10 g silica gel, 24-100% EtOAc in *n*-heptane) to yield 3-deoxy-3-fluoro-D-xylulose

(**5**) as a 4 : 5 : 1 mixture of anomers and the corresponding open chain conformation as a viscous oil (23 mg, 65%): R_f = 0.21 (EtOAc, CAM); $[\alpha]_D^{20}$ = −12.4 (*c* 0.5, $CHCl_3$); <u>major anomer</u>: ^{1}H NMR ($CDCl_3$, 700 MHz): δ = 4.91 (dd, $^2J_{3,F}$ = 51.9 Hz, $^3J_{3,4}$ = 4.2 Hz, 1H, H-3), 4.66 (dddd, $^3J_{4,F}$ = 17.0 Hz, $^3J_{4,5a}$ = 6.1 Hz, $^3J_{4,5b}$ = 5.5 Hz, $^3J_{4,3}$ = 4.2 Hz, 1H, H-4), 4.28 (dd, $^2J_{5a,5b}$ = 10.1 Hz, $^3J_{5a,4}$ = 6.1 Hz, 1H, H-5a), 3.74 (dd, $^2J_{5b,5a}$ = 10.2 Hz, $^3J_{5b,4}$ = 5.5 Hz, 1H, H-5b), 4.68 (dd, $^2J_{1a,1b}$ = 12.0 Hz, $^4J_{1a,F}$ = 2.0 Hz, 1H, H-1a), 3.63 (dd, $^2J_{1b,1a}$ = 12.0 Hz, $^3J_{1b,F}$ = 1.1 Hz, 1H, H-1b);^{13}C NMR ($CDCl_3$, 176 MHz) δ = 105.03 (d, $^2J_{2,F}$ = 16.8 Hz, C-2), 98.42 (d, $^1J_{3,F}$ = 191.9 Hz, C-3), 75.92 (d, $^3J_{4,F}$ = 25.3 Hz, C-4), 72.74 (d, $^3J_{1,F}$ = 7.0 Hz, C-5), 65.88 (s, C-1); ^{19}F NMR ($CDCl_3$, 659 MHz): δ = −203.00 (ddd, $^2J_{F,3}$ = 51.9 Hz, $^3J_{F,4}$ = 17.0 Hz, $^4J_{F,1a}$ = 2.0 Hz); <u>minor anomer</u>: ^{1}H NMR ($CDCl_3$, 700 MHz) δ = 4.90 (dd, $^2J_{3,F}$ = 50.4 Hz, $^3J_{3,4}$ = 1.5 Hz, 1H, H-3), 4.54 (dddd, $^3J_{4,F}$ = 19.0 Hz, $^3J_{4,5a}$ = 6.3 Hz, $^3J_{4,5b}$ = 4.4 Hz, $^3J_{4,3}$ = 1.5 Hz, 1H, H-4), 4.33 (dd, $^2J_{5a,5b}$ = 9.9 Hz, $^3J_{5a,4}$ = 6.3 Hz, 1H, H-5a), 3.96 (dd, $^2J_{5b,5a}$ = 9.9 Hz, $^3J_{5b,4}$ = 4.4 Hz, 1H, H-5b), 3.74 (ddd, $^2J_{1a,1b}$ = 10.9 Hz, $^4J_{1a,F}$ = 2.1 Hz, 1H, H-1a), 3.69 (dd, $^2J_{1b,1a}$ = 10.9 Hz, $^3J_{1b,F}$ = 1.0 Hz, 1H, H-1b); ^{13}C NMR ($CDCl_3$, 176 MHz): δ = 107.61 (d, $^2J_{2,F}$ = 26.0 Hz, C-2), 101.82 (d, $^1J_{3,F}$ = 183.7 Hz, C-3), 76.33 (d, $^3J_{4,F}$ = 27.9 Hz, C-4), 74.96 (d, $^3J_{1,F}$ = 2.38 Hz, C-5), 64.54 (d, $^3J_{5,F}$ = 5.5 Hz, C-1); ^{19}F NMR ($CDCl_3$, 659 MHz): δ = −194.85 (dddd, , $^2J_{F,3}$ = 50.4 Hz, $^3J_{F,4}$ = 19.0 Hz, $^4J_{F,1a}$ = 2.1 Hz, $^3J_{F,1b}$ = 1.0 Hz,); <u>open chain</u> form: ^{1}H NMR ($CDCl_3$, 700 MHz): δ = 5.29 (dd, $^2J_{3,F}$ = 47.0 Hz, $^3J_{3,4}$ = 1.5 Hz, 1H, H-3), 4.70 (dd, $^2J_{1a,1b}$ = 19.9 Hz, $^4J_{1a,F}$ = 1.7 Hz, 1H, H-1a), 4.56 (dd, $^2J_{1b,1a}$ = 19.9 Hz, $^4J_{1b,F}$ = 1.5 Hz, 1H, H-1b), (4.18 (dddd, $^3J_{4,F}$ = 29.8 Hz, $^3J_{4,5b}$ = 6.9 Hz, $^3J_{4,5a}$ = 2.7 Hz, $^3J_{4,3}$ = 1.5 Hz, 1H, H-4), 3.68 (ddd, $^2J_{5a,5b}$ = 12.0 Hz, $^3J_{5a,4}$ = 2.7 Hz, $^4J_{5a,F}$ = 1.1 Hz, 1H, H-5a), 3.69 (ddd, $^2J_{5b,5a}$ = 12.0 Hz, $^3J_{5b,4}$ = 6.9 Hz, $^4J_{5b,F}$ = 1.0 Hz, 1H, H-5b); ^{13}C NMR ($CDCl_3$, 176 MHz): δ = 212.28 (d, $^2J_{2,F}$ = 23.9 Hz, C-2), 97.47 (d, $^1J_{3,F}$ = 185.5 Hz, C-3), 73.84 (d, $^3J_{4,F}$ = 18.5 Hz, C-4), 68.95 (d, $^3J_{1,F}$ = 4.1 Hz, C-1), 63.60 (d, $^3J_{5,F}$ = 5.8 Hz, C-5); ^{19}F NMR ($CDCl_3$, 659 MHz): δ = -194.85 (dddd, $^2J_{F,3}$ = 47.0 Hz, $^3J_{F,4}$ = 29.8 Hz, $^4J_{F,1a}$ = 1.7 Hz); HRMS (ESI+): m/z calc. for $C_5H_9O_4FNa^+$ $[M+Na]^+$: 175.0377, found: 175.0373.

2-*O*-Triisopropylsilyl-D-*erythrono*-1,4-lactone (42). TIPSCl (1.958 g, 10.15 mmol, 2.17 mL, 1.2 equiv.) and imidazole (1.151 g, 16.9 mmol, 2 equiv.) were added to a solution of D-*erythrono*-1,4-lactone (**10**, 1 g, 8.46 mmol, 1 equiv.) in dry THF (12 mL) under argon atmosphere. The reaction mixture was stirred at room temperature for 18 h. After evaporation of the solvent *in vacuo*, the residue was taken up in EtOAc (50 mL) and washed with brine (50 mL). The organic layer was dried ($MgSO_4$), filtered and concentrated under reduced pressure. The crude residue was purified by MPLC (50 g silica gel, 12-100% EtOAc in *n*-heptane) to yield 2-*O*-triisopropylsilyl-D-*erythrono*-1,4-lactone (**42**) as a colorless oil (1.578 g, 68%): R_f = 0.57 (*n*hept/EtOAc 1:1, CAM); $[\alpha]_D^{20}$ = +19.7 (*c* 1.0, $CHCl_3$); ^{1}H NMR ($CDCl_3$, 700 MHz): δ = 4.54 (d, $^3J_{3,4}$ = 4.8 Hz, 1H, H-3), 4.39 (ddd, $^3J_{3,2}$ = 4.8 Hz, $^3J_{3,4b}$ = 3.2 Hz, $^3J_{3,OH}$ = 1.1 Hz, 1H, H-3), 4.36 (d, $^2J_{4a,4b}$ = 10.6 Hz, 1H, H-4a), 4.26 (ddd, $^2J_{4b,4a}$ = 10.6 Hz, $^3J_{4b,3}$ = 3.2 Hz, $^4J_{4b,OH}$ = 1.6 Hz 1H, H-4b), 2.89 (dd, $^4J_{OH,4b}$ = 1.6 Hz, $^3J_{OH,3}$ = 1.1 Hz, 1H, OH-3), 1.24 (sept, $^3J_{CH,CH3}$ = 7.4 Hz, 3H, CH^{TIPS}), 1.12 (ddd, $^3J_{CH3,CH}$ = 7.4 Hz, 18H, CH_3^{TIPS}); ^{13}C NMR ($CDCl_3$, 176 MHz): δ = 173.56 (C-1), 70.56 (C-4), 70.34 (C-2), 68.80 (C-3), 17.75 (4 CH_3^{TIPS}), 17.68 (2 CH_3^{TIPS}), 12.09 (3 CH^{TIPS}); HRMS (ESI+): m/z calc. for $C_{13}H_{26}O_4SiNa^+$ $[M+Na]^+$: 297.1493, found: 297.1493.

3-*O*-Benzoyl-2-*O*-triisopropylsilyl-D-*erythrono*-1,4-lactone (43). Benzoyl chloride (1183 mg, 8.4 mmol, 2.1 equiv.) and 4-(dimethylamino)pyridine (25 mg, 0.2 mmol, 0.05 equiv.) were added to a solution of 2-*O*-triisopropylsilyl-D-*erythrono*-1,4-lactone (**42**, 1.1 g, 4.0 mmol, 1 equiv.) in dry pyridine (21 mL). After stirring for 18 h at room temperature, pyridine was removed *in vacuo*. The residue was taken up in EtOAc (60 mL) and washed with aq. HCl (1 M, 3 × 40 mL) and brine (40 mL). The organic layer was dried ($MgSO_4$), filtered and evaporated under reduced pressure. The resulting crude residue was purified by MPLC (100 g silica gel, 12-100% EtOAc in *n*-heptane) to yield 3-*O*-benzoyl-2-*O*-triisopropylsilyl-D-*erythrono*-1,4-lactone (**43**) as a colorless oil (1.229 g, 81%): R_f = 0.71 (*n*hept/EtOAc 1:1, UV light and CAM); $[\alpha]_D^{20}$ = −85.7 (*c* 1.0, $CHCl_3$); ^{1}H NMR ($CDCl_3$, 700 MHz): δ = 8.08-7.41 (m, 5H, H^{Ar}), 5.79 (dd, $^3J_{3,2}$ = 5.1 Hz, $^3J_{3,4a}$ = 3.3 Hz, 1H, H-3), 4.73 (d, $^3J_{2,3}$ = 5.1 Hz, 1H, H-2), 4.50 (d, $^2J_{4a,4b}$ = 11.3 Hz, $^3J_{4a,3}$ = 3.3 Hz, 1H, H-4a), 4.45 (d, $^2J_{4b,4a}$ = 11.3 Hz, 1H, H-4b), 1.16 (sept, $^3J_{CH,CH3}$ = 7.4 Hz, 3H, CH^{TIPS}), 1.04 (ddd, $^3J_{CH3,CH}$ = 7.4 Hz, 18H, CH_3^{TIPS}); ^{13}C NMR ($CDCl_3$, 176 MHz): δ = 173.32 (C-1), 165.61 (C^{Bz}), 133.56 (CH^{Ar}), 129.94 (2 CH^{Ar}), 129.10 (C^{Ar}), 128.44 (2 CH^{Ar}), 71.13 (C-3), 69.38 (C-2), 68.86 (C-4), 17.67 (4 CH_3^{TIPS}), 17.63 (2 CH_3^{TIPS}), 12.10 (3 CH^{TIPS}); HRMS (ESI+): m/z calc. for $C_{20}H_{30}O_5SiNa^+$ $[M+Na]^+$: 401.1755, found: 401.1752

2,5-Anhydro-4-*O*-benzoyl-1-deoxy-3-*O*-triisopropylsilyl-D-*erythro*-pent-1-enitol (44). This reaction is light sensitive and was carried out in the dark. 3-*O*-Benzoyl-2-*O*-triisopropylsilyl-D-*erythrono*-1,4-lactone (**43**, 1.00 g, 2.6 mmol, 1 equiv.) was dissolved in dry toluene (0.7 mL) under argon atmosphere and Petasis' reagent (0.5 M in dry toluene, 11.6 mL, 2.2 equiv.) was added. The flask was equipped with a reflux condenser and the reaction mixture was stirred at 70°C for 24 h. After cooling to room temperature, *n*-heptane (50 mL) was added and stirring was continued for 30 min. The orange suspension was filtered over Celite®, the filtrate concentrated, and the crude residue was purified by MPLC (100 g silica gel, 5-30% Et_2O in *n*-heptane + 0.5% Et_3N (v/v)) to yield 2,5-anhydro-4-*O*-benzoyl-1-deoxy-3-*O*-triisopropylsilyl-D-*erythro*-pent-1-enitol (**44**) as a light yellow oil (750 mg, 76%): R_f = 0.65 (*n*hept/Et_2O 5:1, UV light and CAM); $[\alpha]_D^{20}$ = −114.8 (*c* 1.0, $CHCl_3$); ^{1}H NMR ($CDCl_3$, 600 MHz): δ = 8.10-7.40 (m, 5H, H^{Ar}), 5.47 (ddd, $^3J_{4,3}$ = 3.6 Hz, $^3J_{4,5a}$ = 3.6 Hz, $^3J_{4,5b}$ = 1.5 Hz, 1H, H-4), 4.93 (ddd, $^3J_{3,4}$ = 3.6 Hz, $^3J_{3,1a}$ = 2.0 Hz, $^3J_{3,1b}$ = 2.0 Hz, 1H, H-3), 4.46 (broad dd, $^2J_{1a,1b}$ = 4.0 Hz, $^3J_{1a,3}$ = 2.0, 1H, H-1a), 4.33 (dd, $^2J_{5a,5b}$ = 10.7 Hz, $^3J_{5a,4}$ = 3.6 Hz, 1H, H-5a), 4.24 (dd, $^2J_{1b,1a}$ = 4.0 Hz, $^3J_{1b,3}$ = 2.0, 1H, H-1b), 4.23 (dd, $^2J_{5b,5a}$ = 10.7 Hz, $^3J_{5b,4}$ = 1.5, 1H, H-5b), 1.12 (sept, $^3J_{CH,CH3}$ = 7.4 Hz, 3H, CH^{TIPS}), 1.04 (ddd, $^3J_{CH3,CH}$ = 7.4 Hz, 18H, CH_3^{TIPS}); ^{13}C NMR ($CDCl_3$, 176 MHz): δ = 166.15 (C^{BZ}), 161.49 (C-2), 133.21 (CH^{Ar}), 129.89 (2 CH^{Ar}), 129.74 (C^{Ar}), 128.31 (CH^{Ar}), 82.04 (C-1), 73.08 (C-4), 71.62 (C-3), 71.20 (C-5), 17.88 (4 CH_3^{TIPS}), 17.83 (2 CH_3^{TIPS}), 12.26 (3 CH^{TIPS}); HRMS (ESI+): m/z calc. for $C_{21}H_{32}O_4SiNa^+$ $[M+Na]^+$: 399.1962, found: 399.1962.

4-*O*-Benzoyl-3-*O*-triisopropylsilyl-D-ribulose (45). 2,5-Anhydro-4-*O*-benzoyl-3-*O*-triisopropylsilyl-1-deoxy-D-*erythro*-pent-1-entiol (**44**, 720 mg, 1.9 mmol, 1 equiv.) was dissolved in a 5:1 mixture of acetone and water (10 mL). Potassium osmate dihydrate (35 mg, 0.1 mmol, 0.05 equiv.) and NMO (447 mg, 3.8 mmol, 2 equiv.) were added and the reaction mixture was stirred at room temperature for 18 h. EtOAc (50 mL) was added, and

the mixture was washed with water (2 × 40 mL). The organic layer was dried ($MgSO_4$), filtered and evaporated *in vacuo.* The crude residue was purified by MPLC (50 g silica gel, 12-100% EtOAc in *n*-heptane) to yield 4-*O*-benzoyl-3-*O*-triisopropylsilyl-D-ribulose (**45**) as a colorless oil (572 mg, 73%): R_f = 0.39 (*n*hept/EtOAc 1:1, UV light and CAM); $[\alpha]_D^{20}$ = −84.5 (*c* 1.0, $CHCl_3$); major anomer: ^{1}H NMR ($CDCl_3$, 600 MHz): δ = 8.08-7.41 (m, 5H, H^{Ar}), 5.56 (ddd, $^3J_{4,3}$ = 5.0 Hz, $^3J_{4,5a}$ = 4.9 Hz, $^3J_{4,5b}$ = 2.0 Hz, 1H, H-4), 4.69 (d, $^3J_{3,4}$ = 5.0 Hz, 1H, H-3), 4.20 (dd, $^2J_{5a,5b}$ = 10.8 Hz, $^3J_{5a,4}$ = 4.9 Hz, 1H, H-5a), 4.16 (dd, $^2J_{5b,5a}$ = 10.8 Hz, $^3J_{5b,4}$ = 2.0 Hz, 1H, H-5b), 4.05 (s, 1H, OH-2), 3.71 (dd, $^2J_{1a,1b}$ = 12.0 Hz, $^3J_{1a,OH}$ = 3.7 Hz, 1H, H-1a), 3.60 (dd, $^2J_{1b,1a}$ = 12.0 Hz, $^3J_{1b,OH}$ = 10.5 Hz, 1H, H-1b), 1.83 (dd, $^2J_{OH,1b}$ = 10.5 Hz, $^3J_{OH,1a}$ = 3.7 Hz, 1H, OH-1), 1.11 (sept, $^3J_{CH,CH3}$ = 7.4 Hz, 3H, CH^{TIPS}), 1.04 (ddd, $^3J_{CH3,CH}$ = 7.4 Hz, 18H, CH_3^{TIPS}); ^{13}C NMR ($CDCl_3$, 151 MHz): δ = 165.80 (C^{Bz}), 133.43 (CH^{Ar}), 129.70 (2 CH^{Ar}), 129.51 (C^{Ar}), 128.49 (2 CH^{Ar}), 102.61 (C-2), 73.35 (C-4), 70.83 (C-3), 70.57 (C-5), 63.53 (C-1), 17.78 (4 CH_3^{TIPS}), 17.71 (2 CH_3^{TIPS}), 12.33 (3 × CH^{TIPS}); minor anomer: ^{1}H NMR ($CDCl_3$, 600 MHz): δ = 8.08-7.41 (m, 5H, H^{Ar}), 5.53 (ddd, $^3J_{4,3}$ = 5.0 Hz, $^3J_{4,5b}$ = 5.0 Hz, $^3J_{4,5a}$ = 3.0 Hz, 1H, H-4), 4.67 (d, $^3J_{3,4}$ = 5.0 Hz, 1H, H-3), 4.29 (dd, $^2J_{5a,5b}$ = 11.2 Hz, $^3J_{5a,4}$ = 3.0 Hz, 1H, H-5a), 4.03 (broad d, $^2J_{5b,5a}$ = 11.2 Hz, 1H, H-5b), 3.99 (dd, $^2J_{1a,1b}$ = 11.7 Hz, $^3J_{1a,OH}$ = 4.3 Hz, 1H, H-1a), 3.84 (dd, $^2J_{1b,1a}$ = 11.7 Hz, $^3J_{1b,OH}$ = 9.7 Hz, 1H, H-1b), 3.60 (s, 1H, OH-2), 2.22 (dd, $^2J_{OH,1b}$ = 9.7 Hz, $^3J_{OH,1a}$ = 4.3 Hz, 1H, OH-1), 1.11 (sept, $^3J_{CH,CH3}$ = 7.4 Hz, 3H, CH^{TIPS}), 1.04 (ddd, $^3J_{CH3,CH}$ = 7.4 Hz, 18H, CH_3^{TIPS}); ^{13}C NMR (151 MHz, $CDCl_3$) δ = 165.88 (C^{BZ}), 133.31 (CH^{Ar}), 129.73 (C^{Ar}), 129.68 (2 CH^{Ar}), 128.47 (2 CH^{Ar}), 104.63 (C-2), 79.01 (C-3), 75.04 (C-4), 69.30 (C-5), 66.15 (C-1), 17.75 (4 CH_3^{TIPS}), 17.71 (2 CH_3^{TIPS}), 12.04 (3 CH^{TIPS}); HRMS (ESI+): m/z calc. for $C_{21}H_{32}O_4SiNa^+$ $[M+Na]^+$: 433.2017, found: 433.2020.

4-*O*-Benzoyl-1,2-*O*-isopropylidene-3-*O*-triisopropylsilyl-D-ribulose (46). 4-*O*-Benzoyl-3-*O*-triisopropylsilyl-D-ribulose (**45**, 300 mg, 0.7 mmol, 1 equiv.) was dissolved in a mixture of 1,2-dimethoxyethane (0.35 mL) and 1,3-dimethoxypropane (228 mg, 2.2 mmol, 0.27 mL, 3 equiv.). $SnCl_2$ (7.0 mg, 0.04 mmol, 0.05 equiv.) was added and the reaction mixture was stirred at 60°C for 1.5 h. Pyridine (0.05 mL) was added and all solvents were removed *in vacuo*. The crude residue was purified by MPLC (50 g silica gel, 12-100% EtOAc in *n*-heptane) to yield 4-*O*-benzoyl-1,2-*O*-isopropylidene-3-*O*-triisopropylsilyl-D-ribulose (**46**) as mixture of anomers as colorless oil (227 mg, 69%): R_f = 0.81 (nhept/EtOAc1:1, UV light and CAM); $[\alpha]_D^{20}$ = −99.2 (*c* 1, $CHCl_3$); major anomer: ^{1}H NMR ($CDCl_3$, 600 MHz): δ = 8.16-7.40 (m, 5H, H^{Ar}), 5.44 (dd, $^3J_{4,3}$ = 4.2 Hz, $^3J_{4,5a}$ = 3.6 Hz, 1H, H-4), 4.67 (d, $^3J_{3,4}$ = 4.2 Hz, 1H, H-3), 4.57 (d, $^2J_{1a,1b}$ = 8.8 Hz, 1H, H-1a), 4.26 (dd, $^2J_{5a,5b}$ = 11.0 Hz, $^3J_{5a,4}$ = 3.6 Hz, 1H, H-5a), 4.05 (d, $^2J_{1b,1a}$ = 8.8 Hz, 1H, H-1b), 3.98 (d, $^2J_{5b,5a}$ = 11.0 Hz, 1H, H-5b), 1.51 (s, CH_3^{ipr}), 1.46 (s, CH_3^{ipr}), 1.10 (sept, $^3J_{CH,CH3}$ = 7.4 Hz, 3H, CH^{TIPS}), 1.04 (ddd, $^3J_{CH3,CH}$ = 7.4 Hz, 18H, CH_3^{TIPS}); ^{13}C NMR ($CDCl_3$, 151 MHz): δ = 165.86 (C^{Bz}), 133.31 (CH^{Ar}), 129.72 (C^{Ar}), 129.60 (2 CH^{Ar}), 128.48 (2 CH^{Ar}), 112.36 (C-2), 75.20 (C-3), 73.87 (C-4), 70.39 (C-1), 69.08 (C-5), 26.08 (CH_3^{ipr}), 25.85 (CH_3^{ipr}), 25.84 (C^{ipr}), 17.83 (4 CH_3^{TIPS}), 17.79 (2 CH_3^{TIPS}), 12.19 (3 CH^{TIPS}); minor anomer: ^{1}H NMR ($CDCl_3$, 600 MHz): δ = 8.08-7.41 (m, 5H, H^{Ar}), 5.40 (ddd, $^3J_{4,3}$ = 5.6 Hz, $^3J_{4,5a}$ = 5.6 Hz, $^3J_{4,5b}$ = 1.7 Hz, 1H, H-4), 4.57 (d, $^3J_{3,4}$ = 5.6 Hz, 1H, H-3), 4.51 (d, $^2J_{1a,1b}$ = 8.9 Hz, 1H, H-1a), 4.29 (dd, $^2J_{5a,5b}$ = 11.0 Hz, $^3J_{5a,4}$ = 5.6 Hz, 1H, H-5a), 4.03 (d, $^2J_{1b,1a}$ = 8.9 Hz, 1H, H-1b), 4.00 (dd, $^2J_{5b,5a}$ = 11.0 Hz, $^3J_{5b,4}$ = 1.7 Hz, 1H, H-5b), 1.49 (s, CH_3^{ipr}), 1.45 (s, CH_3^{ipr}), 1.10 (sept, $^3J_{CH,CH3}$ = 7.4 Hz, 3H, CH^{TIPS}), 1.04 (ddd, $^3J_{CH3,CH}$ = 7.4

Hz, 18H, CH_3^{TIPS}); ^{13}C NMR ($CDCl_3$, 151 MHz): δ = 165.85 (C^{Bz}), 133.04 (CH^{Ar}), 130.08 (2 CH^{Ar}), 129.59 (C^{Ar}), 128.20 (2 CH^{Ar}), 110.15 (C-2), 75.40 (C-3), 71.47 (C-4), 70.85 (C-5), 70.39 (C-1), 26.95 (C^{ipr}), 26.76 (CH_3^{ipr}), 26.47 (CH_3^{ipr}), 17.89 (4 CH_3^{TIPS}), 17.86 (2 CH_3^{TIPS}), 12.47 (3 CH^{TIPS}); HRMS (ESI+): m/z calc. for $C_{24}H_{38}O_6SiNa^+$ $[M+Na]^+$: 473.2330, found: 473.2325

4-*O*-Benzoyl-1,2-*O*-isopropylidene-D-ribulose (47). 4-*O*-Benzoyl-1,2-*O*-isopropylidene-3-*O*-triisopropylsilyl-D-ribulose (**46**, 120 mg, 0.3 mmol, 1 equiv.) was dissolved in dry THF (0.5 mL) under argon atmosphere and cooled to 0° C. Tetrabutylammonium fluoride (1 M in THF, 0.56 mL, 0.6 mmol, 2 equiv.) was added and the reaction mixture was stirred at 0°C for 1 h. A sat. aq. $NaHCO_3$-sol. (3 mL) was added, and the product was extracted with EtOAc (3 × 10 mL). The combined organic layers were dried ($MgSO_4$), filtered and concentrated *in vacuo.* The crude residue was purified by MPLC (10 g silica gel, toluene/EtOAc 3:1) to yield 4-*O*-benzoyl-1,2-*O*-isopropylidene-D-ribulose (**47**) as a mixture of anomers as colorless oil (31 mg, 40%): R_f = 0.63 (toluene/EtOAc 1:1, UV light and CAM); $[\alpha]_D^{20}$ = −88.6 (*c* 1.0, $CHCl_3$); major anomer: ^1H NMR ($CDCl_3$, 600 MHz): δ = 8.06-7.44 (m, 5H, H^{Ar}), 5.58 (ddd, $^3J_{4,5a}$ = 6.0 Hz, $^3J_{4,3}$ = 5.6 Hz, $^3J_{4,5b}$ = 4.1 Hz, 1H, H-4), 4.42 (d, $^2J_{1a,1b}$ = 9.9 Hz, 1H, H-1a), 4.41 (dd, $^3J_{3,4}$ = 5.6 Hz, $^3J_{3,OH}$ = 5.2 Hz, 1H, H-3), 4.37 (dd, $^2J_{5a,5b}$ = 10.1 Hz, $^3J_{5a,4}$ = 6.0 Hz, 1H, H-5a), 4.07 (d, $^2J_{1b,1a}$ = 9.9 Hz, 1H, H-1b), 4.02 (dd, $^2J_{5b,5a}$ = 10.1 Hz, $^3J_{5b,4}$ = 4.1 Hz, 1H, H-5b), 2.21 (d, $^3J_{OH,3}$ = 5.2 Hz, 1H, OH), 1.51 (s, CH_3^{ipr}), 1.43 (s, CH_3^{ipr}); ^{13}C NMR ($CDCl_3$, 151 MHz): δ = 166.0 (C^{Bz}), 133.63 (CH^{Ar}), 129.72 (2 CH^{Ar}), 129.10 (C^{Ar}), 128.59 (2 CH^{Ar}), 111.09 (C-2), 74.74 (C-3), 73.97 (C-4), 69.19 (C-1), 68.70 (C-5), 26.35 (CH^{ipr}), 26.01 (CH_3^{ipr}); minor anomer: ^1H NMR ($CDCl_3$, 600 MHz): δ = 8.10-7.44 (m, 5H, H^{Ar}), 5.49 (ddd, $^3J_{4,3}$ = 8.0 Hz, $^3J_{4,5a}$ = 5.9 Hz, $^3J_{4,5b}$ = 2.3 Hz, 1H, H-4), 4.30 (dd, $^2J_{5a,5b}$ = 11.0 Hz, $^3J_{5a,4}$ = 5.9 Hz, 1H, H-5a), 4.16 (d, $^2J_{1a,1b}$ = 9.1 Hz, 1H, H-1a), 4.12 (d, $^2J_{1b,1a}$ = 9.1 Hz, 1H, H-1b), 4.14 (dd, $^3J_{3,OH}$ = 11.7 Hz, $^3J_{3,4}$ = 8.0 Hz, 1H, H-3), 4.05 (dd, $^2J_{5b,5a}$ = 11.0 Hz, $^3J_{5b,4}$ = 2.3 Hz, 1H, H-5b), 2.53 (d, $^3J_{OH,3}$ = 11.7 Hz, 1H, OH), 1.57 (s, CH_3^{ipr}), 1.47 (s, CH_3^{ipr}); ^{13}C NMR ($CDCl_3$, 151 MHz): δ = 165.74 (C^{Bz}), 133.35 (CH^{Ar}), 129.79 (2 CH^{Ar}), 129.21 (C^{Ar}), 128.48 (2 CH^{Ar}), 112.59 (C-2), 71.33 (C-3), 70.78 (C-4), 70.71 (C-1), 70.67 (C-5), 26.69 (CH_3^{ipr}), 26.25 (CH_3^{ipr}); HRMS (ESI+): m/z calc. for $C_{15}H_{18}O_6Na^+$ $[M+Na]^+$: 317.0996, found: 317.0996.

4-*O*-Benzoyl-1,2-*O*-isopropylidene-3-*O*-trifluoromethanesulfonyl-D-ribulose (48). Pyridine (43 mg, 0.04 mL, 0.6 mmol, 8.5 equiv.) and Tf_2O (38 mg, 0.022 mL, 0.1 mmol, 2 equiv.) were added to a solution of 4-*O*-Benzoyl-1,2-*O*-isopropylidene-D-ribulose (**47**, 20 mg, 0.07 mmol, 1 equiv.) in dry CH_2Cl_2 (0.6 mL) at 0 °C. The reaction mixture was stirred at 0°C for 40 minutes before the addition of ice-cold aq. HCl (1 M, 0.6 mL).The layers were separated, the aqueous layer extracted with CH_2Cl_2 (2 × 10 mL), and the combined organic layers washed with sat. aq. $NaHCO_3$-solution (20 mL), dried ($MgSO_4$), filtered and concentrated to yield 4-*O*-benzoyl-1,2-*O*-isopropylidene-3-*O*-trifluoromethanesulfonyl-D-ribulose (**48**) as a 5:1 mixture of anomers as yellow oil (24 mg, 80%): $[\alpha]_D^{20}$ = −82.3 (*c* 1.0, $CHCl_3$); ^1H NMR ($CDCl_3$, 700 MHz): δ = 8.12-7.44 (m, 5H, H^{Ar}), 5.86 (ddd, $^3J_{4,5a}$ = 7.0 Hz, $^3J_{4,5b}$ = 5.9 Hz, $^3J_{4,3}$ = 4.3 Hz, 1H, H-4), 5.22 (d, $^3J_{3,4}$ = 4.3 Hz, 1H, H-3), 4.47 (dd, $^2J_{5a,5b}$ = 9.5 Hz, $^3J_{5a,4}$ = 7.0 Hz, 1H, H-5a), 4.32 (d, $^2J_{1a,1b}$ = 10.0 Hz, 1H, H-1a), 4.16 (d, $^2J_{1b,1a}$ = 10.0 Hz, 1H, H-1b), 4.01 (dd, $^2J_{5b,5a}$ = 9.5 Hz, $^3J_{5b,4}$ = 5.9 Hz, 1H, H-5b), 1.51 (s, CH_3^{ipr}), 1.43 (s, CH_3^{ipr});

^{13}C NMR ($CDCl_3$, 176 MHz): δ = 165.44 (C-2), 164.87 (C^{Bz}), 133.87 (CH^{Ar}), 129.98 (2 CH^{Ar}), 129.73 (C^{Ar}), 128.63 (2 CH^{Ar}), 86.08 (C-3), 70.73 (C-4), 69.02 (C-1), 68.26 (C-5), 29.69 (C^{ipr}), 26.11 (CH_3^{ipr}), 25.75 (CH_3^{ipr}); ^{19}F NMR ($CDCl_3$, 659 MHz): δ = −74.56 (s); <u>minor anomer</u>: ^{1}H NMR ($CDCl_3$, 700 MHz): δ = 8.12-7.44 (m, 5H, H^{Ar}), 5.53 (ddd, $^3J_{4,3}$ = 6.4 Hz, $^3J_{4,5a}$ = 6.4 Hz, $^3J_{4,5b}$ = 5.7 Hz, 1H, H-4), 5.06 (d, $^3J_{3,4}$ = 6.4 Hz, 1H, H-3), 4.40 (dd, $^2J_{5a,5b}$ = 10.0 Hz, $^3J_{5a,4}$ = 6.4 Hz, 1H, H-5a), 4.20 (d, $^2J_{1a,1b}$ = 9.8 Hz, 1H, H-1a), 4.18 (d, $^2J_{1b,1a}$ = 9.8 Hz, 1H, H-1b), 4.10 (dd, $^2J_{5b,5a}$ = 10.0 Hz, $^3J_{5b,4}$ = 5.7 Hz, 1H, H-5b), 1.54 (s, CH_3^{ipr}), 1.50 (s, CH_3^{ipr}); ^{13}C NMR ($CDCl_3$, 176 MHz): δ = 165.44 (C^{Bz}), 133.78 (CH^{Ar}), 129.98 (2 CH^{Ar}), 128.56 (2 CH^{Ar}), 128.43 (C^{Ar}), 110.25 (C-2), 81.10 (C-3), 68.19 (C-4), 71.17 (C-1), 69.37 (C-5), 29.69 (C^{ipr}), 25.99 (CH_3^{ipr}), 25.82 (CH_3^{ipr}); ^{19}F NMR ($CDCl_3$, 659 MHz): δ = –74.81 (s)**;** HRMS (ESI+): m/z calc. for $C_{16}H_{17}O_8F_3SNa^+$ $[M+Na]^+$: 449.0488, found: 449.0499.

NMR spectra

Pictures of the recorded ^{1}H, ^{13}C and ^{19}F NMR spectra of all synthetized compounds are available as additional material. All assignments, solvents and device parameters are given in the **Synthetic Procedures** (section above). The first spectrum shown in each series is the full ^{1}H NMR spectrum. Expansions are depicted where they were regarded as necessary. Then the ^{13}C NMR spectrum is depicted in the same manner, followed by the proton decoupled - and finally the proton coupled ^{19}F NMR spectrum. The x-axes and peak labels are is in ppm for all shown spectra. Structures are always given on top of the full ^{1}H NMR spectrum. Integrals are denoted below the x-axes where they are regarded as necessary and the integration range is marked. The numbering and order of compounds is in accordance with the numbering and order of substances in the main text.

^{1}H NMR (600 MHz, $CDCl_3$) of bis(cyclopentadienyl)dimethyltitanium/Petasis' reagent (49)

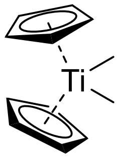

49

$C_{12}H_{16}Ti$

208.13 g/mol

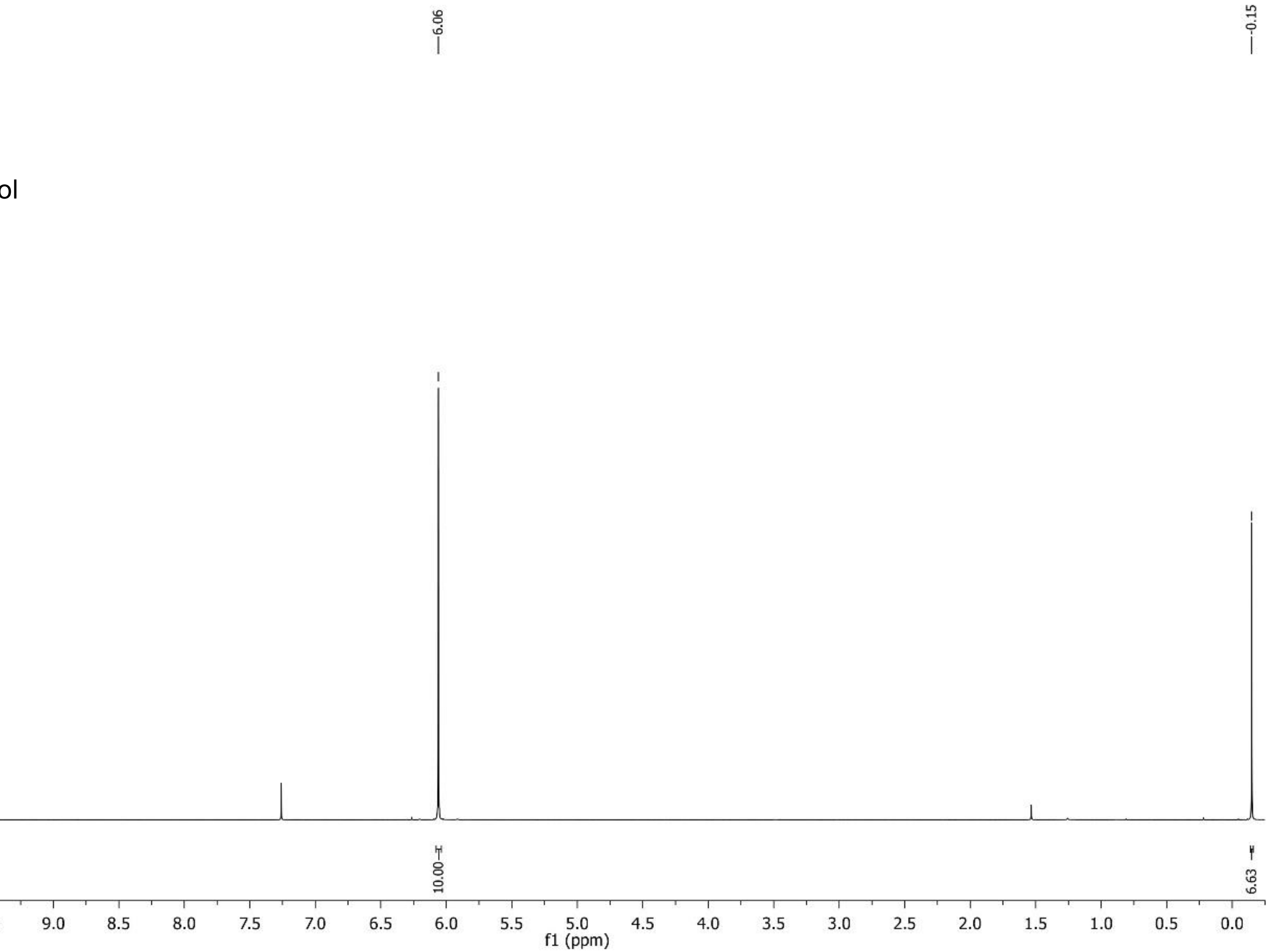

^{13}C NMR (151 MHz, $CDCl_3$) of bis(cyclopentadienyl)dimethyltitanium/Petasis' reagent (49)

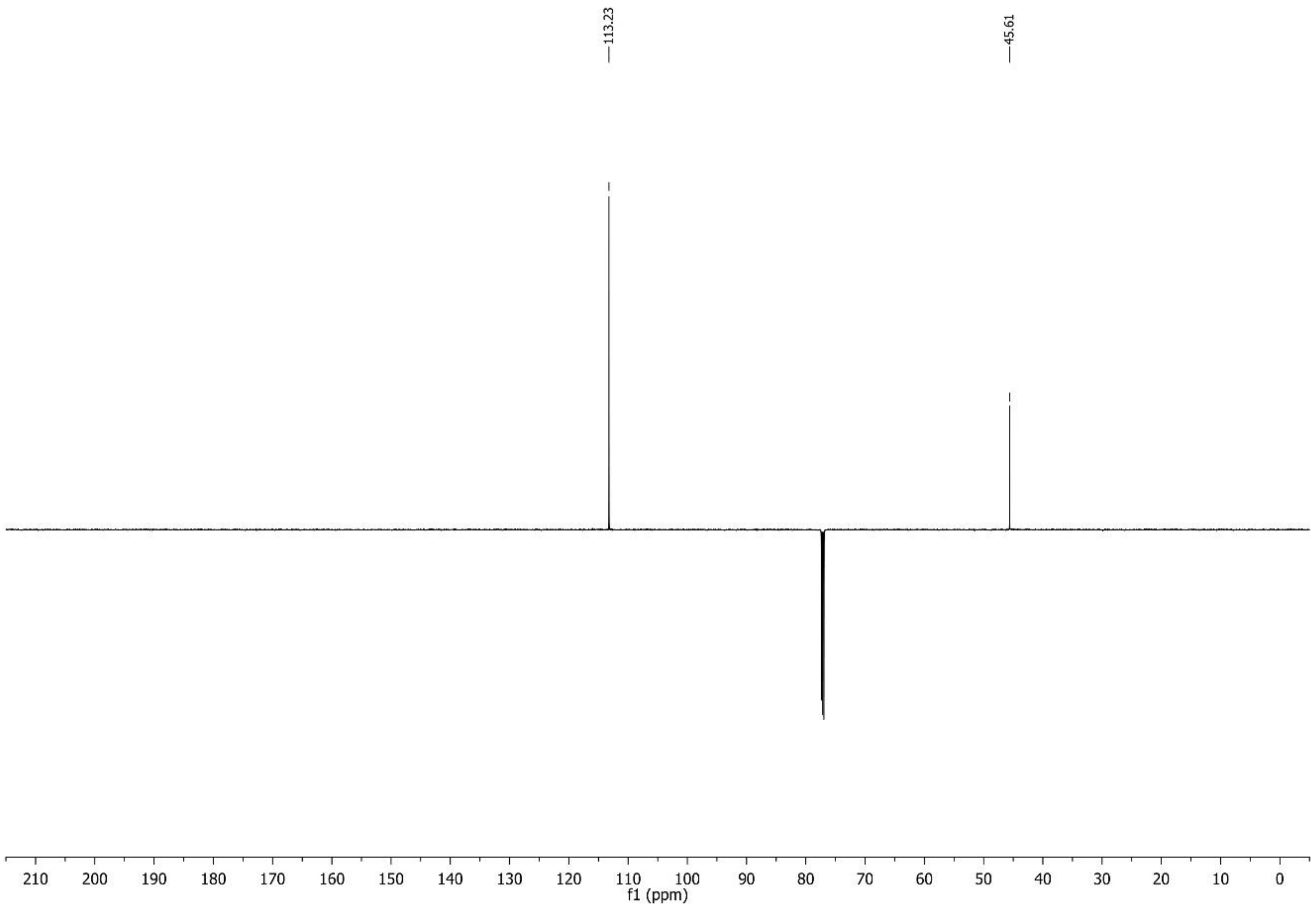

^{1}H NMR (600 MHz, d_6-DMSO) of D-*erythrono*-1,4-lactone (10)

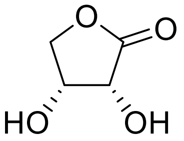

10

$C_4H_6O_4$

118.09 g/mol

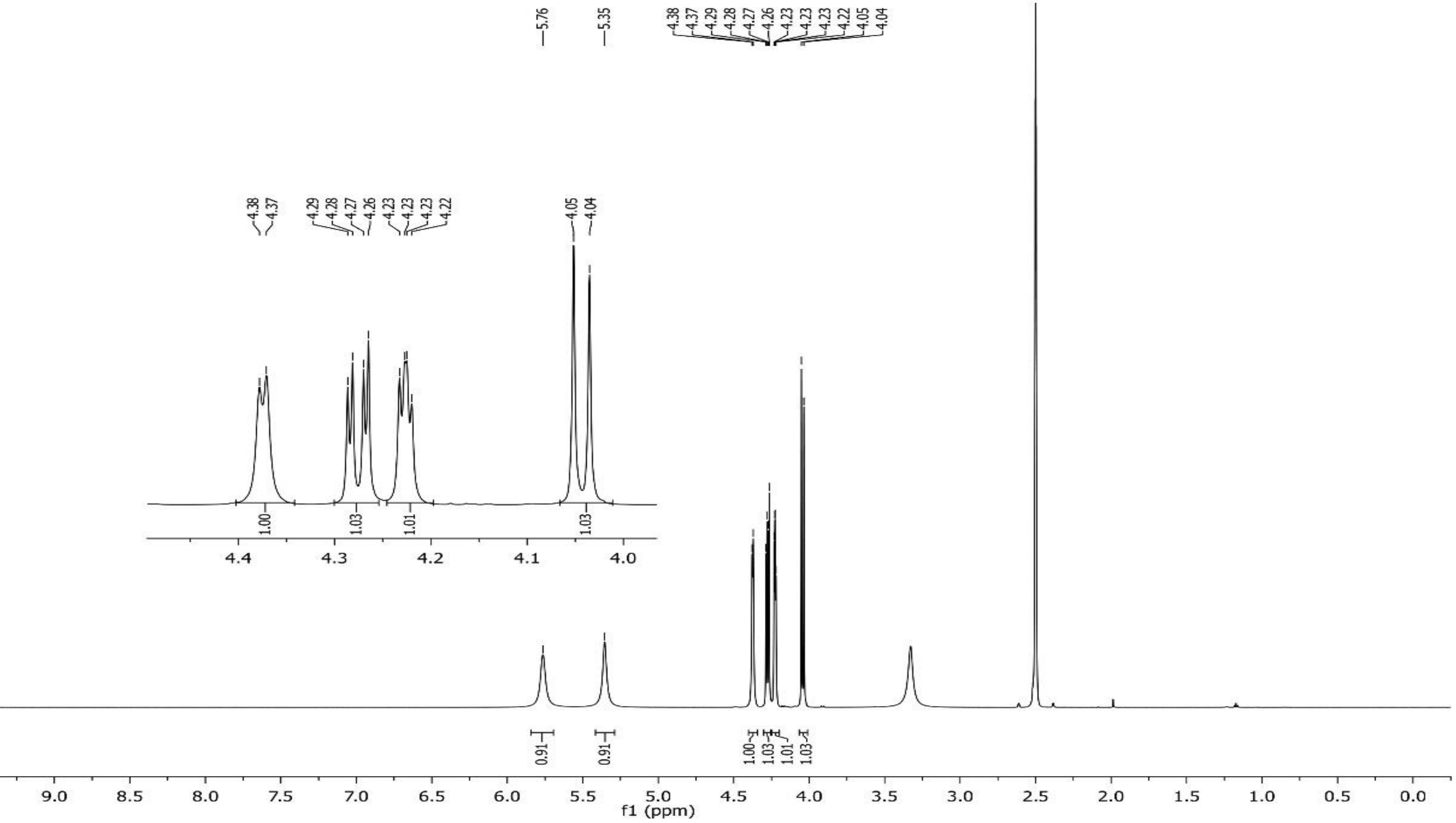

^{13}C NMR (151 MHz, d_6-DMSO) of D-*erythrono*-1,4-lactone (10)

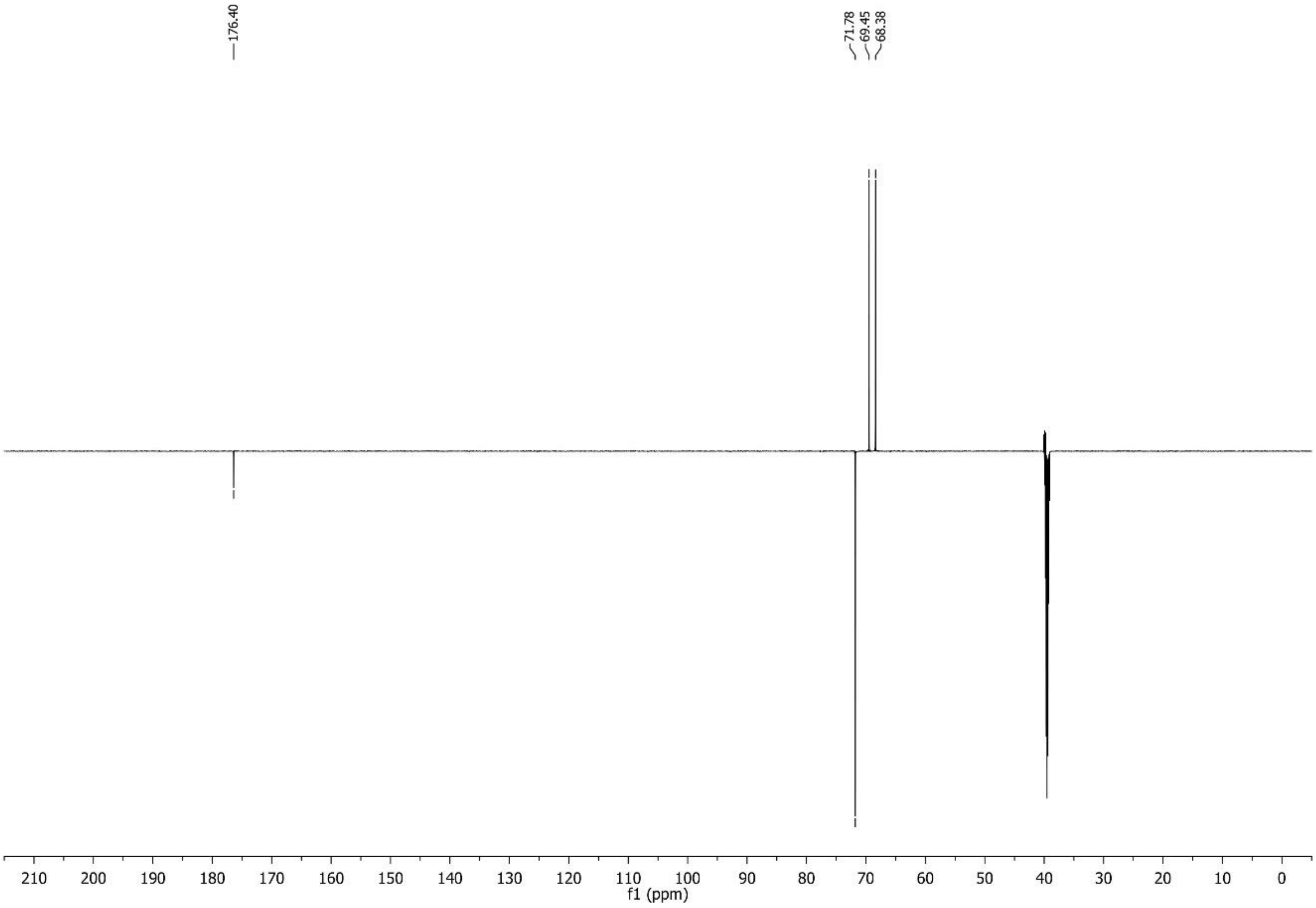

^{1}H NMR (600 MHz, CDCl3) 2,3-*O*-cyclohexylidene-D-*erythrono*-1,4-lactone (11)

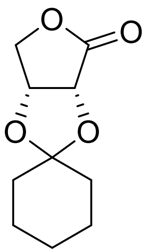

11
$C_{10}H_{14}O_4$
198.22 g/mol

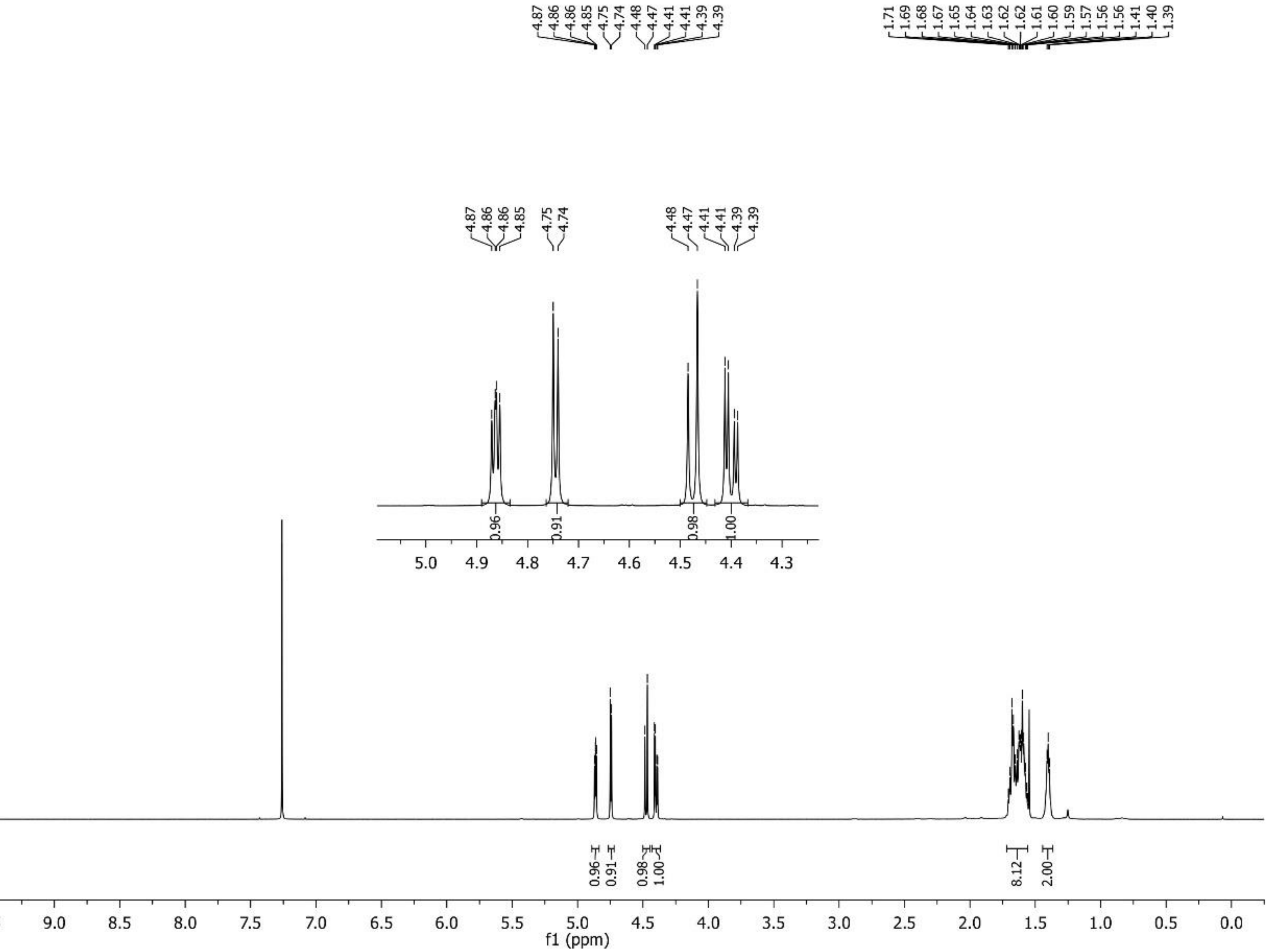

^{13}C NMR (151 MHz, CDCl3) of 2,3-*O*-cyclohexylidene-D-*erythrono*-1,4-lactone (11)

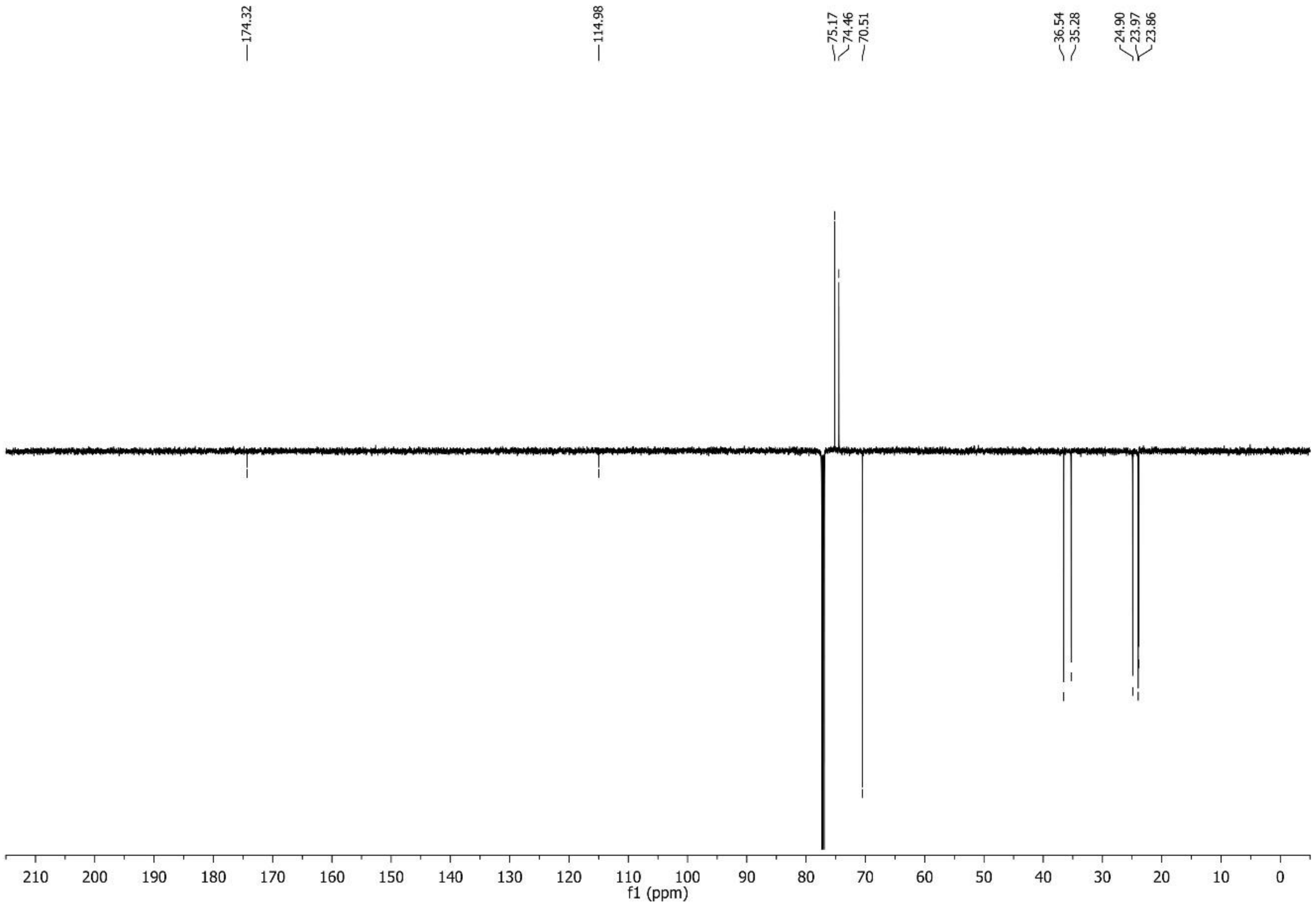

^{1}H NMR (600 MHz, $CDCl_3$) of 2,5-anhydro-3,4-*O*-cyclohexylidene-1-deoxy-D-*erythrono*-pent-1-enitol (12)

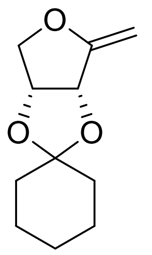

12
$C_{11}H_{16}O_3$
196.25 g/mol

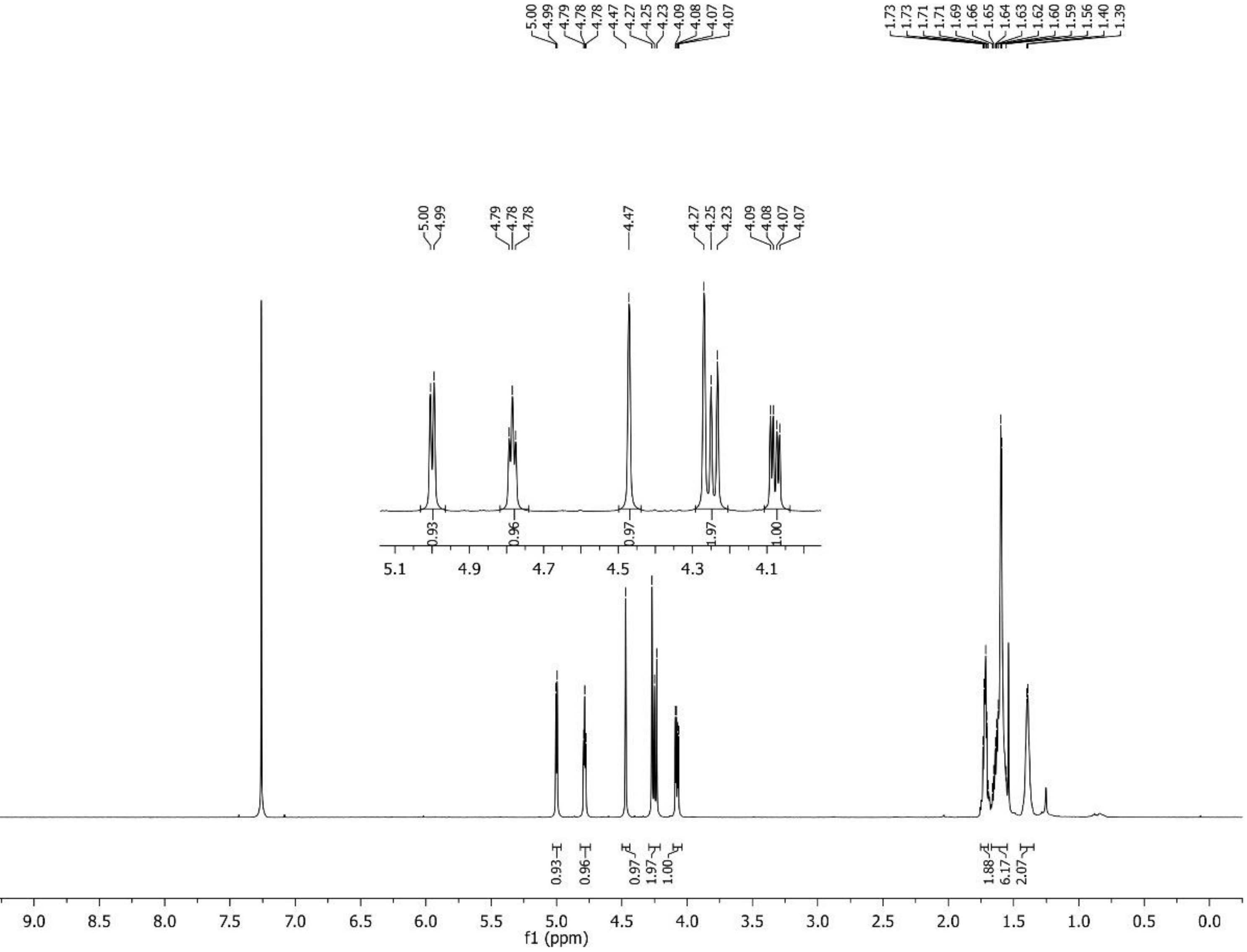

^{13}C NMR (151 MHz, $CDCl_3$) of 2,5-anhydro-3,4-*O*-cyclohexylidene-1-deoxy-D-*erythrono*-pent-1-enitol (12)

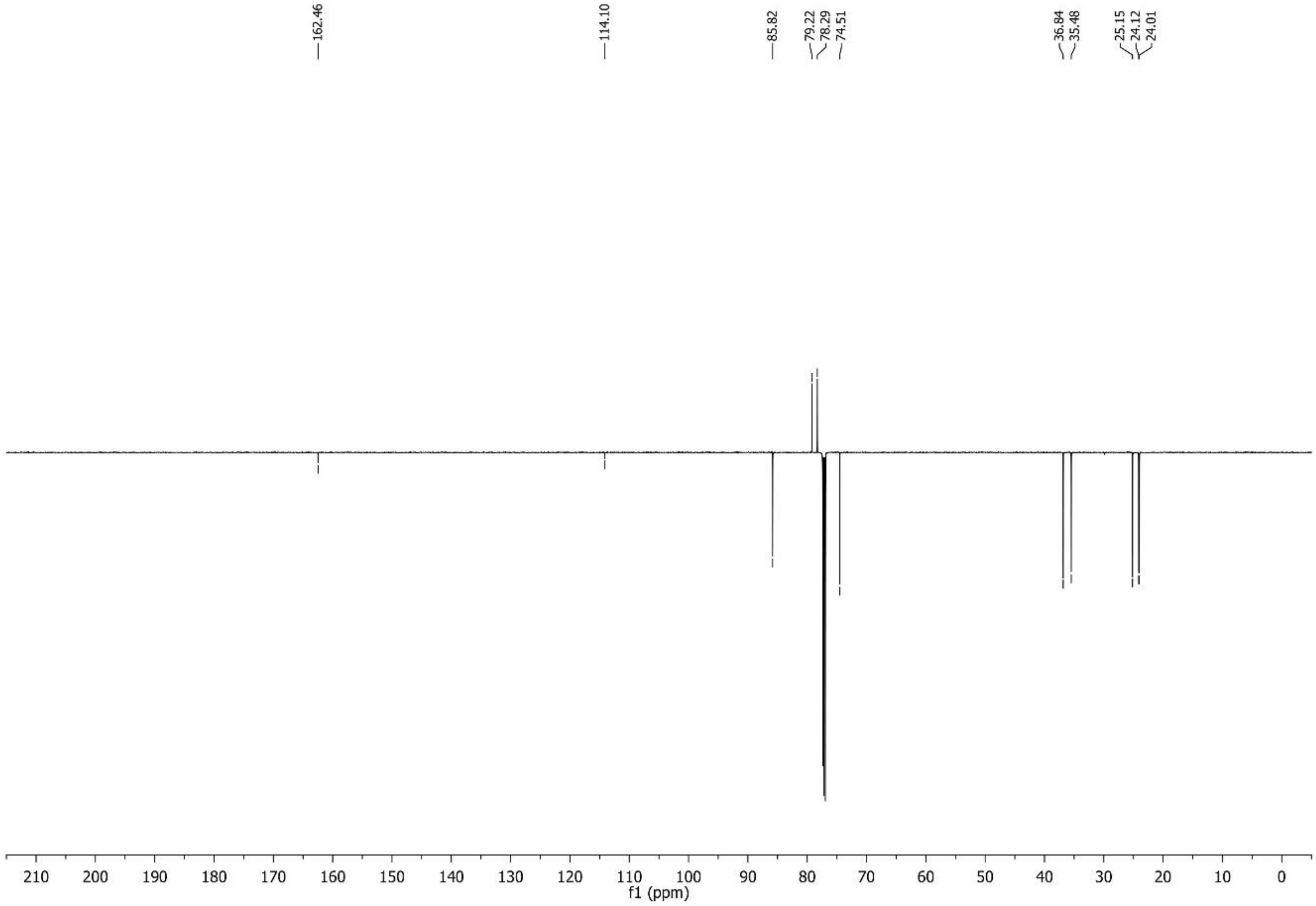

^{1}H NMR (700 MHz, $CDCl_3$) of 3,4-*O*-cyclohexylidene-1-deoxy-1-fluoro-D-ribulose (13)

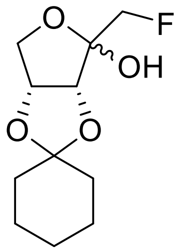

13

$C_{11}H_{17}FO_4$

232.25 g/mol

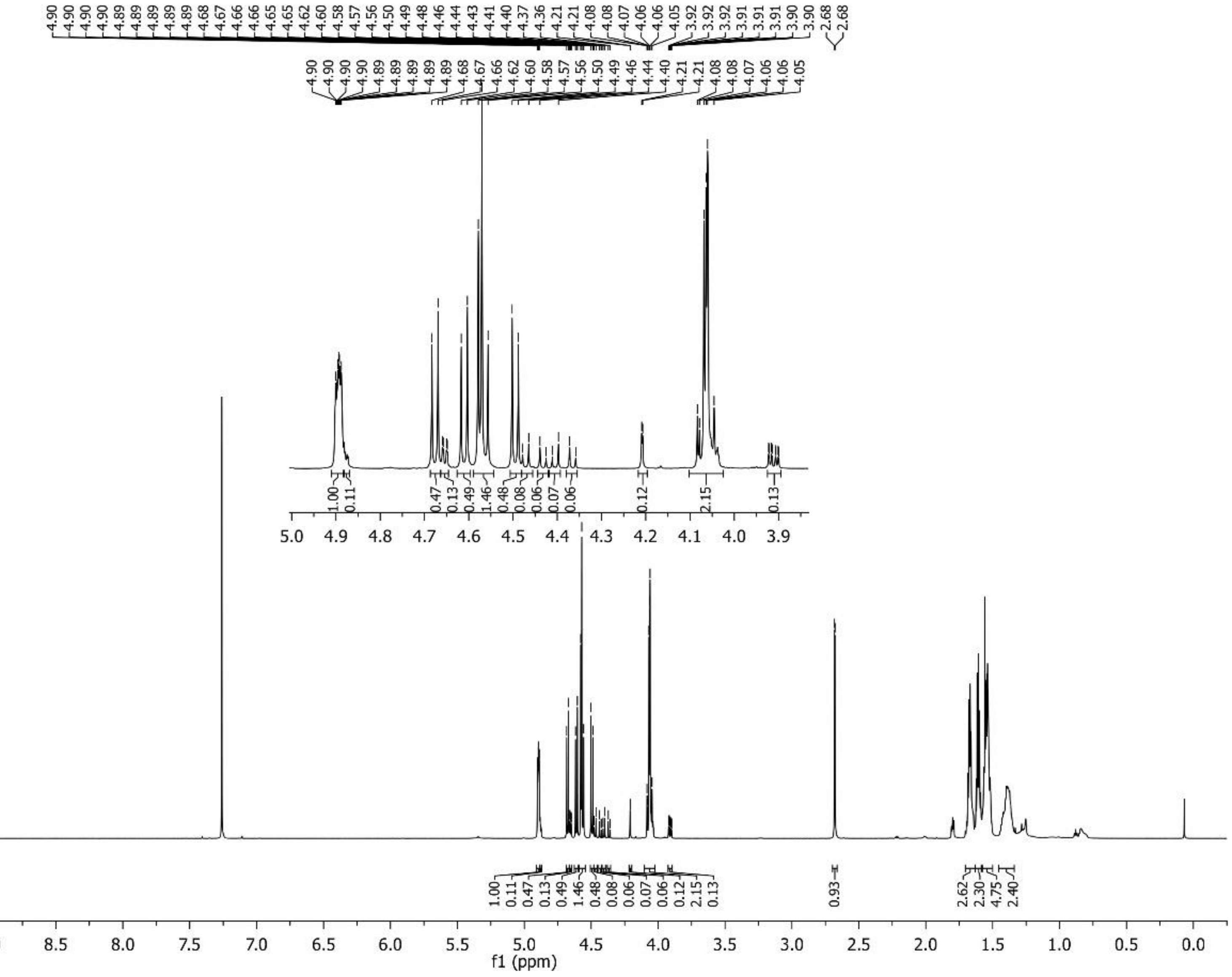

^{13}C NMR (176 MHz, $CDCl_3$) 3,4-*O*-cyclohexylidene-1-deoxy-1-fluoro-D-ribulose (13)

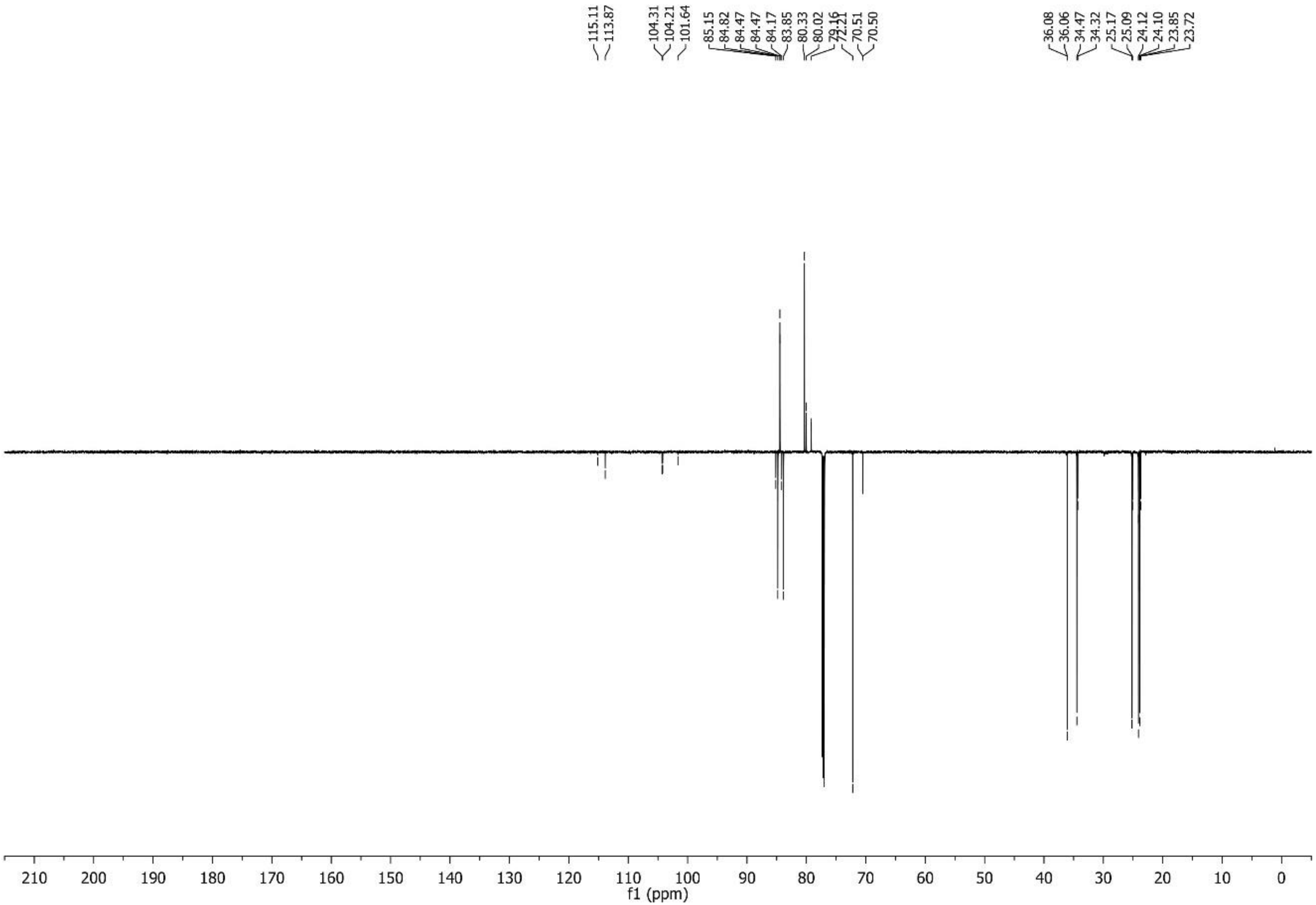

^{19}F NMR (659 MHz, $CDCl_3$) of 3,4-*O*-cyclohexylidene-1-deoxy-1-fluoro-D-ribulose (13)

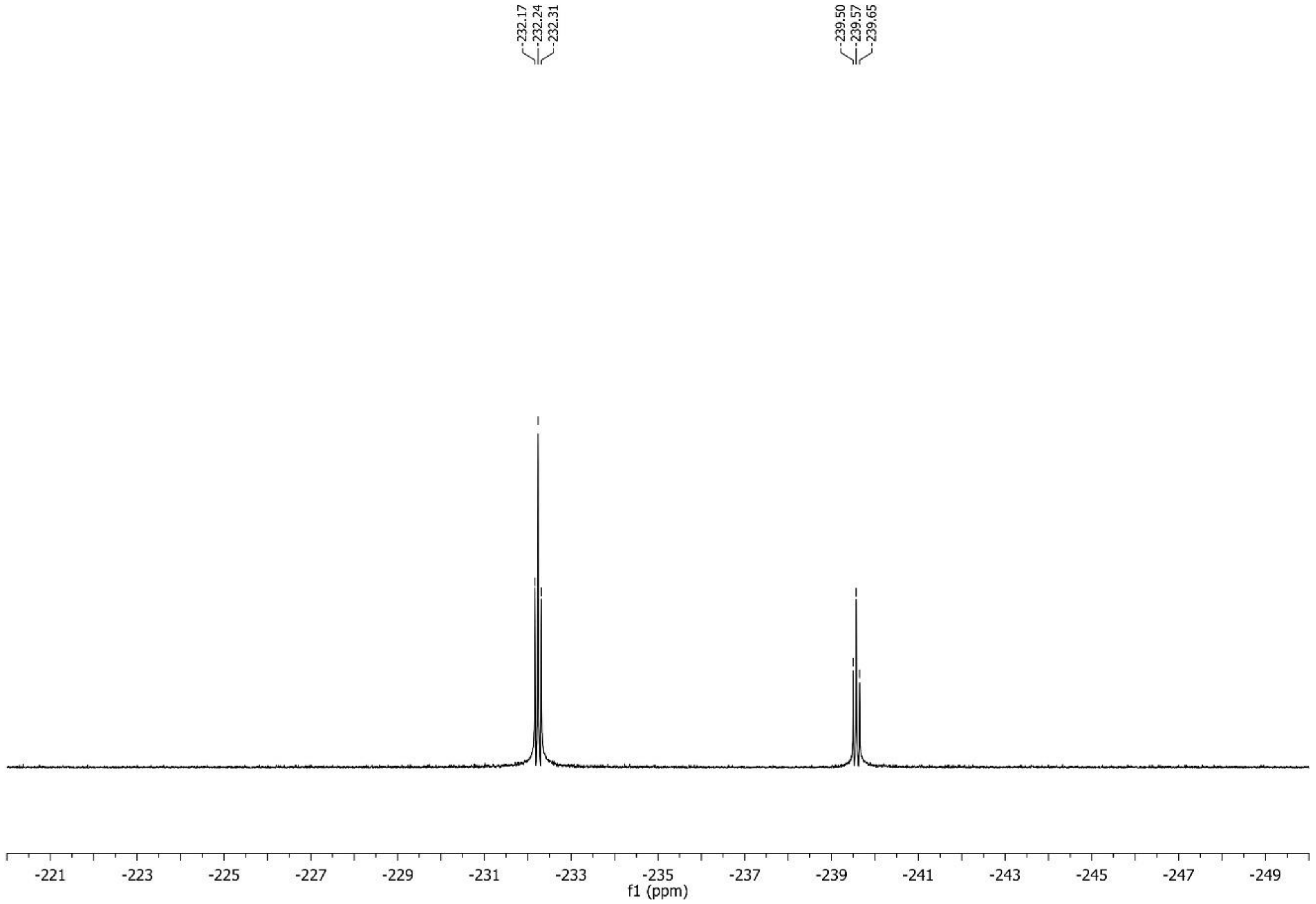

^{1}H NMR (700 MHz, D_2O) of 1-deoxy-1-fluoro-D-ribulose (1DFRu, 3)

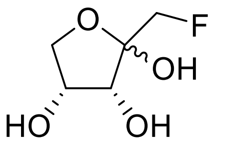

3, 1DFRu

$C_5H_9FO_4$

152.12 g/mol

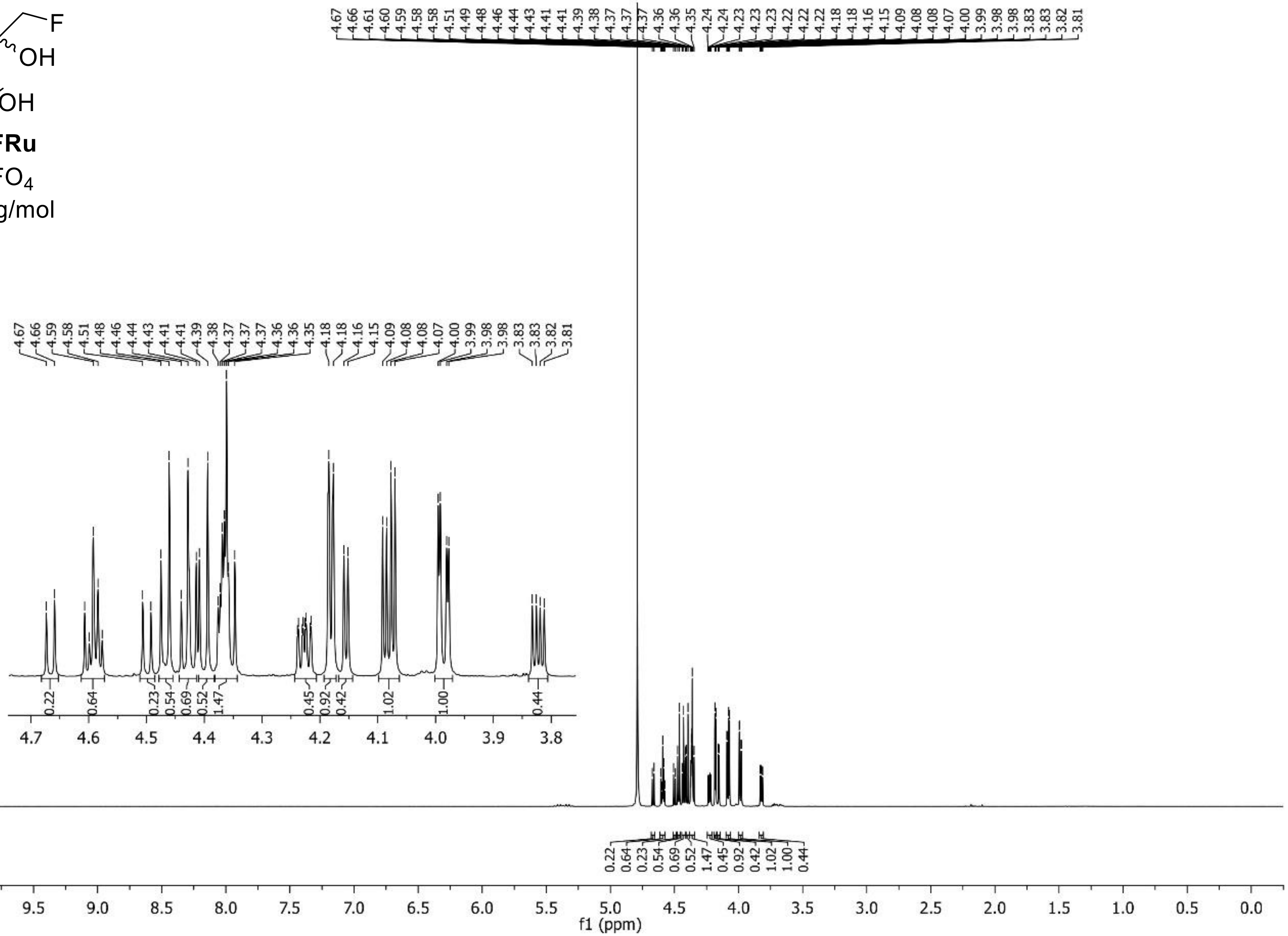

^{13}C NMR (176 MHz, D_2O) of 1-deoxy-1-fluoro-D-ribulose (1DFRu, 3)

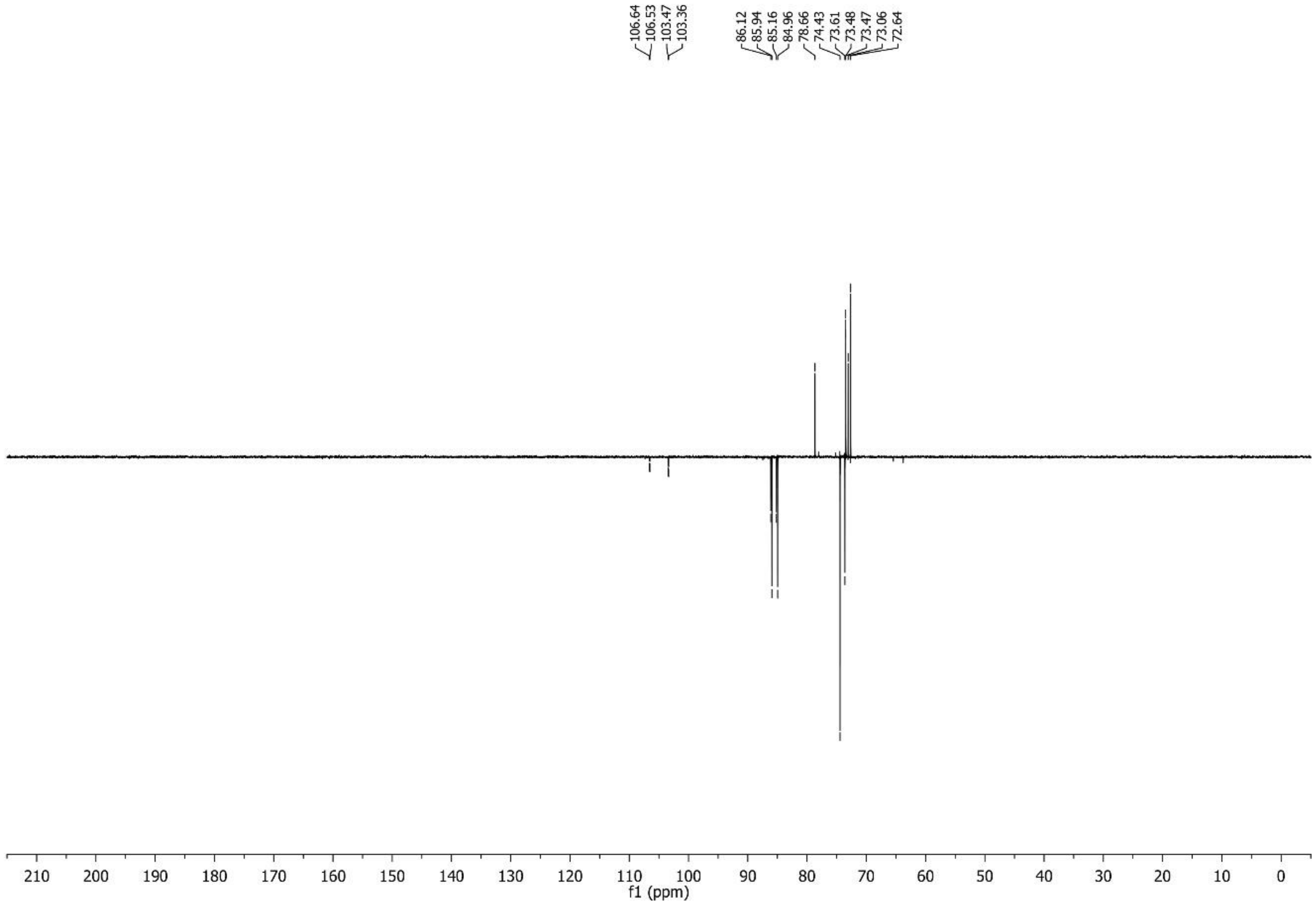

^{19}F NMR (659 MHz, D_2O) of 1-deoxy-1-fluoro-D-ribulose (1DFRu, 3)

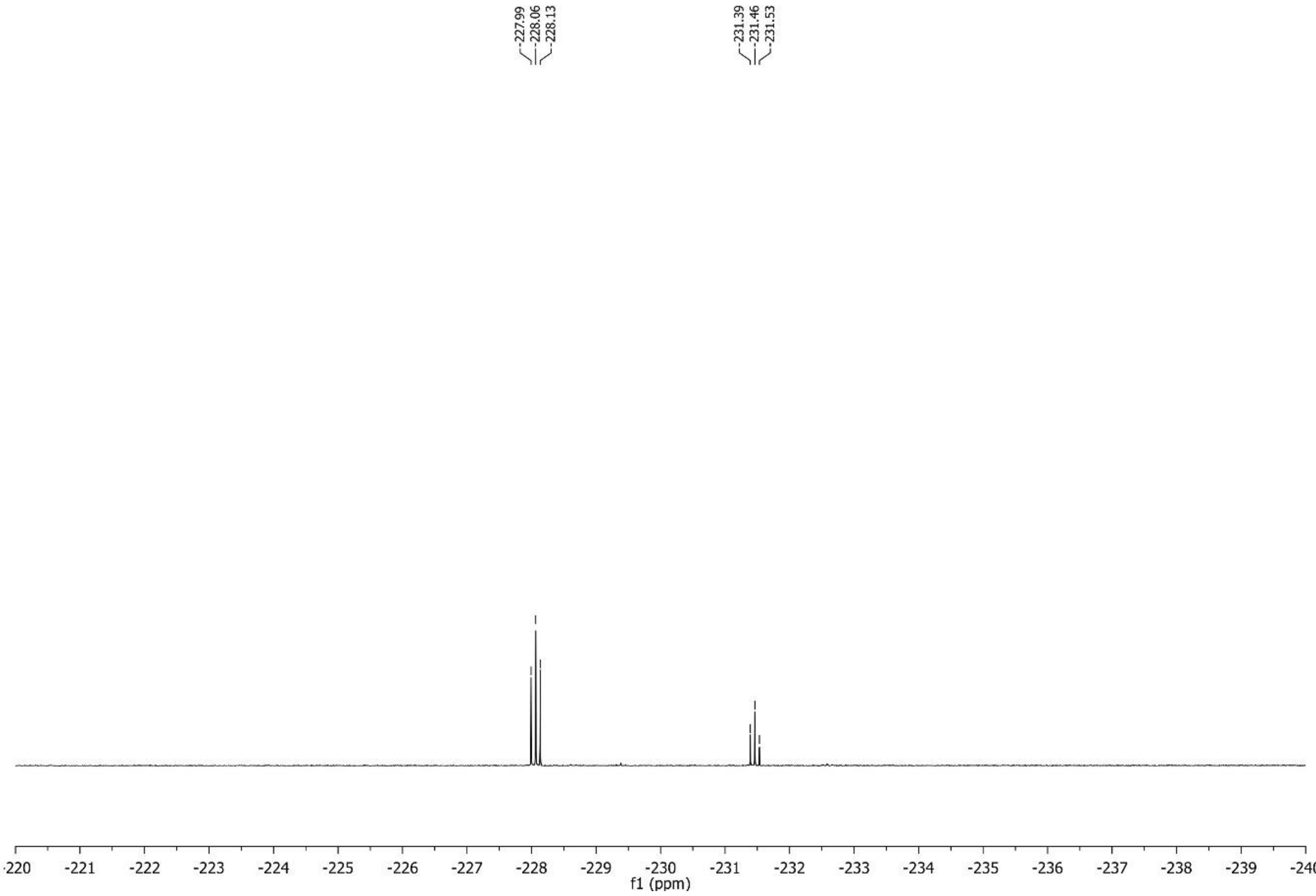

^{1}H NMR (700 MHz, $CDCl_3$) of methyl 3,4-*O*-cyclohexylidene-D-ribulofuranoside (14)

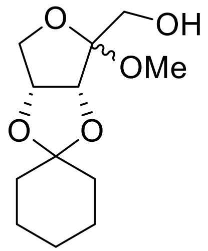

14

$C_{12}H_{20}O_5$

244.29 g/mol

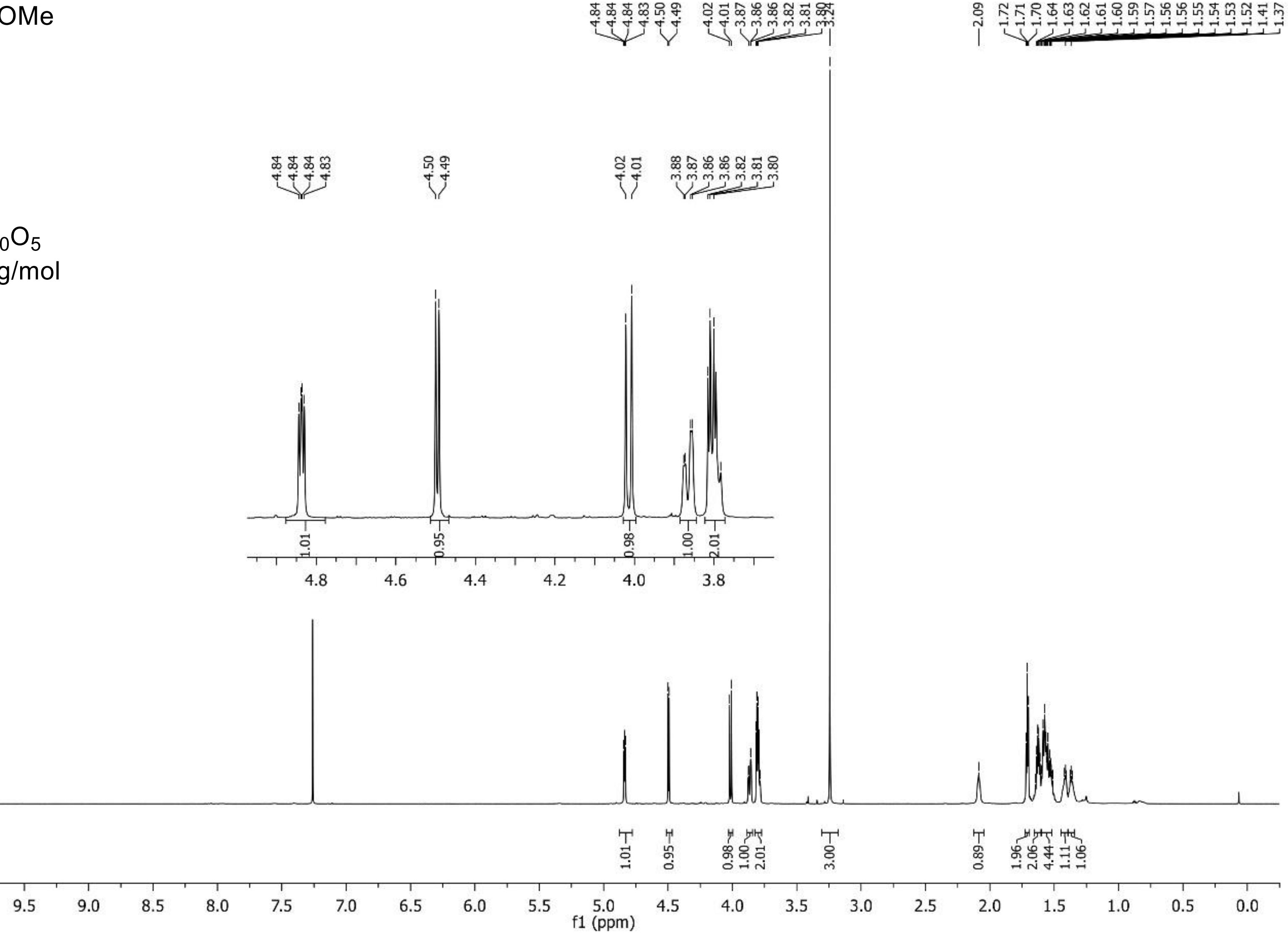

^{13}C NMR (176 MHz, $CDCl_3$) of methyl 3,4-*O*-cyclohexylidene-D-ribulofuranoside (14)

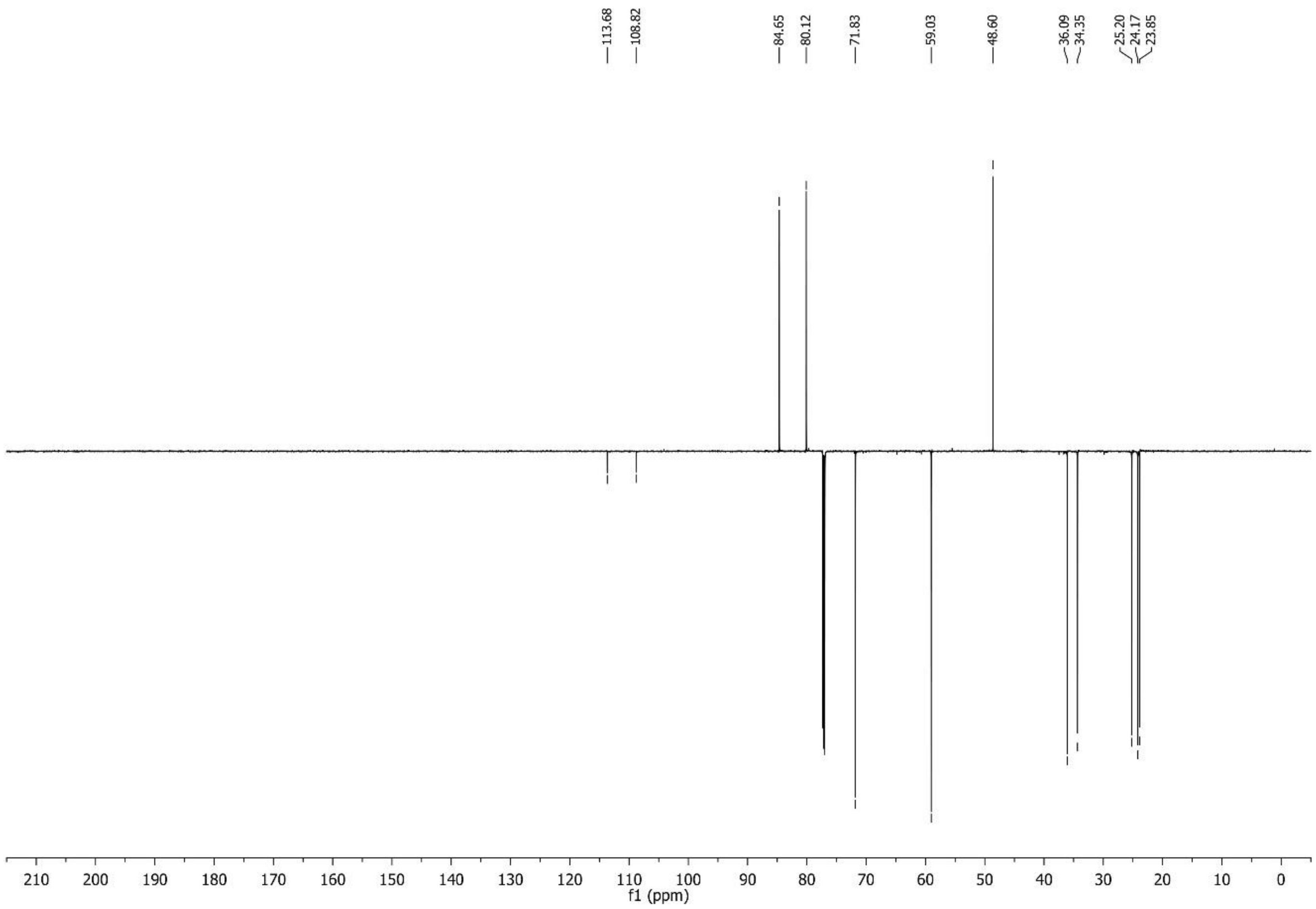

^{1}H NMR (600 MHz, CDCl$_3$) of methyl 3,4-*O*-cyclohexylidene-1-*O*-trifluoromethanesulfonyl-D-ribulofuranoside (15)

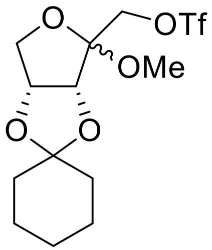

15

$C_{13}H_{19}F_3O_7S$

376.34 g/mol

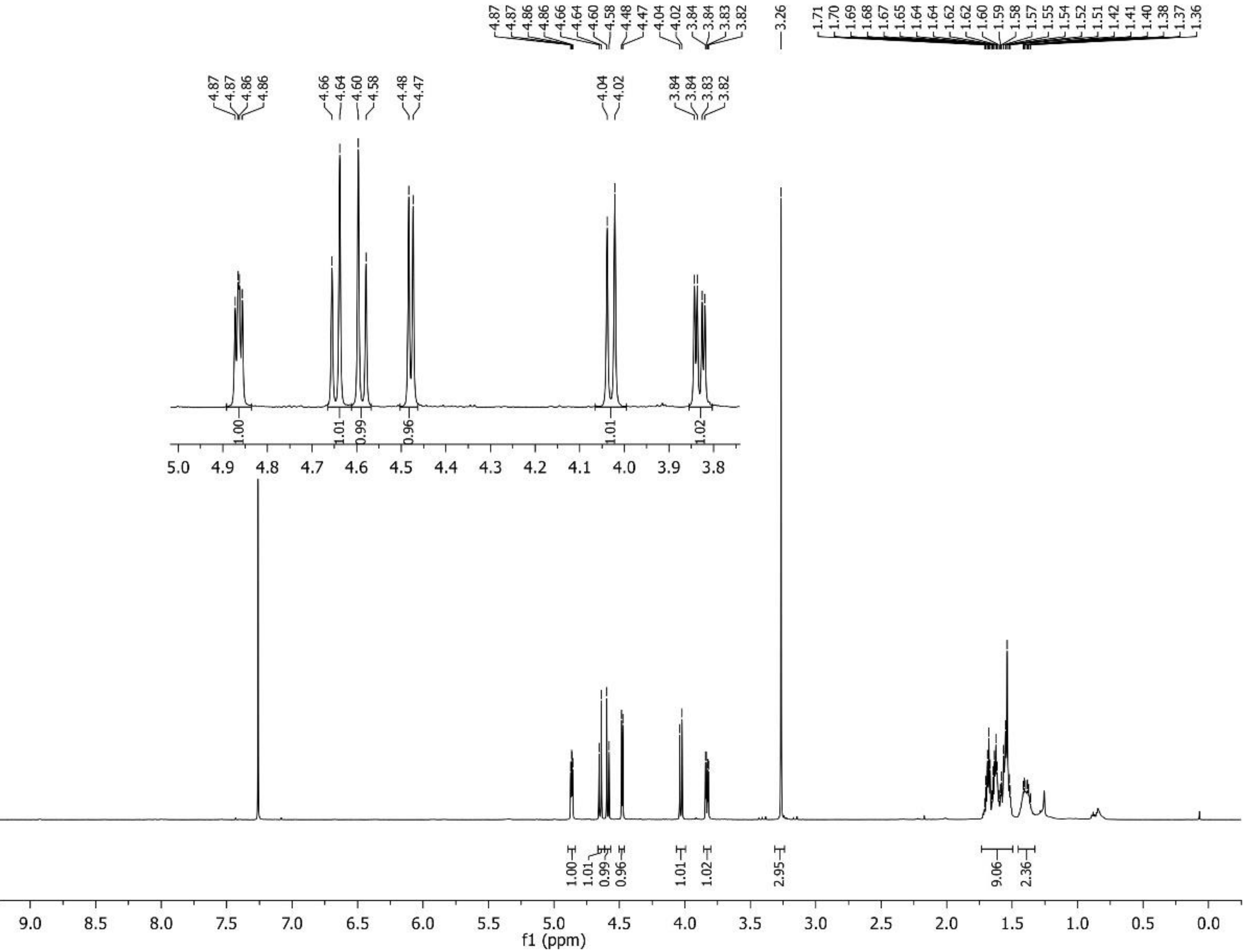

^{13}C NMR (151 MHz, $CDCl_3$) of methyl 3,4-*O*-cyclohexylidene-1-*O*-trifluoromethanesulfonyl-D-ribulofuranoside (15)

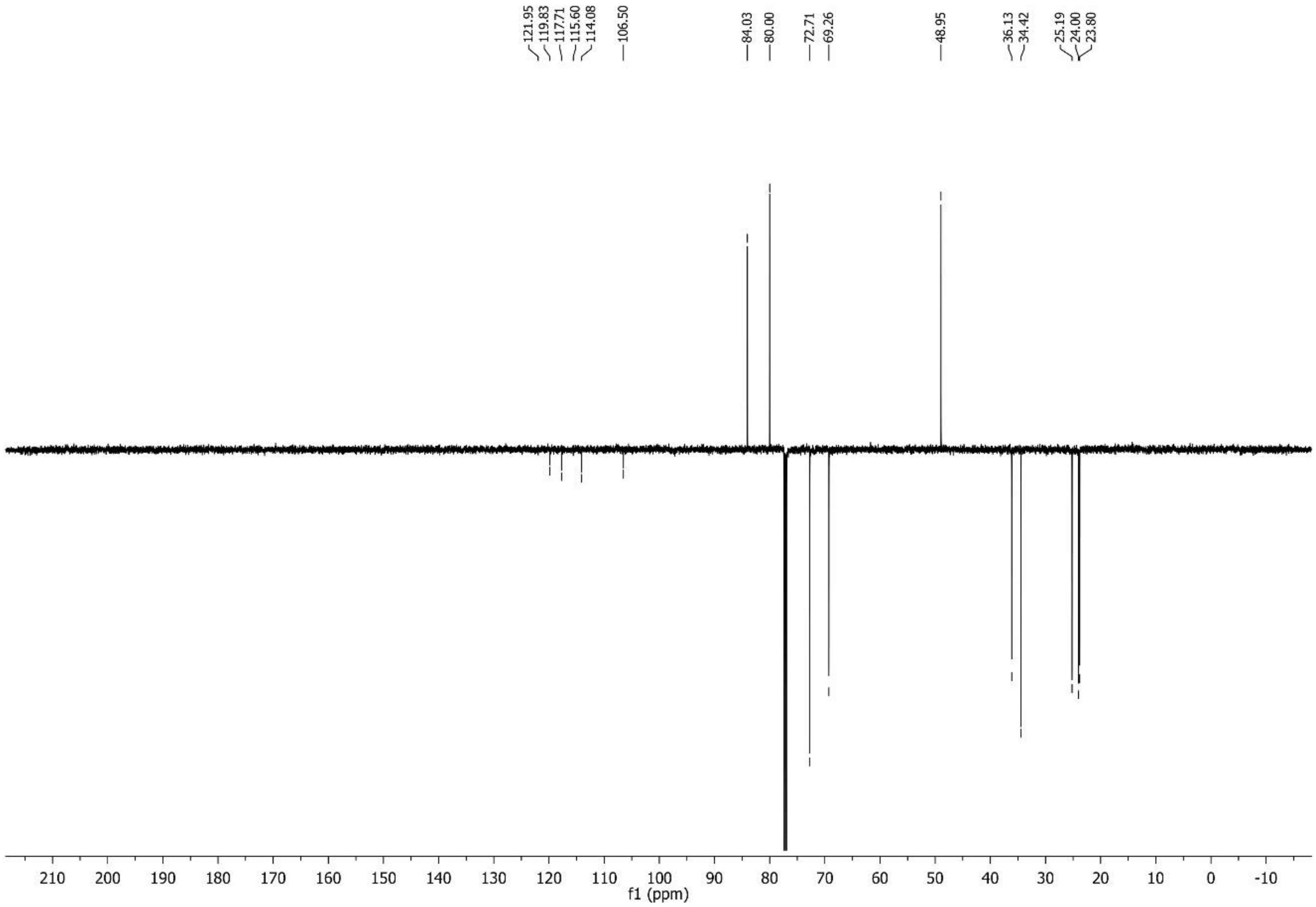

^{19}F NMR (659 MHz, $CDCl_3$) of methyl 3,4-*O*-cyclohexylidene-1-*O*-trifluoromethanesulfonyl-D-ribulofuranoside (15)

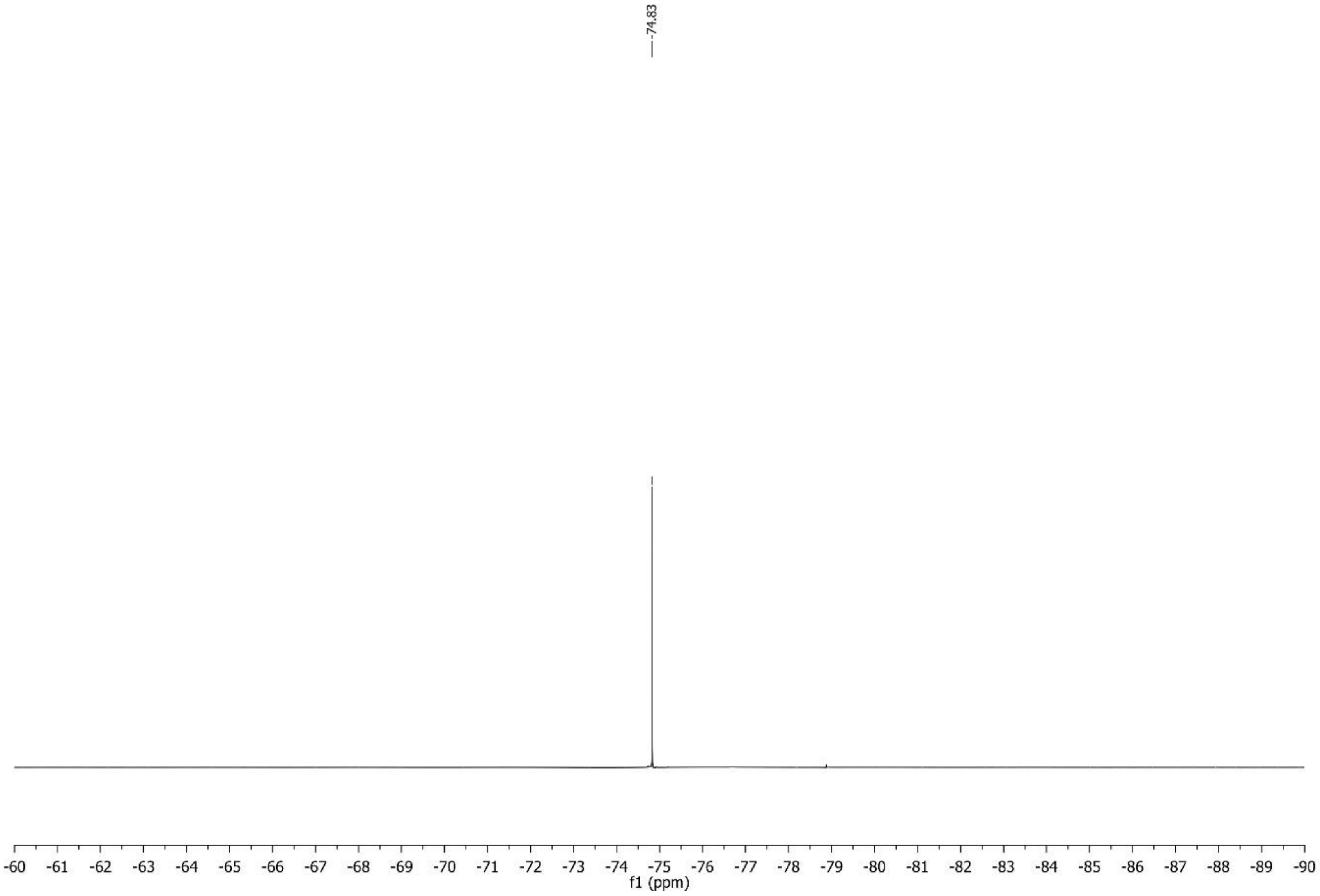

^{1}H NMR (700 MHz, CDCl₃) of 3-*O*-Formyl-1,2-*O*-isopropylidene-β-D-threose (17)

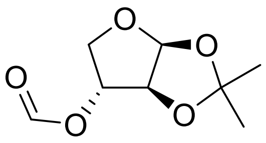

17

$C_8H_{12}O_5$

188.18 g/mol

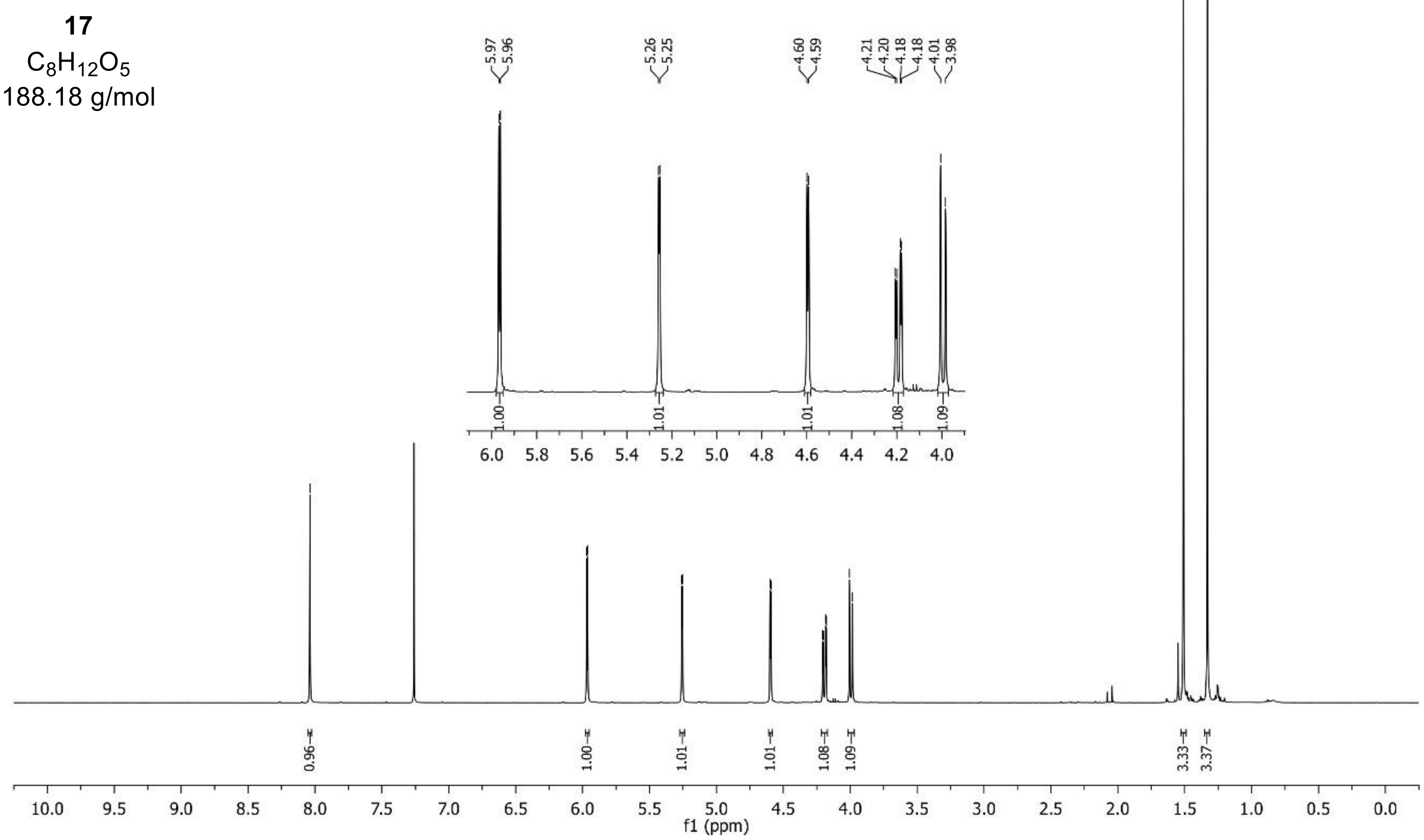

^{13}C NMR (176 MHz, $CDCl_3$) of 3-*O*-Formyl-1,2-*O*-isopropylidene-β-D-threose (17)

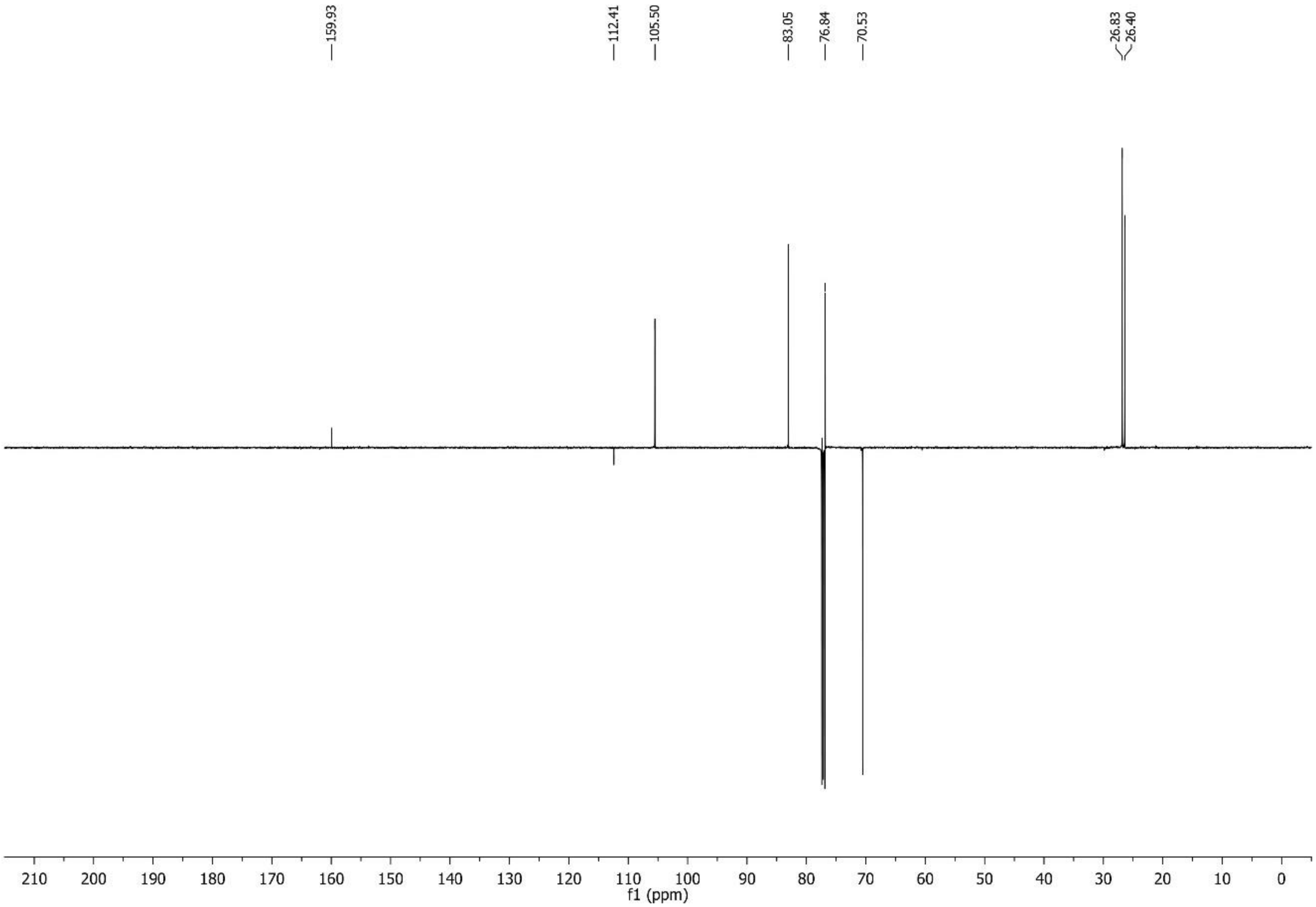

^{1}H NMR (500 MHz, $CDCl_3$) of 1,2-*O*-isopropylidene-β-D-threose (18)

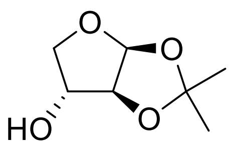

18

$C_7H_{12}O_4$

160.17 g/mol

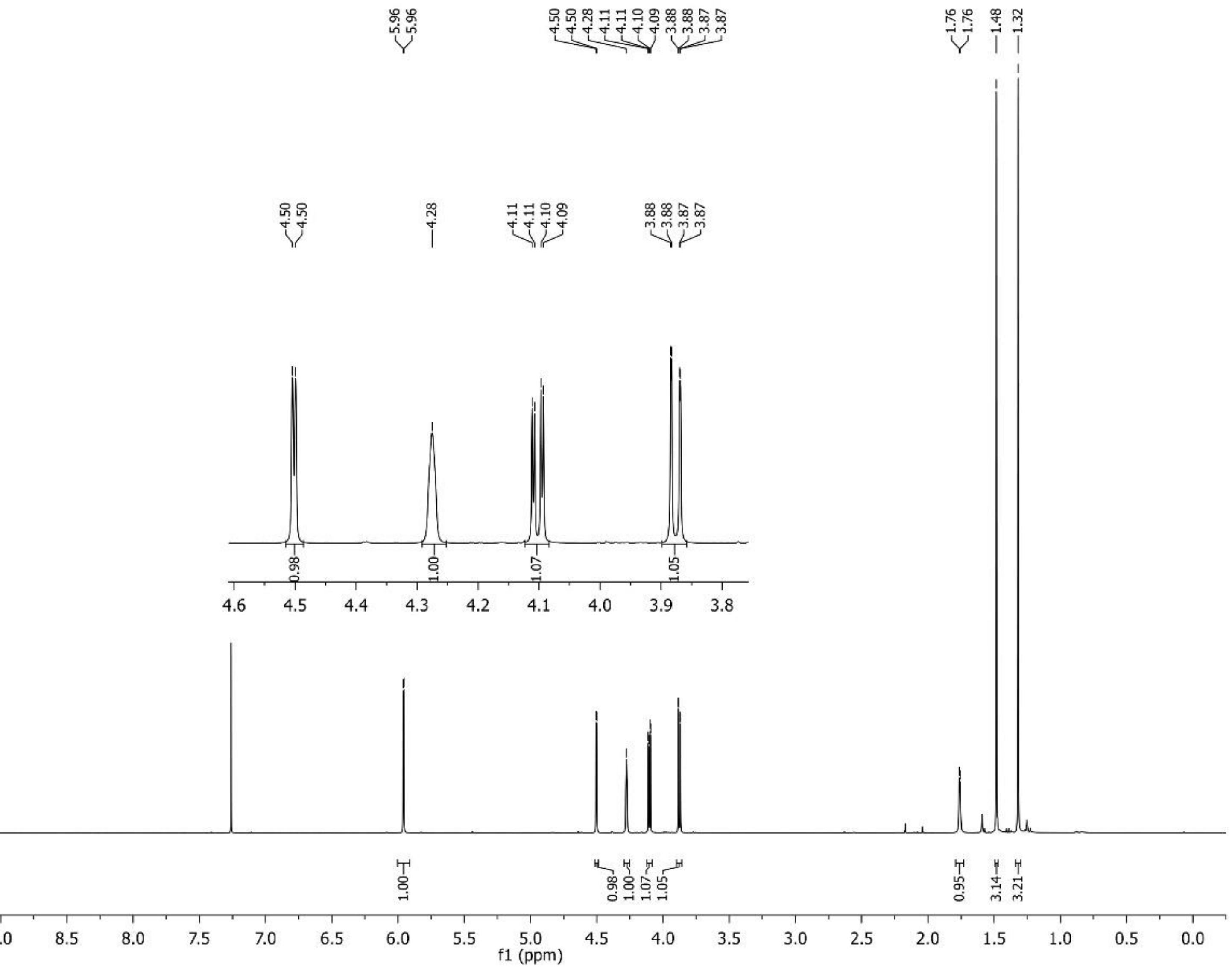

^{13}C NMR (126 MHz, $CDCl_3$) of 1,2-*O*-isopropylidene-β-D-threose (18)

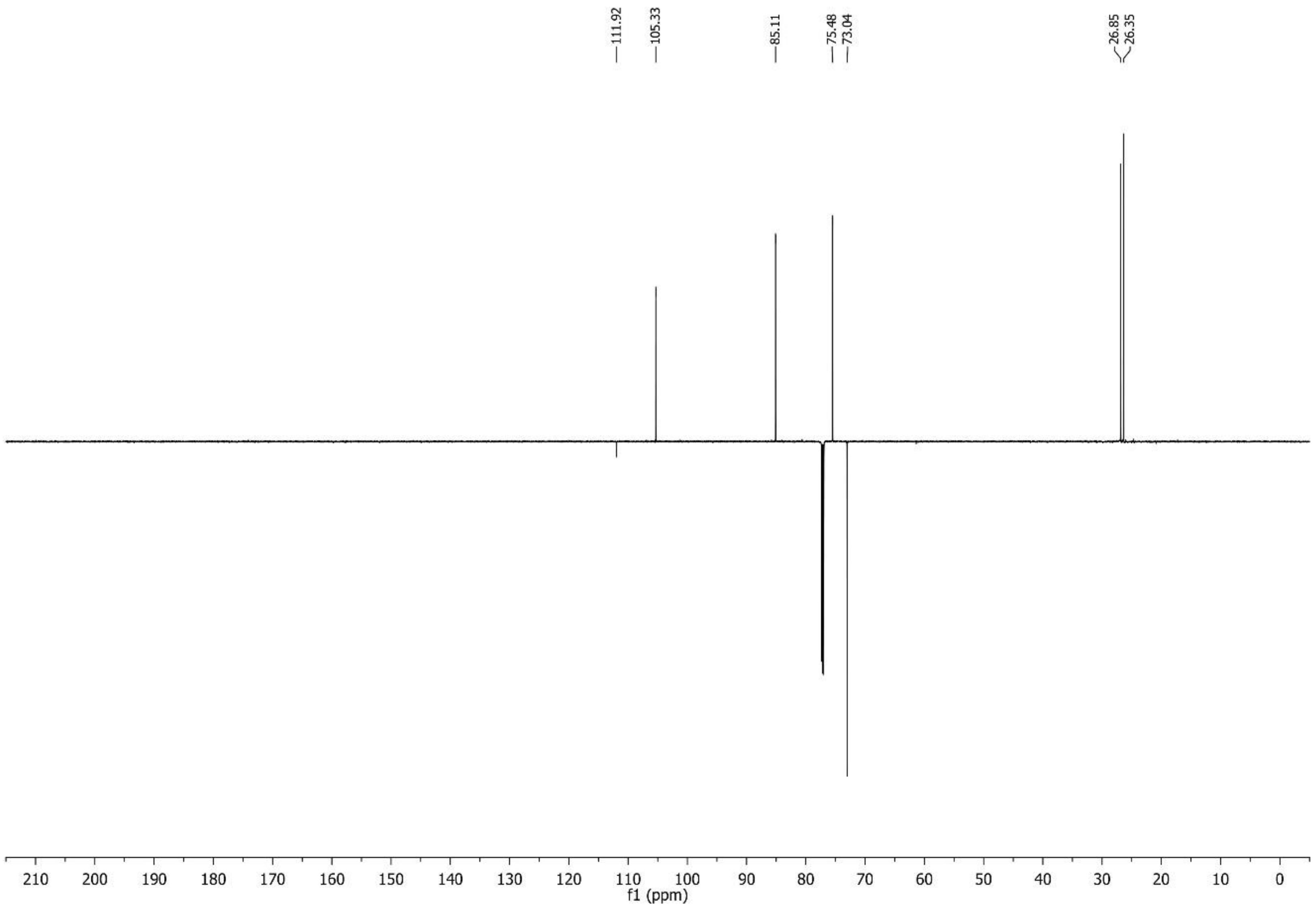

^{1}H NMR (700 MHz, CDCl$_3$) of 3-*O*-benzyl-1,2-*O*-isopropylidene-β-D-threose (19)

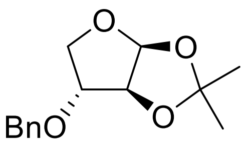

19

$C_{14}H_{18}O_4$

250.29 g/mol

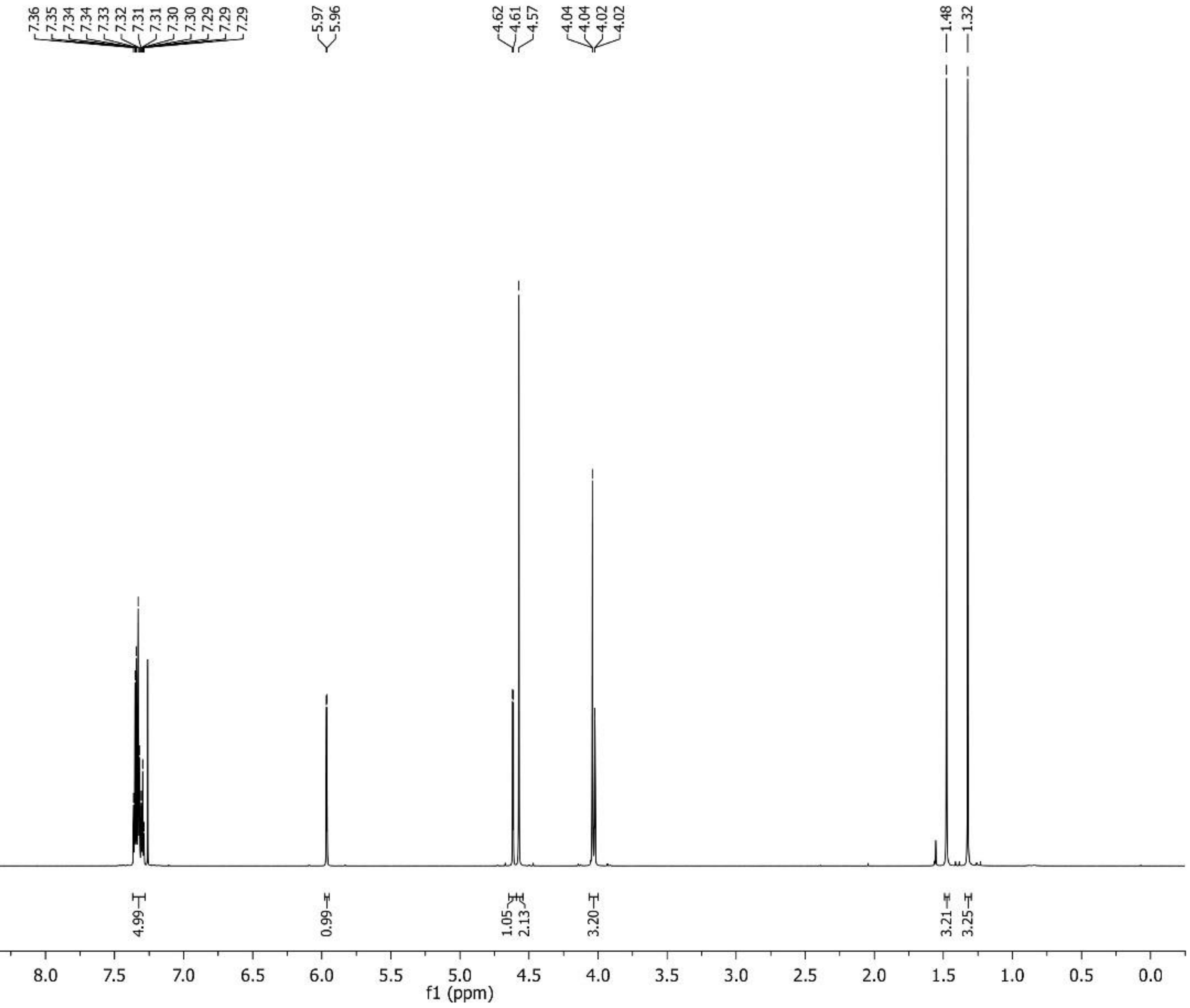

^{13}C NMR (176 MHz, $CDCl_3$) of 3-*O*-benzyl-1,2-*O*-isopropylidene-β-D-threose (19)

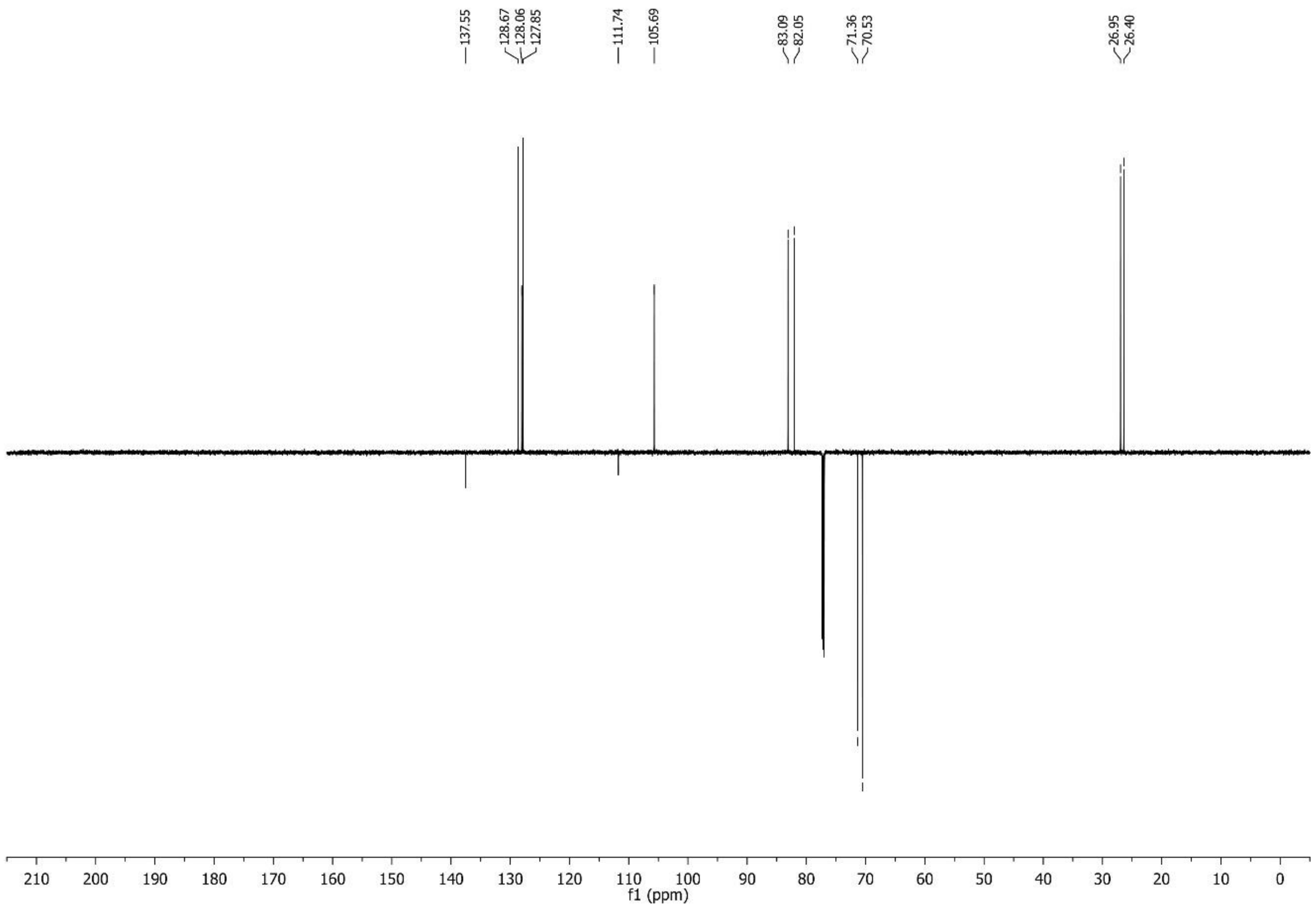

^{1}H NMR (600 MHz, CDCl₃) of methyl 3-*O*-benzyl-D-*threo*-furanoside (20)

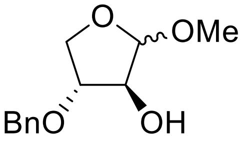

20

$C_{12}H_{16}O_4$

224.26 g/mol

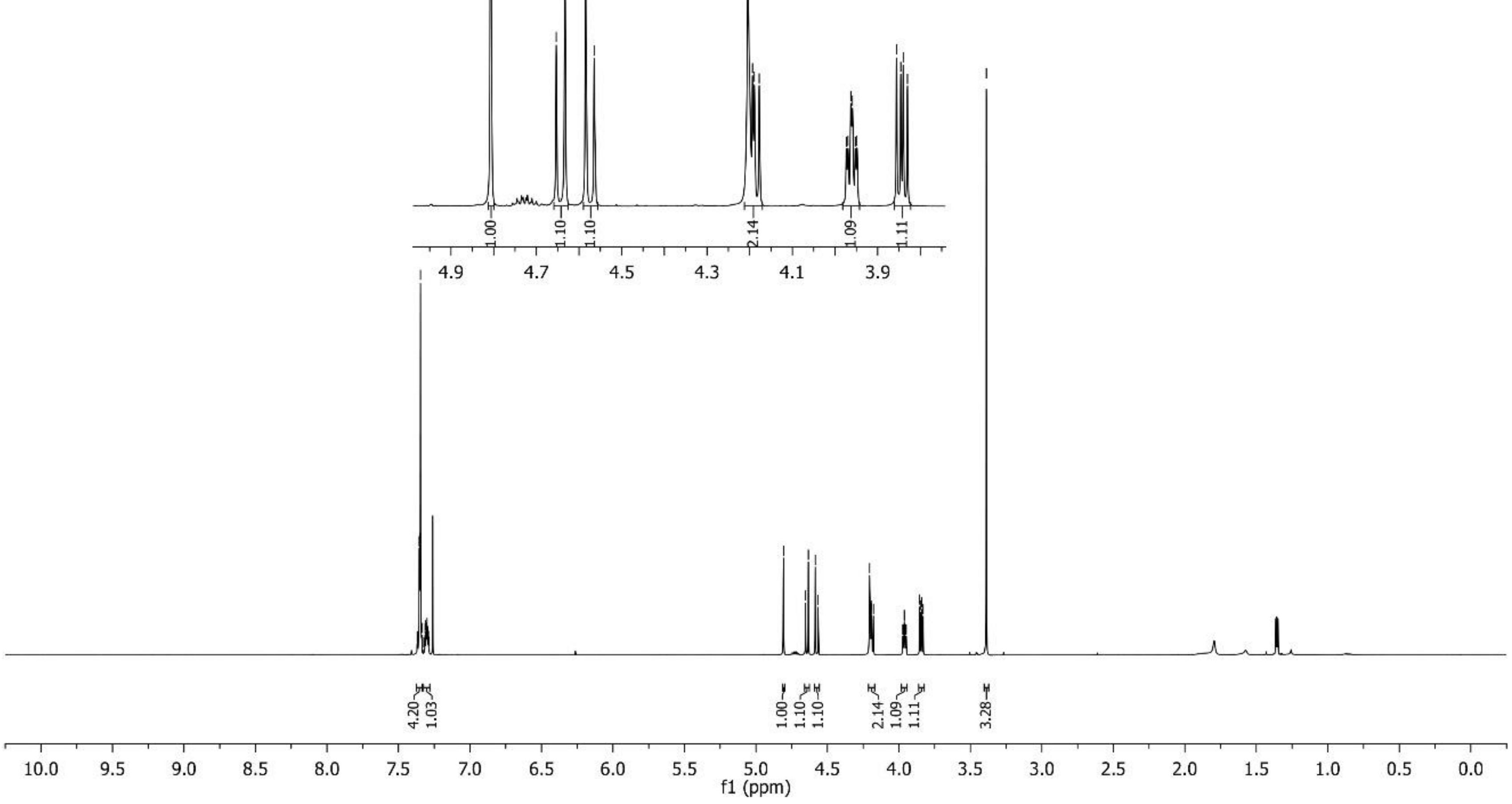

^{13}C NMR (151 MHz, CDCl$_3$) of methyl 3-*O*-benzyl-D-*threo*-furanoside (20)

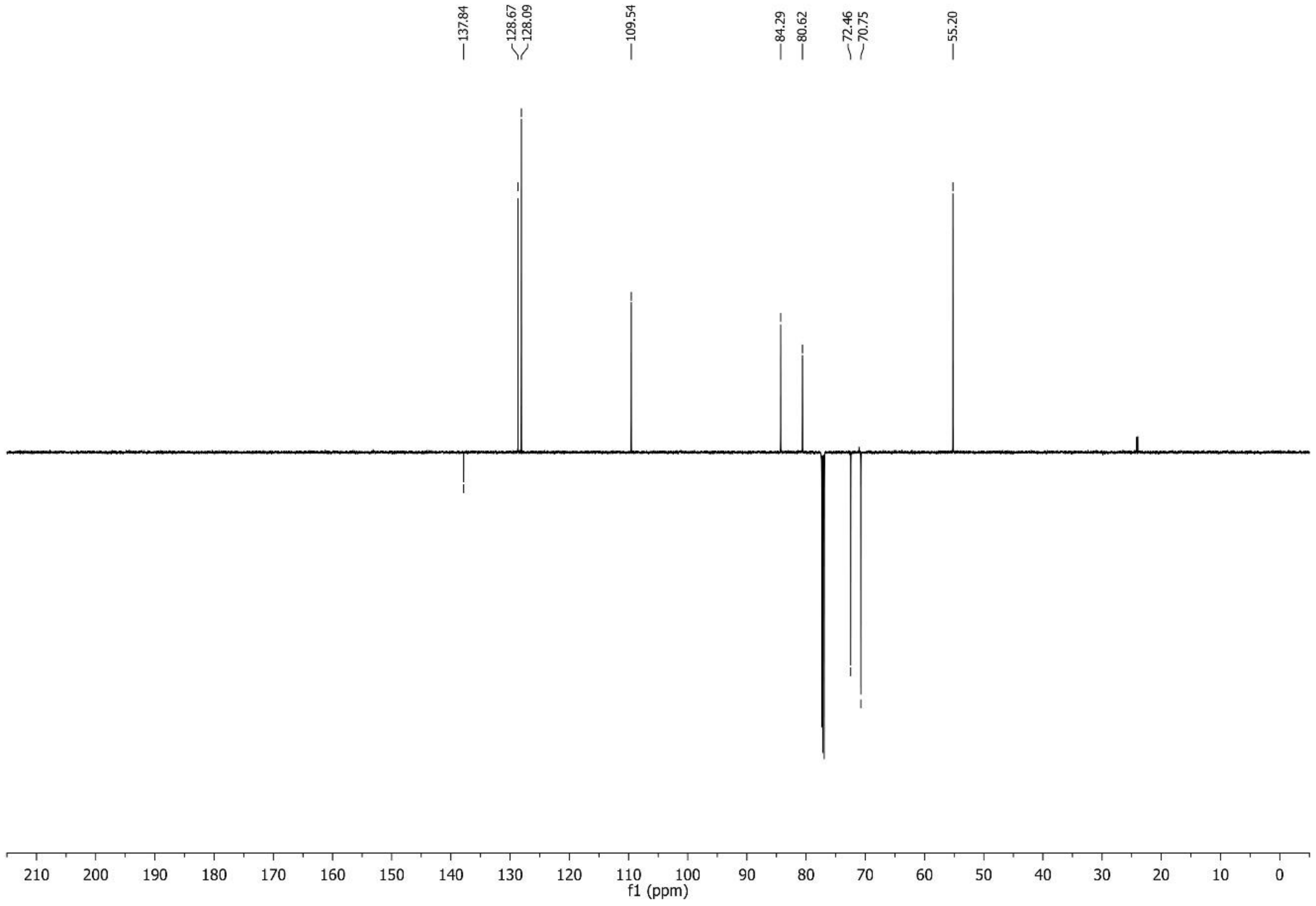

^{1}H NMR (600 MHz, $CDCl_3$) of methyl 3-*O*-benzyl-2-*O*-trifluoromethanesulfonyl-D-*threo*-furanoside (21)

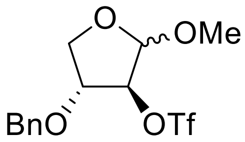

21

$C_{13}H_{15}F_3O_6S$

356.31 g/mol

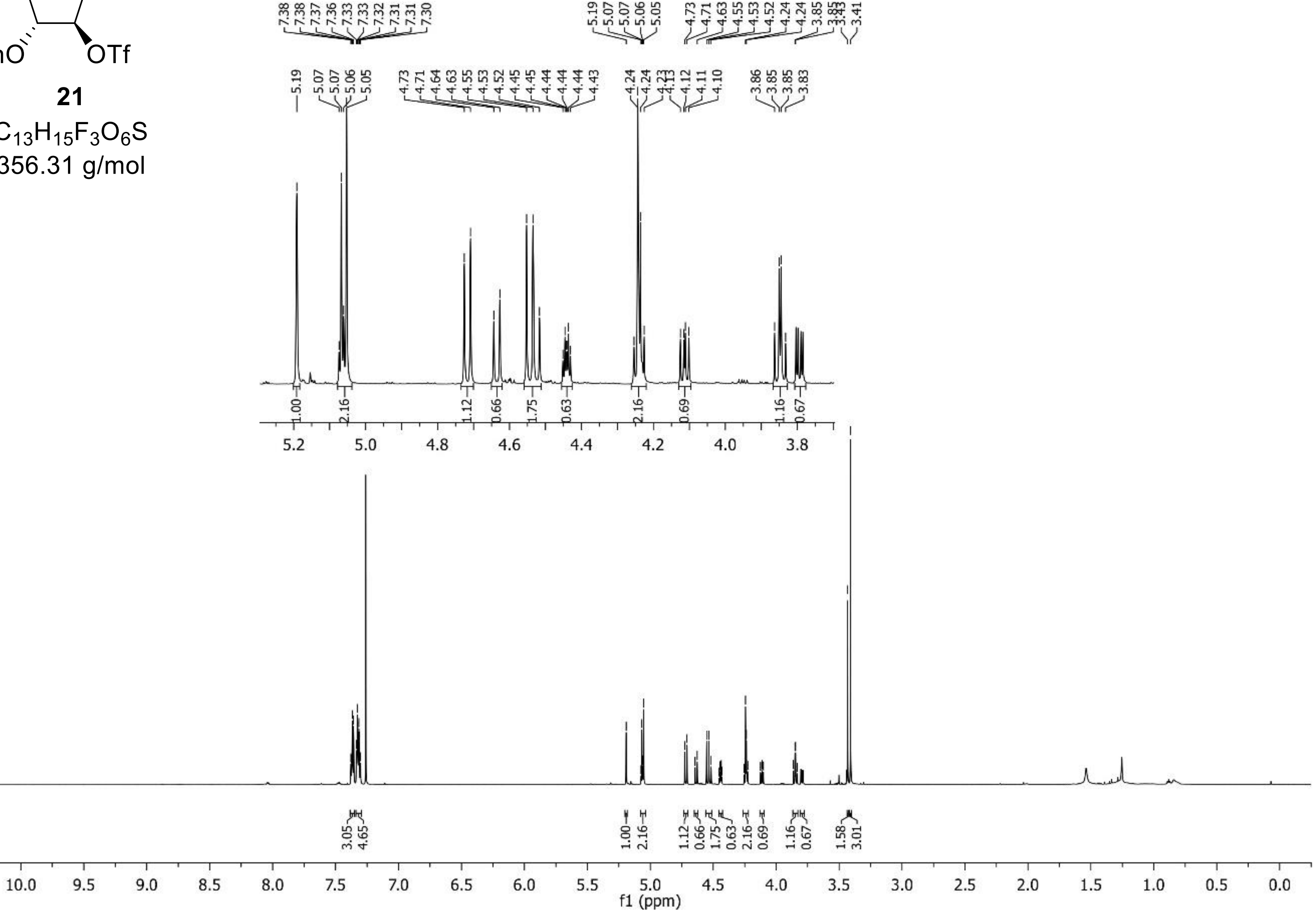

^{13}C NMR (176 MHz, CDCl$_3$) of methyl 3-*O*-benzyl-2-*O*-trifluoromethanesulfonyl-D-*threo*-furanoside (21)

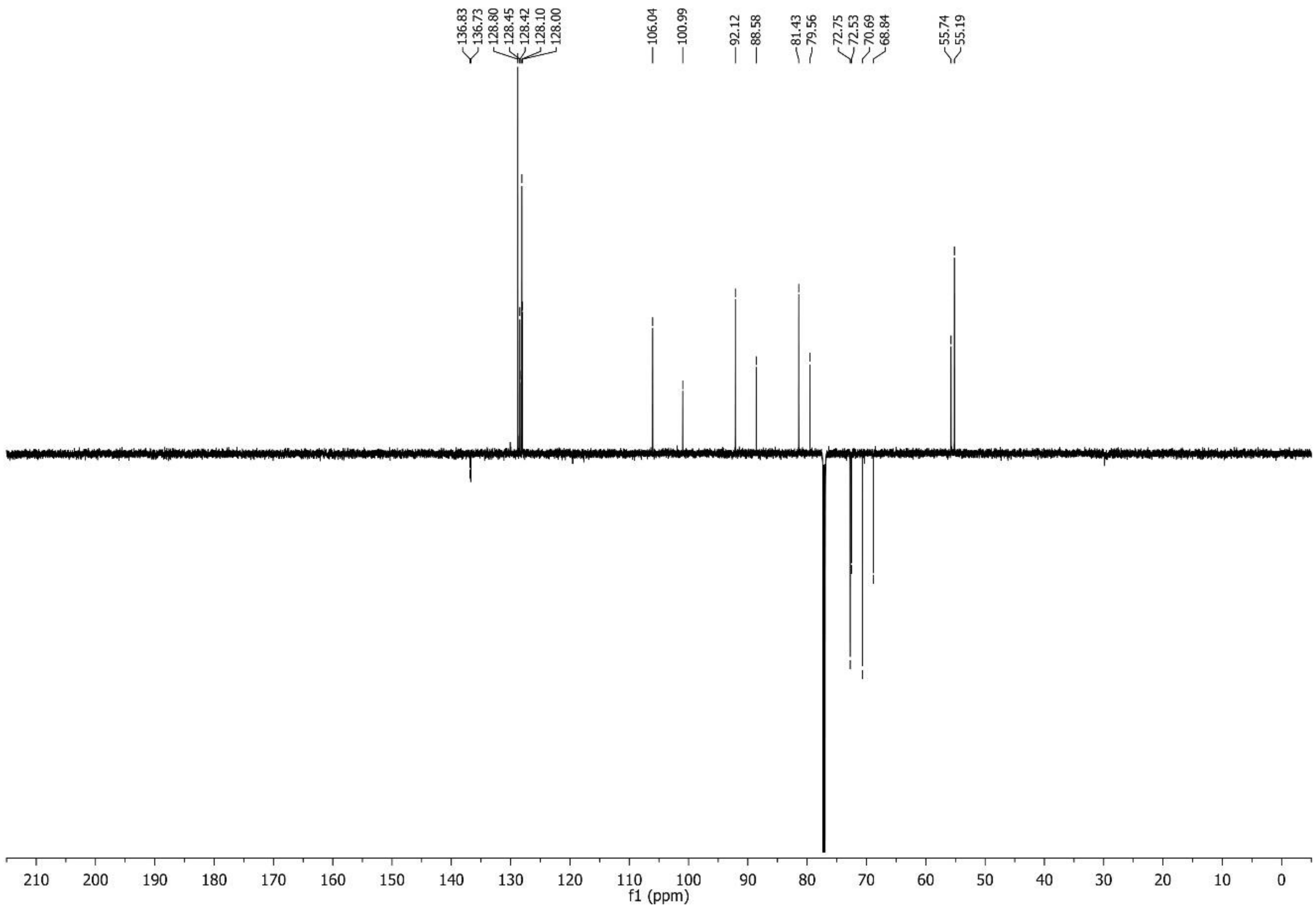

^{19}F NMR (659 MHz, $CDCl_3$) of methyl 3-*O*-benzyl-2-*O*-trifluoromethanesulfonyl-D-*threo*-furanoside (21)

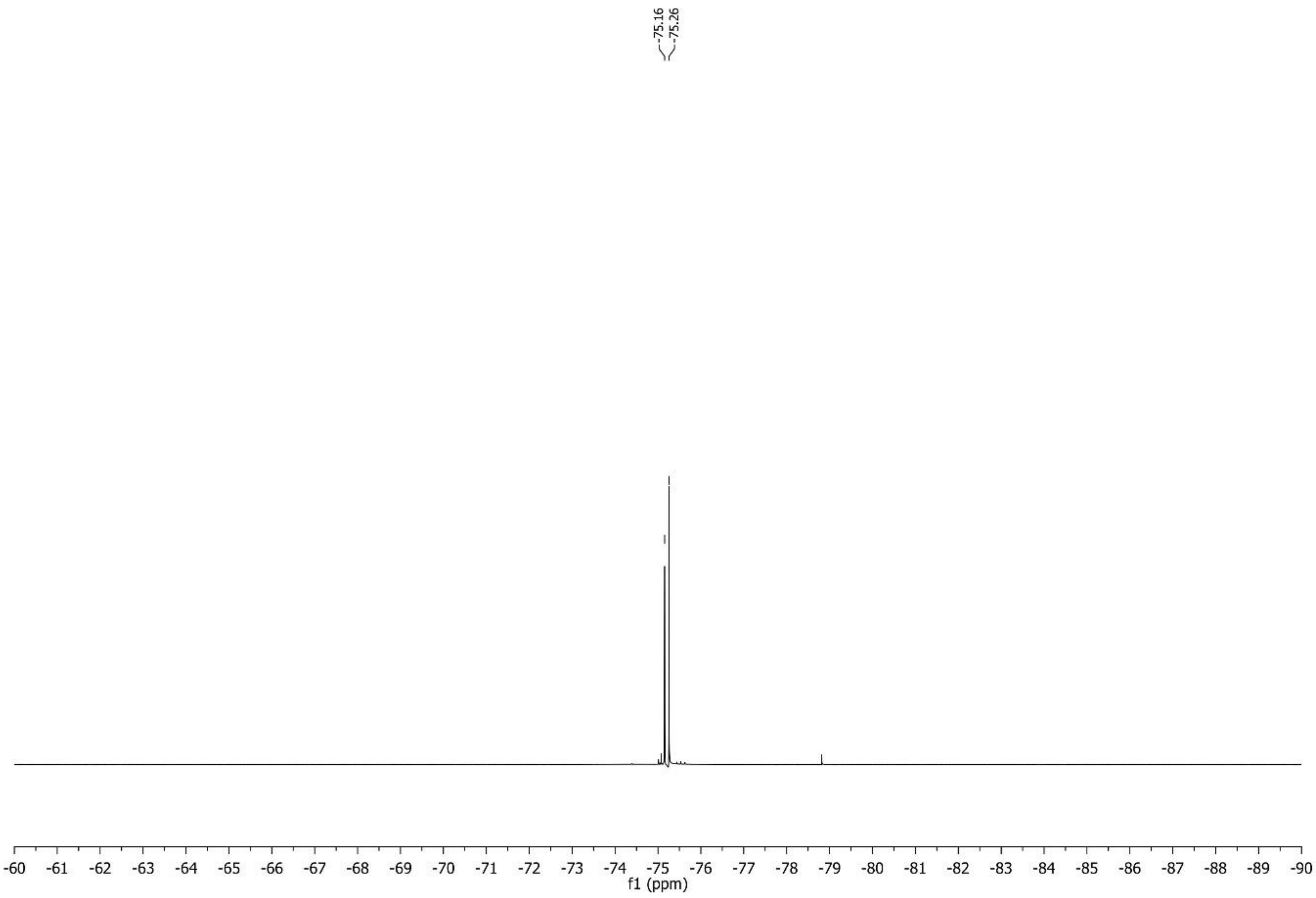

^{1}H NMR (600 MHz, $CDCl_3$) of methyl 3-O-benzyl-2-deoxy-2-fluoro-D-*erythro*-furanoside (22)

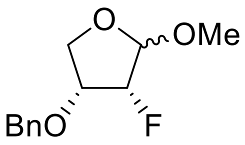

22

$C_{12}H_{15}FO_3$

226.25 g/mol

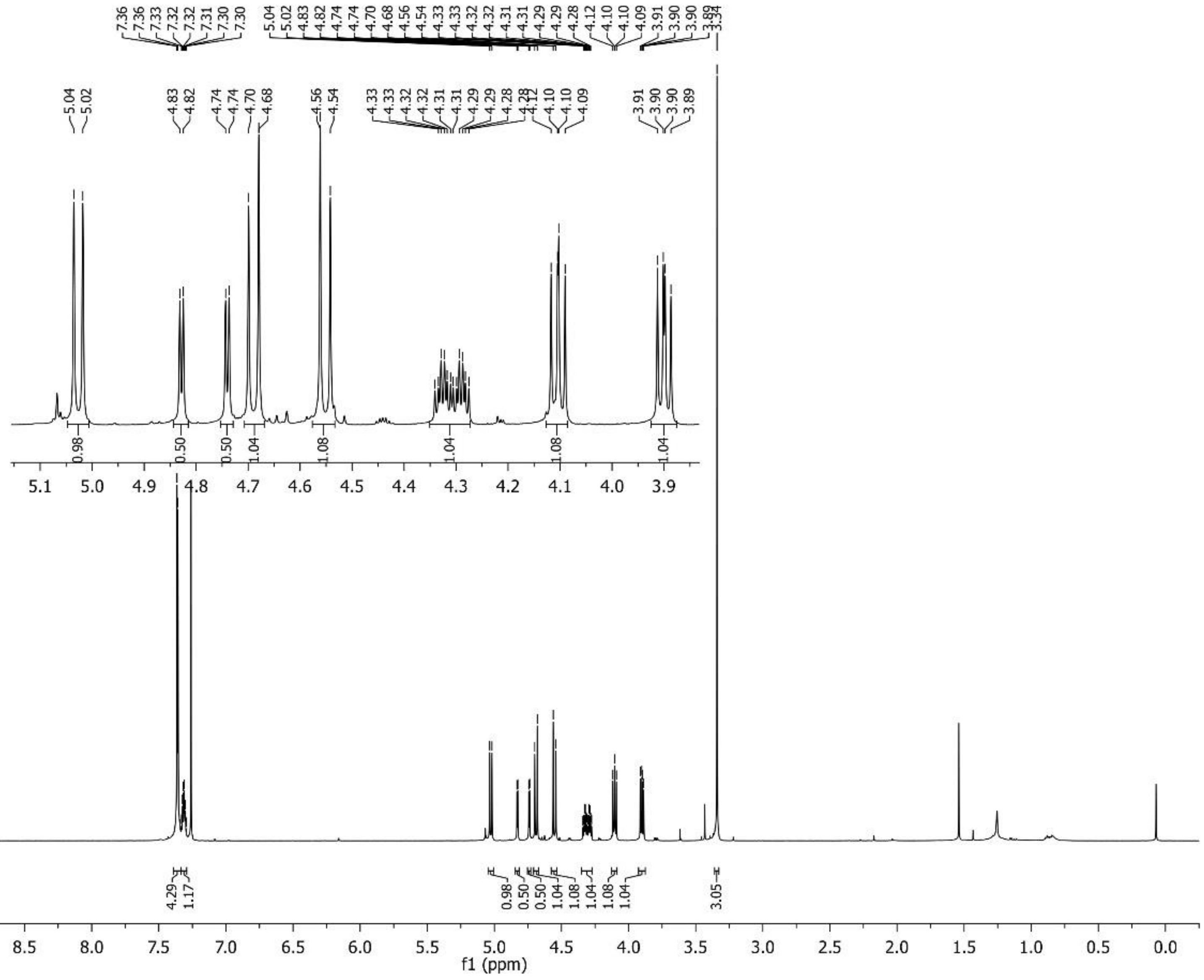

^{13}C NMR (151 MHz, $CDCl_3$) of methyl 3-O-benzyl-2-deoxy-2-fluoro-D-*erythro*-furanoside (22)

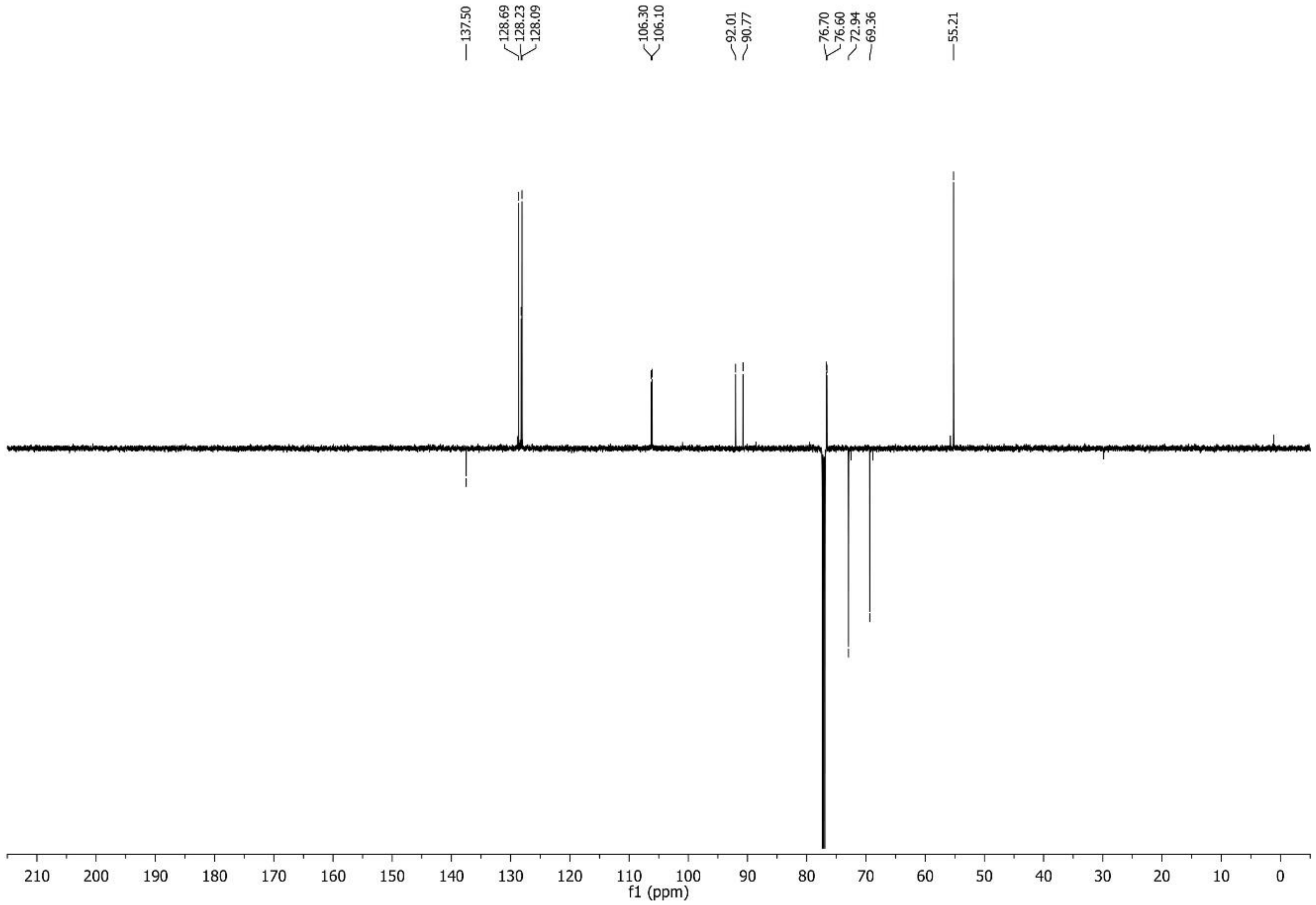

^{19}F NMR (659 MHz, CDCl$_3$) of methyl 3-O-benzyl-2-deoxy-2-fluoro-D-*erythro*-furanoside (22)

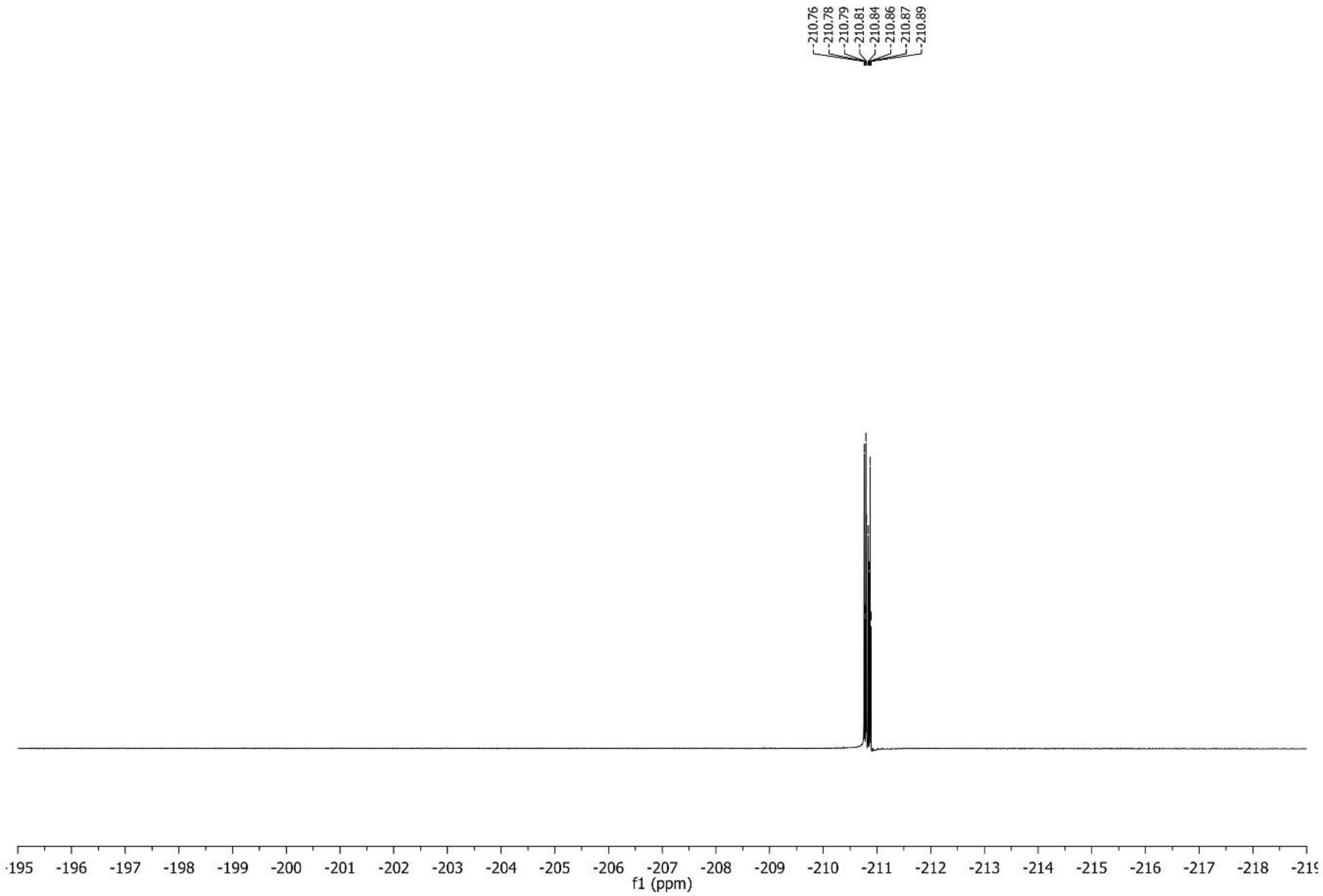

^{1}H NMR (600 MHz, $CDCl_3$) of 3-*O*-benzyl-2-deoxy-2-fluoro-D-erythrose (23)

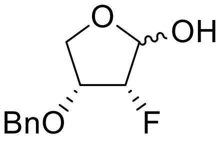

23

$C_{11}H_{13}FO_3$

212.22 g/mol

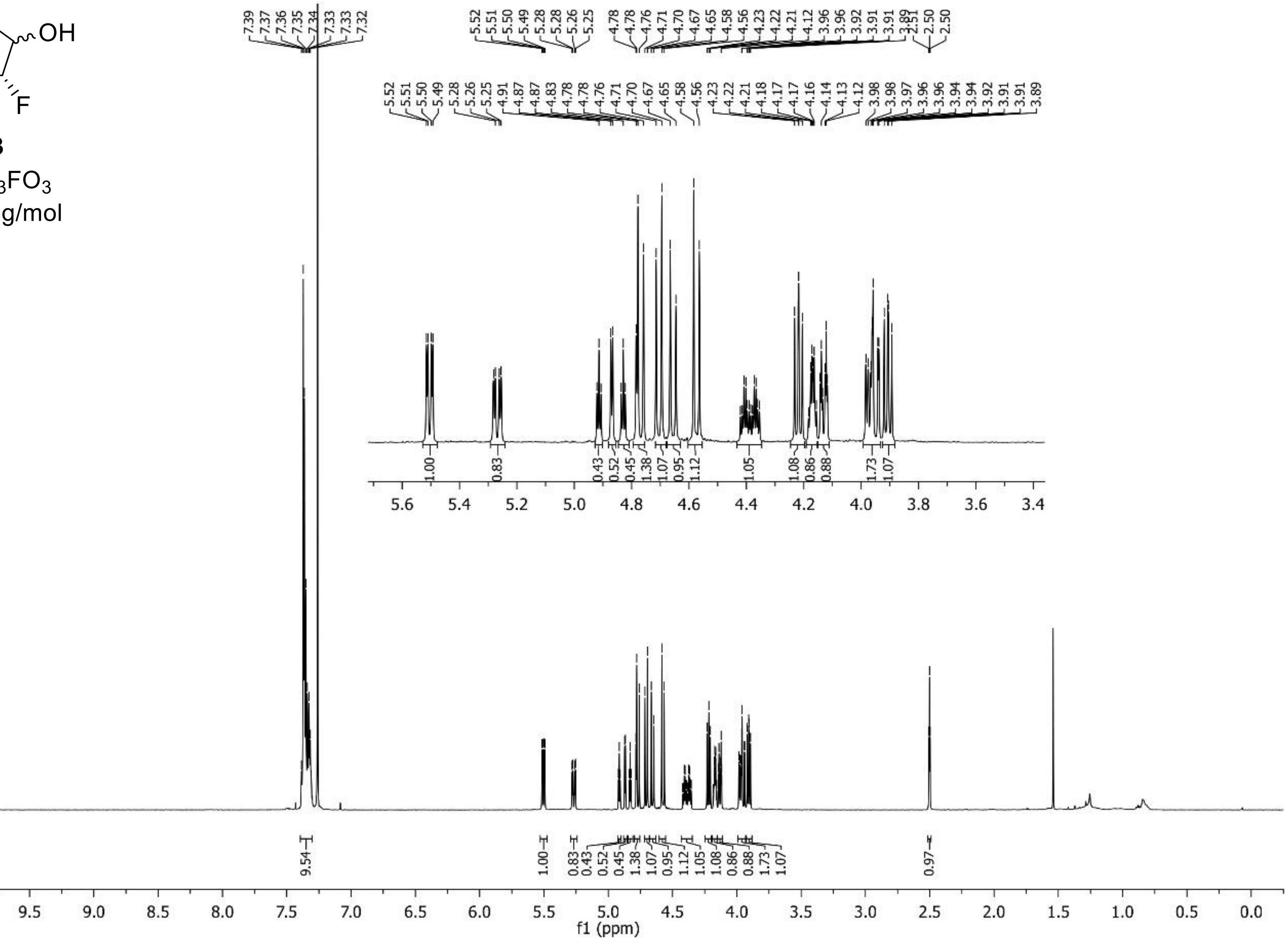

^{13}C NMR (151 MHz, $CDCl_3$) of 3-*O*-benzyl-2-deoxy-2-fluoro-D-erythrose (23)

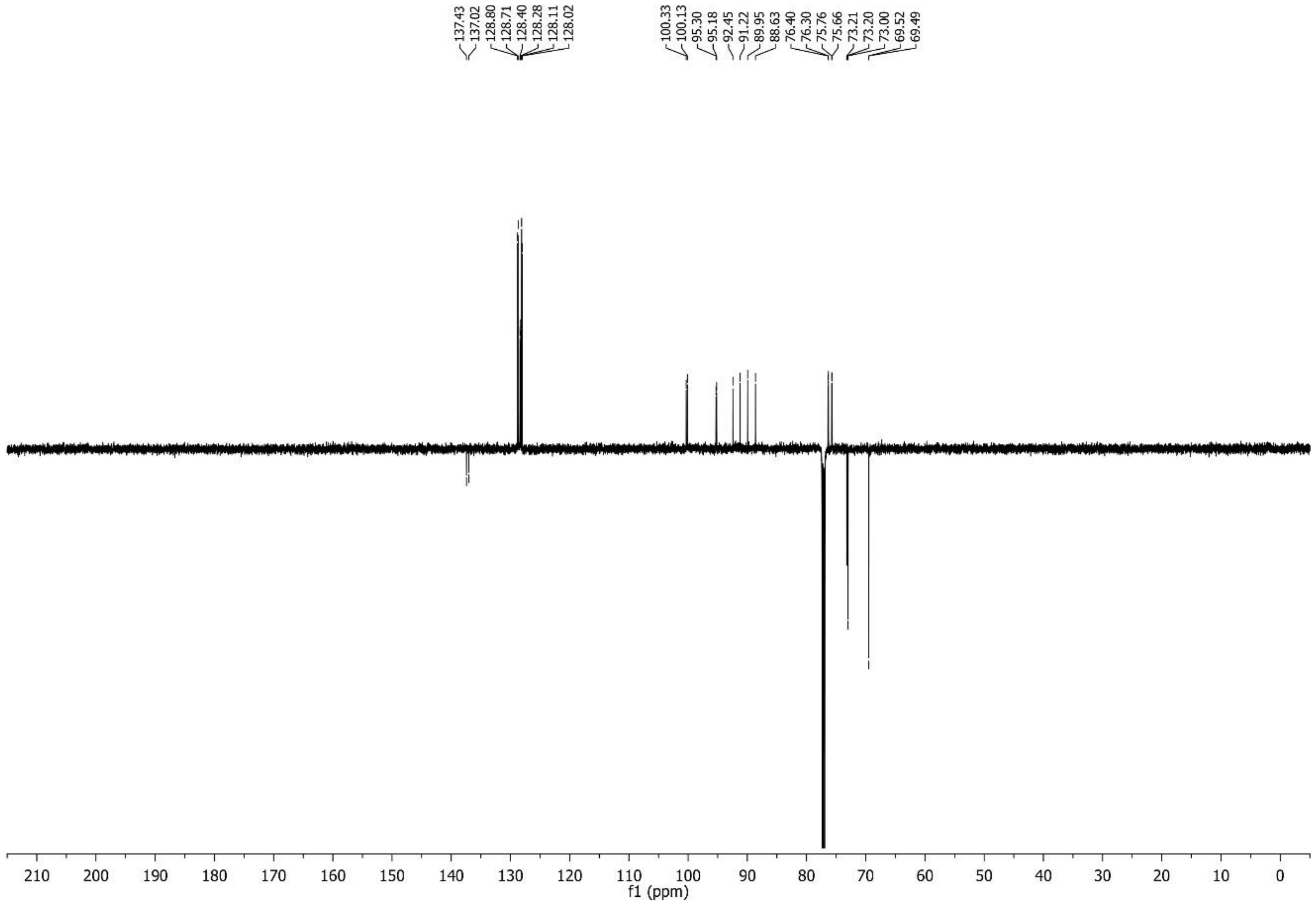

^{19}F NMR (659 MHz, CDCl$_3$) of 3-*O*-benzyl-2-deoxy-2-fluoro-D-erythrose (23)

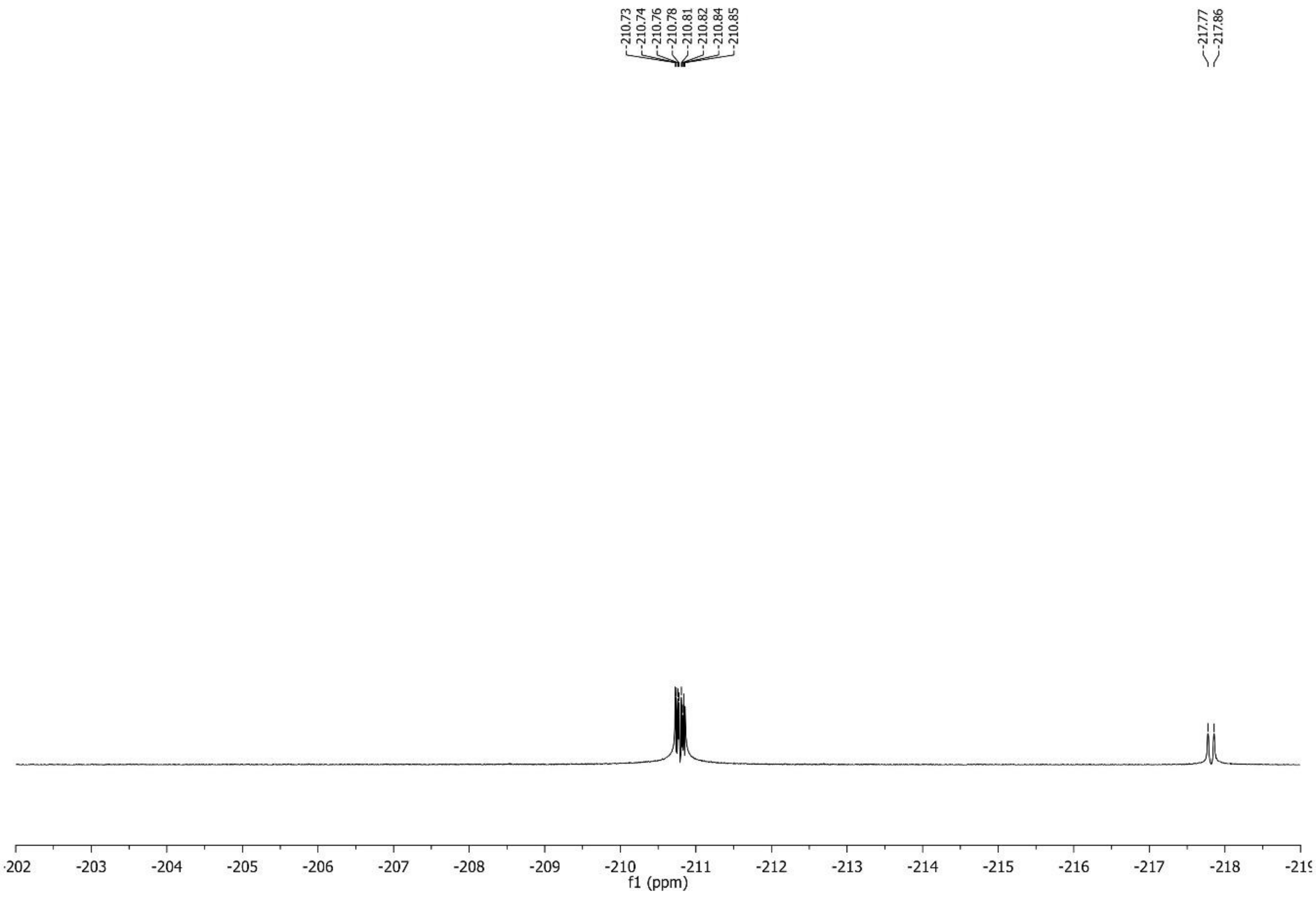

^{1}H NMR (700 MHz, CDCl$_3$) of 3-*O*-benzyl-2-deoxy-2-fluoro-D-*erythro*-1,4-lactone (24)

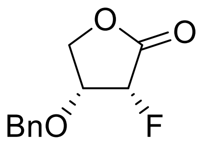

24

$C_{11}H_{11}FO_3$

210.20 g/mol

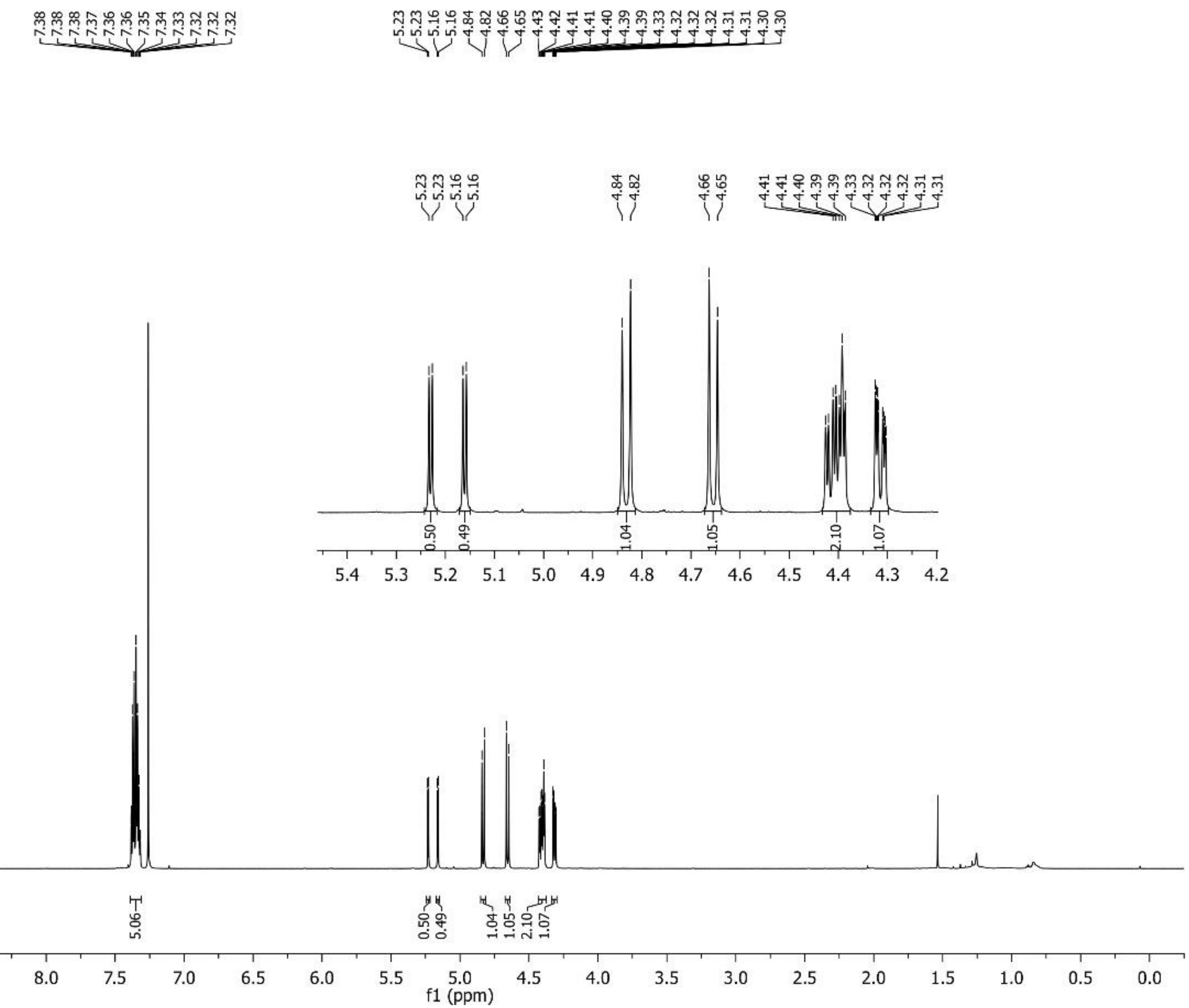

^{13}C NMR (176 MHz, CDCl$_3$) of 3-*O*-benzyl-2-deoxy-2-fluoro-D-*erythro*-1,4-lactone (24)

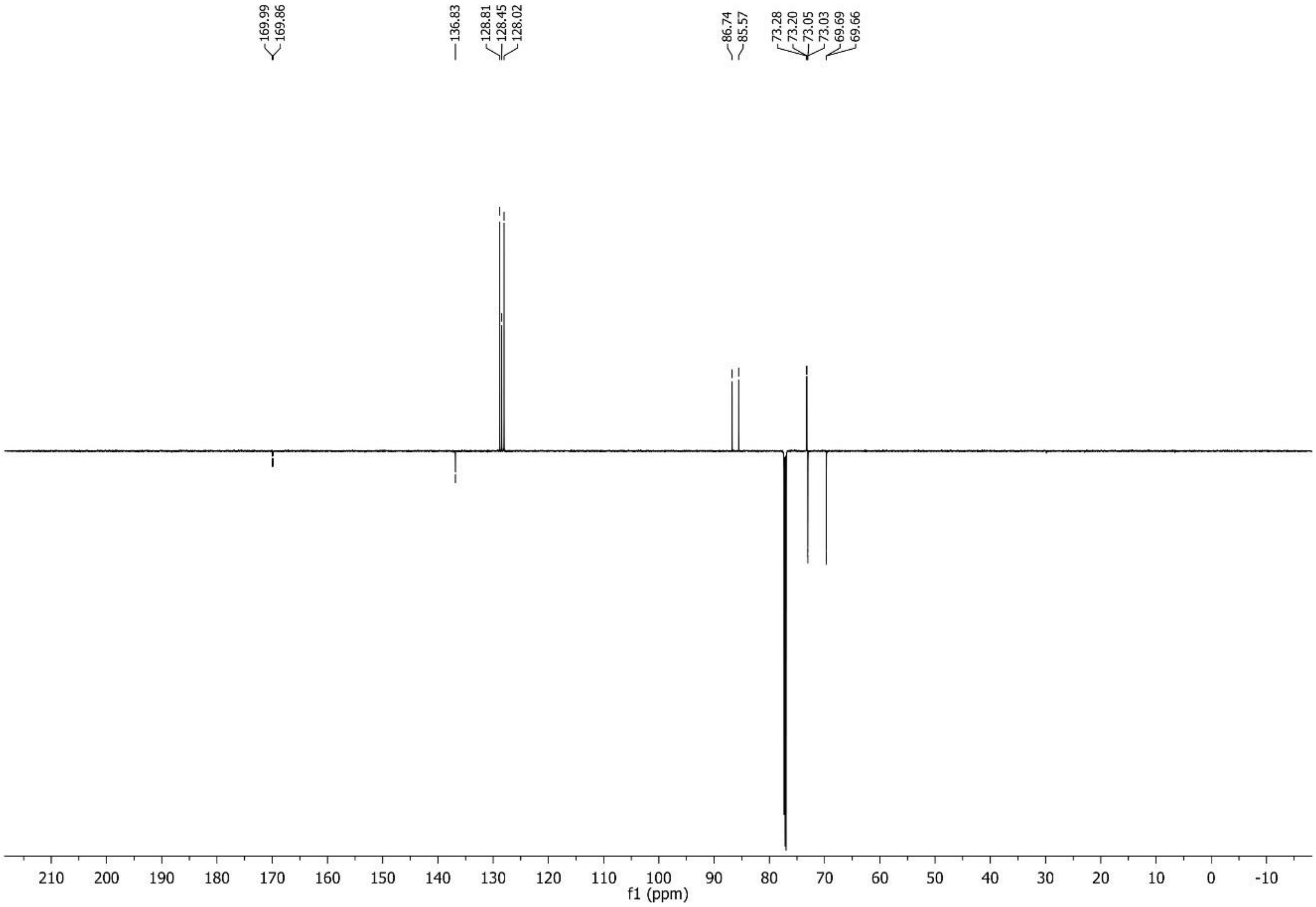

^{19}F NMR (659 MHz, CDCl$_3$) of 3-*O*-benzyl-2-deoxy-2-fluoro-D-*erythro*-1,4-lactone (24)

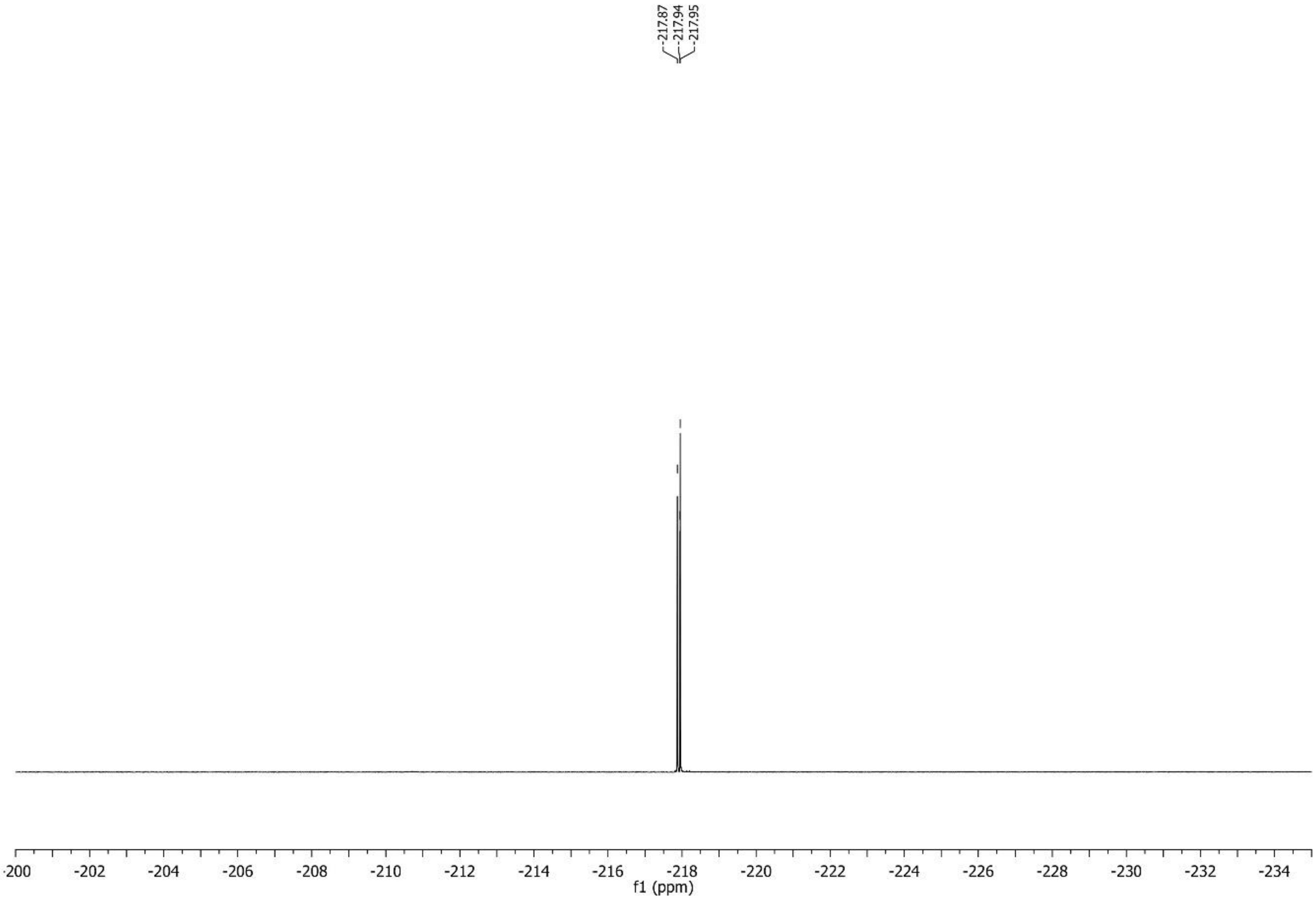

^{1}H NMR (600 MHz, CDCl$_3$) of 2,5-anhydro-4-*O*-benzyl-3-deoxy-3-fluoro-D-*erythro*-pent-1-enitol (25)

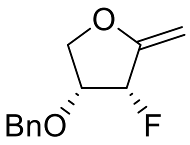

25

$C_{12}H_{13}FO_2$

208.23 g/mol

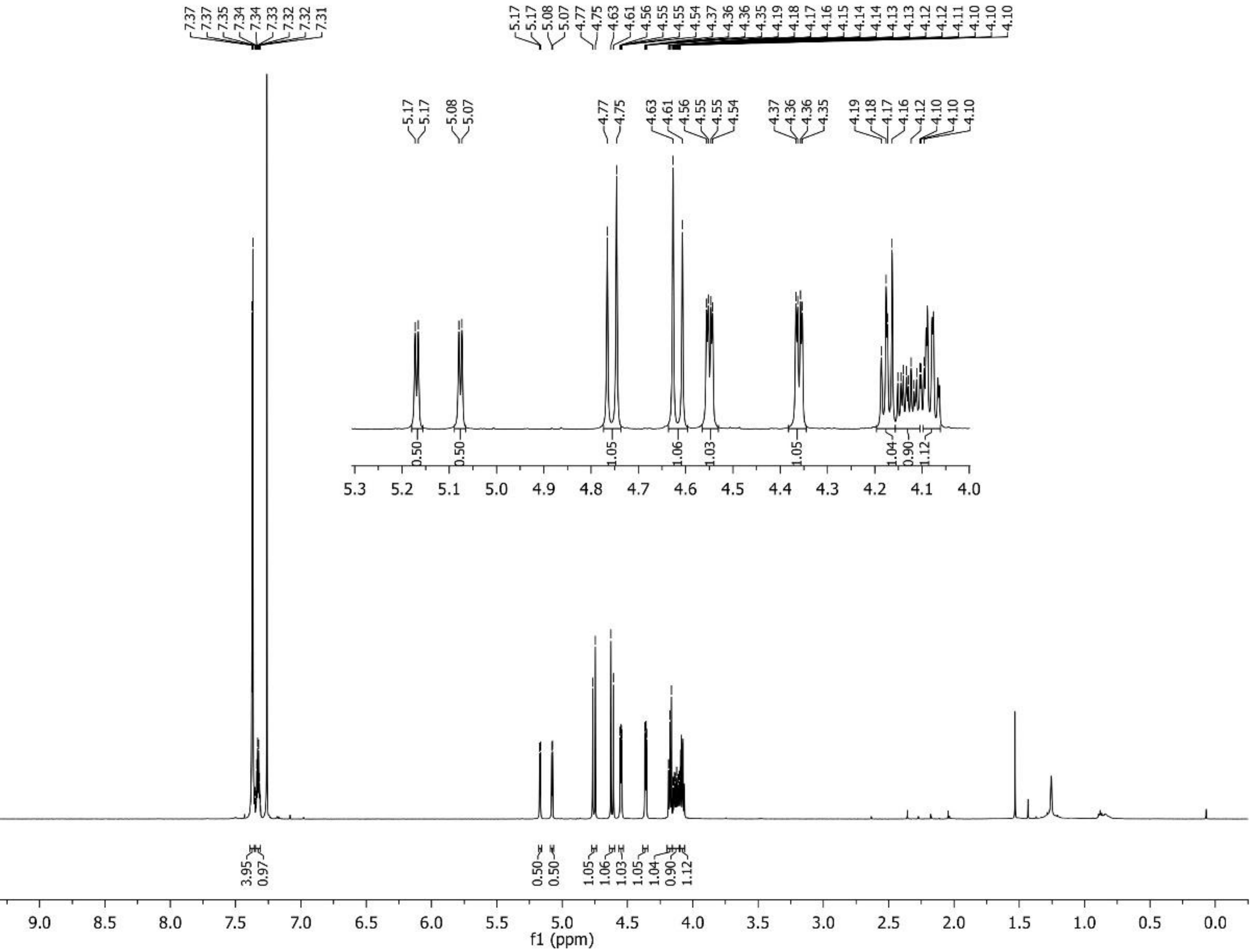

^{13}C NMR (151 MHz, $CDCl_3$) of 2,5-anhydro-4-*O*-benzyl-3-deoxy-3-fluoro-D-*erythro*-pent-1-enitol (25)

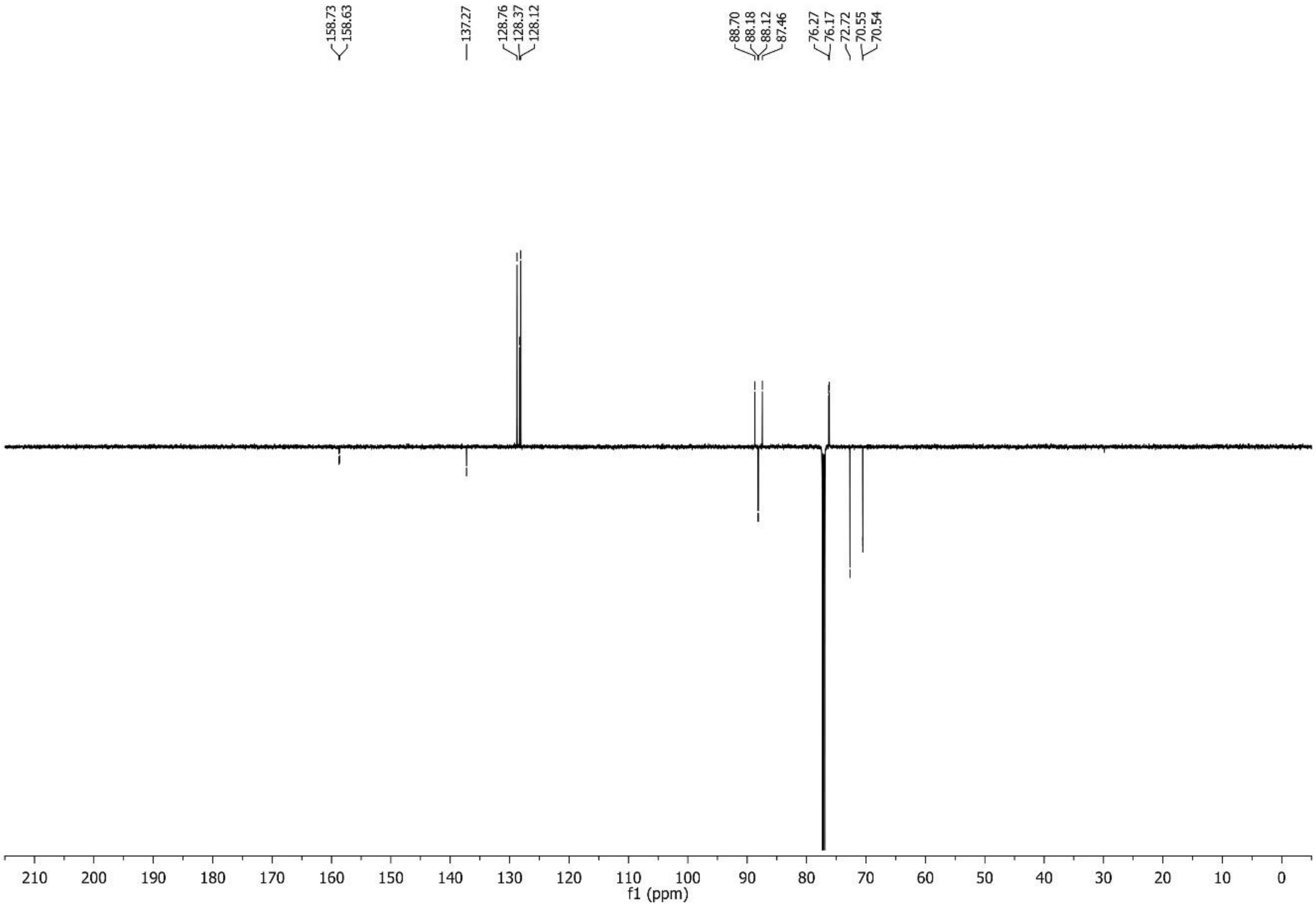

^{19}F NMR (659 MHz, CDCl$_3$) of 2,5-anhydro-4-*O*-benzyl-3-deoxy-3-fluoro-D-*erythro*-pent-1-enitol (25)

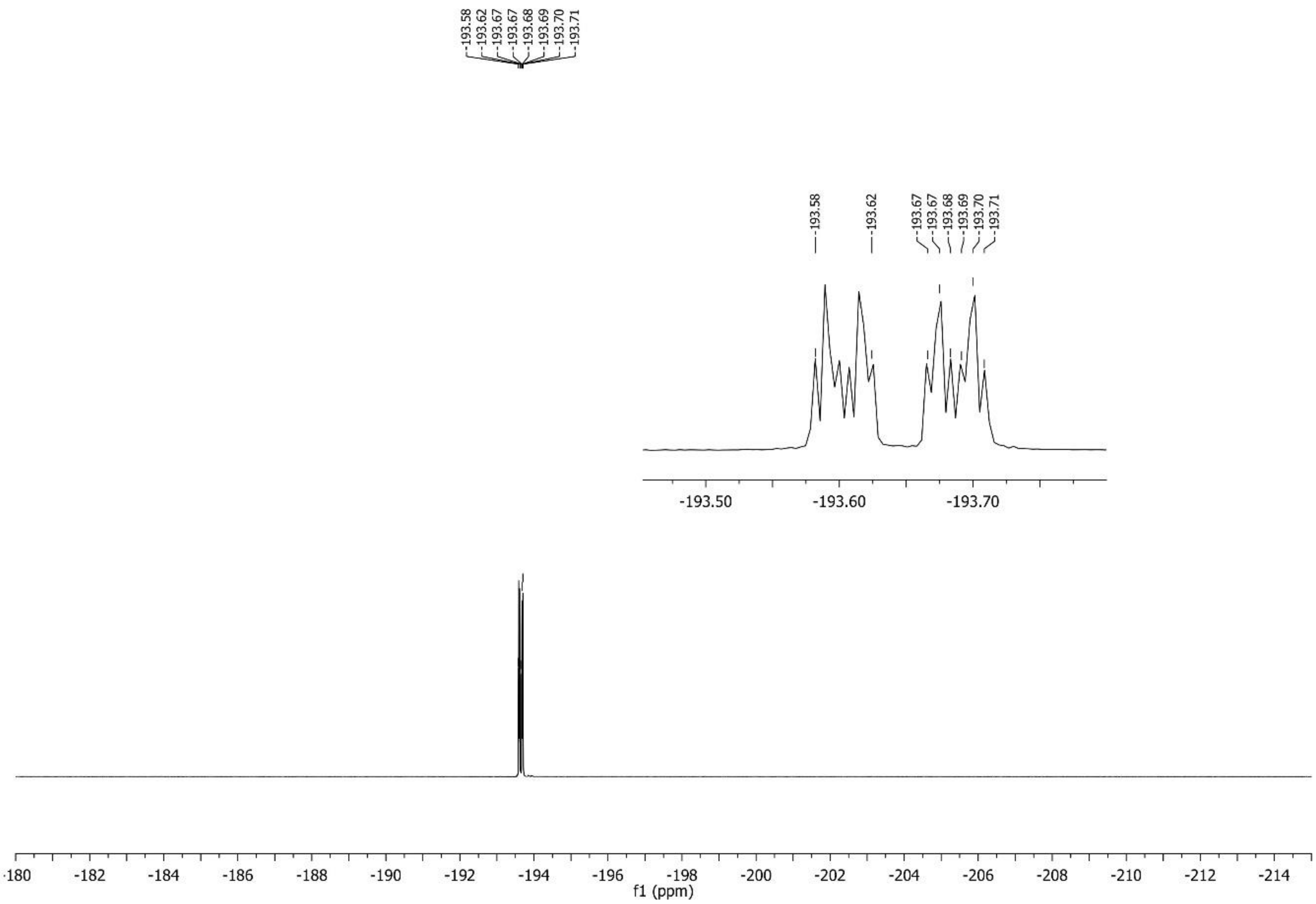

^{1}H NMR (700 MHz, $CDCl_3$) of 4-*O*-benzyl-3-deoxy-3-fluoro-D-ribulose (26)

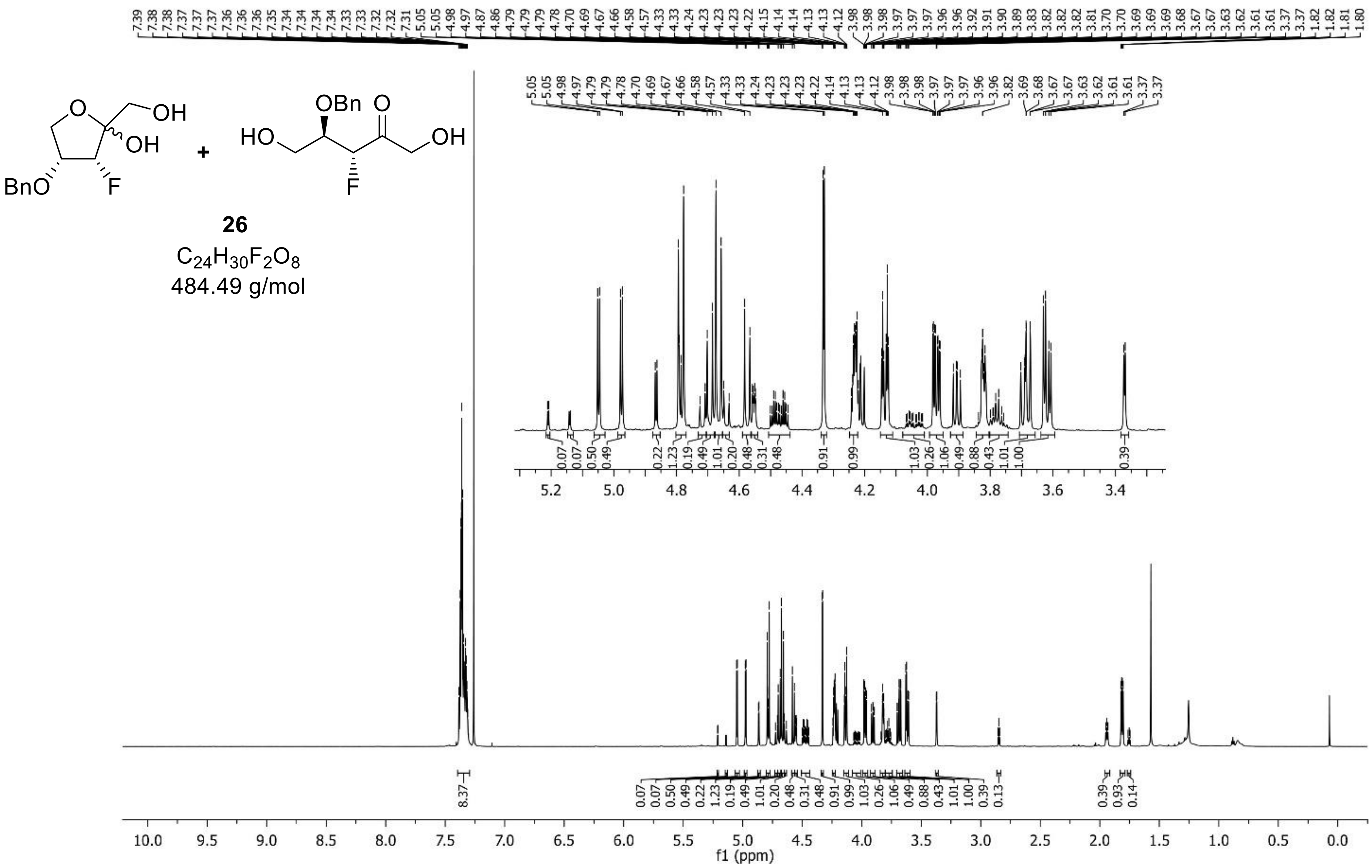

26

$C_{24}H_{30}F_2O_8$

484.49 g/mol

^{13}C NMR (176 MHz, CDCl$_3$) of 4-*O*-benzyl-3-deoxy-3-fluoro-D-ribulose (26)

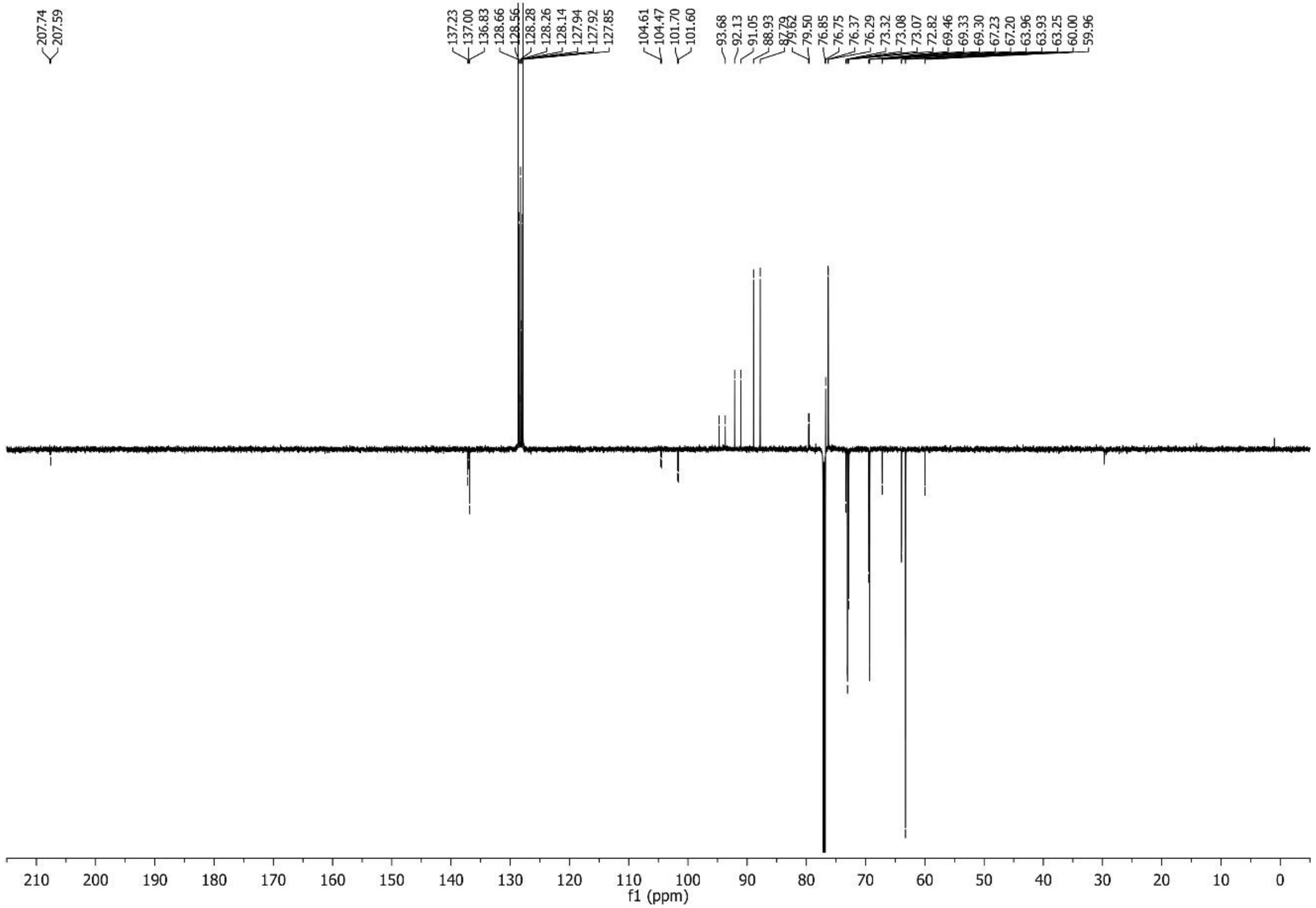

^{19}F NMR (659 MHz, $CDCl_3$) of 4-*O*-benzyl-3-deoxy-3-fluoro-D-ribulose (26)

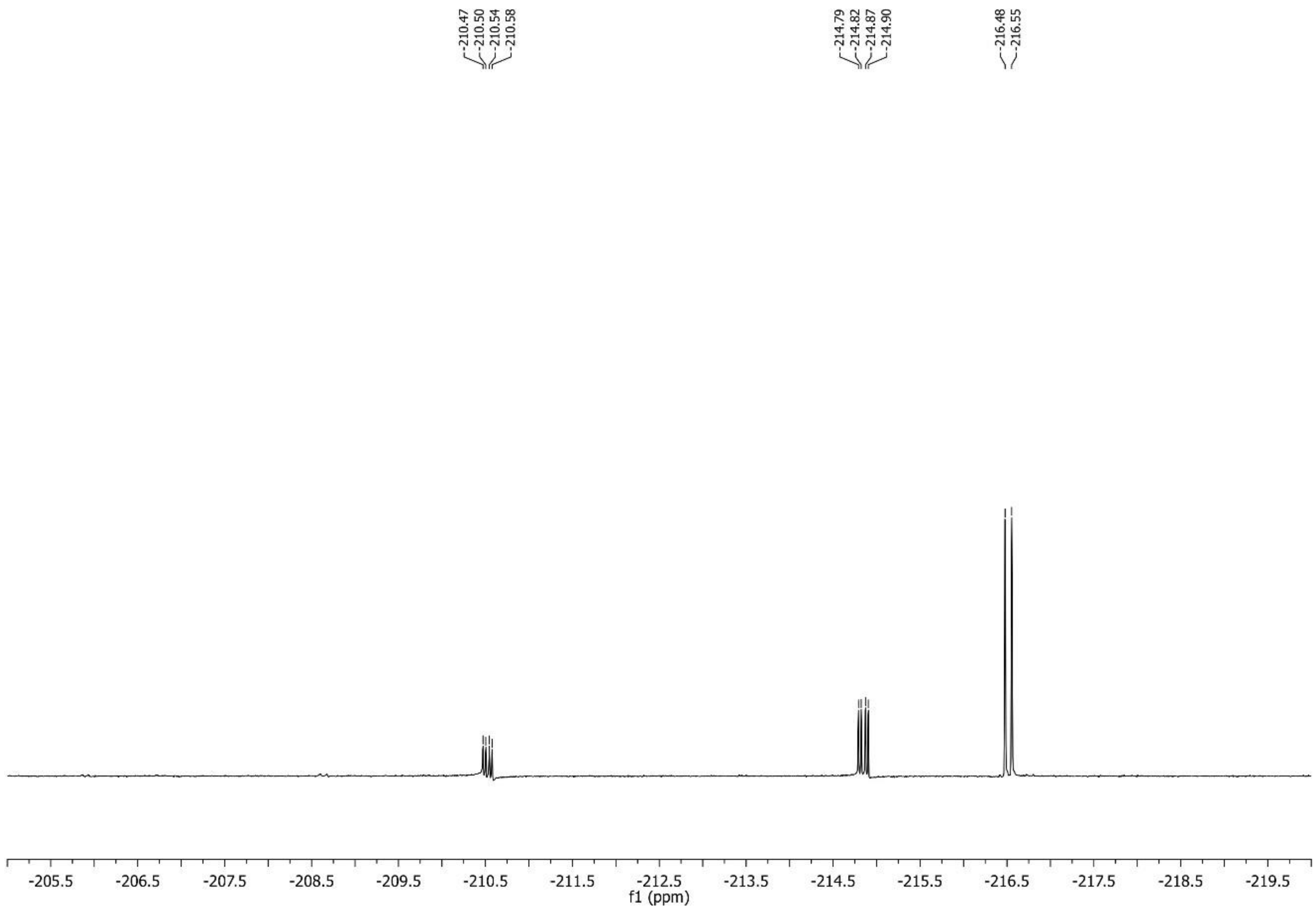

^{1}H NMR (700 MHz, D_2O) of 3-deoxy-3-fluoro-D-ribulose (3DFRu, 4)

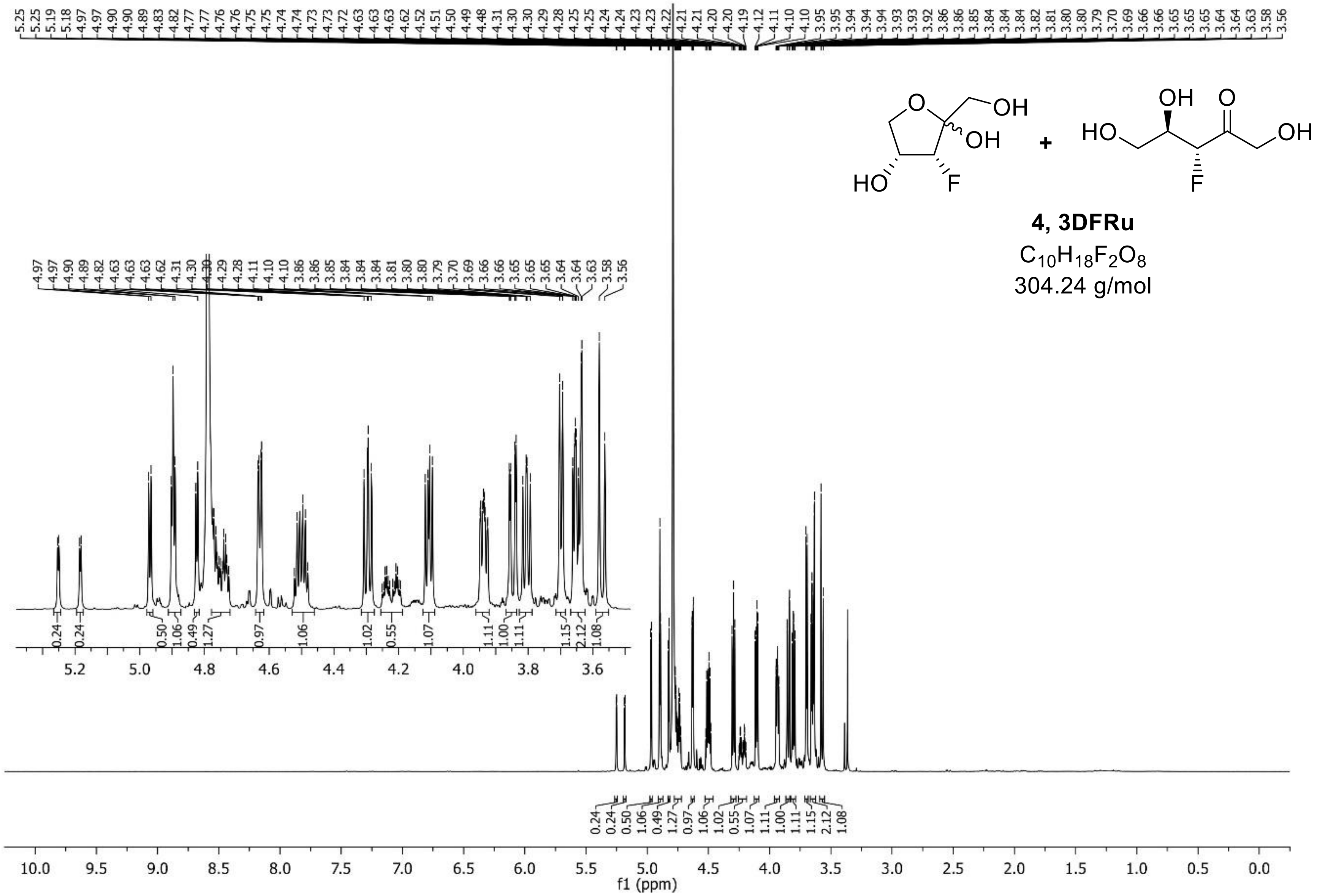

4, 3DFRu

$C_{10}H_{18}F_2O_8$

304.24 g/mol

^{13}C NMR (176 MHz, D_2O) of 3-deoxy-3-fluoro-D-ribulose (3DFRu, 4)

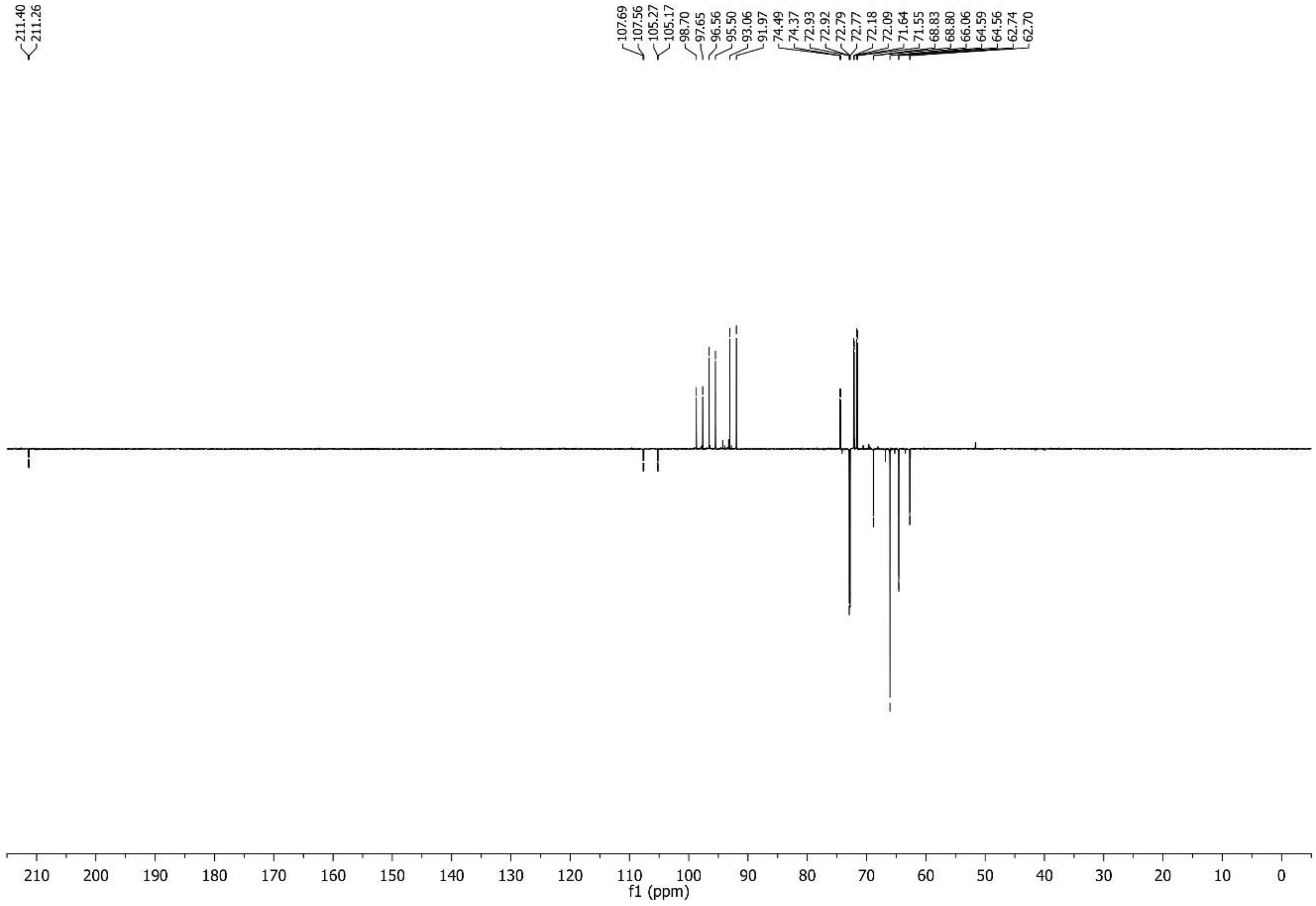

^{19}F NMR (659 MHz, D_2O) of 3-deoxy-3-fluoro-D-ribulose (3DFRu, 4)

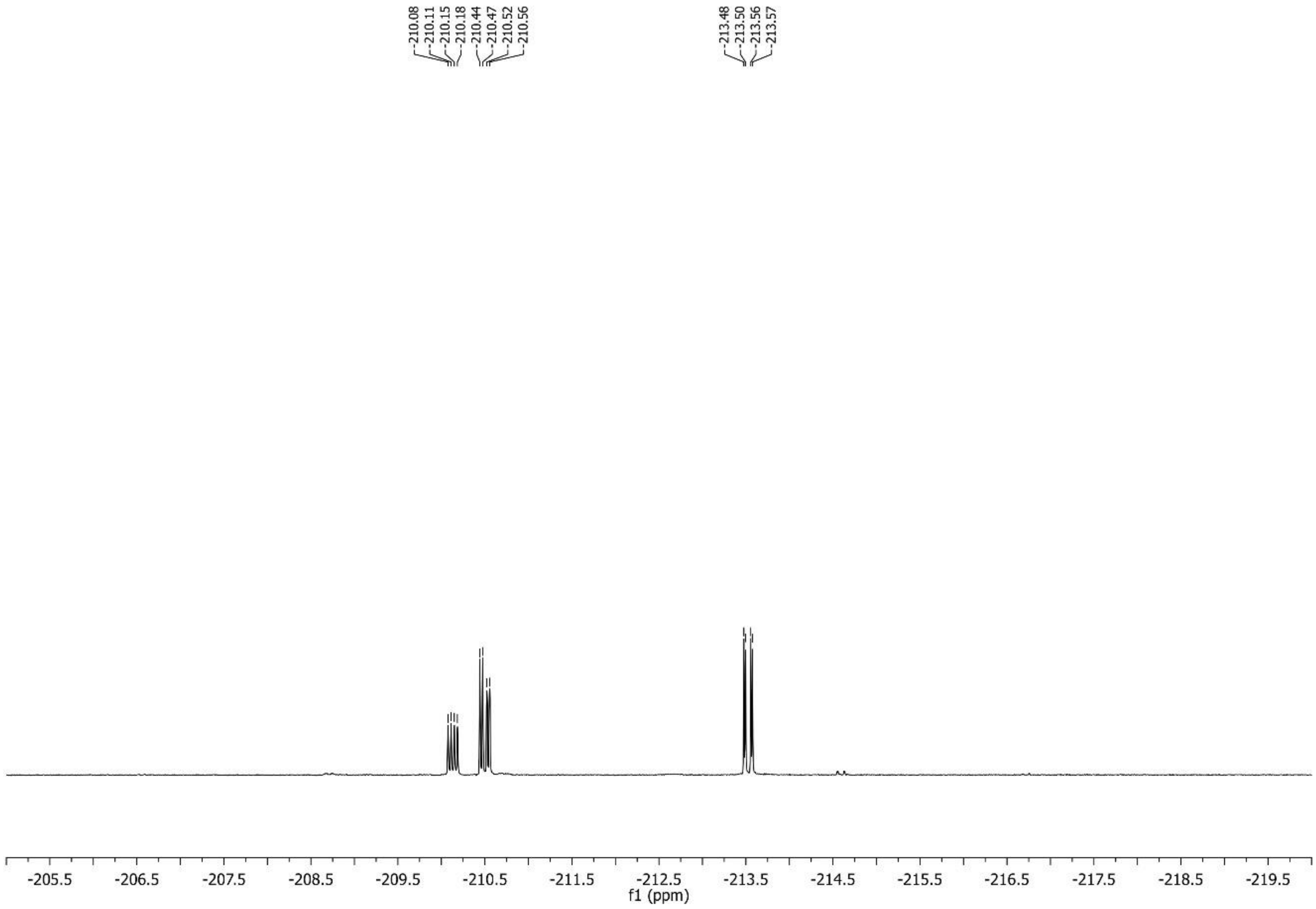

^{1}H NMR (600 MHz, *d_6*-DMSO) of D-*threono*-1,4-lactone (28)

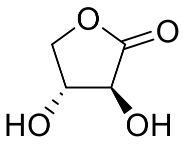

28

$C_4H_6O_4$

118.09 g/mol

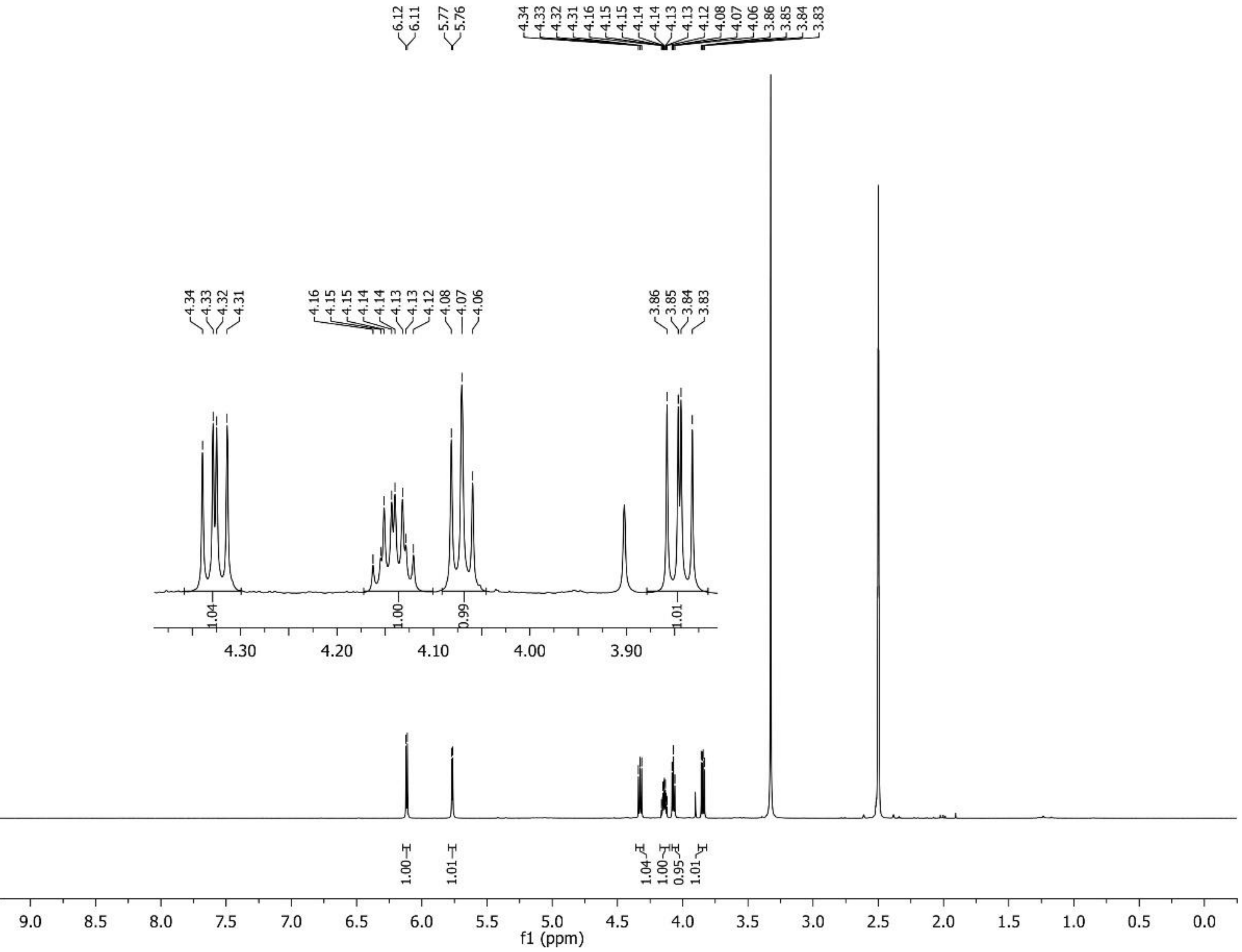

^{13}C NMR (151 MHz, d_6-DMSO) of D-*threono*-1,4-lactone (28)

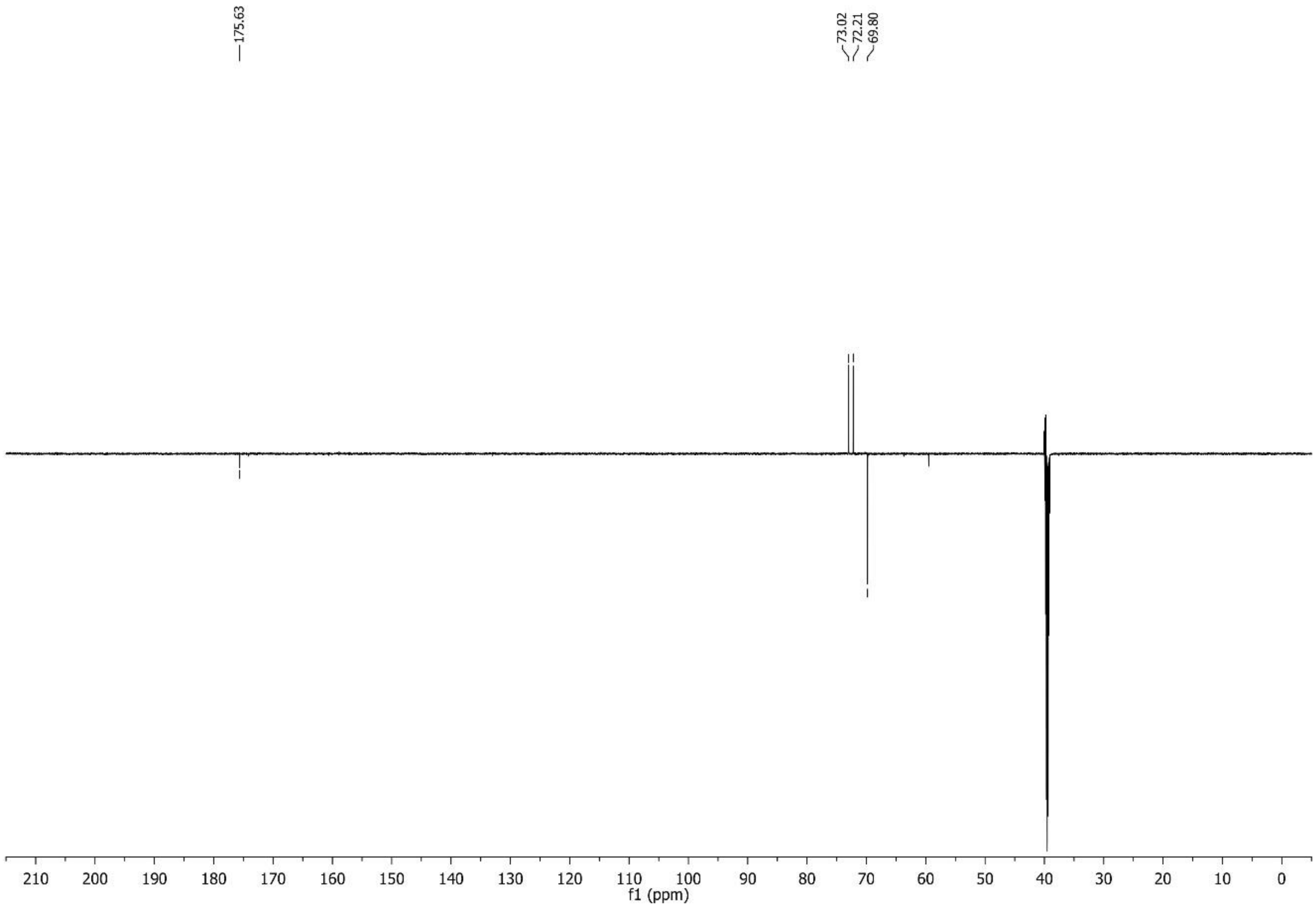

^{1}H NMR (600 MHz, $CDCl_3$) of 2-*O*-triisopropylsilyl-D-*threono*-1,4-lactone (29)

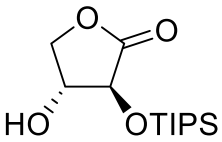

29

$C_{13}H_{26}O_4Si$

274.43 g/mol

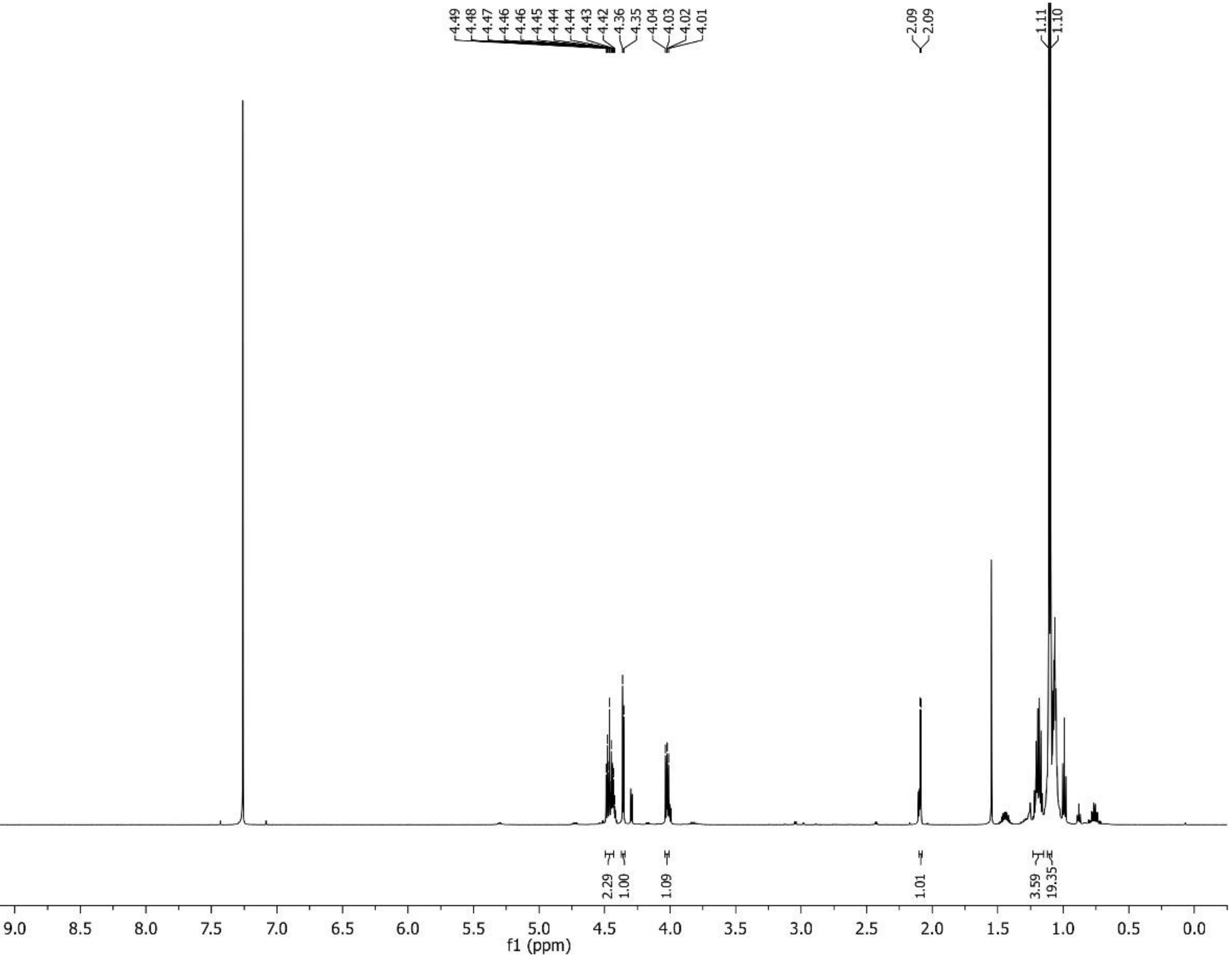

^{13}C NMR (151 MHz, $CDCl_3$) of 2-*O*-triisopropylsilyl-D-*threono*-1,4-lactone (29)

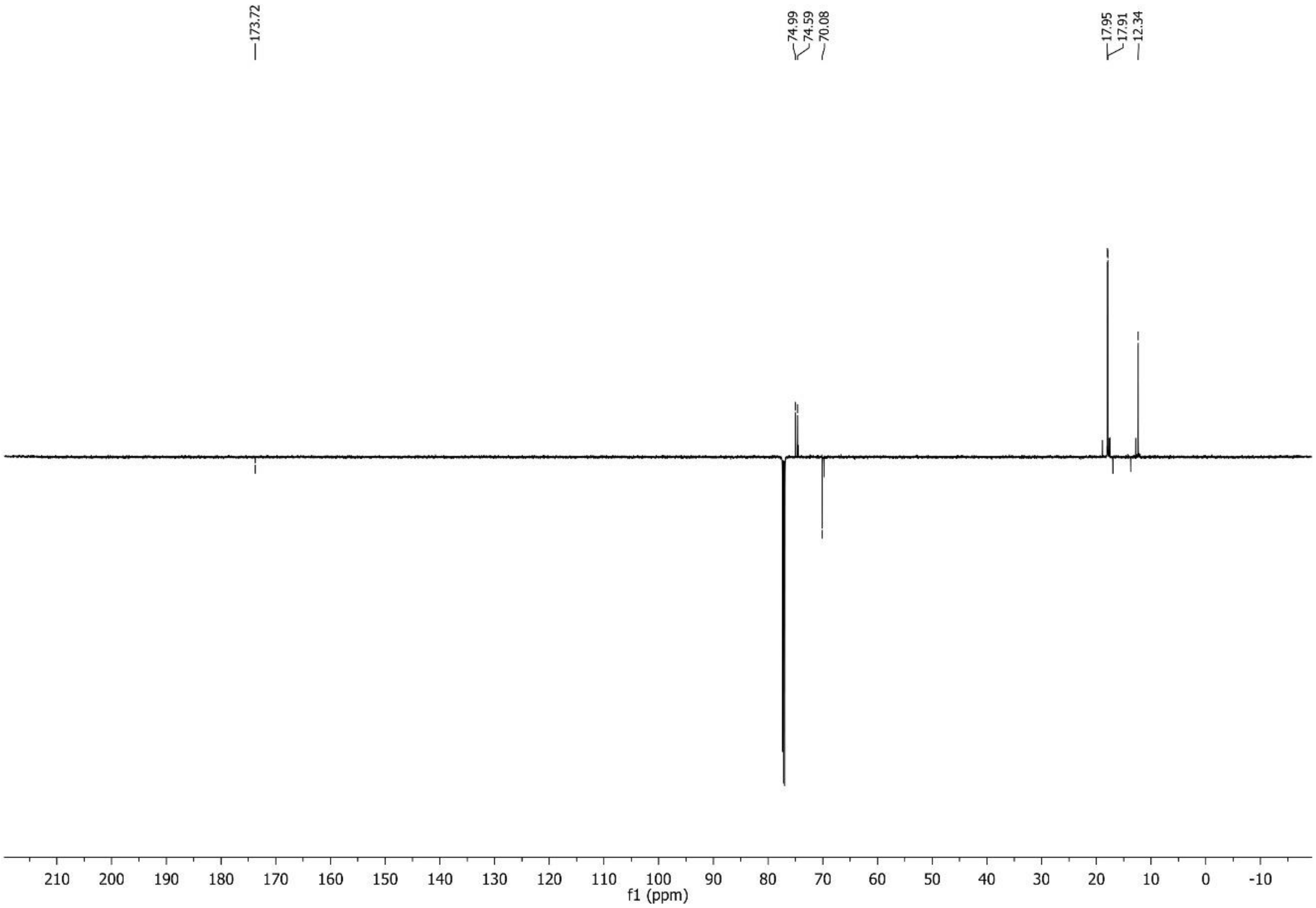

^{1}H NMR (700 MHz, $CDCl_3$) of 3-*O*-benzoyl-2-*O*-triisopropylsilyl-D-*threono*-1,4-lactone (30)

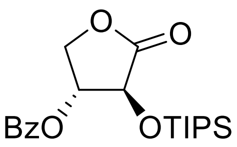

30
$C_{20}H_{30}O_5Si$
378.54 g/mol

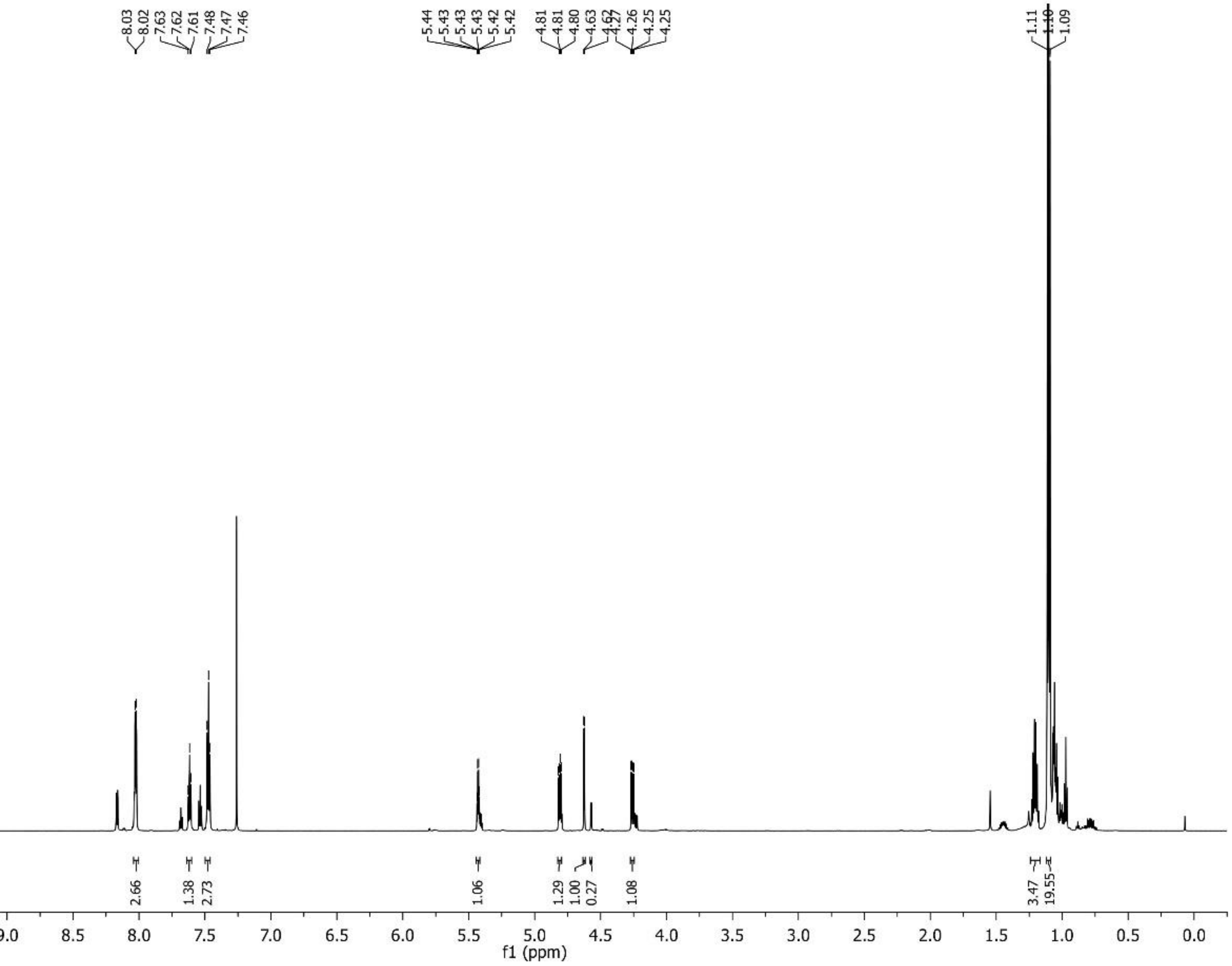

^{13}C NMR (176 MHz, $CDCl_3$) of 3-*O*-benzoyl-2-*O*-triisopropylsilyl-D-*threono*-1,4-lactone (30)

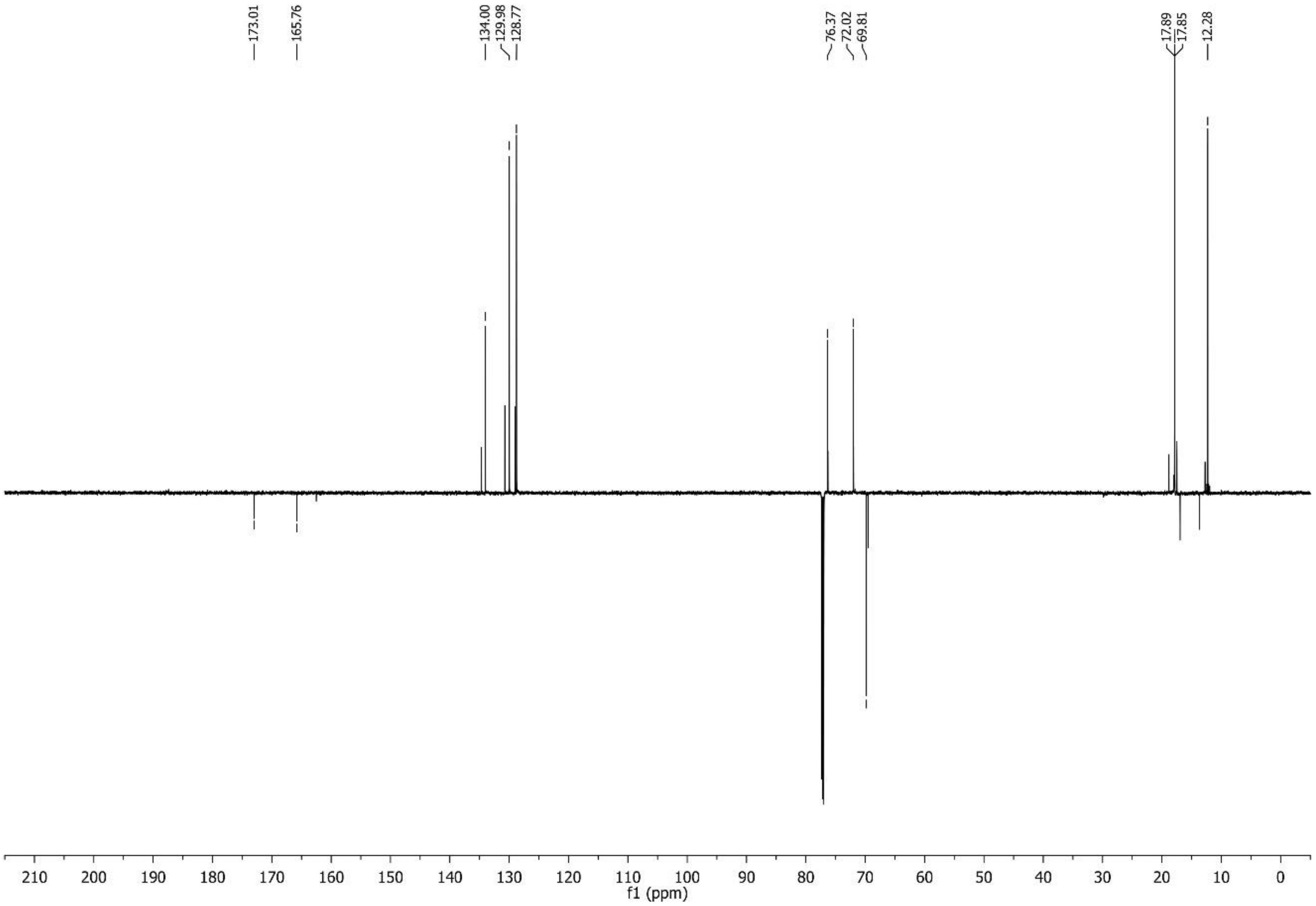

^{1}H NMR (700 MHz, $CDCl_3$) of 2,5-anhydro-4-*O*-benzoyl-1-deoxy-3-*O*-triisopropylsilyl-D-*threo*-pent-1-enitol (31)

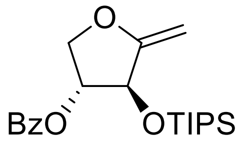

31

$C_{21}H_{32}O_4Si$

376.57 g/mol

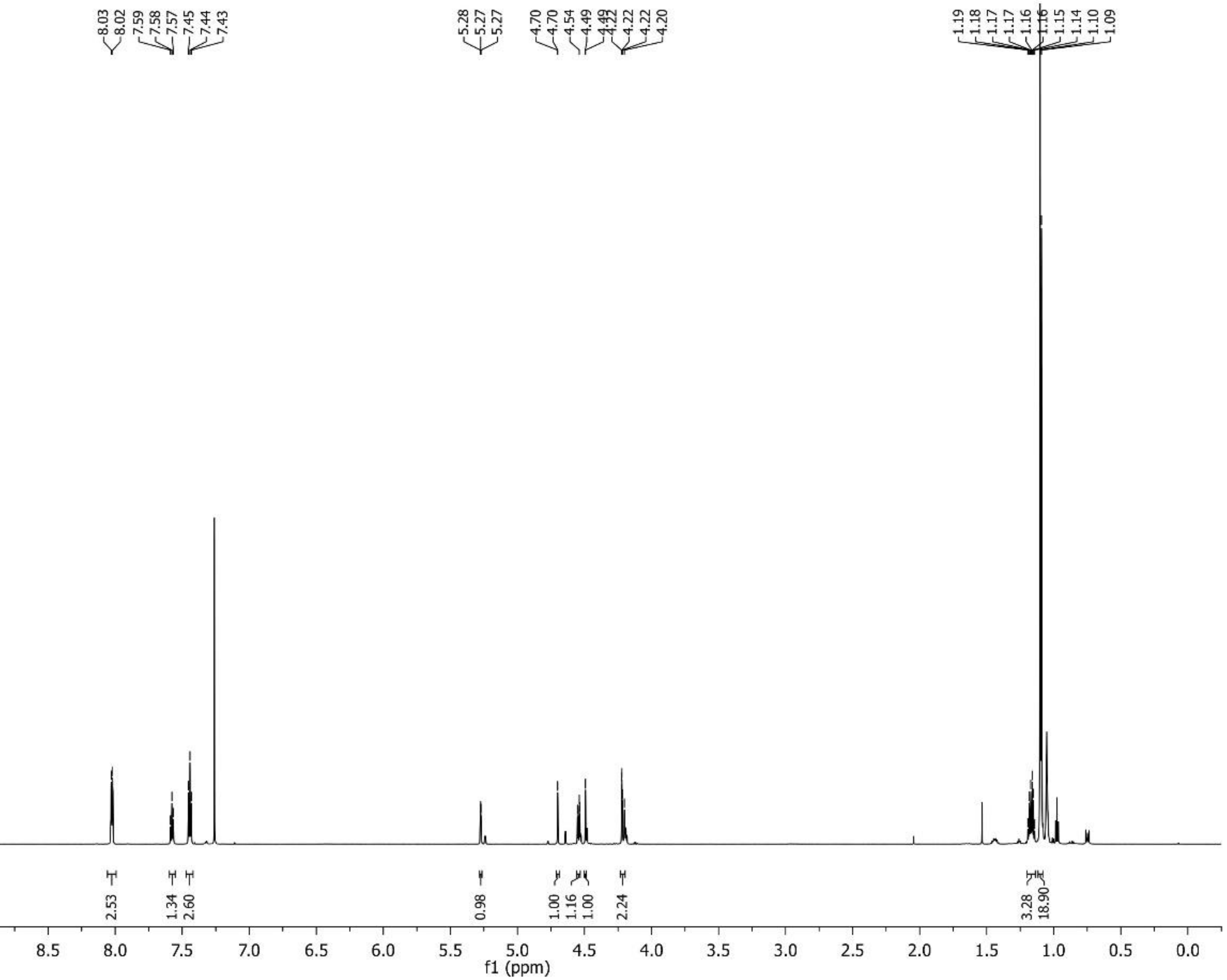

^{13}C NMR (176 MHz, $CDCl_3$) of 2,5-anhydro-4-*O*-benzoyl-1-deoxy-3-*O*-triisopropylsilyl-D-*threo*-pent-1-enitol (31)

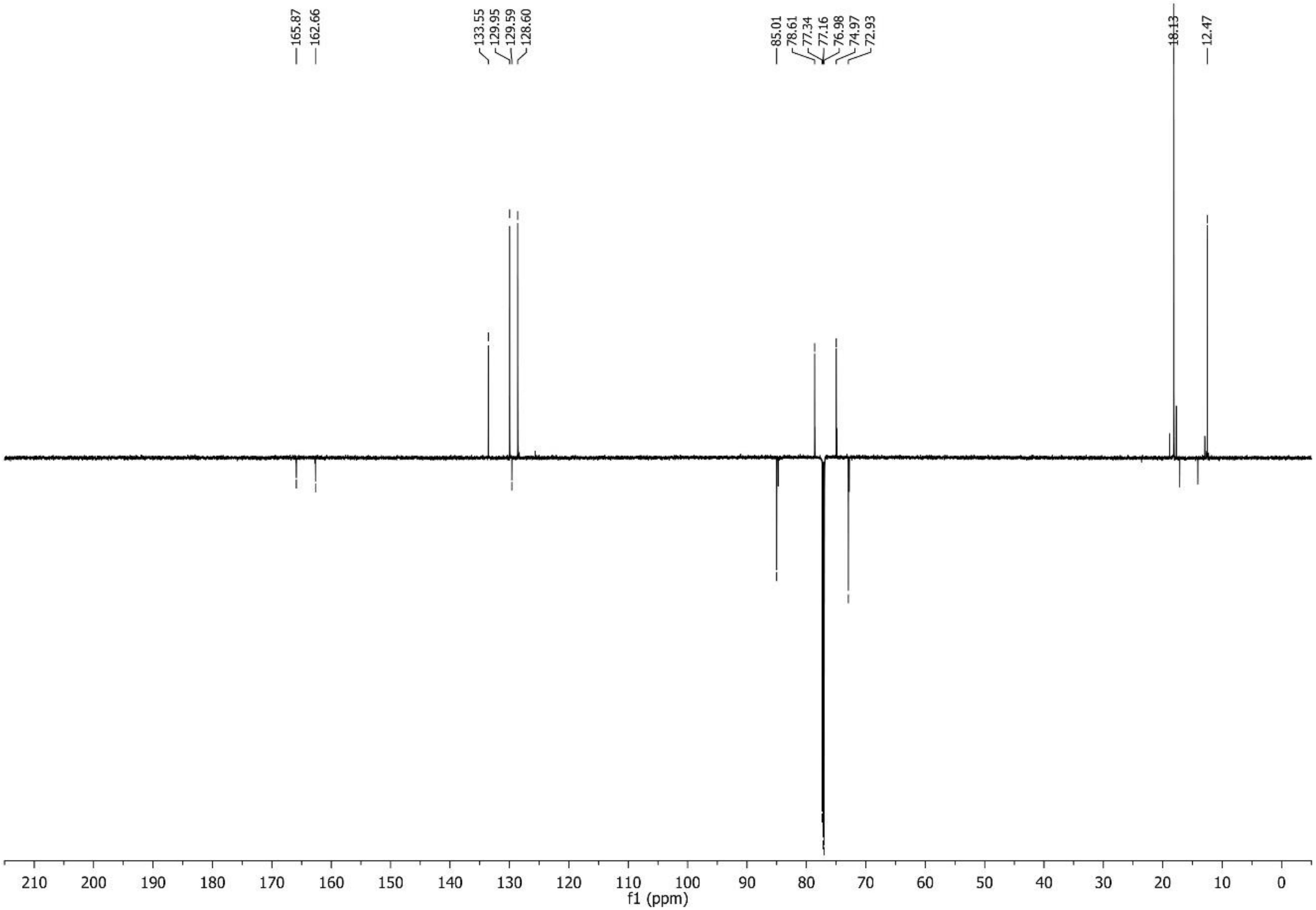

¹H NMR (700 MHz, CDCl₃) of 4-*O*-benzoyl-3-*O*-triisopropylsilyl-D-xylulose (32)

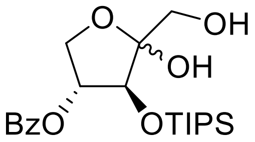

32

$C_{21}H_{34}O_6Si$

410.58 g/mol

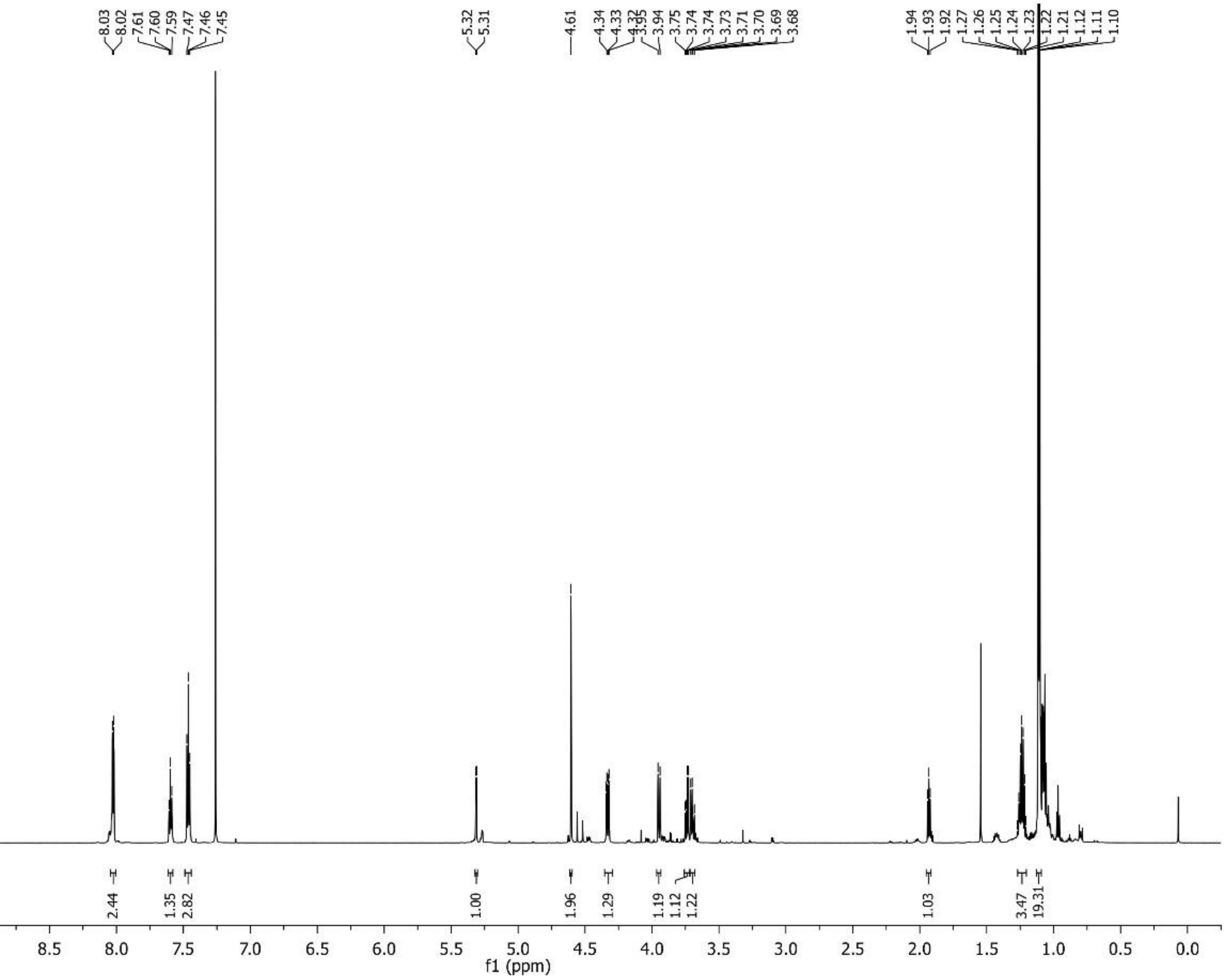

^{13}C NMR (176 MHz, $CDCl_3$) of 4-*O*-benzoyl-3-*O*-triisopropylsilyl-D-xylulose 32)

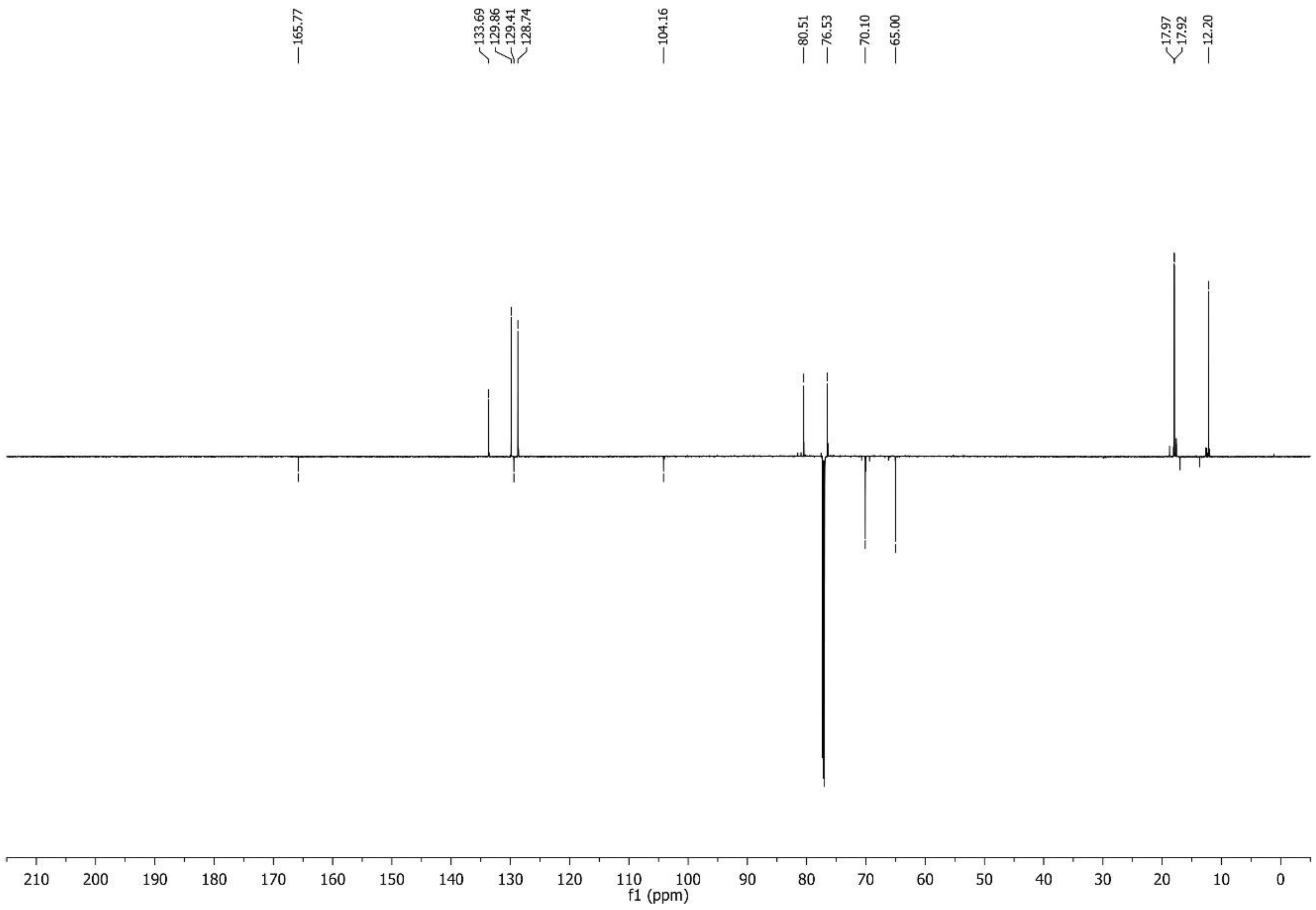

^{1}H NMR (700 MHz, CDCl$_3$) of 4-*O*-benzoyl-1,2-*O*-isopropylidene-3-*O*-triisopropylsilyl-D-xylulose (33)

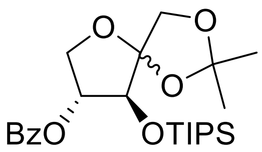

33

$C_{24}H_{38}O_6Si$

450.65 g/mol

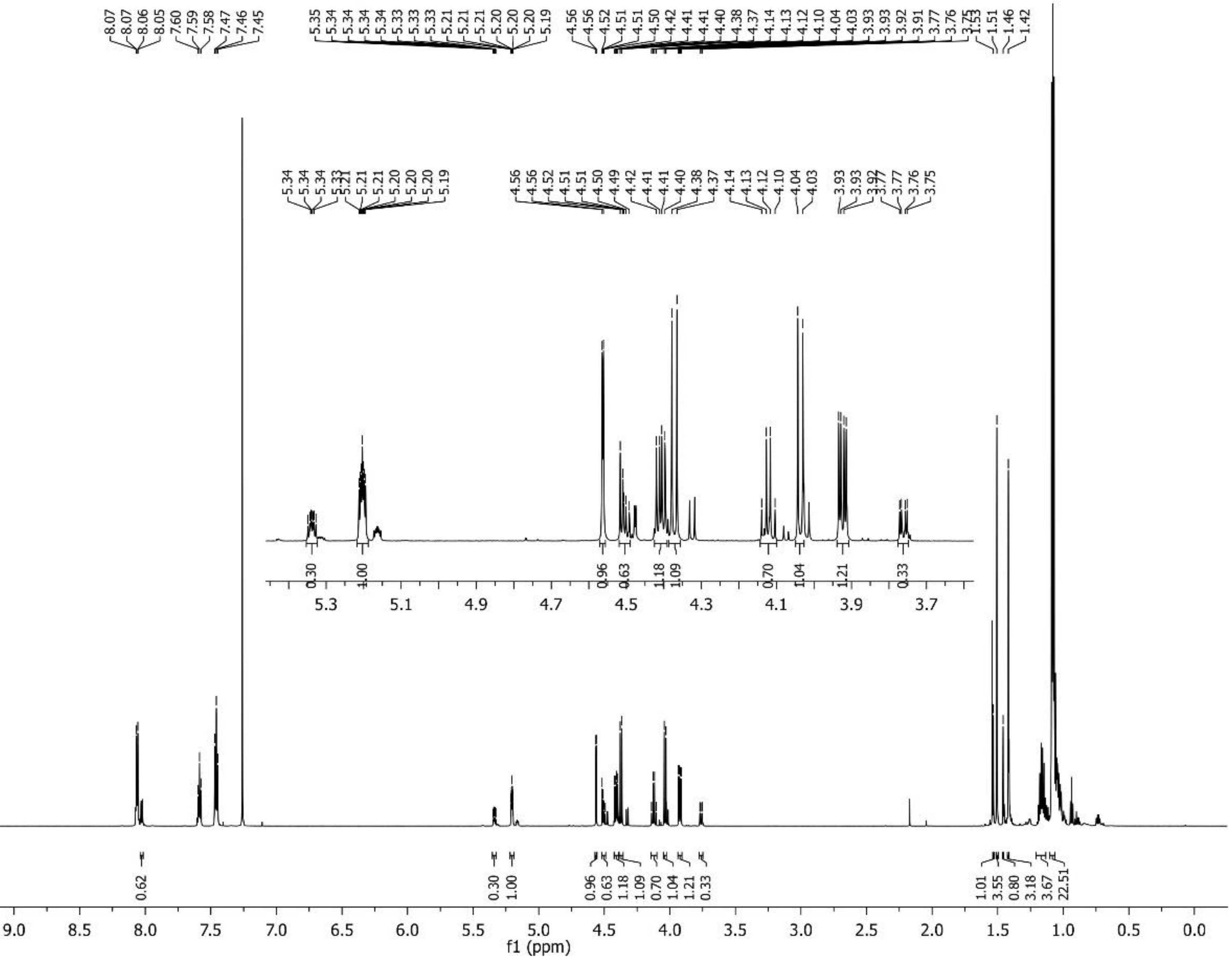

^{13}C NMR (176 MHz, CDCl$_3$) of 4-*O*-benzoyl-1,2-*O*-isopropylidene-3-*O*-triisopropylsilyl-D-xylulose (33)

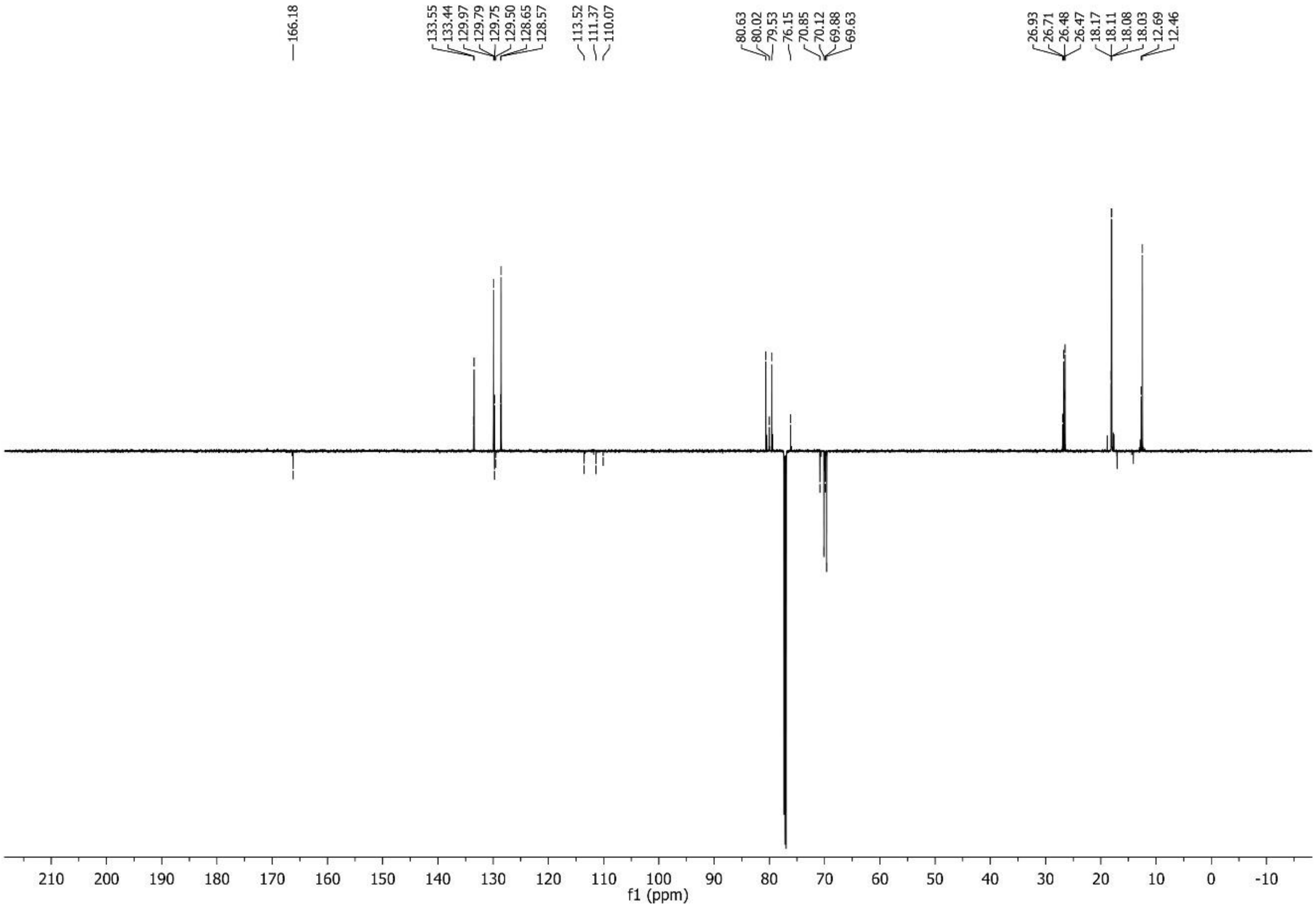

^{1}H NMR (700 MHz, $CDCl_3$) of 4-*O*-benzoyl-1,2-*O*-isopropylidene-D-xylulose (34)

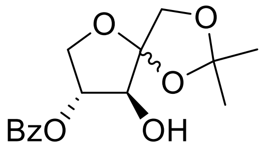

34

$C_{15}H_{18}O_6$

294.30 g/mol

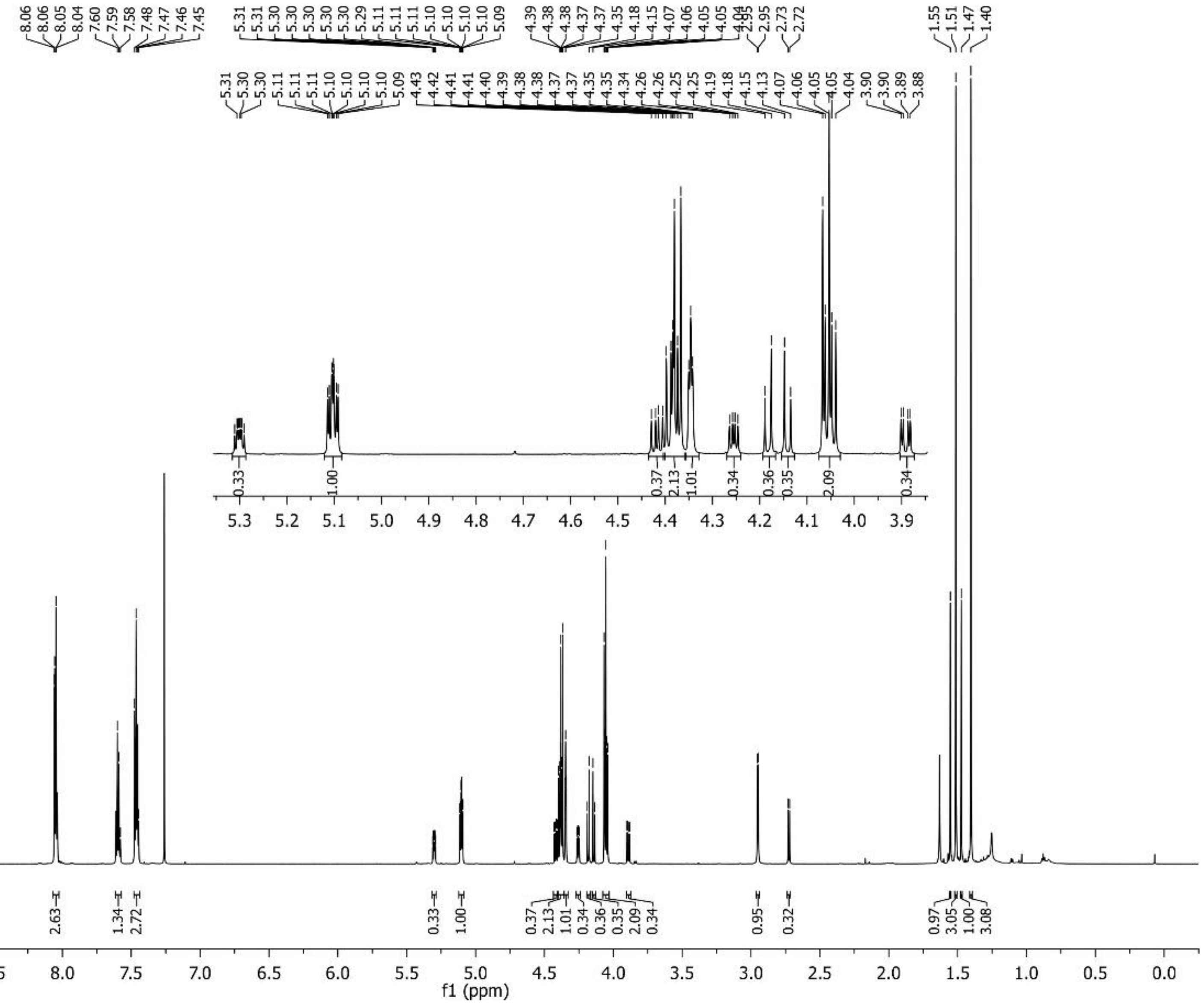

^{13}C NMR (176 MHz, $CDCl_3$) of 4-*O*-benzoyl-1,2-*O*-isopropylidene-D-xylulose (34)

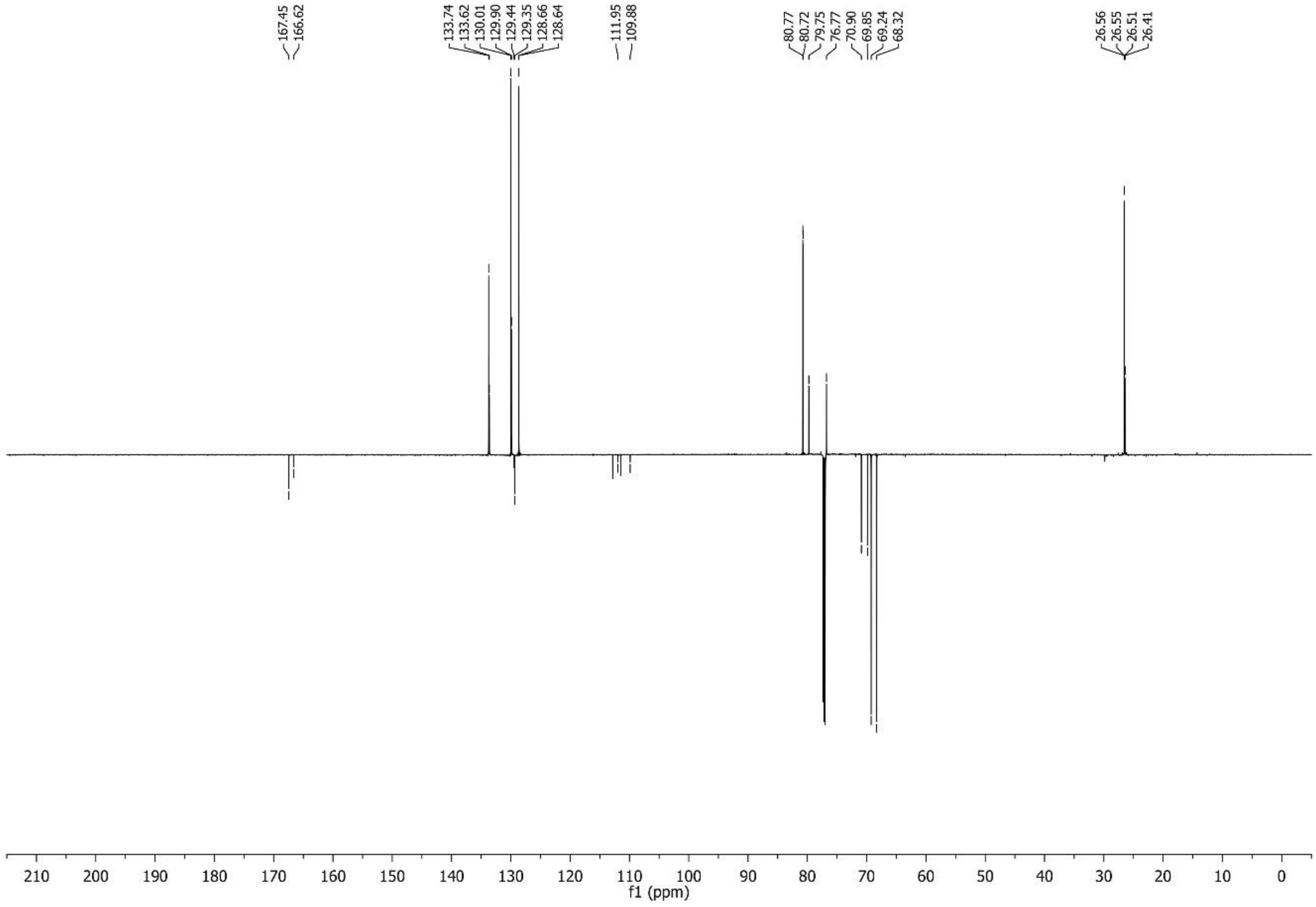

H NMR (700 MHz, $CDCl_3$) of 4-*O*-benzoyl-1,2-*O*-isopropylidene-3-*O*-trifluoromethanesulfonyl-D-xylulose (35)

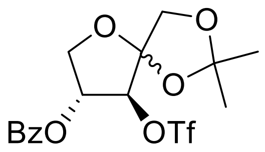

35

$C_{16}H_{17}F_3O_8S$

426.36 g/mol

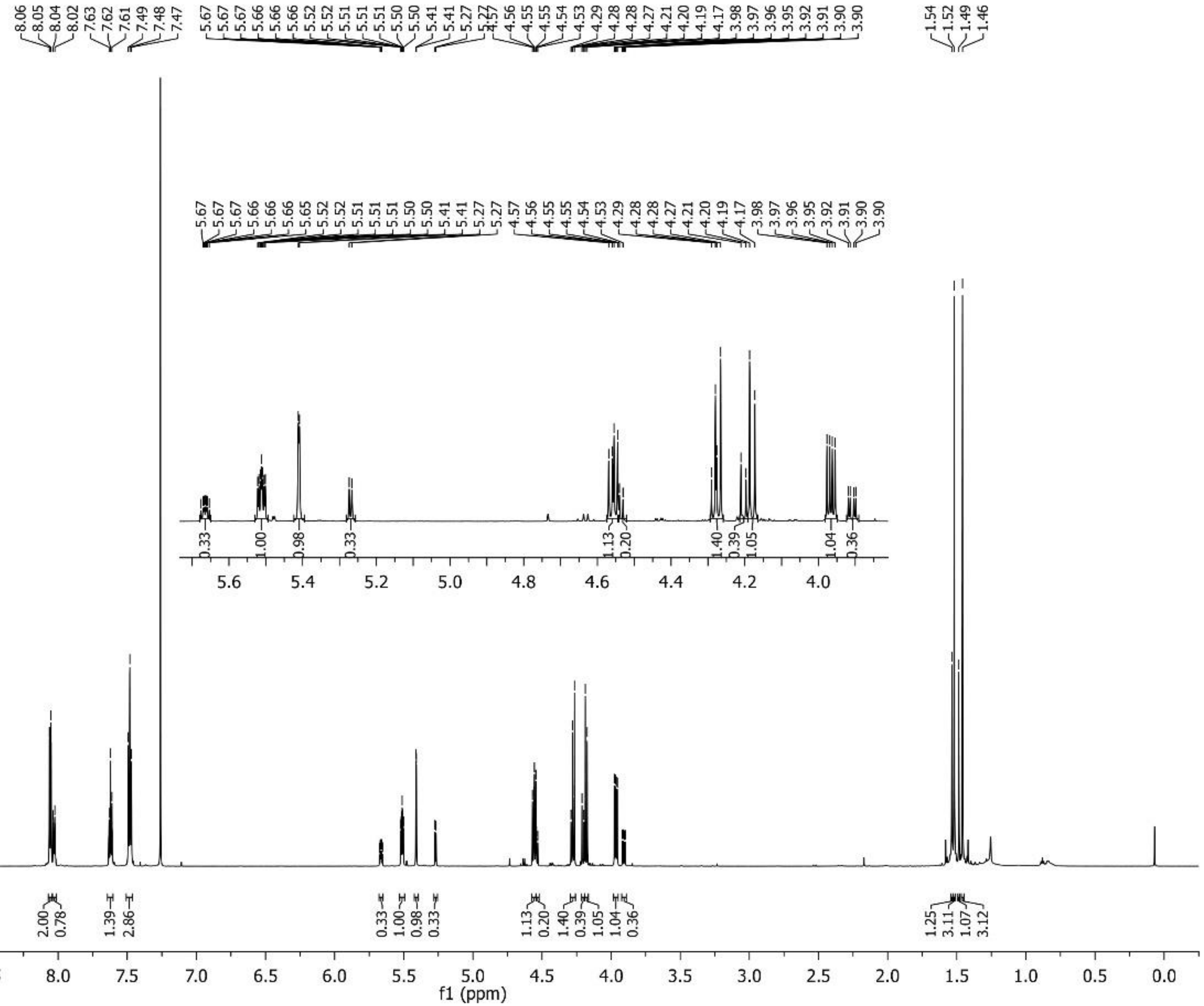

^{13}C NMR (176 MHz, CDCl$_3$) of 4-*O*-benzoyl-1,2-*O*-isopropylidene-3-*O*-trifluoromethanesulfonyl-D-xylulose (35)

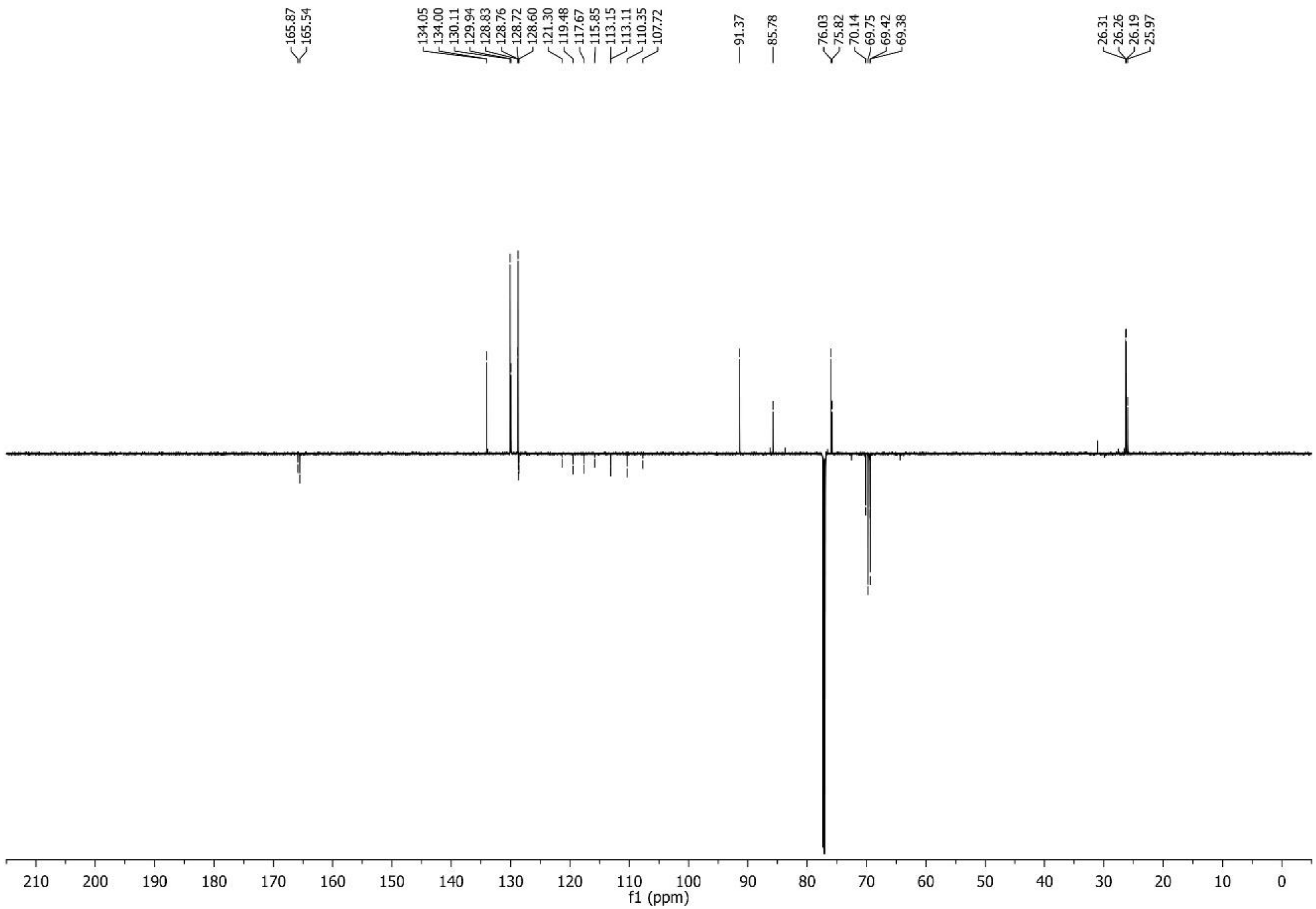

^{19}F NMR (659 MHz, CDCl$_3$) of 4-*O*-benzoyl-1,2-*O*-isopropylidene-3-*O*-trifluoromethanesulfonyl-D-xylulose (35)

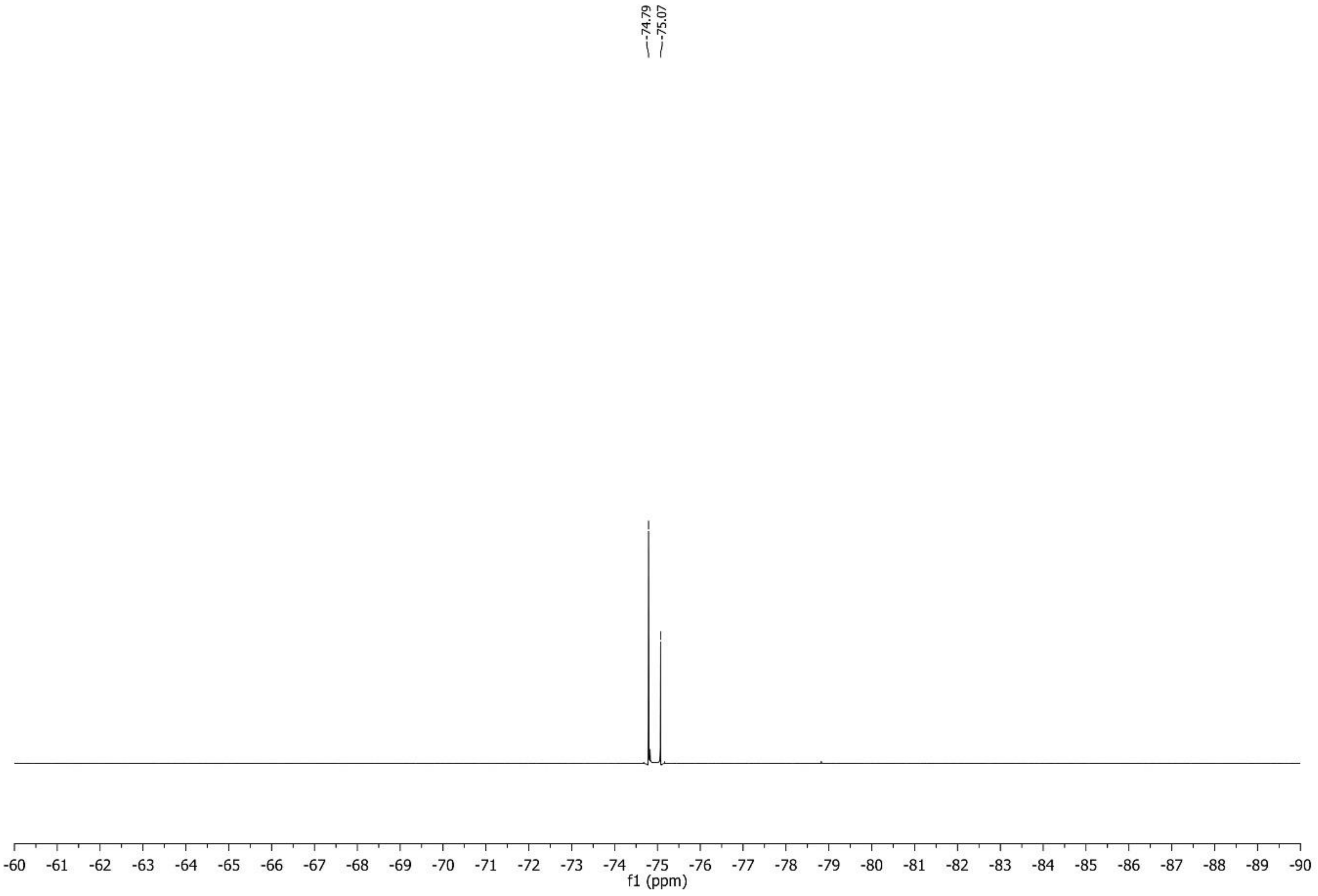

^{1}H NMR (700 MHz, $CDCl_3$) of 2-*O*-*para*-toluenesulfunyl-D-*erythrono*-1,4-lactone (36)

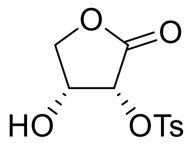

36

$C_{11}H_{12}O_6S$

272.27 g/mol

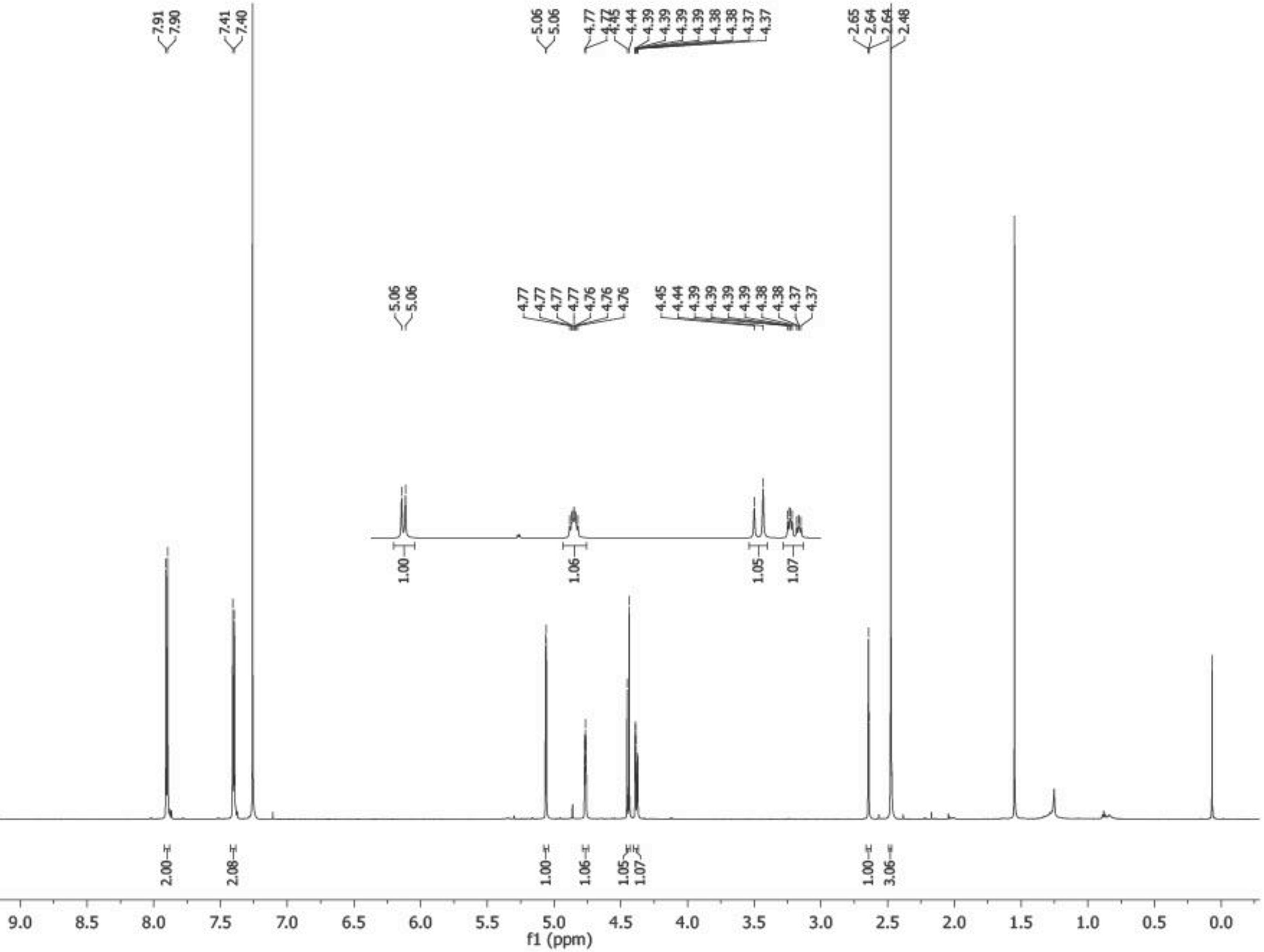

^{13}C NMR (176 MHz, CDCl$_3$) of 2-*O*-*para*-toluenesulfunyl-D-*erythrono*-1,4-lactone (36)

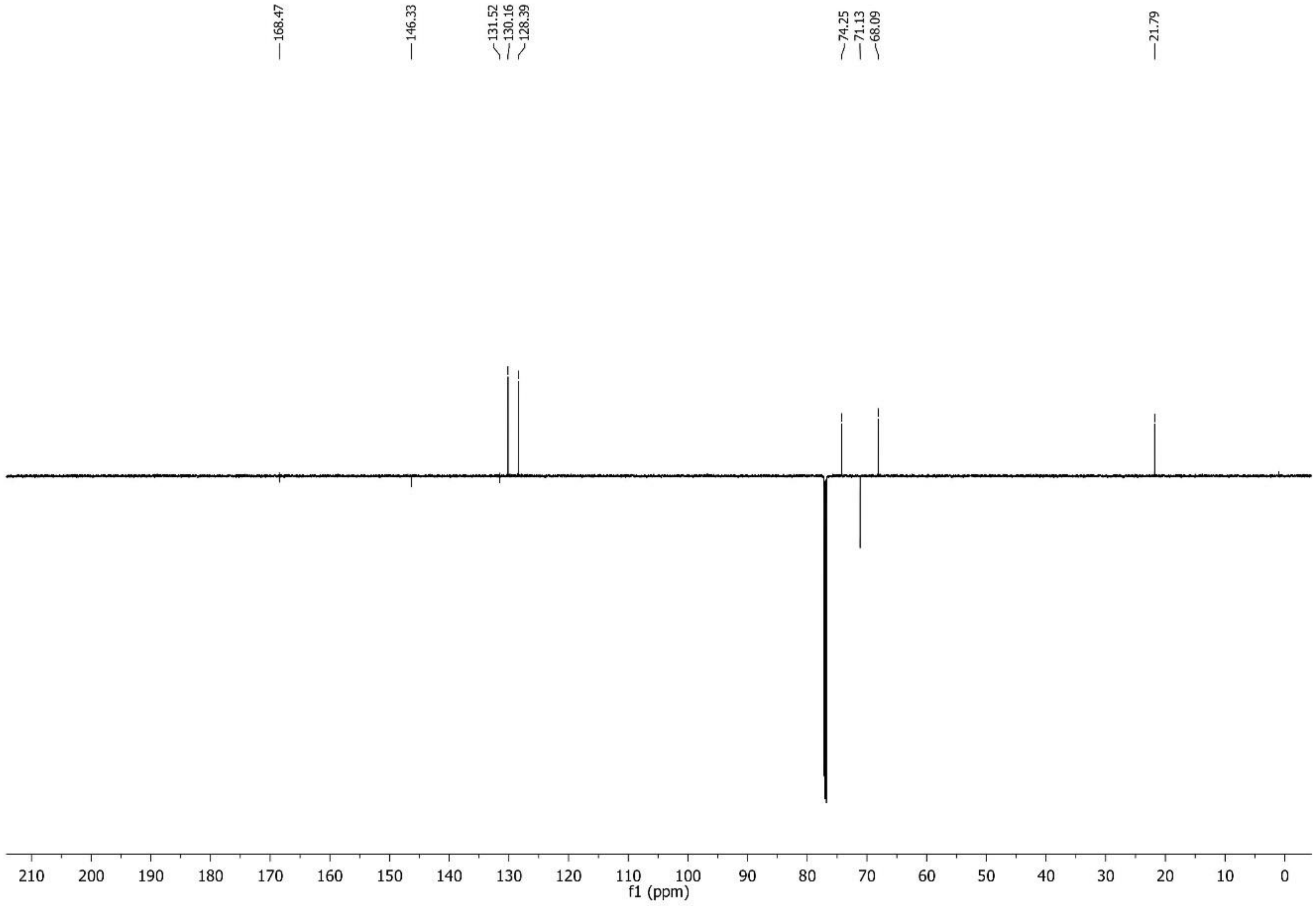

^{1}H NMR (600 MHz, $CDCl_3$) of 2,3-anhydro-D-*erythrono*-1,4-lactone (37)

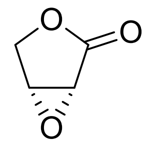

37

$C_4H_4O_3$

100.07 g/mol

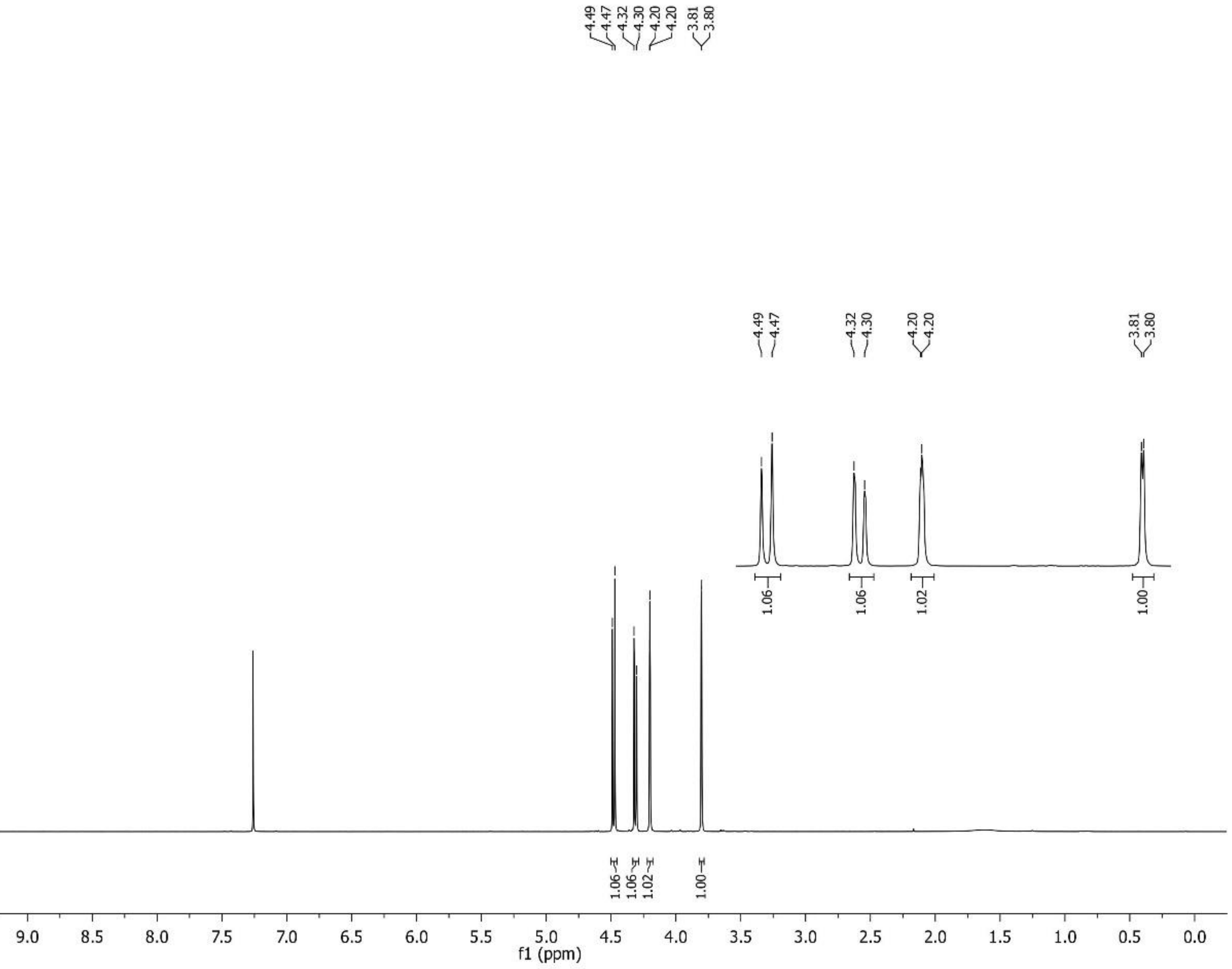

^{13}C NMR (151 MHz, $CDCl_3$) of 2,3-anhydro-D-*erythrono*-1,4-lactone (37)

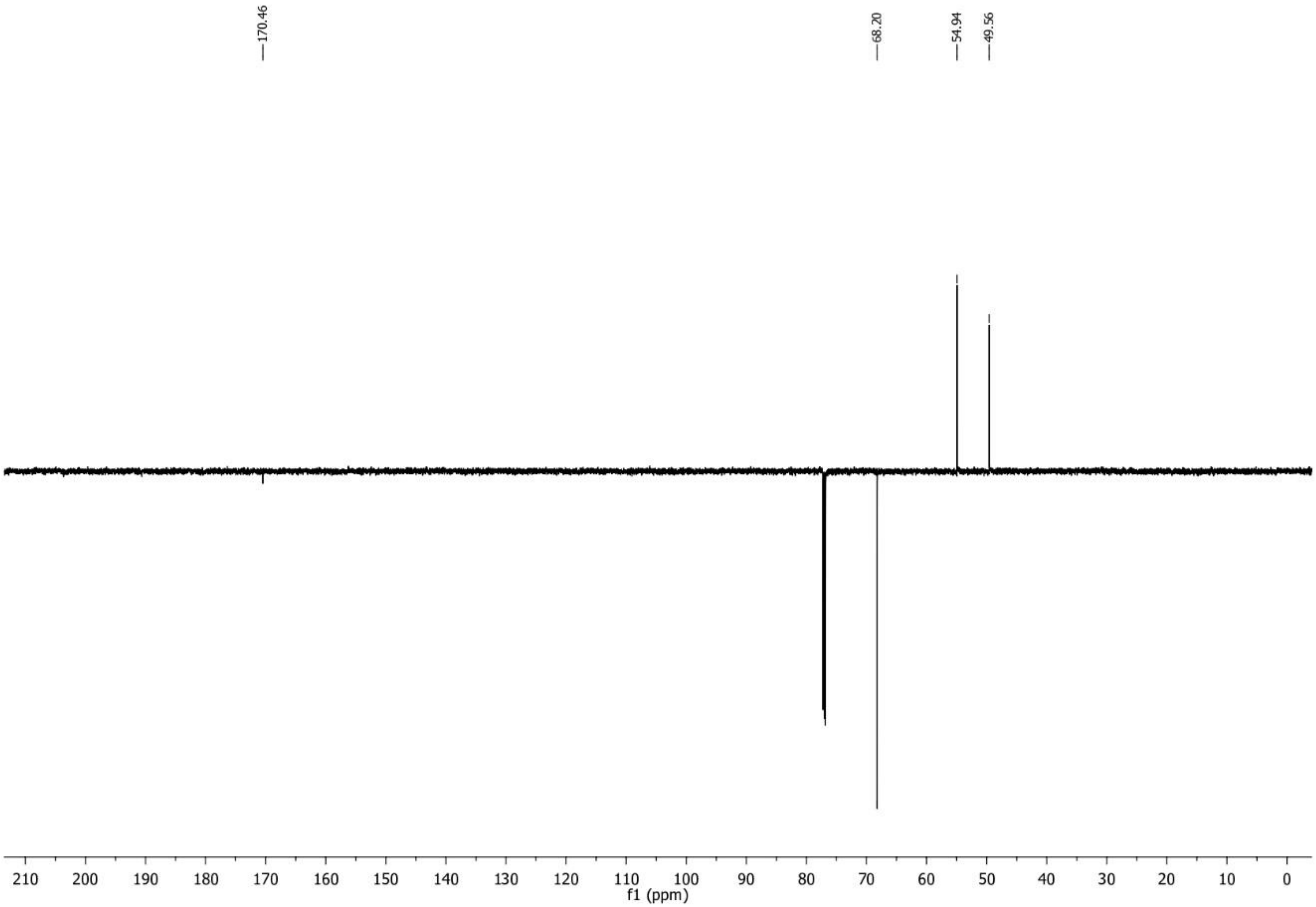

^{1}H NMR (700 MHz, $CDCl_3$) of 2-deoxy-2-fluoro-D-*threono*-1,4-lactone (38)

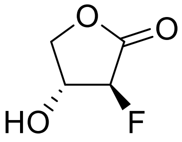

38

$C_4H_5FO_3$

120.08 g/mol

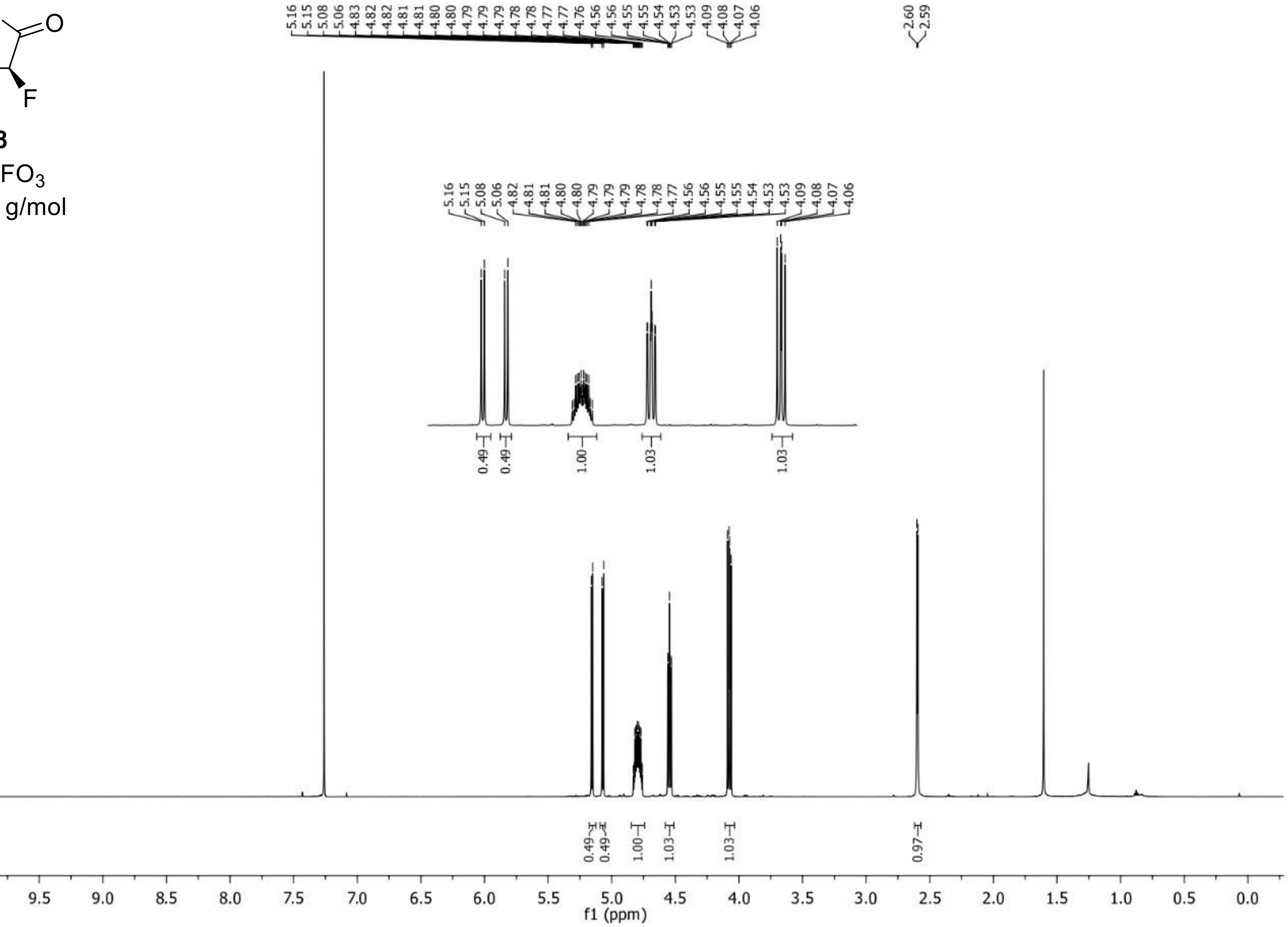

^{13}C NMR (176 MHz, $CDCl_3$) of 2-deoxy-2-fluoro-D-*threono*-1,4-lactone (38)

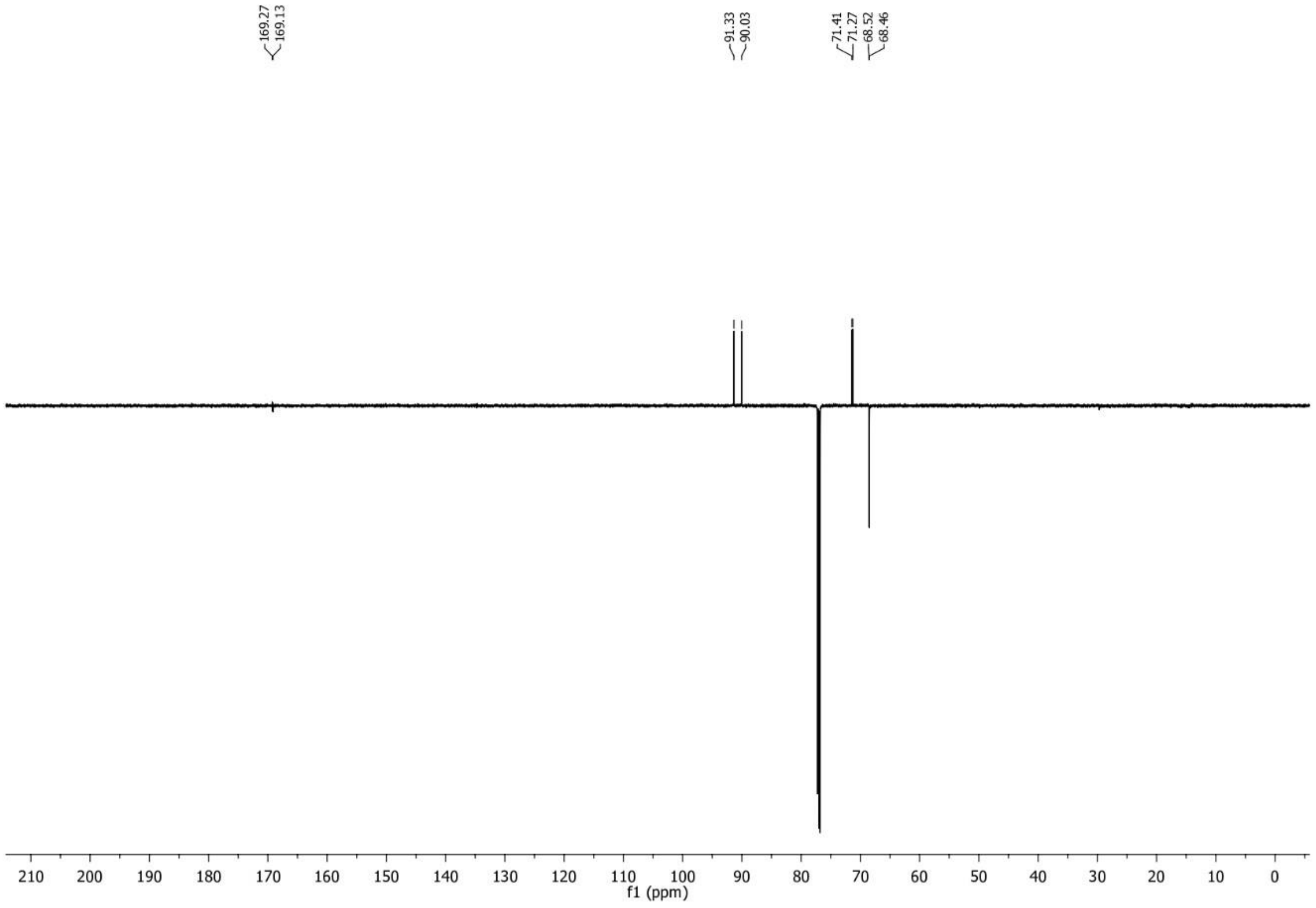

^{19}F NMR (659 MHz, $CDCl_3$) of 2-deoxy-2-fluoro-D-*threono*-1,4-lactone (38)

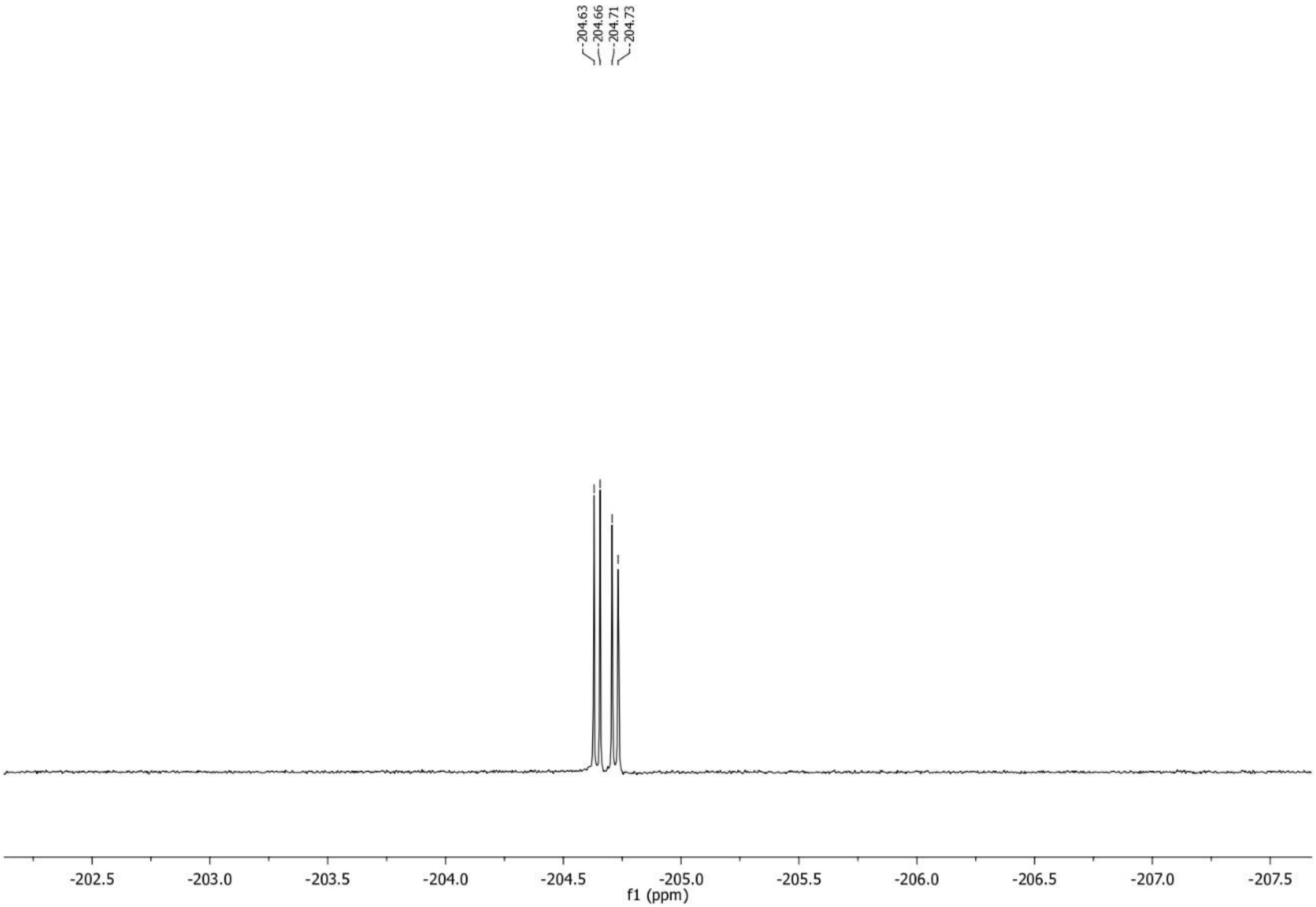

^{1}H NMR (600 MHz, $CDCl_3$) of 3-*O*-*tert*-butyldimethylsilyl-2-deoxy-2-fluoro-D-*threono*-1,4-lactone (39)

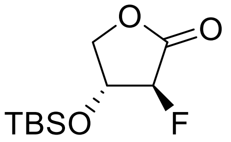

39

$C_{10}H_{19}FO_3Si$

234.34 g/mol

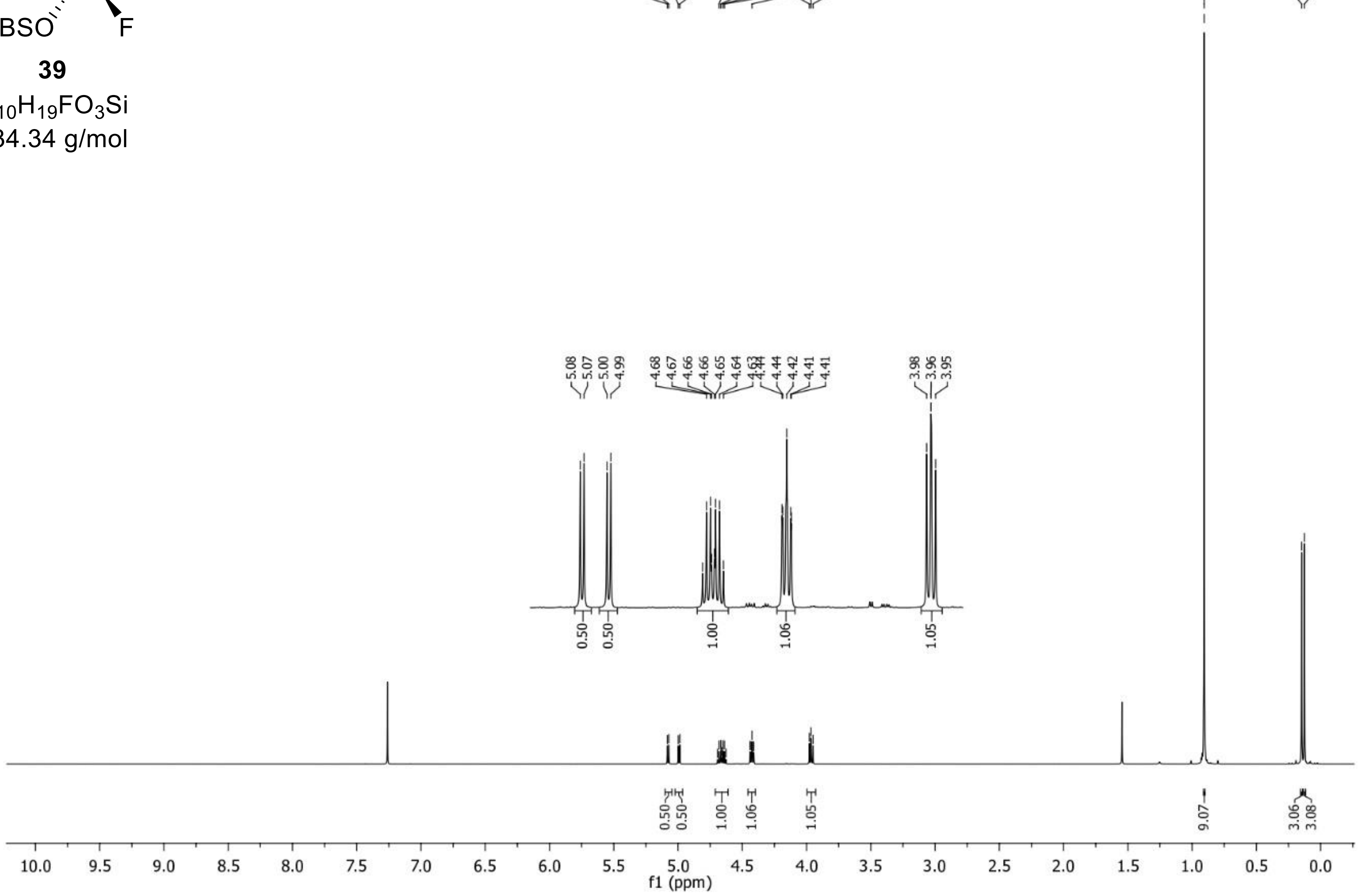

^{13}C NMR (151 MHz, CDCl$_3$) of 3-*O*-*tert*-butyldimethylsilyl-2-deoxy-2-fluoro-D-*threono*-1,4-lactone (39)

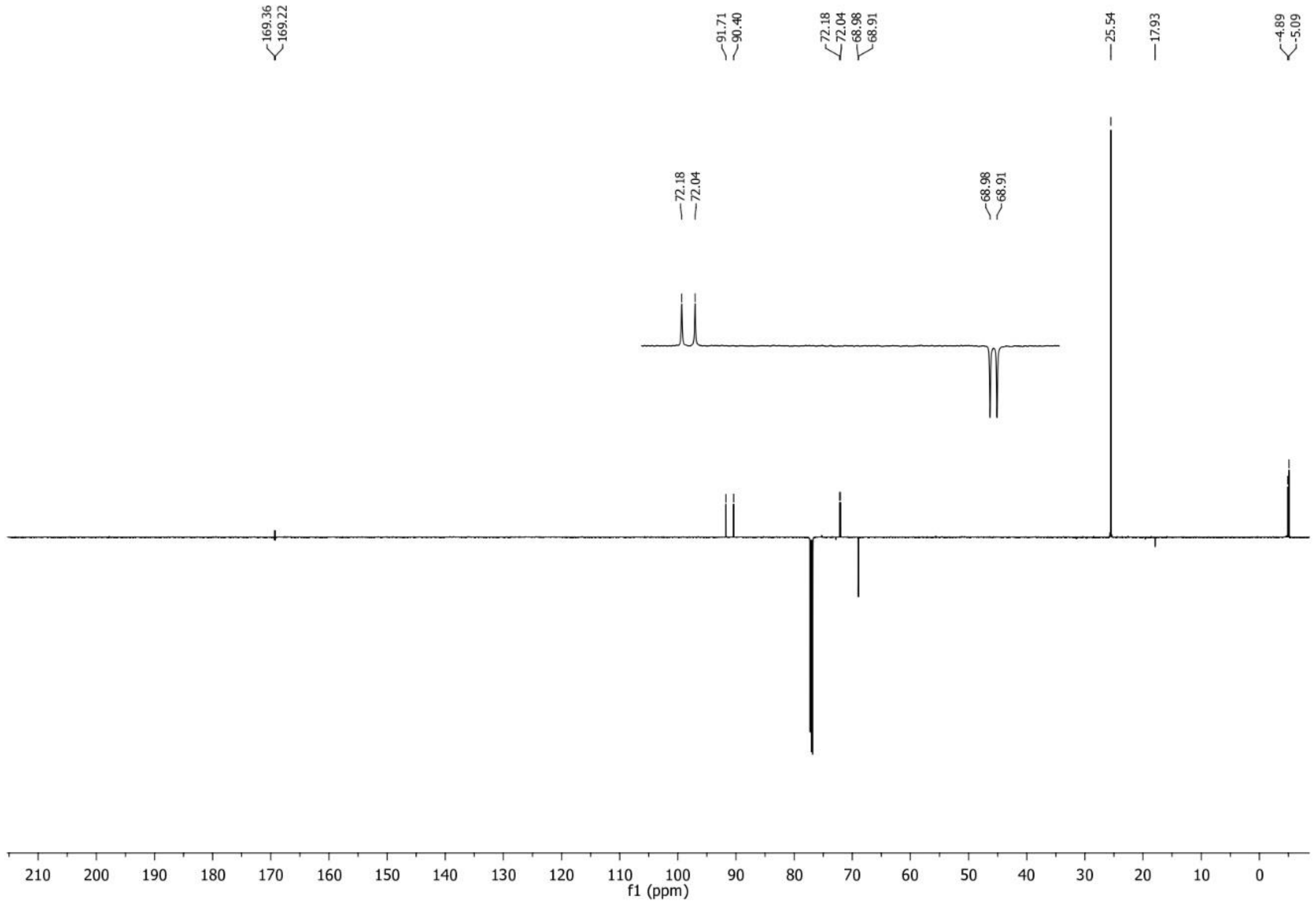

^{19}F NMR (400 MHz, $CDCl_3$) of 3-*O*-*tert*-butyldimethylsilyl-2-deoxy-2-fluoro-D-*threono*-1,4-lactone (39)

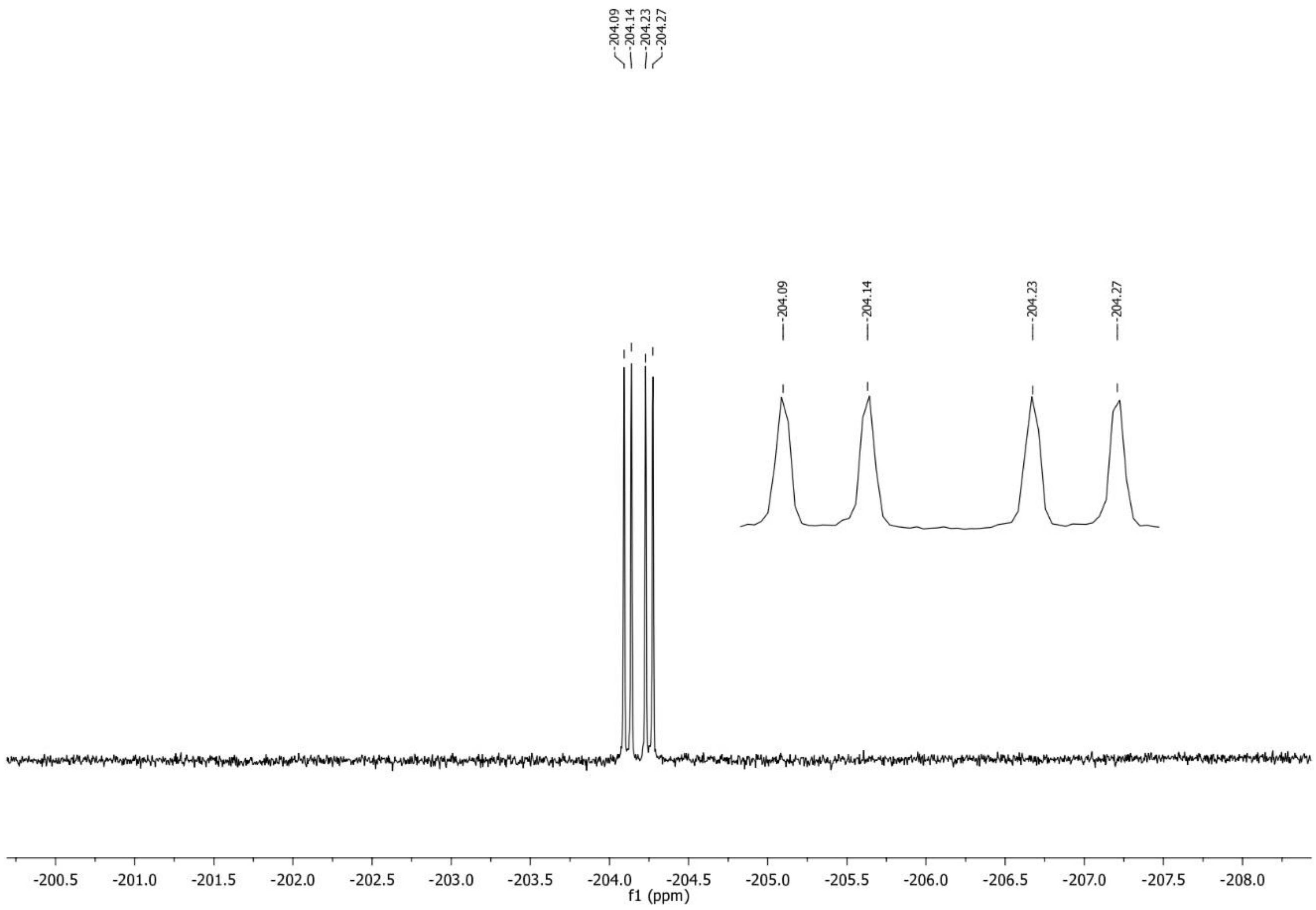

^{1}H NMR (700 MHz, CDCl$_3$) of 2,5-anhydro-4-*O*-*tert*-butyldimethylsilyl-1,3-dideoxy-3-fluoro-D-*threono*-pent-1-entiol (40)

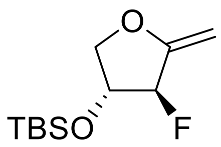

40

$C_{11}H_{21}FO_2Si$

232.37 g/mol

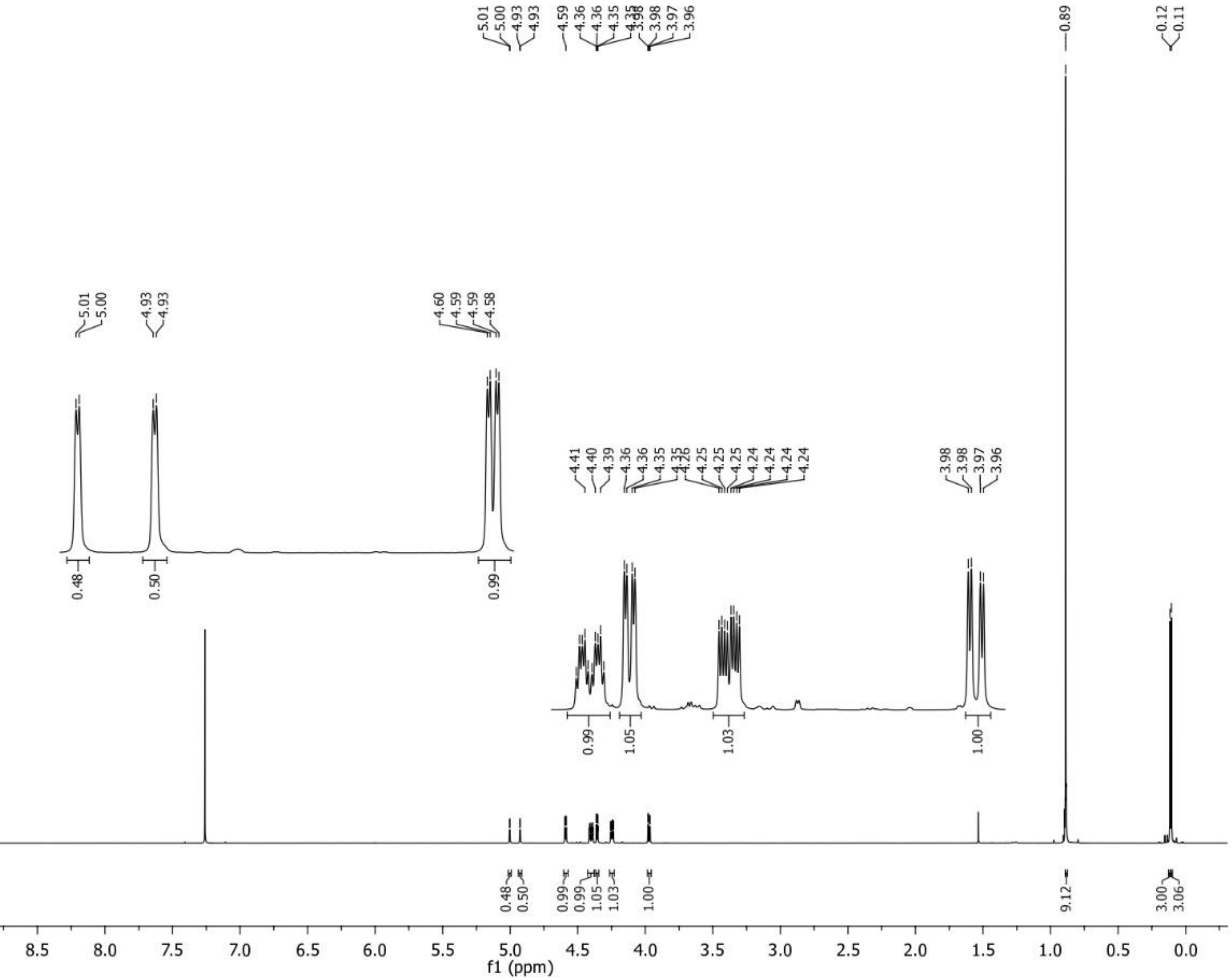

^{13}C NMR (176 MHz, $CDCl_3$) of 2,5-anhydro-4-*O*-*tert*-butyldimethylsilyl-1,3-dideoxy-3-fluoro-D-*threono*-pent-1-entiol (40)

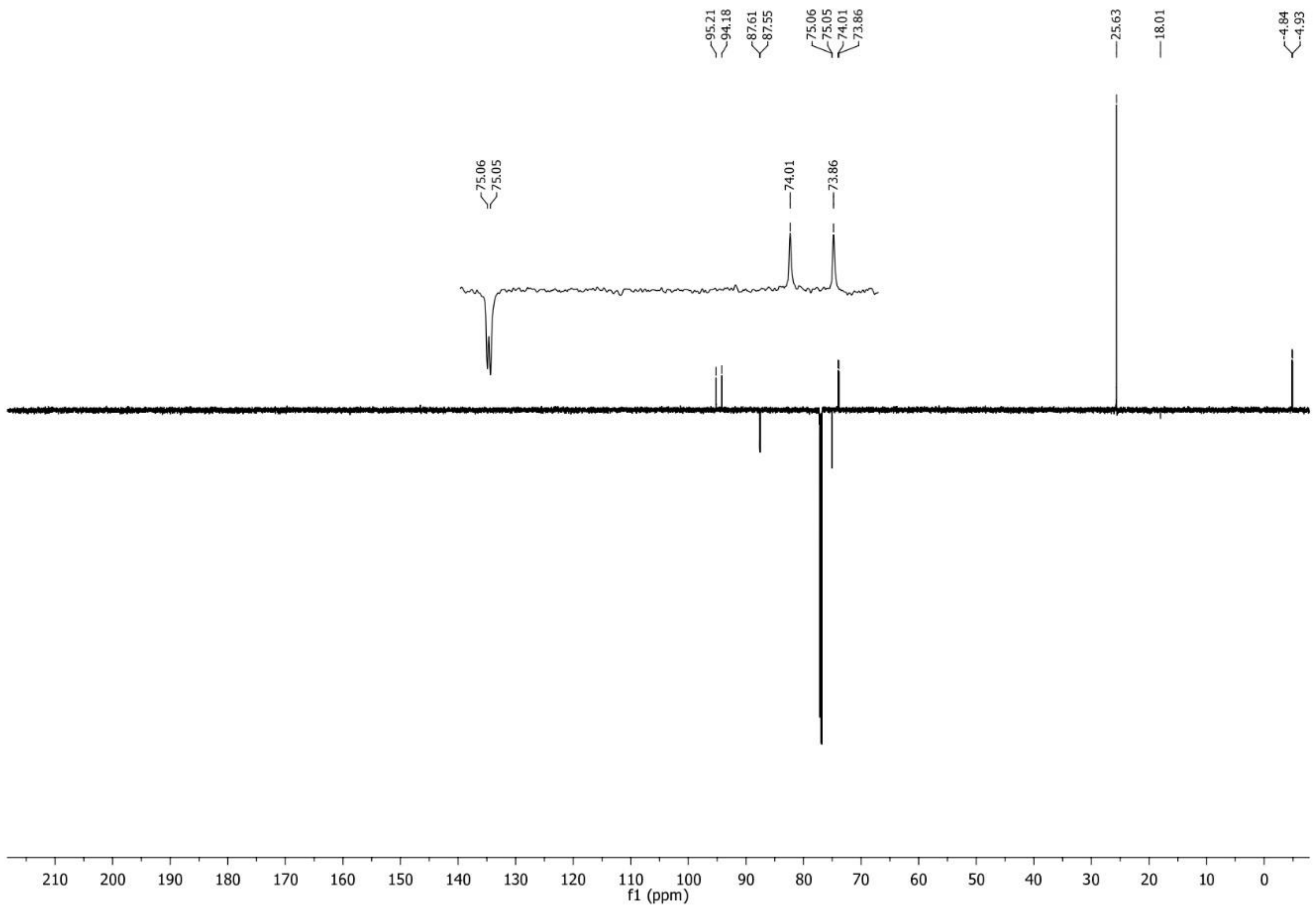

^{19}F NMR (659 MHz, $CDCl_3$) of 2,5-anhydro-4-*O*-*tert*-butyldimethylsilyl-1,3-dideoxy-3-fluoro-D-*threono*-pent-1-entiol (40)

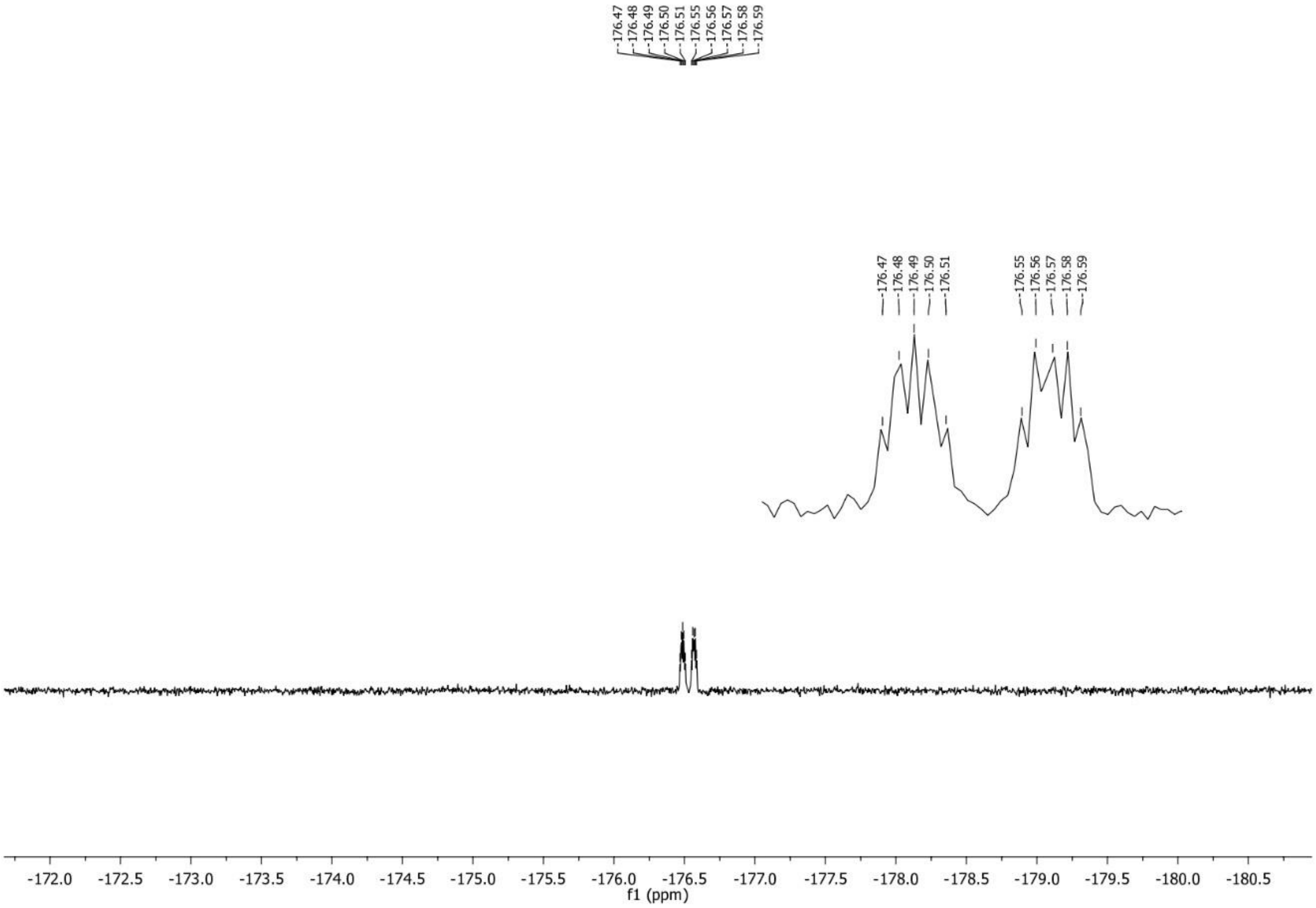

^{1}H NMR (600 MHz, $CDCl_3$) of 4-*O*-*tert*-butyldimethylsilyl-3-deoxy-3-fluoro-D-*xylulose* (41)

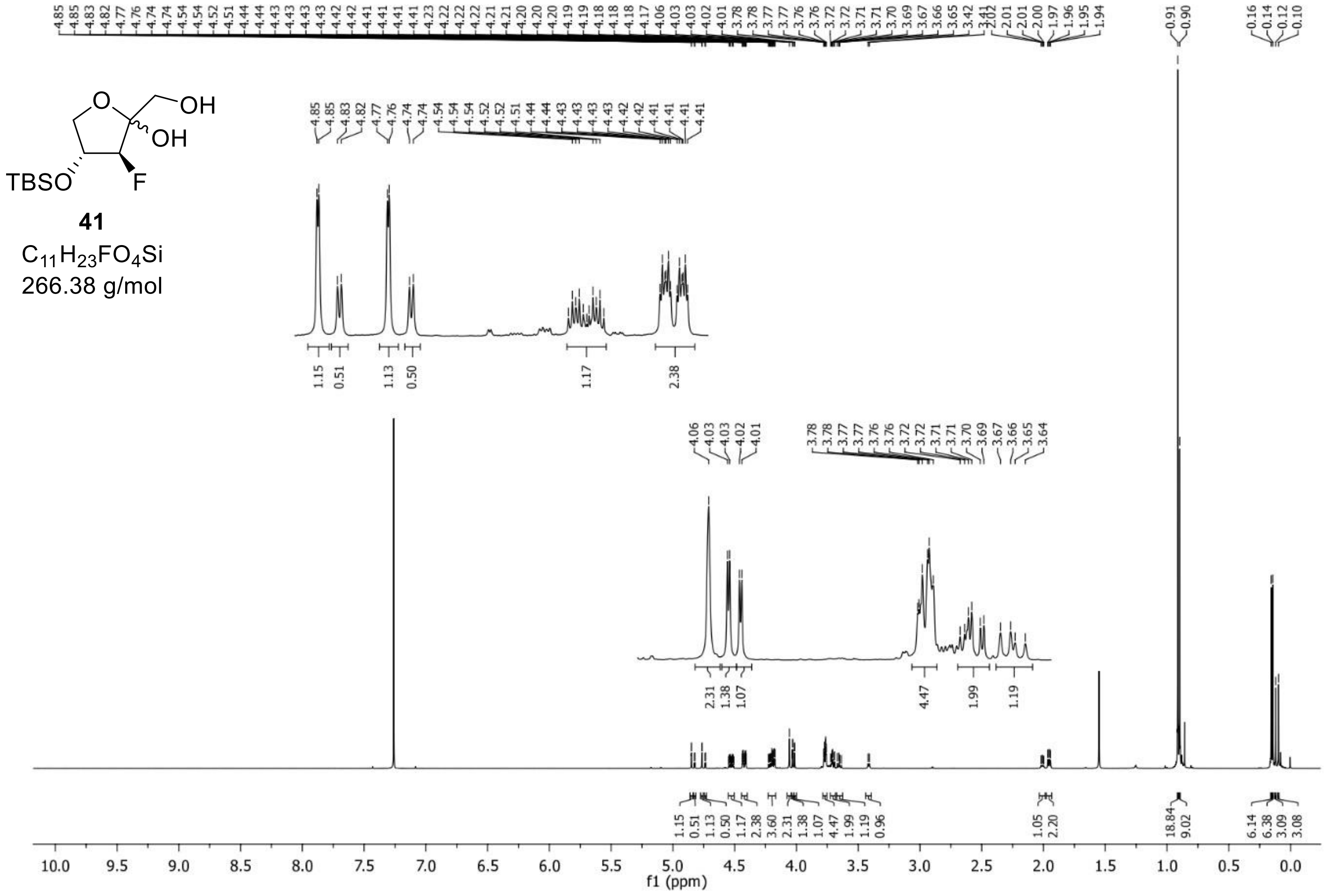

^{13}C NMR (151 MHz, $CDCl_3$) of 4-*O-tert*-butyldimethylsilyl-3-deoxy-3-fluoro-D-*xylulose* (41)

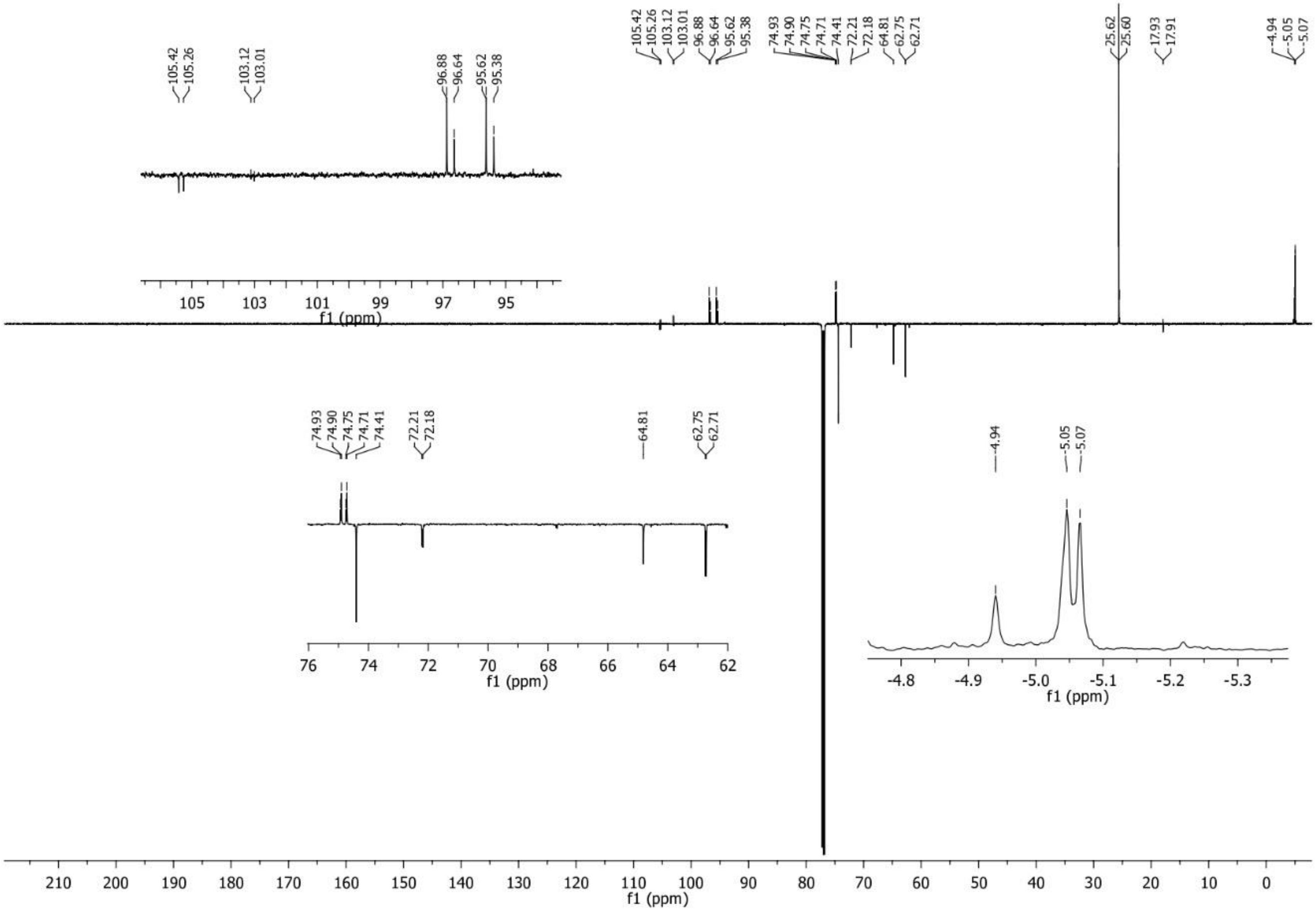

^{19}F NMR (659 MHz, CDCl$_3$) of 4-*O*-*tert*-butyldimethylsilyl-3-deoxy-3-fluoro-D-*xylulose* (41)

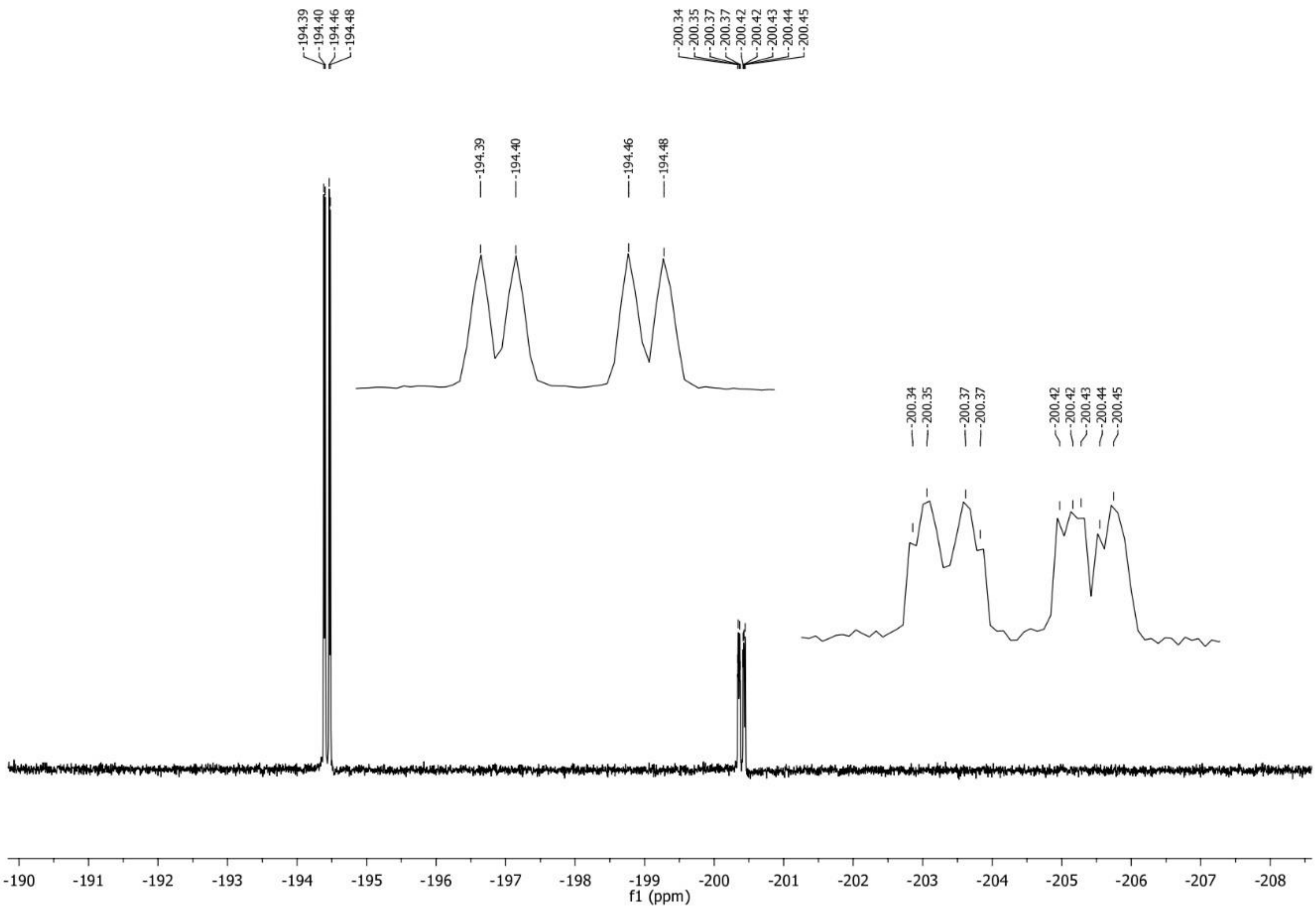

^{1}H NMR (700 MHz, D_2O) of 3-deoxy-3-fluoro-D-xylulose (3DFXu, 5)

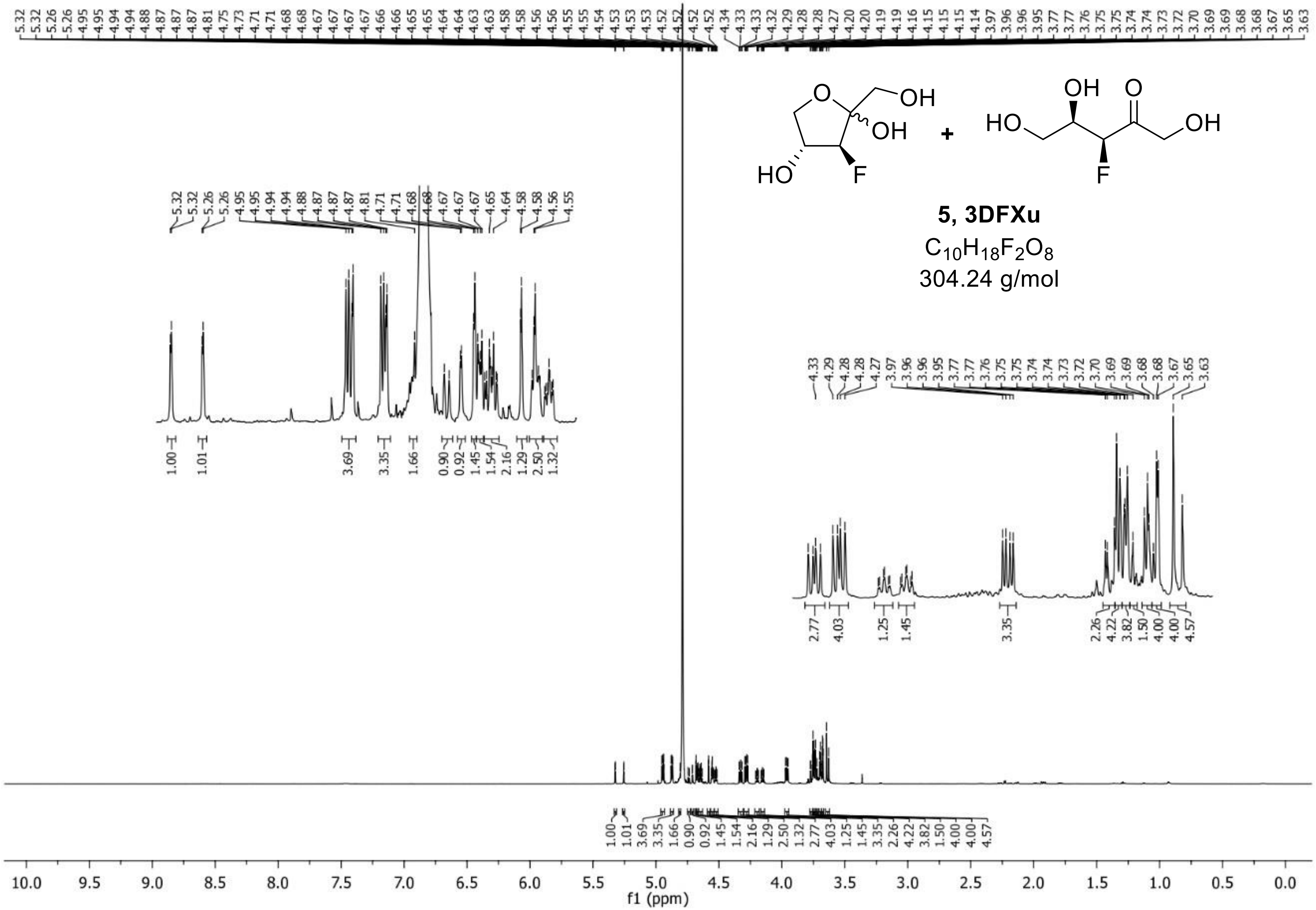

^{13}C NMR (176 MHz, D_2O) of 3-deoxy-3-fluoro-D-*xylulose* (3DFXu, 5)

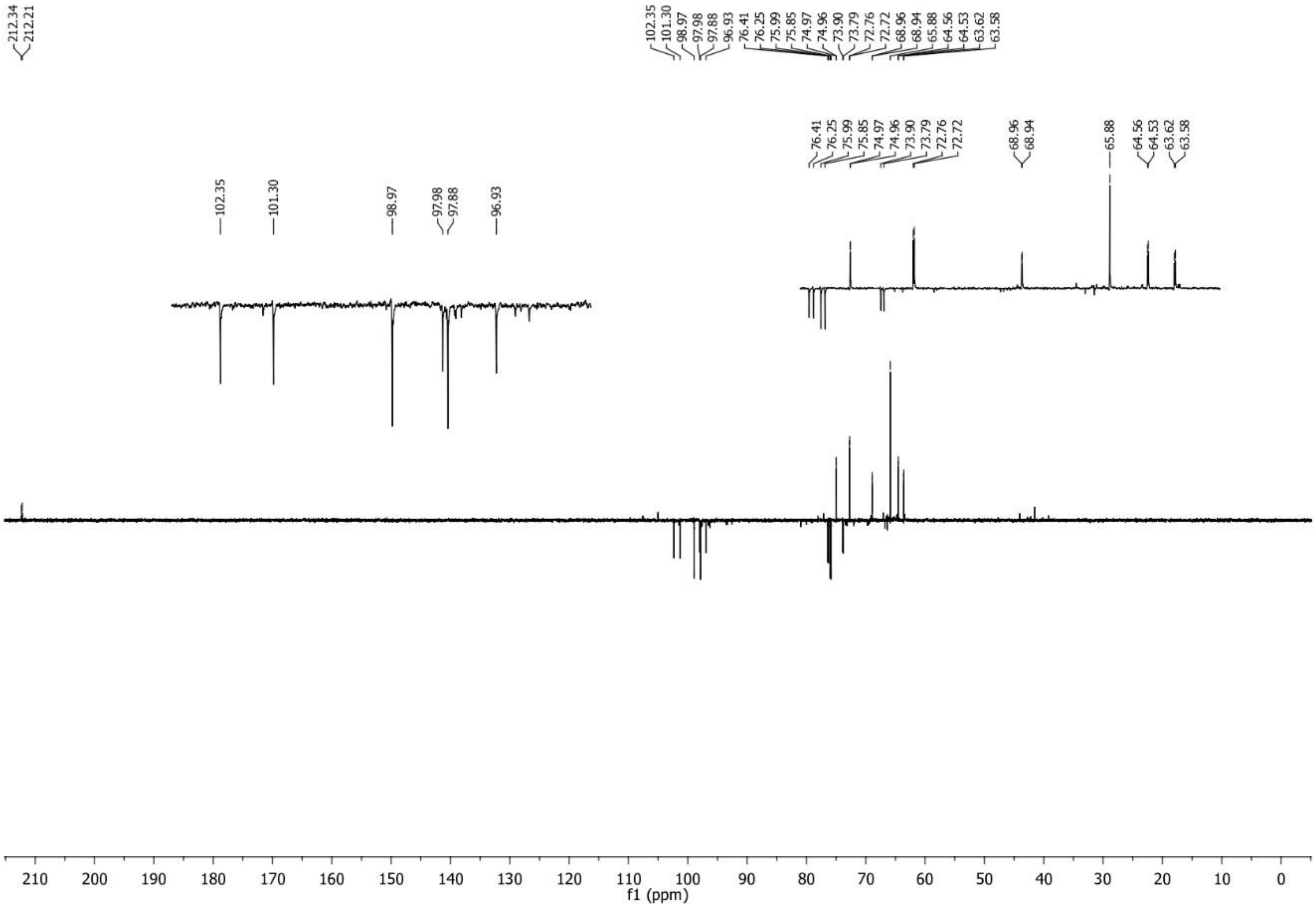

^{19}F NMR (659 MHz, D_2O) of 3-deoxy-3-fluoro-D-*xylulose* (3DFXu, 5)

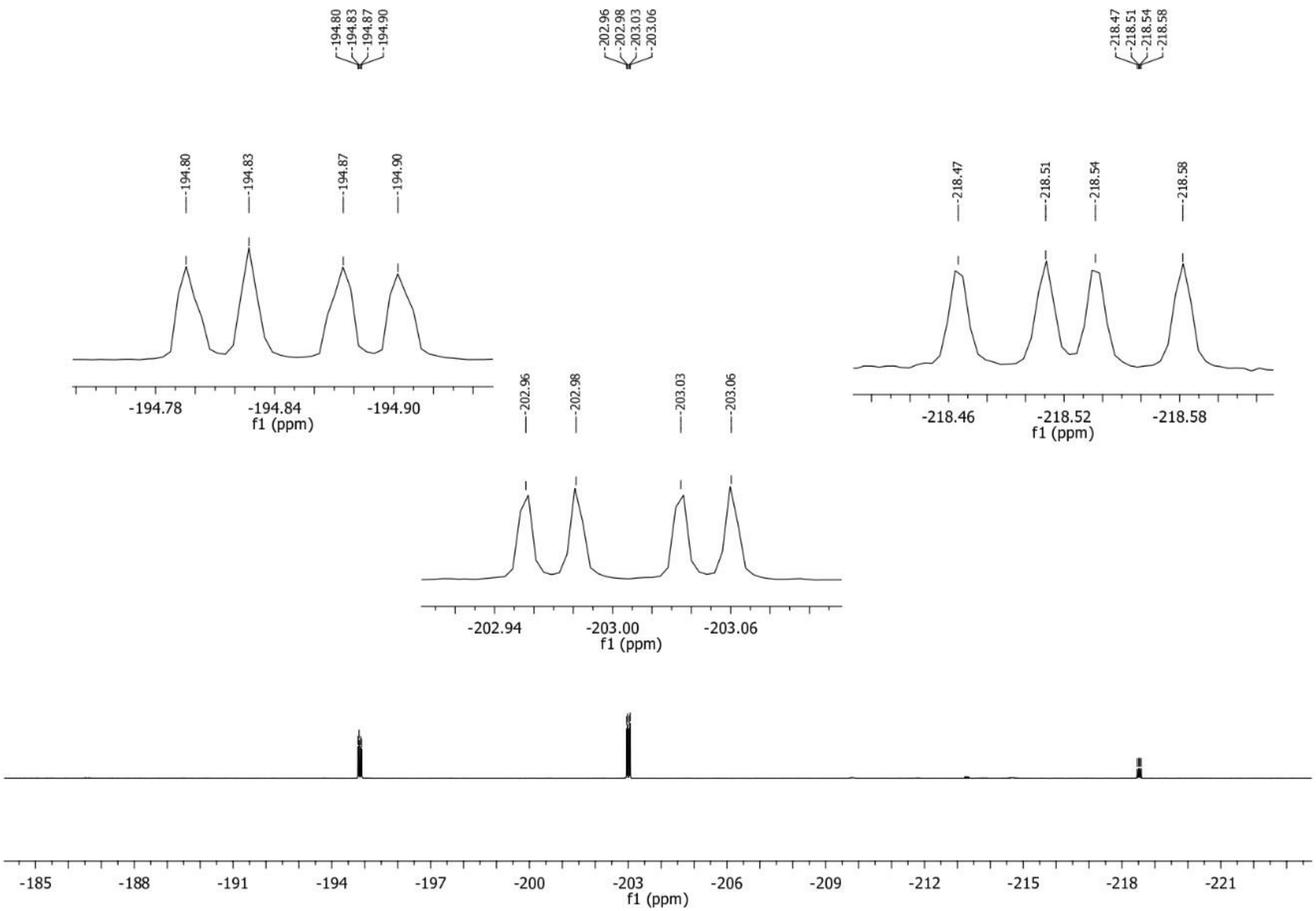

^{1}H NMR (700 MHz, $CDCl_3$) of 2-*O*-triisopropylsilyl-D-*erythrono*-1,4-lactone (42)

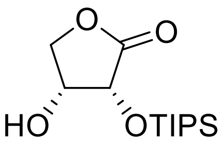

42

$C_{13}H_{26}O_4Si$

274.43 g/mol

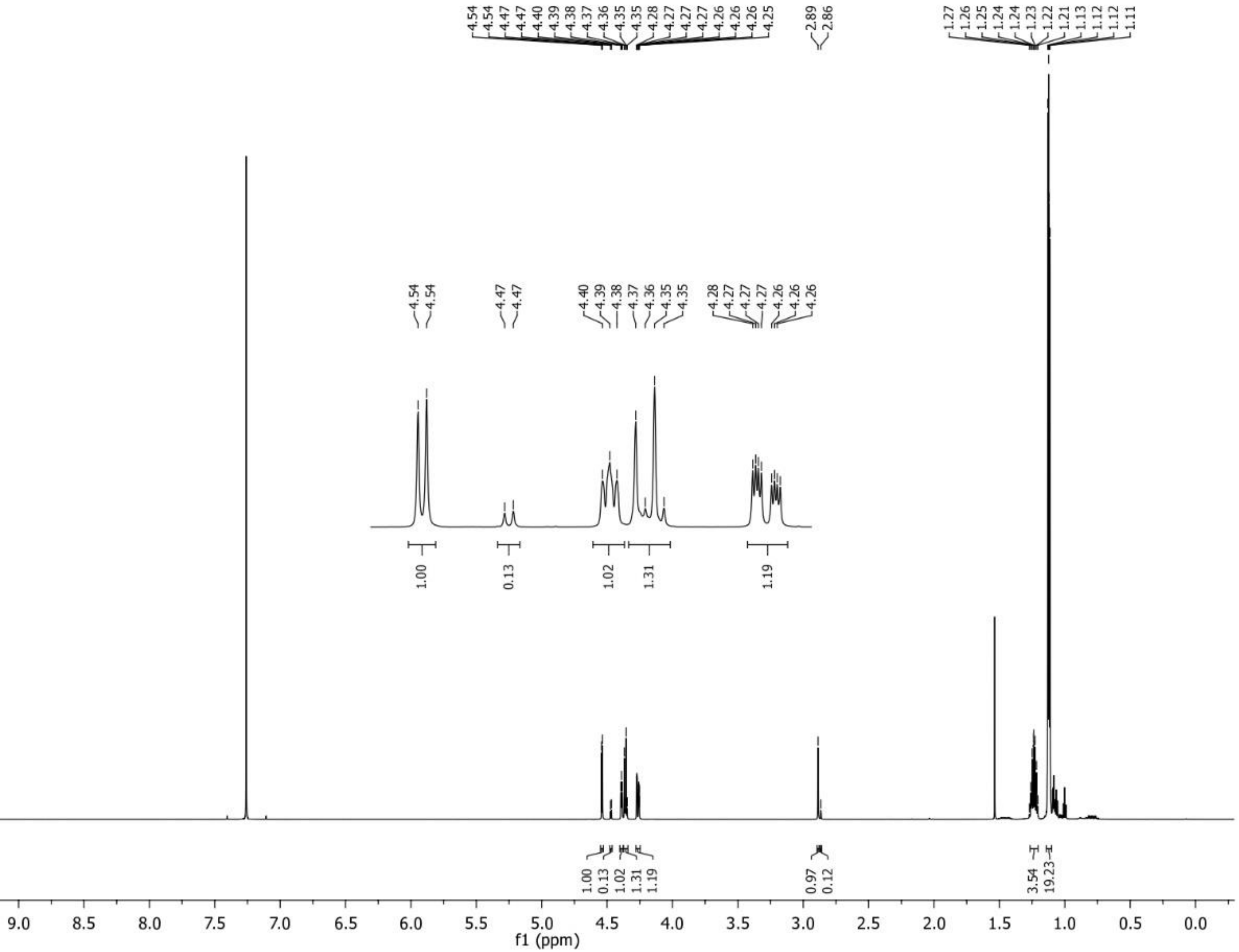

^{13}C NMR (176 MHz, $CDCl_3$) of 2-*O*-triisopropylsilyl-D-*erythrono*-1,4-lactone (42)

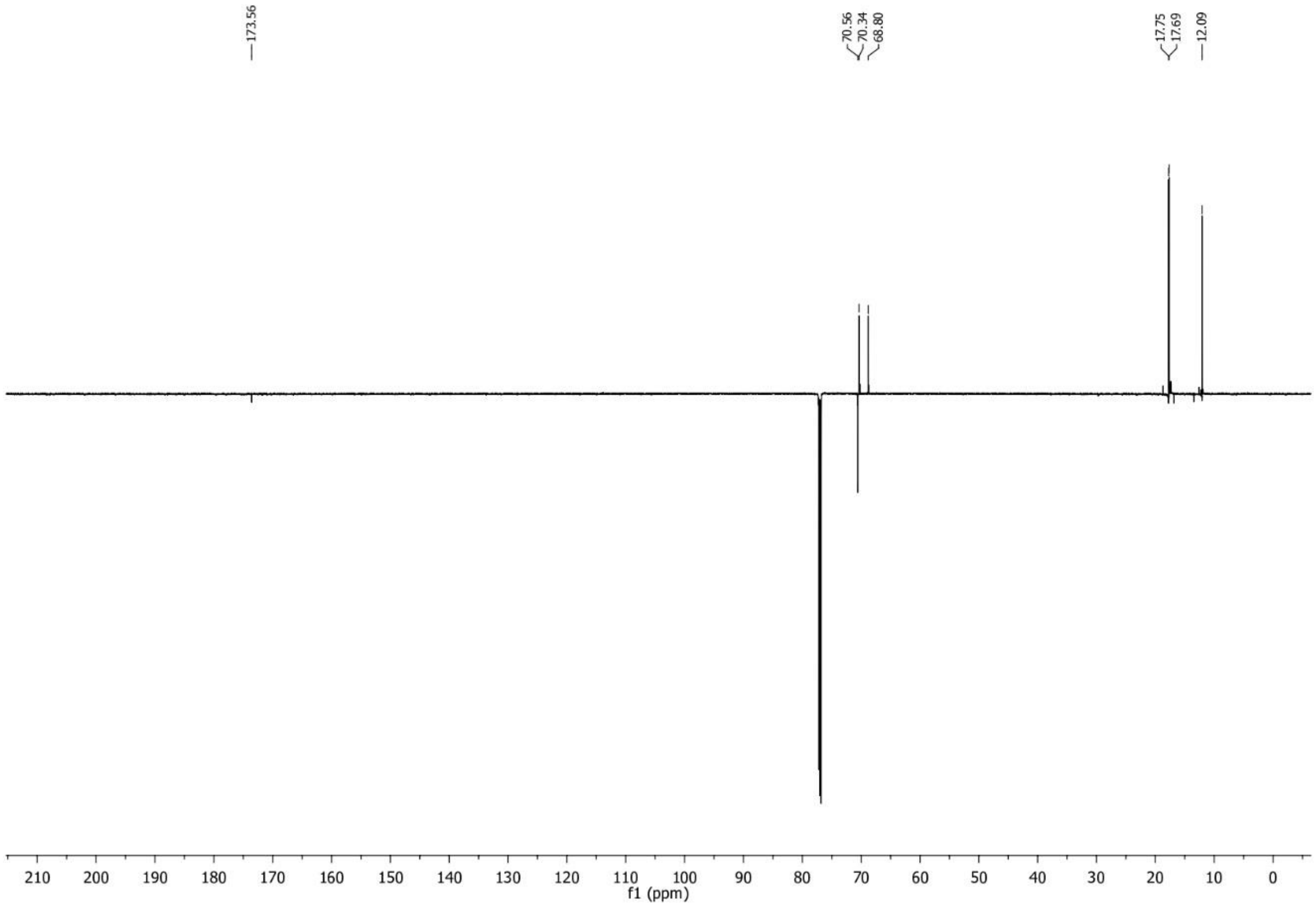

^{1}H NMR (700 MHz, CDCl3) of 3-*O*-benzoyl-2-*O*-triisopropylsilyl-D-*erythrono*-1,4-lactone (43)

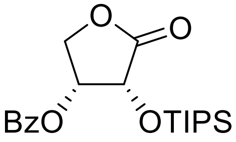

43

$C_{20}H_{30}O_5Si$

378.54 g/mol

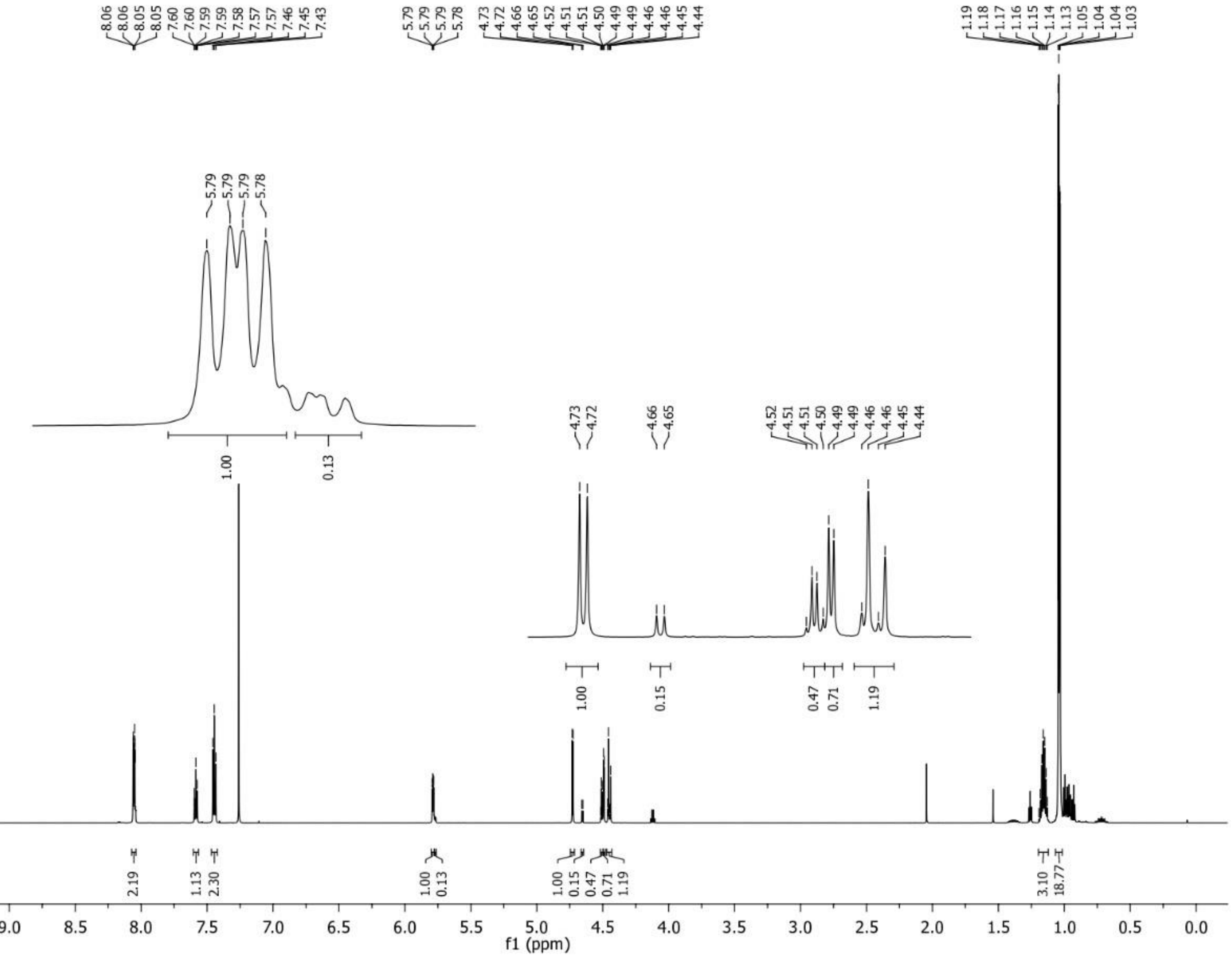

^{13}C NMR (176 MHz, $CDCl_3$) of 3-*O*-benzoyl-2-*O*-triisopropylsilyl-D-*erythrono*-1,4-lactone (43)

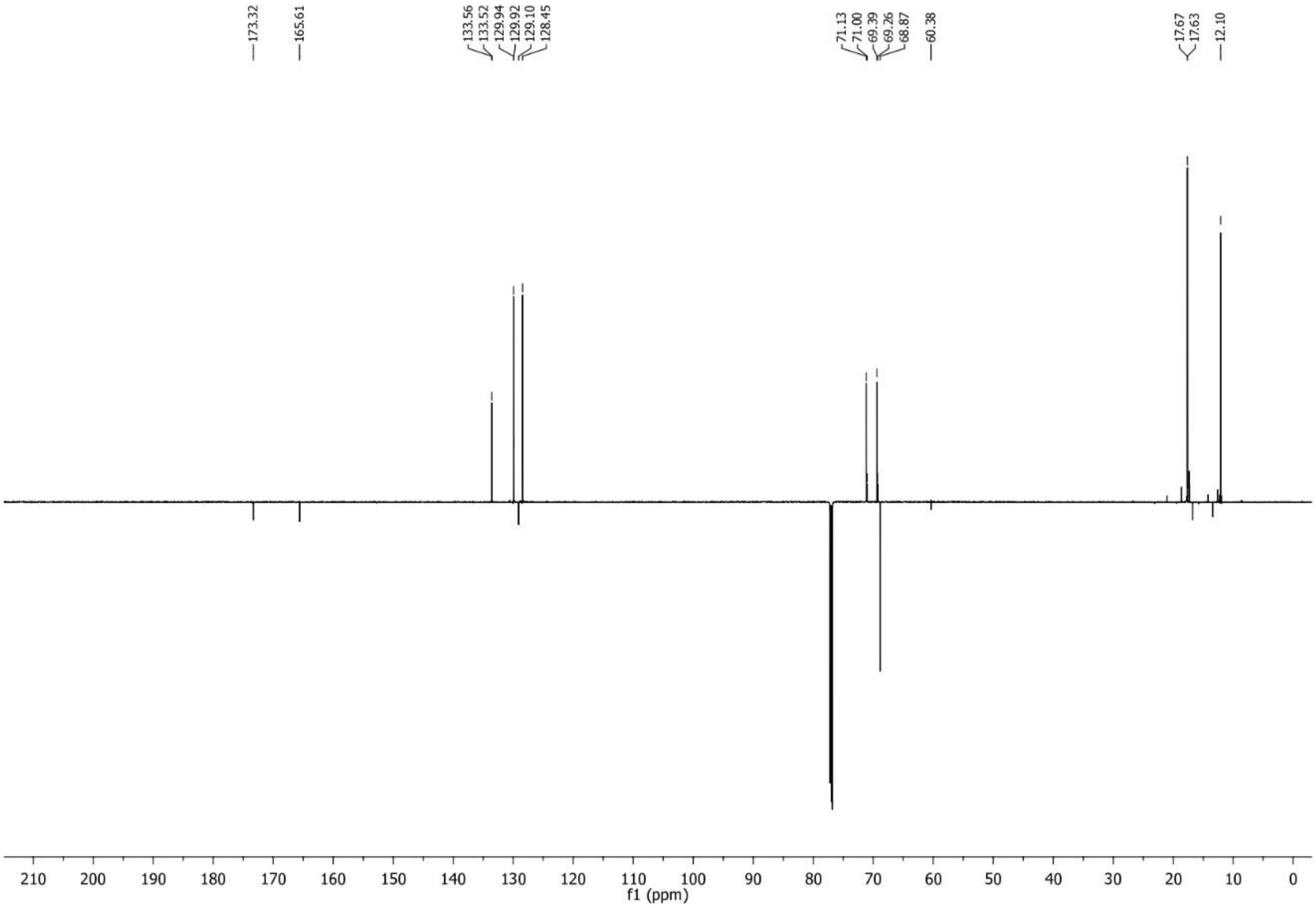

^{1}H NMR (600 MHz, $CDCl_3$) of 2,5-anhydro-4-*O*-benzoyl-1-deoxy-3-*O*-triisopropylsilyl-D-*erythro*-pent-1-entiol (44)

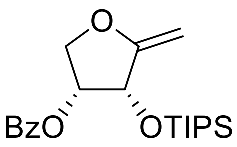

44

$C_{21}H_{32}O_4Si$

376.57 g/mol

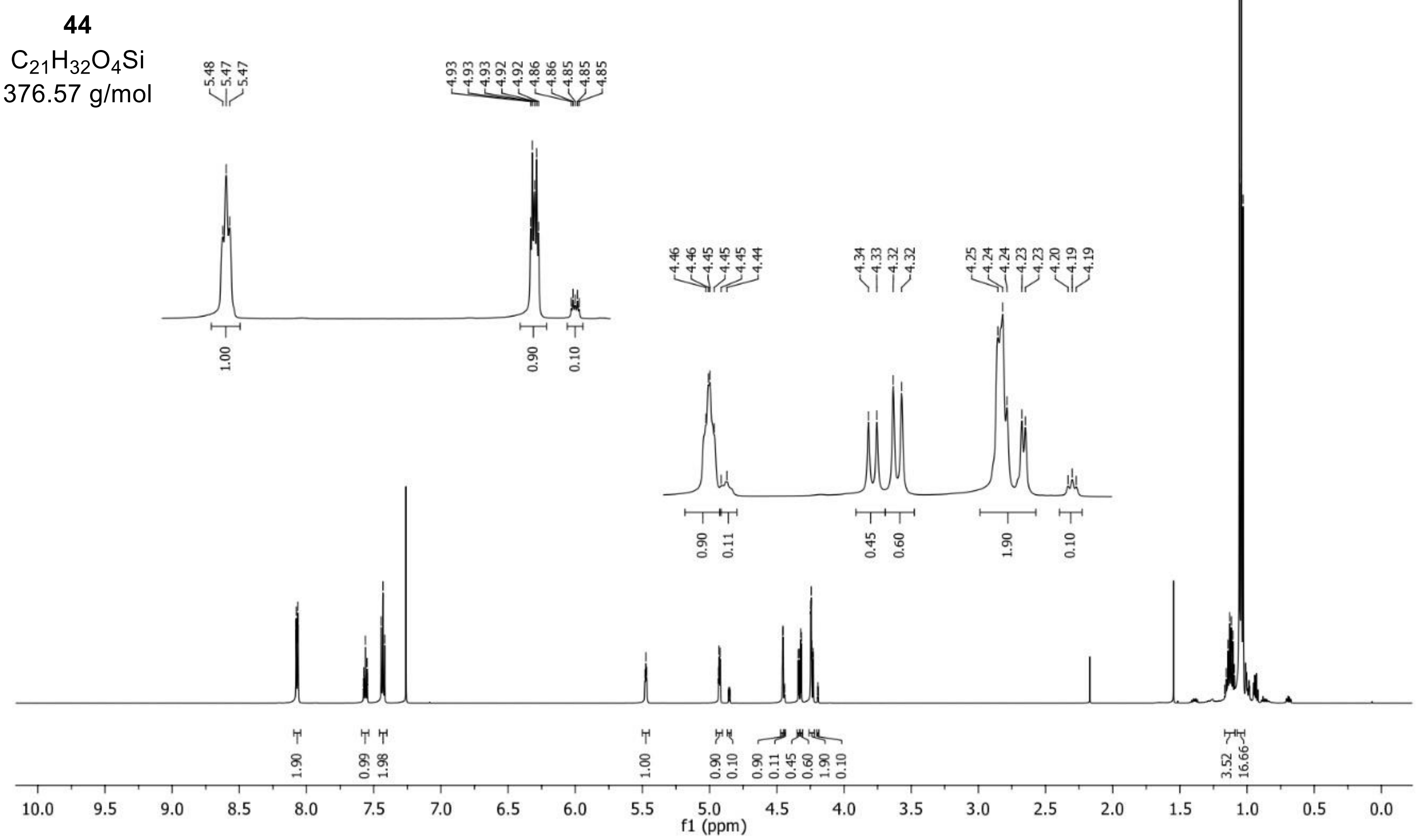

^{13}C NMR (151 MHz, $CDCl_3$) of 2,5-anhydro-4-*O*-benzoyl-1-deoxy-3-*O*-triisopropylsilyl-D-*erythro*-pent-1-entiol (44)

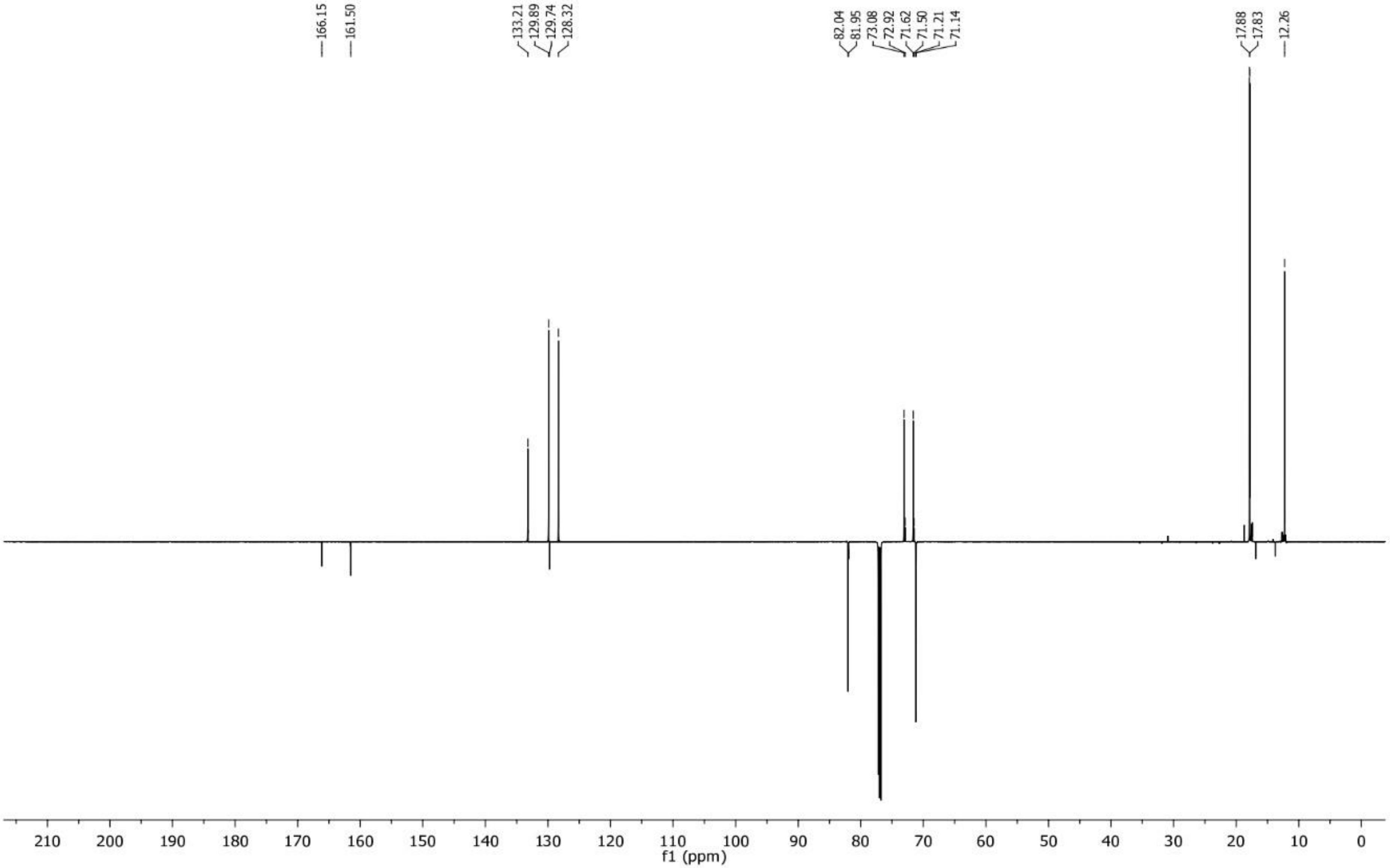

^{1}H NMR (600 MHz, CDCl$_3$) of 4-*O*-benzoyl-3-*O*-triisopropylsilyl-D-*ribulose* (45)

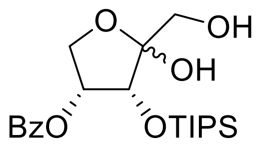

45

$C_{21}H_{34}O_6Si$

410.58 g/mol

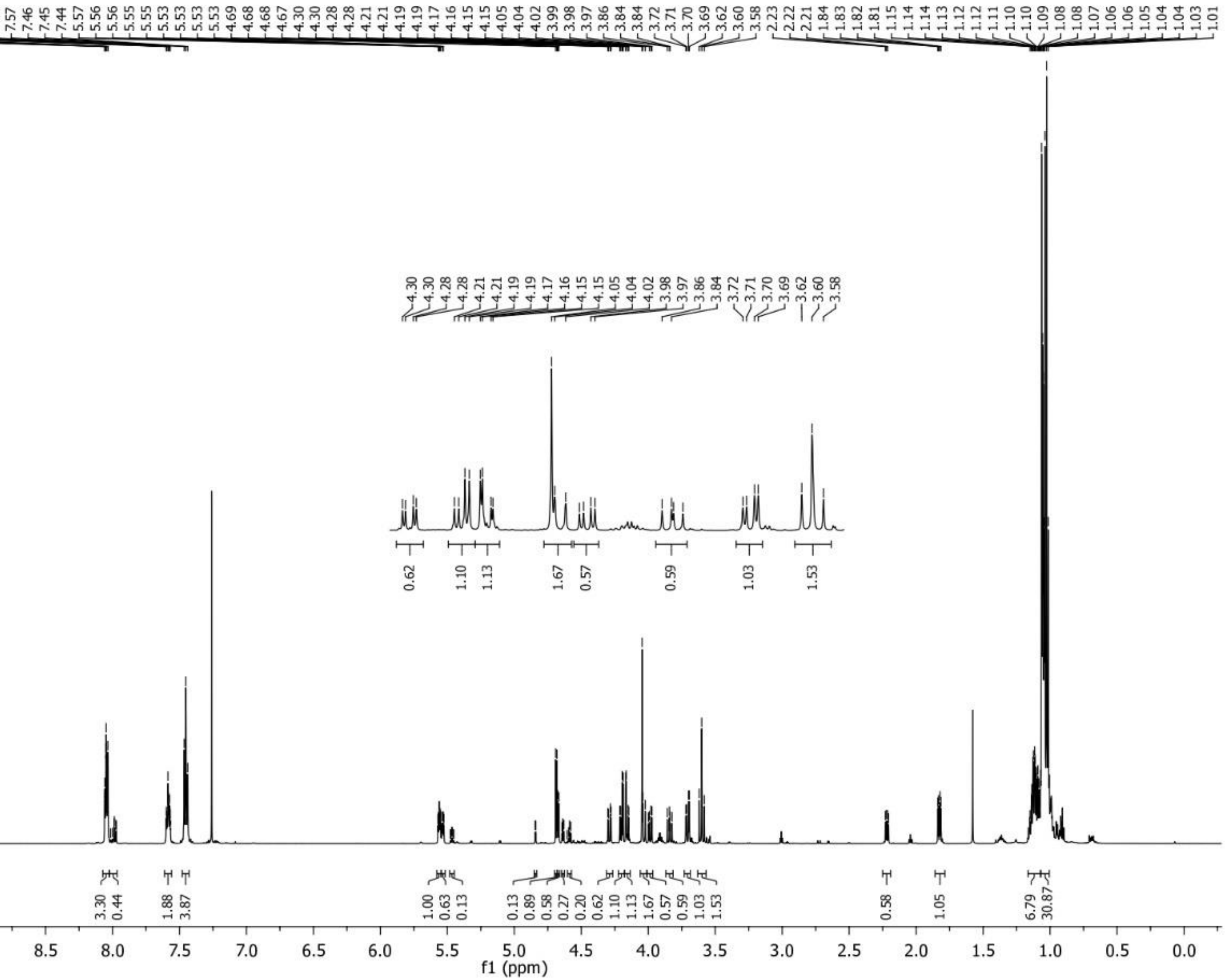

^{13}C NMR (151 MHz, CDCl$_3$) of 4-*O*-benzoyl-3-*O*-triisopropylsilyl-D-*ribulose* (45)

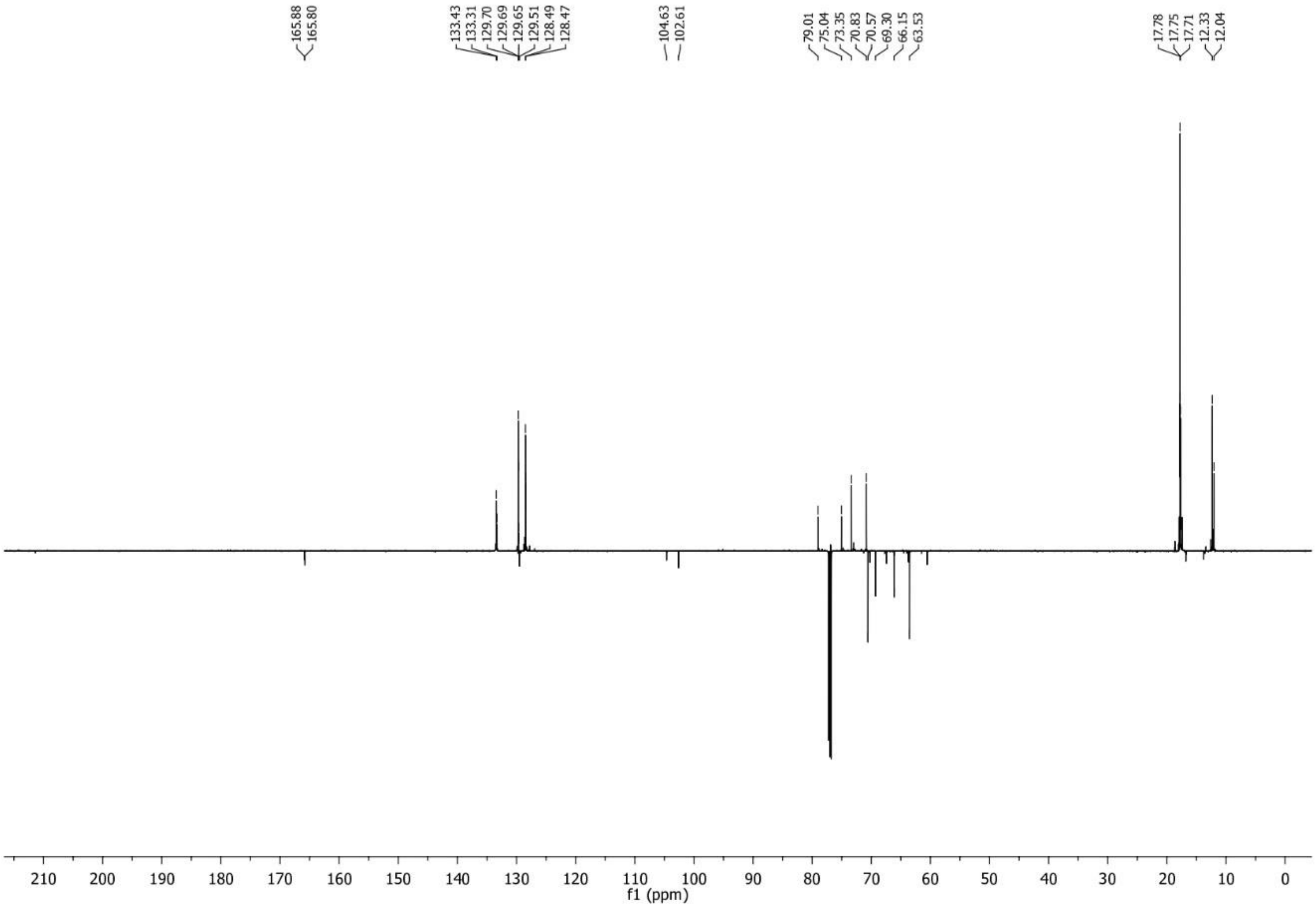

^{1}H NMR (600 MHz, CDCl₃) of 4-*O*-benzoyl-1,2-*O*-isopropylidene-3-*O*-triisopropylsilyl-D-*ribulose* (46)

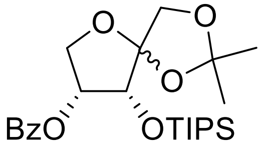

46

$C_{24}H_{38}O_6Si$

450.65 g/mol

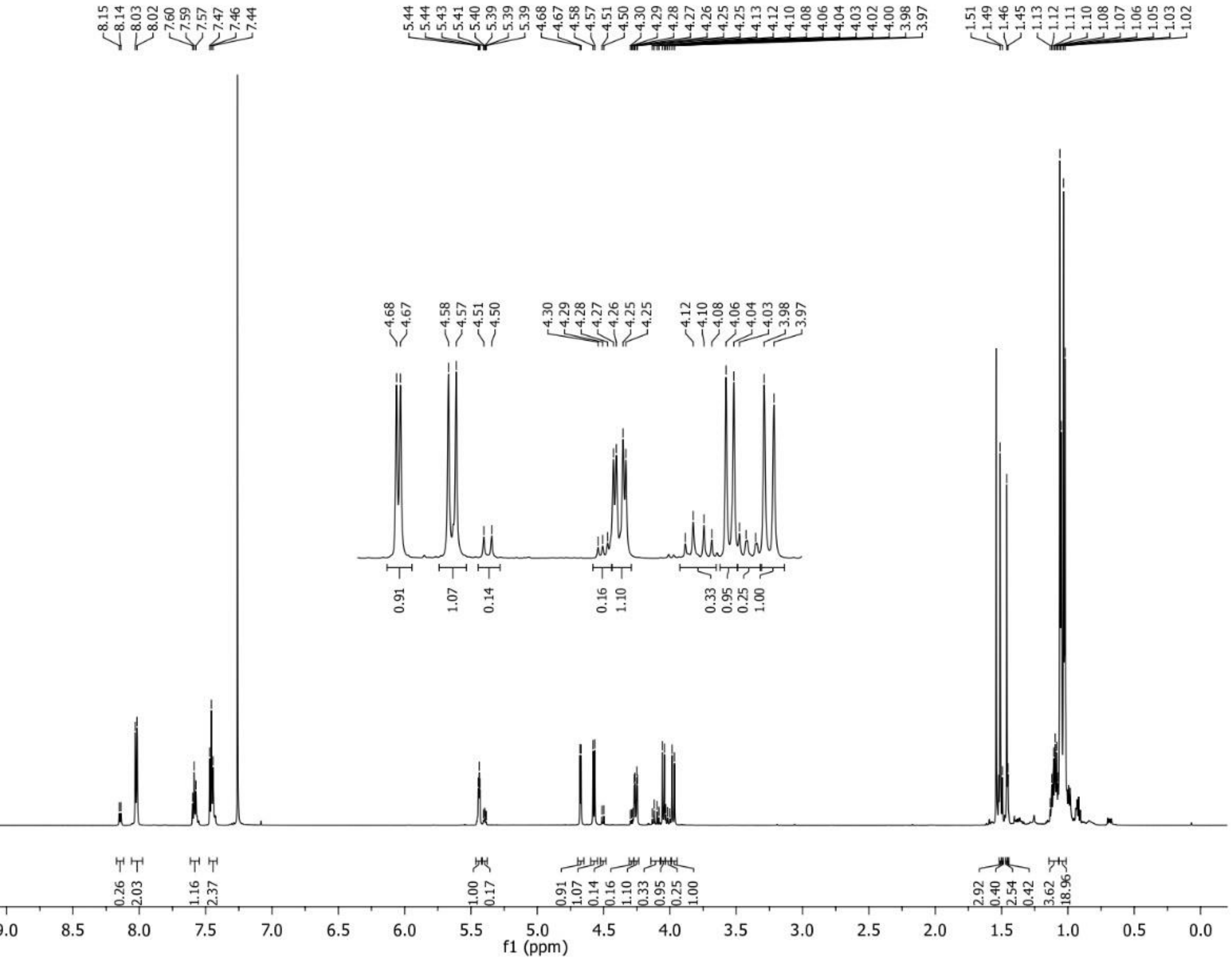

^{13}C NMR (151 MHz, CDCl$_3$) of 4-*O*-benzoyl-1,2-*O*-isopropylidene-3-*O*-triisopropylsilyl-D-*ribulose* (46)

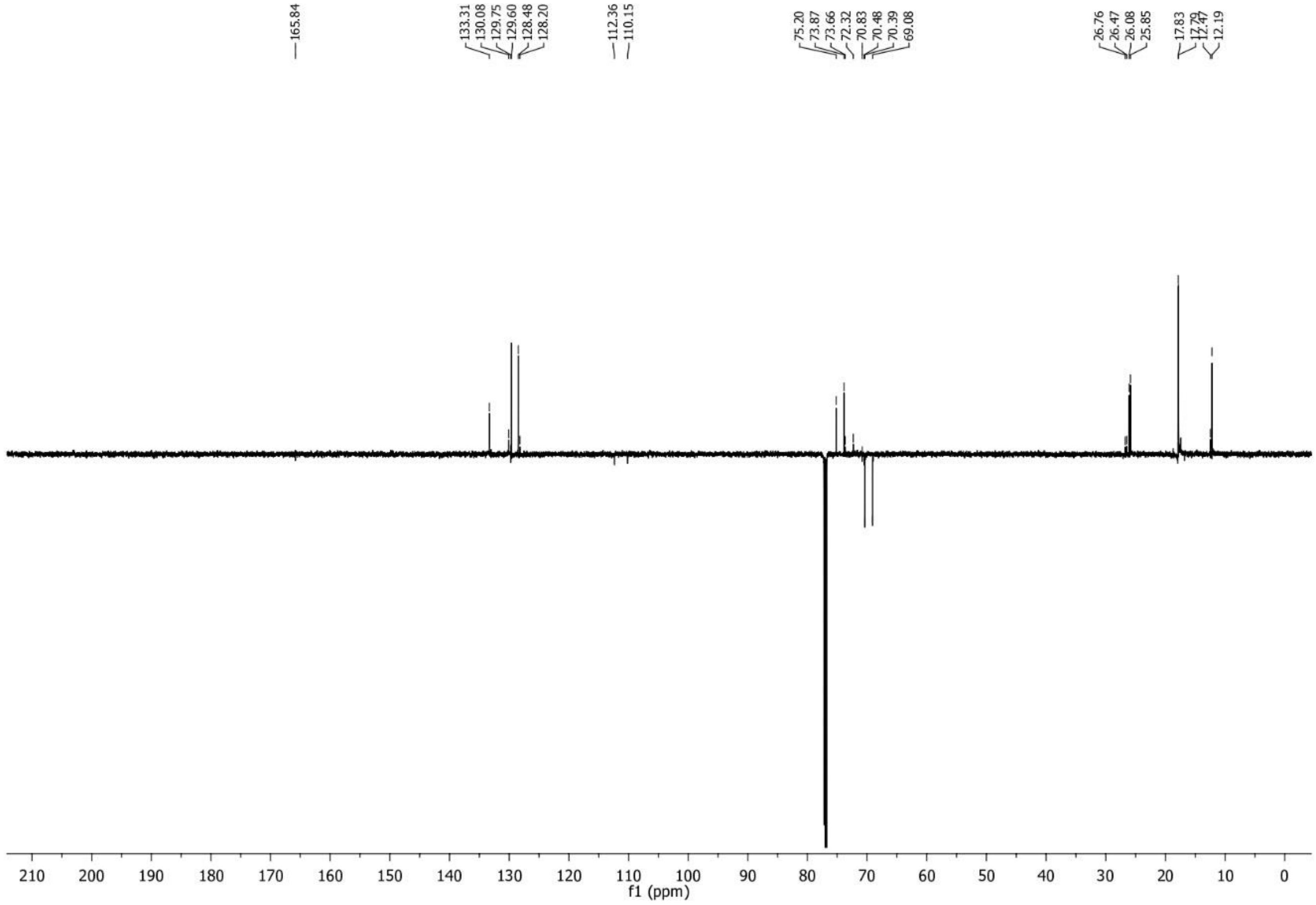

^{1}H NMR (600 MHz, $CDCl_3$) of 4-*O*-benzoyl-1,2-*O*-isopropylidene-D-*ribulose* (47)

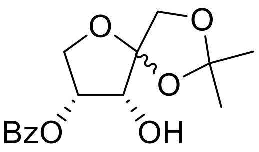

47

$C_{15}H_{18}O_6$

294.30 g/mol

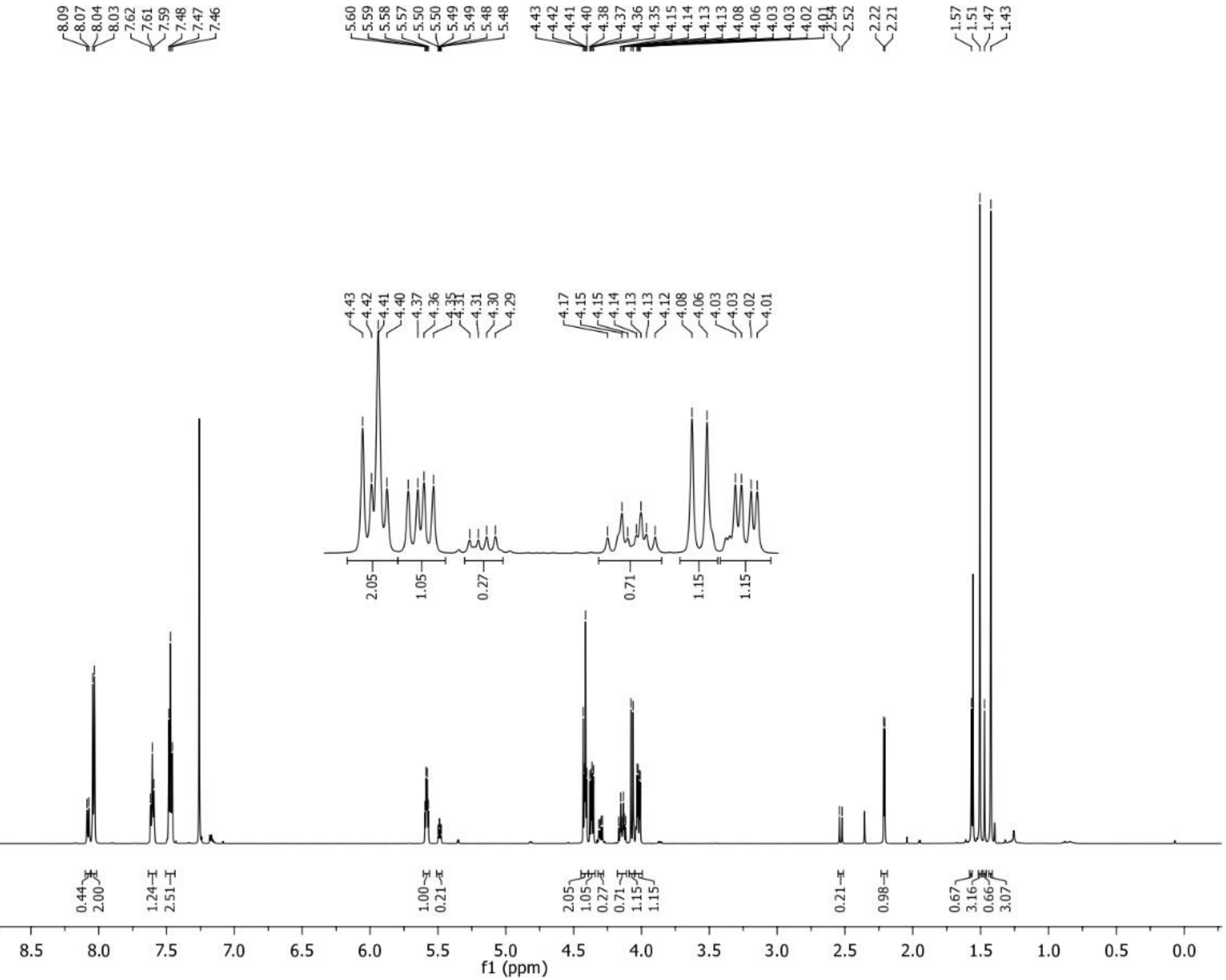

^{13}C NMR (151 MHz, $CDCl_3$) of 4-*O*-benzoyl-1,2-*O*-isopropylidene-D-*ribulose* (47)

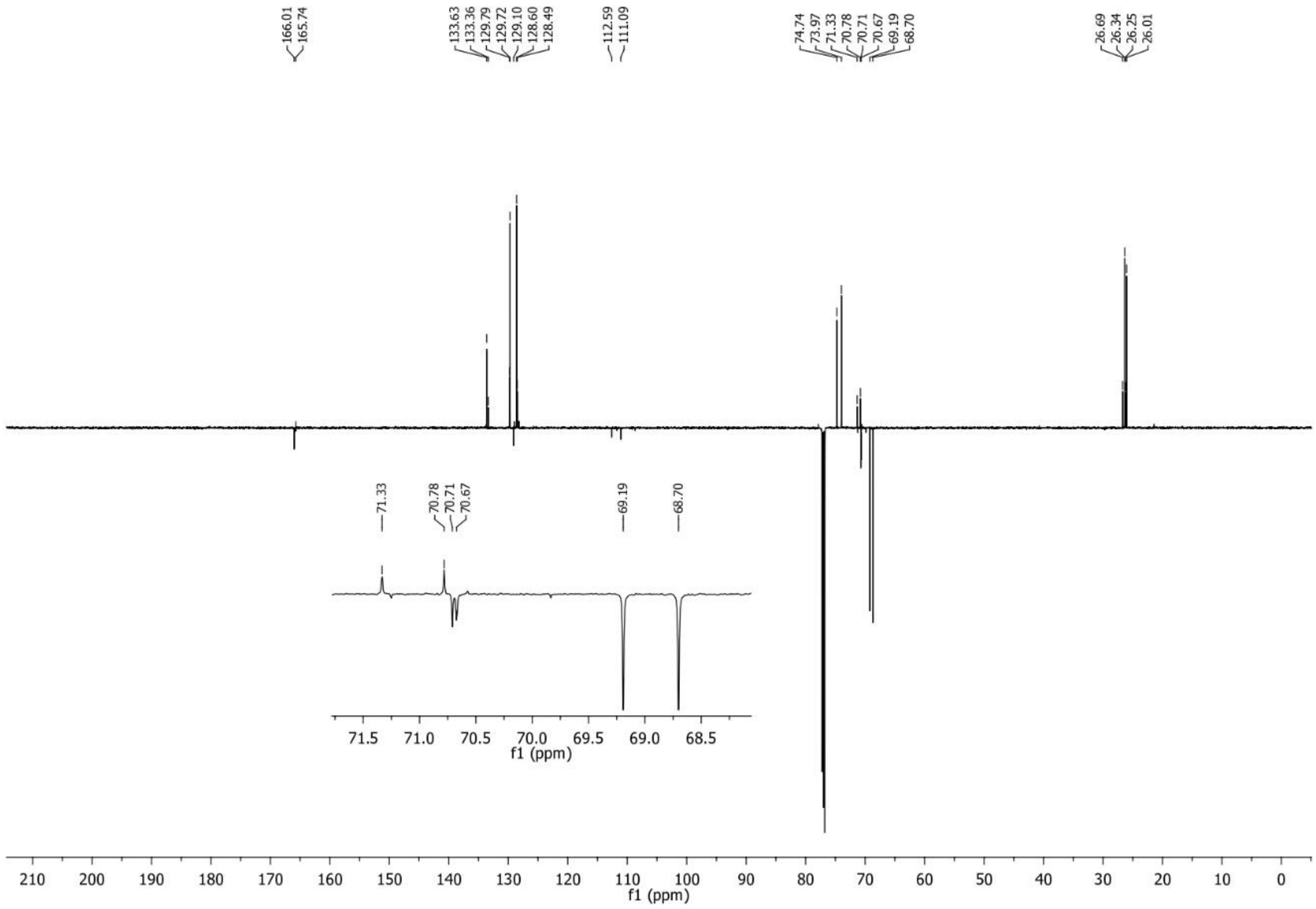

^{1}H NMR (700 MHz, CDCl₃) of 4-*O*-benzoyl-1,2-*O*-isopropylidene-3-*O*-trifluoromethanesulfonyl-D-*ribulose* (48)

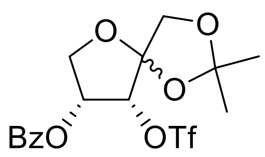

48

$C_{16}H_{17}F_3O_8S$

426.36 g/mol

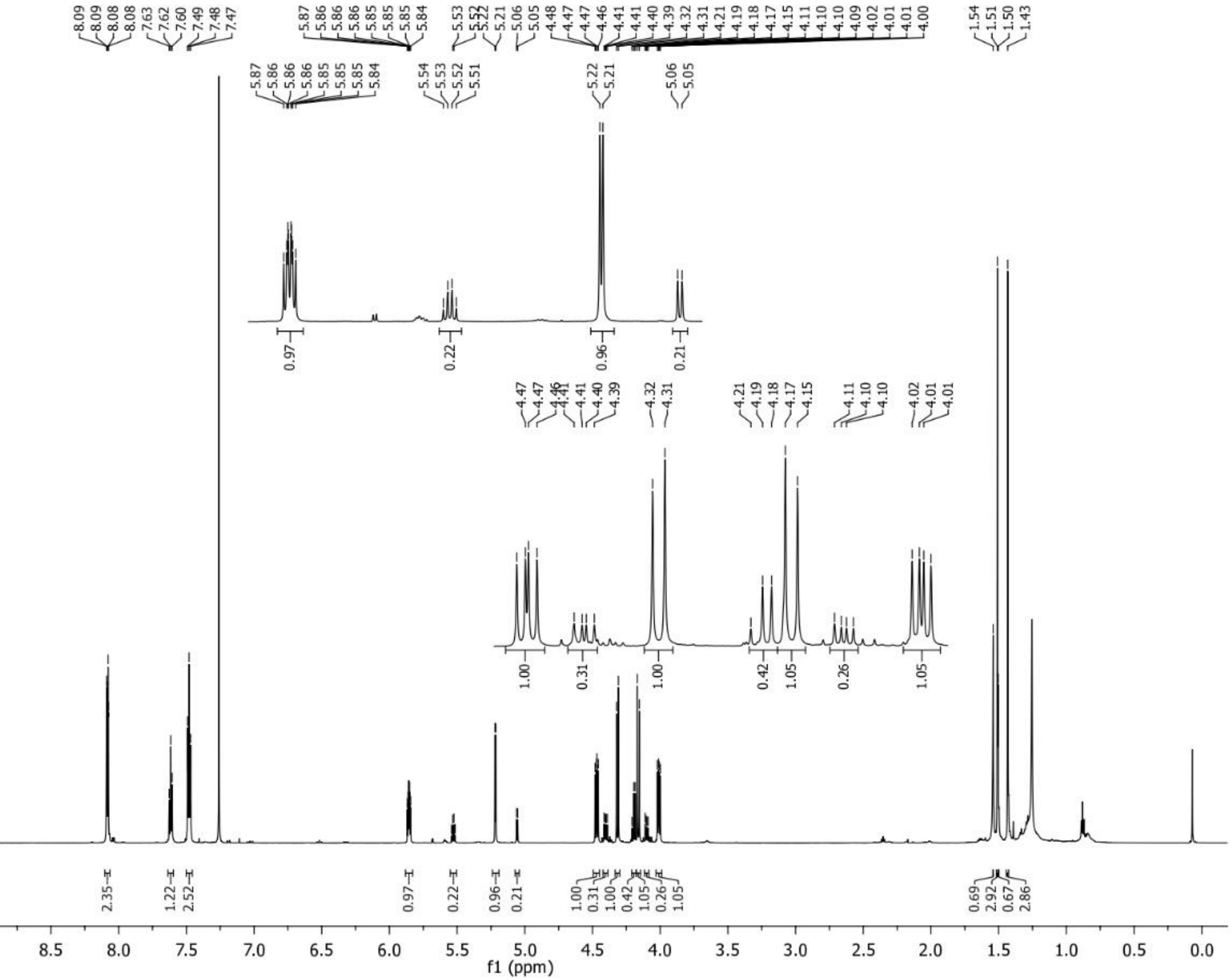

^{13}C NMR (151 MHz, CDCl$_3$) of 4-*O*-benzoyl-1,2-*O*-isopropylidene-3-*O*-trifluoromethanesulfunyl-D-*ribulose* (48)

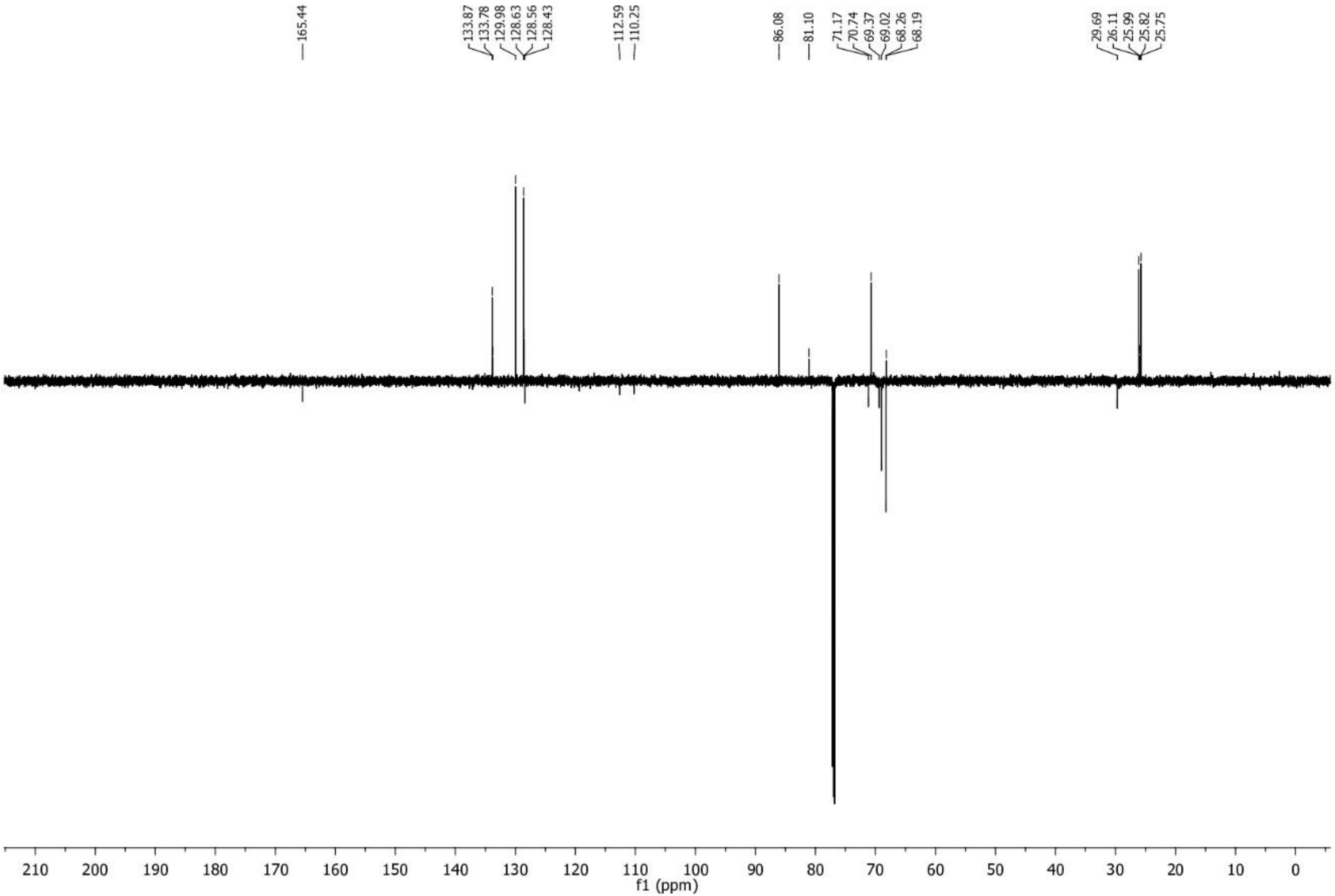

Supplement: Supplementary file 1 — Supporting Information [file EJOC-26-0-s001.pdf]
